# Proteomics of REPLICANT perfusate detects changes in the metastatic lymph node microenvironment

**DOI:** 10.1038/s41523-021-00227-7

**Published:** 2021-03-05

**Authors:** Julia Stevenson, Rachel Barrow-McGee, Lu Yu, Angela Paul, David Mansfield, Julie Owen, Natalie Woodman, Rachael Natrajan, Syed Haider, Cheryl Gillett, Andrew Tutt, Sarah E. Pinder, Jyoti Choudary, Kalnisha Naidoo

**Affiliations:** 1 The Breast Cancer Now Toby Robins Research Centre, The Institute of Cancer Research, London, UK; 2 Division of Cancer Biology, The Institute of Cancer Research, London, UK; 3 Division of Radiotherapy and Imaging, The Institute of Cancer Research, London, UK; 4 King’s Health Partners Cancer Biobank, Guy’s Comprehensive Cancer Centre, London, UK; 5 School of Cancer and Pharmaceutical Sciences, King’s College London, Guy’s Comprehensive Cancer Centre, London, UK; 6 Department of Cellular Pathology, Imperial College Healthcare NHS Trust, Charing Cross Hospital, London, UK

**Keywords:** Metastasis, Cancer models, Cancer microenvironment, Tumour biomarkers

## Abstract

In breast cancer (BC), detecting low volumes of axillary lymph node (ALN) metastasis pre-operatively is difficult and novel biomarkers are needed. We recently showed that patient-derived ALNs can be sustained ex-vivo using normothermic perfusion. We now compare reactive (tumour-free; *n* = 5) and macrometastatic (containing tumour deposits >2 mm; *n* = 4) ALNs by combining whole section multiplex immunofluorescence with TMT-labelled LC-MS/MS of the circulating perfusate. Macrometastases contained significantly fewer B cells and T cells (CD4^+^/CD8^+^/regulatory) than reactive nodes (*p* = 0.02). Similarly, pathway analysis of the perfusate proteome (119/1453 proteins significantly differentially expressed) showed that immune function was diminished in macrometastases in favour of ‘extracellular matrix degradation’; only ‘neutrophil degranulation’ was preserved. Qualitative comparison of the perfusate proteome to that of node-positive pancreatic and prostatic adenocarcinoma also highlighted ‘neutrophil degranulation’ as a contributing factor to nodal metastasis. Thus, metastasis-induced changes in the REPLICANT perfusate proteome are detectable, and could facilitate biomarker discovery.

## Introduction

Precise histopathological quantification of axillary tumour burden remains central to the management of patients with early-stage breast cancer (BC)^1^. This volumetric assessment of metastatic nodal disease is determined by counting the total number of metastatic axillary lymph nodes (ALNs), and measuring the size of the largest tumour deposit^1^. Recent efforts to delineate when it is safe to leave metastatic ALNs in situ, obviating the risks associated with a surgical ALN dissection (ALND), have proved controversial^1–5^. At a biological level, we have yet to determine at which size/volume a tumour deposit ‘switches off’ the immune response in an ALN, facilitating tumour growth and spread. In other words, how tumour biology and intra-tumour heterogeneity (ITH) affect ALN colonisation is unclear^6^.

This uncertainty is confounded by the fact that while preoperative imaging can reliably quantify high volumes of axillary disease, detecting smaller amounts is difficult, even when coupled with a needle-biopsy^7^. Furthermore, no reliable predictive biomarkers of axillary tumour burden exist at present. This is partly because every surgically excised ALN has to be formalin-fixed and paraffin-wax embedded (FFPE) for diagnosis and treatment planning^1,8^. As such, obtaining human nodal tissue for biomarker discovery and validation is difficult.

Although the liquid biopsy overcomes these issues of access and sample quantity, the complexity of blood has proved challenging for biomarker discovery^9^. Exactly how much nodal disease is required for circulating tumour DNA (ctDNA) to become detectable has yet to be determined. Interestingly, it has been shown that ctDNA can be identified pre-operatively in treatment naïve patients with more than three metastatic ALNs, and that levels drop following ALN removal^10^. However, since cell death or ‘shedding’ is required to release ctDNA, it could be argued that ‘non-shedding’ tumours might escape detection by this method altogether^11^. For these reasons, monitoring ctDNA in early-stage cancer is not recommended currently^11^.

With regard to proteomics, there is little concordance between the few studies that have tried to identify biomarkers of axillary disease in human serum/plasma samples^12–17^. Similarly, human tissue studies comparing the proteomes of primary BC to matched ALN metastases have yielded disparate data^18–23^. On the whole, ALN metastases have been shown to be similar to the primary BC at a protein level but minimal overlap was seen between studies. No studies thus far have compared the protein expression of reactive to metastatic ALN tissue.

Intriguingly, lymph seems to contain higher concentrations of circulating biomarkers, particularly in the early stages of metastasis^24^. Proteomic studies have shown that lymph reflects the pathophysiology of the tissue from which it derives^25–27^, and has recently been shown to be relevant to melanoma biomarker discovery and stage prediction^24^. To our knowledge, no such studies have been performed on lymphatic exudate from BC patients undergoing an ALND however.

In the ‘REPLICANT’ study, we recently showed that human ALNs from BC patients can be sustained ex vivo for scientific investigation using normothermic perfusion^8^. Herein, we characterise the proteome of the circulating fluid collected from these perfused ALNs (‘perfusate’) using Tandem Mass Tag (TMT) labelled mass spectrometry (MS)-based shotgun proteomics, and show that it can discriminate between reactive (tumour-free) and macrometastatic ALNs (i.e. containing a tumour deposit >2 mm in maximal dimension).

## Results

### Immune cells decrease significantly as tumour grows within an ALN

**Fig. 1:**
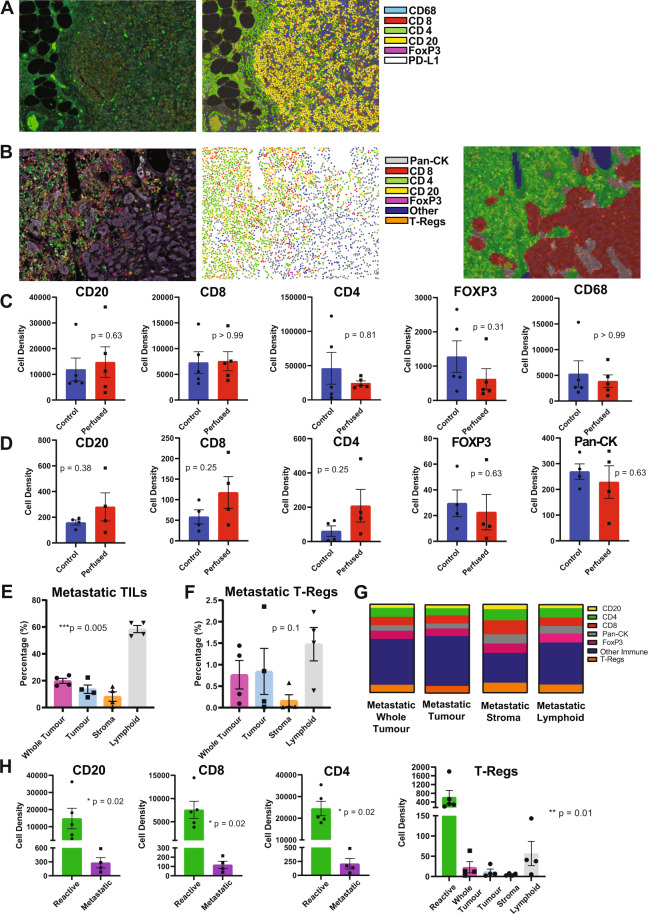
Multiplex immunofluorescence of REPLICANT axillary lymph nodes (ALNs). Reactive (*n* = 5) and macrometastatic (*n* = 4) ALNs were fluorescently stained for CD8, CD4, CD20, FoxP3 and PD-L1; reactive nodes, for CD68 in addition; metastatic nodes, additionally for pan-cytokeratin (pan-CK). Representative images of a reactive (**A**) and macrometastatic (**B**) node are shown (x20 magnification; field of view: 670 µm × 500 µm). From left to right, the first panels show immunofluorescent staining; second panels show cell phenotype maps, which were generated algorithmically. In metastatic nodes, the tissue also was segmented (third panel in **B**) into areas containing mostly cancer cells (‘tumour’; red), stromal regions (‘stroma’; light grey), and areas comprising mainly lymphoid cells (‘lymphoid’; green) for tumour infiltrating lymphocyte (mTILs) and PD-L1 analysis. No statistically significant differences in immune composition were seen between control (fixed at baseline) and perfused nodes (reactive nodes are shown in **C**, metastatic in **D**; cell density = total cells/mm^2^; Wilcoxon test). Subsequent analysis was therefore done on perfused ALNs only. The percentage of mTILs across the whole tissue section of metastatic ALNs (‘whole tumour’), as well as in ‘tumour’, ‘stroma’, and ‘lymphoid’ areas, is shown in **E** (*n* = 4). The differences between these regions was statistically significant (*p* < 0.0001; Kruskal-Wallis test). The regional distribution of regulatory T cells (T-regs) did not differ significantly however (**F**). The co-localisation of PD-L1 (average signal intensity) with other cell markers is shown in **G**. 49% (whole tumour) and 30–54% (regional) of the PD-L1 staining did not co-localise with any of the other markers that we had stained for (‘other immune’). Most of the remaining signal was present on T-lymphocytes (36% (whole tumour); 32% (tumour); 46% (stroma); and 37% (lymphoid)), with cancer cells contributing between 5 and 10%, and B cells <5%, to overall intensity. **H** The average cell density of CD20 ^+^ B cells (*p* = 0.02), CD8 ^+^ T cells (*p* = 0.02), CD4 ^+^ T cells (*p* = 0.02) and T-regs (*p* = 0.02) was significantly decreased in nodes replaced by macrometastases (latter, Kruskal–Wallis; former three, Mann–Whitney). (Graphs show mean with standard error of the mean (SEM)).

All of the ALNs collected during the study (10 perfused and 10 baseline control) underwent multiplex immunofluorescence (MIF). Representative images of a reactive (Fig. 1A) and a macrometastatic ALN (Fig. 1B) are shown (×20 magnification; field of view: 670 µm × 500 µm). The first panels (left to right) show MIF staining, while second panels show the cell phenotype maps that were generated algorithmically from the MIF images. In addition to analysing cell density (i.e. total cells per mm^2^) over the whole tissue section, the tissue in metastatic nodes was segmented (third panel in Fig. 1B) into areas containing mostly cancer cells (‘tumour’; red), stromal regions (‘stroma’; light grey), and areas comprising mainly lymphoid cells (‘lymphoid’; green). The node containing a micrometastasis (i.e. a tumour deposit measuring 0.2–2 mm) was excluded from further analysis (including proteomics) since the tumour cells had cut out after sectioning, thus confounding evaluation. Thus, nine ALNs were analysed in total (i.e. five reactive and four macrometastatic).

Although some quantitative differences in immune composition were seen between control and perfused ALNs, none reached statistical significance (reactive nodes (*n* = 5) are shown in Fig. 1C and macrometastatic nodes (*n* = 4) are shown in Fig. 1D). These data provide further evidence that perfusion does not appear to alter the tumour-immune microenvironment within ALNs^8^, and therefore all subsequent analysis was done on perfused ALNs only.

We calculated the percentage of tumour infiltrating lymphocytes in the metastatic tumour (mTILs; Fig. 1E)^28^, in the whole section and in the three regions described above. ‘Lymphoid’ areas, unsurprisingly, had the highest concentration of mTILs, while the lowest concentration was seen within ‘tumour’ foci. Importantly, the difference in mTILs between the compartments was statistically significant (*p* < 0.0001), suggesting that stromal mTILs might not be a surrogate for whole tumour mTILs in metastatic nodes. Regulatory T cells (T-regs) are an immunosuppressive subset of CD4^+^ T cells which also express FOXP3^29^. These cells can dampen the anticancer immune response, promoting tumour growth and progression; they also express immune-checkpoint molecules and, as such, are the target of immune checkpoint inhibitor therapies^29^. We therefore evaluated their distribution in macrometastatic ALNs. T-regs distribution followed the pattern of total mTIL distribution in the various compartments, but did not reach statistical significance (Fig. 1F).

The ubiquitous co-expression of PD-L1 made cell density quantification difficult. To overcome this, we calculated the average signal intensity for each cell type within each macrometastatic node (Fig. 1G). As can be seen, 49% (whole tumour) and 30–54% (regional) of the PD-L1 staining did not co-localise with any of the other markers that we had stained for; these are probably macrophages or dendritic cells^30^. Most of the remaining signal was present on T-lymphocytes (36% (whole section) and 32–46% (regional)), with cancer cells contributing between 5 and 10%, and B cells <5%, to overall intensity. Furthermore, the signal distribution was consistent in each area quantified (Representative images of PD-L1 MIF are shown in Supplementary Fig. 1).

Finally, we compared the immune composition of reactive to metastatic ALNs (Fig. 1H). The average cell density of B cells (*p* = 0.02), cytotoxic T cells (*p* = 0.02); T-helper cells (*p* = 0.02) and T-regs (*p* = 0.02) decreased significantly when macrometastases were present. This reflects the fact that nodal architecture is destroyed and replaced by metastatic tumour during ALN colonisation^6^.

### The perfusate proteome reflects the pathophysiology of perfused ALNs

**Fig. 2:**
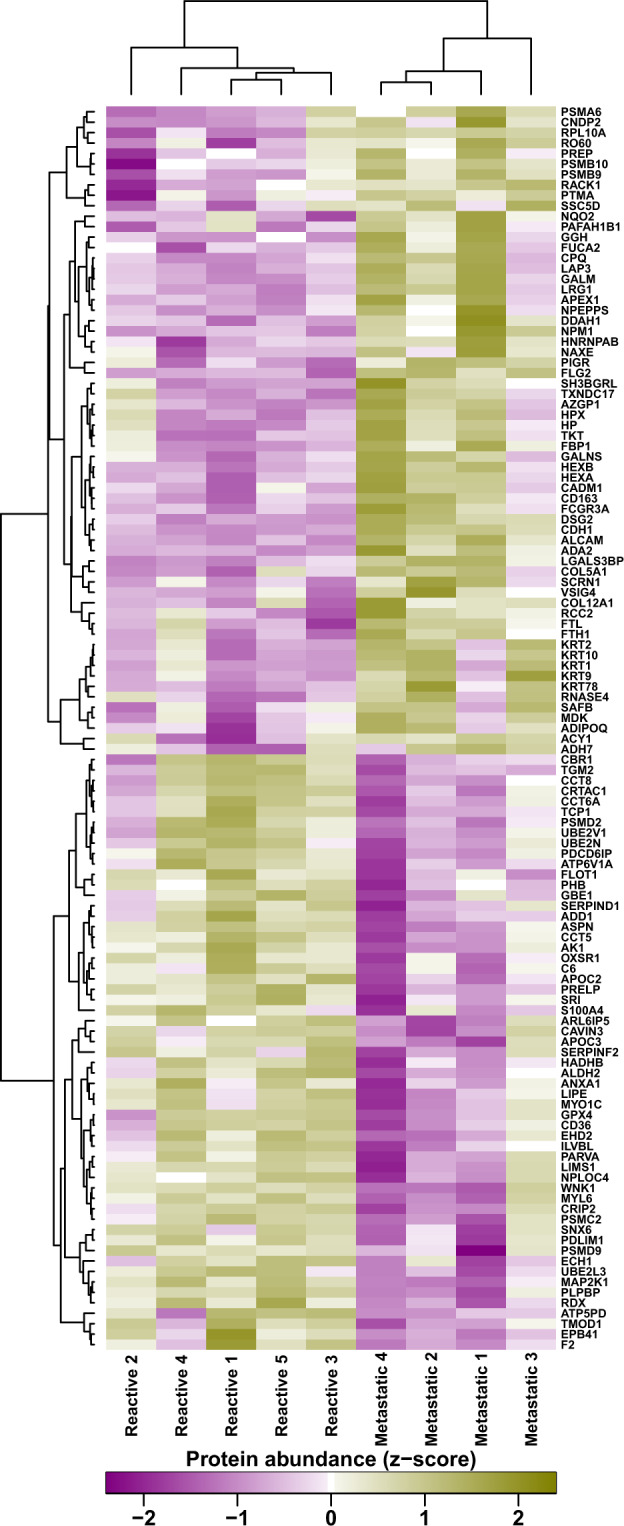
Hierarchical cluster analysis of the 119 significantly differentially expressed proteins separates reactive from metastatic nodes. A heat map showing hierarchical clustering of the nine perfusate samples (taken from nine different patients). A clear separation of reactive and metastatic nodes is seen.

Proteomic analysis of the perfusate samples (*n* = 9) identified 1453 proteins in total (Supplementary Table 1). Of these, 119 (8%) were significantly differentially expressed (DE) between reactive and metastatic samples (Table 1; *p* ≤ 0.05). Hierarchical cluster analysis of these 119 significantly differentially expressed proteins (DEP; Fig. 2) showed a clear separation of reactive and metastatic samples. This suggests that certain biological features within metastatic nodes are not only distinctive, but shared across different patients.

**Table 1:** 119 significantly differentially expressed proteins between reactive (*n* = 5) and metastatic (*n* = 4) perfusate.

Uniprot ID	Description	# Unique peptides	Reactive_AVE_	Metastatic_AVE_	log2ratio	*T*-test
P02655	Apolipoprotein C-II	5	153.72	32.83	−2.23	0.03
P21980	Protein-glutamine gamma-glutamyltransferase 2	11	144.44	44.45	−1.70	0.02
P00568	Adenylate kinase isoenzyme 1	8	141.80	47.70	−1.57	0.04
P68036	Ubiquitin-conjugating enzyme E2 L3	2	141.16	48.55	−1.54	0.01
A1L0T0	Acetolactate synthase-like protein	2	141.06	48.73	−1.53	0.01
Q9BXN1	Asporin	3	140.36	49.55	−1.50	0.001
Q13404	Ubiquitin-conjugating enzyme E2 variant 1	2	139.68	50.43	−1.47	0.05
P51888	Prolargin	5	139.20	50.98	−1.45	0.005
P02656	Apolipoprotein C-III	2	139.16	51.00	−1.45	0.03
P35611	Alpha-adducin	11	138.52	51.85	−1.42	0.04
P40227	T-complex protein 1 subunit zeta	5	137.98	52.58	−1.39	0.05
O00159	Unconventional myosin-Ic	13	136.70	54.08	−1.34	0.03
Q05469	Hormone-sensitive lipase	12	136.52	54.33	−1.33	0.01
P16671	Platelet glycoprotein 4	6	136.50	54.35	−1.33	0.02
P55084	Trifunctional enzyme subunit beta, mitochondrial	5	136.20	54.70	−1.32	0.02
O75955	Flotillin-1	4	135.58	55.58	−1.29	0.04
P35232	Prohibitin	2	159.43	65.55	−1.28	0.004
P50990	T-complex protein 1 subunit theta	11	134.98	56.30	−1.26	0.03
P52943	Cysteine-rich protein 2	3	134.94	56.38	−1.26	0.03
P48643	T-complex protein 1 subunit epsilon	9	134.48	56.88	−1.24	0.01
Q8WUM4	Programmed cell death 6-interacting protein	9	134.40	56.98	−1.24	0.01
O00151	PDZ and LIM domain protein 1	8	134.34	57.08	−1.23	0.03
P17987	T-complex protein 1 subunit alpha	11	134.24	57.20	−1.23	0.03
O75947	ATP synthase subunit d, mitochondrial	3	133.98	57.53	−1.22	0.04
Q9NQ79	Cartilage acidic protein 1	3	133.82	57.73	−1.21	0.03
Q02750	Dual specificity mitogen-activated protein kinase kinase 1	3	133.34	58.33	−1.19	0.003
P05091	Aldehyde dehydrogenase, mitochondrial	11	133.26	58.40	−1.19	0.02
P38606	V-type proton ATPase catalytic subunit A	3	133.14	58.60	−1.18	0.02
Q13200	26S proteasome non-ATPase regulatory subunit 2	6	132.62	59.20	−1.16	0.05
Q9NVD7	Alpha-parvin	6	131.72	60.35	−1.13	0.05
P61088	Ubiquitin-conjugating enzyme E2 N	5	131.66	60.43	−1.12	0.05
Q9NZN4	EH domain-containing protein 2	19	131.20	60.98	−1.11	0.04
P35998	26S proteasome regulatory subunit 7	4	130.98	61.25	−1.10	0.02
O75915	PRA1 family protein 3	4	130.18	62.33	−1.06	0.04
O95747	Serine/threonine-protein kinase OSR1	5	130.04	62.43	−1.06	0.04
P28289	Tropomodulin-1	10	130.02	62.48	−1.06	0.03
Q969G5	Caveolae-associated protein 3	5	129.74	62.83	−1.05	0.05
P16152	Carbonyl reductase [NADPH] 1	5	129.52	63.08	−1.04	0.05
Q9UNH7	Sorting nexin-6	2	129.18	63.53	−1.02	0.04
P11171	Protein 4.1	19	129.16	63.53	−1.02	0.05
P30626	Sorcin	7	128.06	64.95	−0.98	0.04
P26447	Protein S100-A4	3	127.96	65.05	−0.98	0.03
P04083	Annexin A1	15	127.46	65.68	−0.96	0.04
P48059	LIM and senescent cell antigen-like-containing domain protein 1	3	127.00	66.25	−0.94	0.02
Q04446	1,4-alpha-glucan-branching enzyme	3	125.16	68.53	−0.87	0.05
P36969	Phospholipid hydroperoxide glutathione peroxidase	2	124.38	69.50	−0.84	0.05
P35241	Radixin	9	123.96	70.05	−0.82	0.02
P05546	Heparin cofactor 2	10	123.84	70.20	−0.82	0.05
Q8TAT6	Nuclear protein localisation protein 4 homologue	2	123.80	70.28	−0.82	0.05
Q9H4A3	Serine/threonine-protein kinase WNK1	5	122.78	71.55	−0.78	0.03
P60660	Myosin light polypeptide 6	7	122.56	71.83	−0.77	0.04
P08697	Alpha-2-antiplasmin	6	122.54	71.88	−0.77	0.05
Q13011	Delta(3,5)-Delta(2,4)-dienoyl-CoA isomerase, mitochondrial	3	122.16	72.33	−0.76	0.03
O00233	26S proteasome non-ATPase regulatory subunit 9	5	121.20	73.50	−0.72	0.03
P13671	Complement component C6	17	119.66	75.40	−0.67	0.04
P00734	Prothrombin	23	117.14	78.60	−0.58	0.04
O94903	Pyridoxal phosphate homoeostasis protein	2	115.56	80.53	−0.52	0.004
P63244	Receptor of activated protein C kinase 1	8	83.76	120.30	0.52	0.01
P43034	Platelet-activating factor acetylhydrolase IB subunit alpha	11	83.36	120.80	0.54	0.05
P48147	Prolyl endopeptidase	7	80.98	123.78	0.61	0.05
Q99715	Collagen alpha-1(XII) chain	2	78.12	127.43	0.71	0.05
A1L4H1	Soluble scavenger receptor cysteine-rich domain-containing protein SSC5D	6	77.54	128.10	0.72	0.04
Q03154	Aminoacylase-1	5	77.18	128.53	0.74	0.05
P62906	60S ribosomal protein L10a	5	75.50	130.63	0.79	0.02
P02794	Ferritin heavy chain	7	72.98	133.78	0.87	0.02
Q15424	Scaffold attachment factor B1	5	72.98	133.80	0.87	0.05
P34096	Ribonuclease 4	2	72.52	134.35	0.89	0.04
P10155	60 kDa SS-A/Ro ribonucleoprotein	11	72.36	134.55	0.89	0.04
P60900	Proteasome subunit alpha type-6	12	71.68	135.45	0.92	0.05
Q9P258	Protein RCC2	2	70.92	136.35	0.94	0.04
P01833	Polymeric immunoglobulin receptor	4	70.64	136.73	0.95	0.006
Q9BRA2	Thioredoxin domain-containing protein 17	3	70.62	136.70	0.95	0.05
P55786	Puromycin-sensitive aminopeptidase	25	69.06	138.65	1.01	0.05
Q9Y646	Carboxypeptidase Q	3	68.80	139.00	1.01	0.02
Q12765	Secernin-1	6	68.36	139.53	1.03	0.03
Q08380	Galectin-3-binding protein	10	66.60	141.70	1.09	0.002
Q96KP4	Cytosolic non-specific dipeptidase	19	65.62	142.98	1.12	0.05
Q9BTY2	Plasma alpha-L-fucosidase	3	65.56	143.08	1.13	0.05
Q9BY67	Cell adhesion molecule 1	2	65.24	143.45	1.14	0.03
Q9Y279	V-set and immunoglobulin domain-containing protein 4	2	64.62	144.28	1.16	0.03
P02792	Ferritin light chain	8	64.00	145.00	1.18	0.03
P02790	Hemopexin	29	63.04	146.23	1.21	0.05
O94760	N(G),N(G)-dimethylarginine dimethylaminohydrolase 1	6	62.68	146.63	1.23	0.04
Q15848	Adiponectin	2	62.36	147.03	1.24	0.04
P16083	Ribosyldihydronicotinamide dehydrogenase [quinone]	6	61.62	147.98	1.26	0.04
P29401	Transketolase	28	61.58	148.03	1.27	0.03
Q14126	Desmoglein-2	6	61.34	148.35	1.27	0.0006
O75368	SH3 domain-binding glutamic acid-rich-like protein	9	60.98	148.78	1.29	0.04
Q92820	Gamma-glutamyl hydrolase	6	60.32	149.55	1.31	0.04
Q8NCW5	NAD(P)H-hydrate epimerase	5	59.88	150.18	1.33	0.04
P06748	Nucleophosmin	3	59.52	150.63	1.34	0.02
P40394	Alcohol dehydrogenase class 4 mu/sigma chain	2	57.40	153.28	1.42	0.05
Q86VB7	Scavenger receptor cysteine-rich type 1 protein M130	17	56.38	154.55	1.45	0.007
P35527	Keratin, type I cytoskeletal 9	21	54.94	156.30	1.51	0.04
P07686	Beta-hexosaminidase subunit beta	7	54.56	156.78	1.52	0.01
P34059	N-acetylgalactosamine-6-sulfatase	3	54.30	157.13	1.53	0.04
P28838	Cytosol aminopeptidase	20	52.70	159.15	1.59	0.03
P06865	Beta-hexosaminidase subunit alpha	5	52.08	159.88	1.62	0.02
P06454	Prothymosin alpha	2	50.02	162.48	1.70	0.005
P00738	Haptoglobin	14	49.14	163.58	1.73	0.05
P04264	Keratin, type II cytoskeletal 1	30	48.58	164.30	1.76	0.02
P08637	Low affinity immunoglobulin gamma Fc region receptor III-A	4	48.32	164.60	1.77	0.03
P25311	Zinc-alpha-2-glycoprotein	19	46.80	166.50	1.83	0.05
P20908	Collagen alpha-1(V) chain	3	46.76	166.50	1.83	0.02
P27695	DNA-(apurinic or apyrimidinic site) lyase	10	44.50	169.33	1.93	0.05
P35908	Keratin, type II cytoskeletal 2 epidermal	22	44.34	169.58	1.94	0.02
Q99729	Heterogeneous nuclear ribonucleoprotein A/B	2	44.24	169.70	1.94	0.05
P28065	Proteasome subunit beta type-9	3	43.40	170.70	1.98	0.01
Q8N1N4	Keratin, type II cytoskeletal 78	2	42.74	171.58	2.01	0.03
Q96C23	Aldose 1-epimerase	7	42.46	171.93	2.02	0.02
P13645	Keratin, type I cytoskeletal 10	24	41.14	173.58	2.08	0.01
Q5D862	Filaggrin-2	3	41.00	173.75	2.08	0.0004
P02750	Leucine-rich alpha-2-glycoprotein	6	40.84	173.95	2.09	0.02
P40306	Proteasome subunit beta type-10	3	39.42	175.73	2.16	0.02
Q9NZK5	Adenosine deaminase 2	5	36.10	179.85	2.32	0.03
P09467	Fructose-1,6-bisphosphatase 1	9	35.96	180.05	2.32	0.03
P21741	Midkine	2	35.18	181.03	2.36	0.04
Q13740	CD166 antigen	8	34.90	181.40	2.38	0.005
P12830	Cadherin-1	8	19.48	200.65	3.36	0.01

**Fig. 3:**
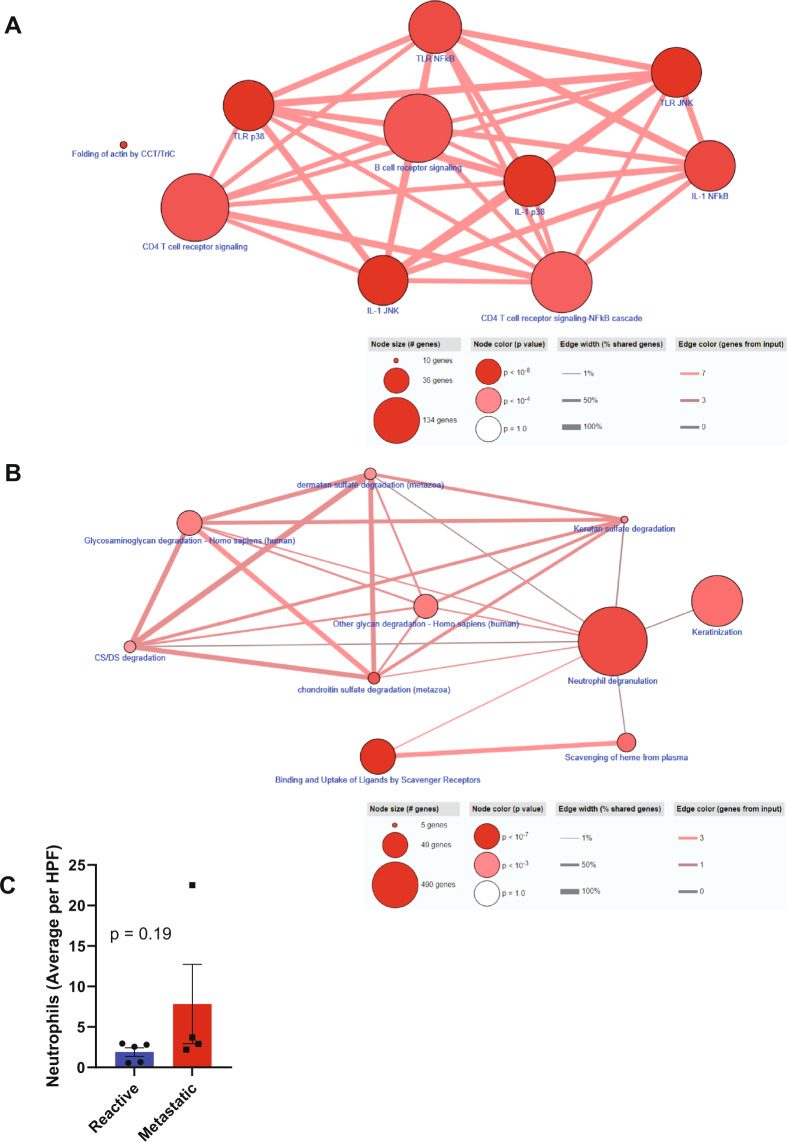
The perfusate proteome reflects the pathophysiology of the axillary lymph node (ALN) from which it derives. The 57 significantly up-regulated proteins in reactive ALNs (**A**; *n* = 5) and the 62 significantly up-regulated proteins in macrometastatic ALNs (**B**; *n* = 4) were subjected to pathway analysis in ConsensusPathDB. Reactive ALNs maintained immune function whilst this was lost in macrometastatic nodes, with the notable exception on ‘neutrophil degranulation’. The identification of ‘keratinisation’ in macrometastatic nodes reflects the presence of cancer cells within the node. The other pathways identified in these nodes related to extracellular matrix (ECM) degradation. Histological neutrophil counts (haematoxylin and eosin stained ALN tissue sections) are shown in (**C**; graph shows mean with standard error of the mean (SEM)).

The 57 significantly upregulated proteins in reactive ALNs (*n* = 5) and the 62 significantly up-regulated proteins in metastatic ALNs (*n* = 4) were subjected to pathway analysis in ConsensusPathDB^31^. The 10 most enriched pathways for both disease states are shown in Fig. 3A (reactive) and B (metastatic).

In reactive nodes, as expected, the 10 most common pathways identified all linked to immunity, both innate and adaptive. Conversely, in the metastatic perfusate samples, proteins reflecting active immune function were poorly represented, supporting the picture painted by MIF. Most of the pathways identified in these nodes relate to extracellular matrix (ECM) degradation. The ECM is known to regulate cell behaviour and differentiation in lymph nodes (LNs) in both health and disease, changing dynamically in response to injury^32–36^. The fact that ‘keratinisation’ was identified in the metastatic perfusate samples reflects the presence of tumour cells within the ALNs, since only epithelial cells contain keratin. These cells are not a normal LN constituent, and thus served as a good positive control. The only immune process identified in the metastatic perfusate samples was ‘neutrophil degranulation’.

Since ‘neutrophil degranulation’ had been identified as being significantly up-regulated in metastatic perfusate samples, we reviewed the haematoxylin and eosin (H&E) sections of the nine perfused ALNs^8^ to see if the absolute number of neutrophils was increased in metastatic nodes (Fig. 3C). Although there was a trend towards higher numbers of neutrophils in metastatic nodes, this did not reach statistical significance (*p* = 0.19).

Overall, these data suggest that the proteome reflects the pathophysiology of the perfused ALNs, and that a shift to ‘ECM degradation’ and ‘neutrophil degranulation’ can be used to infer when macrometastases are present or not.

### Analysing differences between reactive and metastatic perfusate samples reveals novel patterns of protein dysregulation

In order to see how the perfusate proteome compared to that of primary BC tissue samples, we mined TCGA samples which underwent proteomic analysis in the CPTAC study^37–39^. Qualitatively, 1361 of the 1453 perfusate proteins were present in the TCGA(CPTAC) dataset (i.e. 94% overlap; data not shown). However, since an average of 11,632 proteins/tumour were found in that study, the perfusate probably contains a fraction of the proteins expressed in primary BC^39^.

We then stratified the TCGA(CPTAC) samples into those from patients who had undergone a sentinel LN biopsy (SLNB) only (i.e. no/low axillary tumour burden); those who had also had a completion clearance (CC) (i.e. presumed higher axillary tumour burden than the SLNB group); and those who had had an upfront ALND (i.e. presumed high axillary tumour burden at diagnosis). The rationale for this was that the SLNB group would be similar to the reactive perfusate samples, whilst the CC/ALND groups would be comparable to the metastatic perfusate samples to varying degrees. We tested this hypothesis using the 10 most abundant proteins found in the reactive (namely APOC2, TGM2, AK1, UBE2L3, ILVBL, ASPN, UBE2V1, PRELP, APOC3 and ADD1) and metastatic (namely GALM, KRT10, FLG2, LRG1, PSMB10, ADA2, FBP1, MDK, ALCAM and CDH1) perfusate, respectively.

**Fig. 4:**
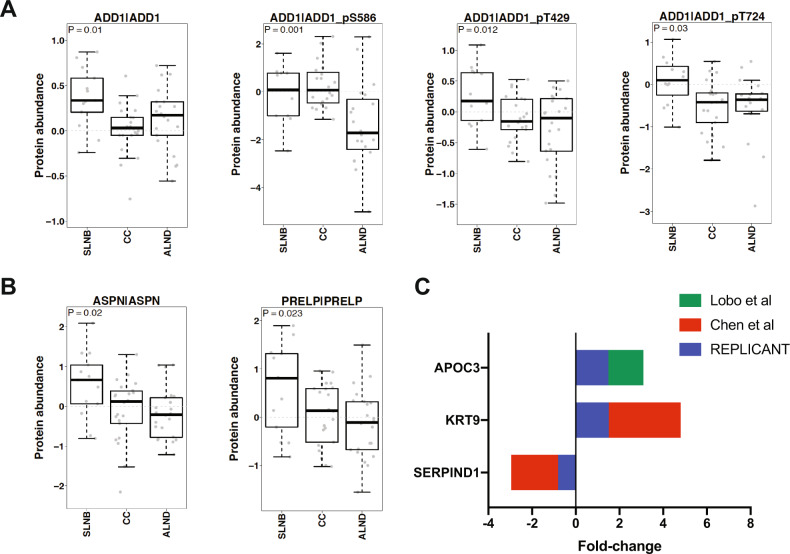
Comparison of the perfusate proteome to primary breast cancer (BC) and plasma proteomes yields novel data. We stratified the TCGA(CPTAC) primary BC proteomics data into three groups according to axillary tumour burden (ATB): those who had a sentinel lymph node biopsy only (SLNB; no/low ATB); those who had a completion clearance (CC; higher burden of ATB than the SLNB group); and those who had an axillary lymph node dissection, and high ATB, at diagnosis (ALND). Of the 10 most abundant reactive proteins, ADD1 (*p* = 0.01; **A**) and specific ADD1 phospho-isoforms; ASPN (*p* = 0.02; **B**) and PRELP (*p* = 0.023; **B**) were significantly up-regulated in the SLNB group, matching our data. No metastatic proteins were found to be significantly differentially expressed. Comparison of the perfusate proteome with BC plasma proteomic studies (**C**) showed minimal overlap, with APOC3 and KRT9 identified as up-regulated in metastatic nodes/perfusate, and SERPIND1 as up-regulated in reactive nodes/perfusate. (Graphs show median with interquartile range).

Eight reactive perfusate proteins were identified in the TCGA(CPTAC) samples, and the DE of three of the proteins between the clinical groups was as expected (Fig. 4A, B). ADD1 was significantly more abundant in patients in the SLNB group (*p* = 0.01). Since phosphoproteomics had been performed in that study, we could also identify which phospho-isoforms of ADD1 were significantly DE between the clinical groups (Fig. 4A); however, the implications of this are currently unknown. ASPN (*p* = 0.02) and PRELP (*p* = 0.023) were also significantly more abundant in patients in the SLNB group (Fig. 4B). The trends in expression for the other five reactive proteins are shown in Supplementary Fig. 2A (post-hoc analysis using Tukey’s honestly significantly difference (HSD) test is shown in Supplementary Table 2).

Eight metastatic proteins were identified in the TCGA(CPTAC) samples, but none were significantly DE between the clinical groups (Supplementary Fig. 2B**;** post-hoc analysis using Tukey’s HSD test is shown in Supplementary Table 2). CDH1 expression did increase as axillary tumour burden increased, but this did not reach significance (*p* = 0.081).

Closer inspection of the TCGA(CPTAC) data explained this disparity to a certain extent. Our assumption regarding the 13 patients in this study who had undergone an SLNB alone was correct—none had histopathological evidence of axillary disease following surgery. However, approximately 40% of the patients in the CC and ALND groups were staged as N0 following surgery^40^ i.e. all of the retrieved LNs were reactive, and showed no evidence of metastatic tumour on H&E staining. Thus, these two groups did not consist entirely of patients with a higher burden of axillary disease, and were not therefore entirely comparable with the metastatic perfusate samples.

The total proteome data for only one study comparing matched primary BC and ALN metastases was available/accessible for comparison^19^. Since this had used a gel-based method of protein separation, fewer proteins (135 in total) were identified; 86 of these were present in the perfusate proteome (64%; Supplementary Table 3). Quantitative analysis was not feasible for this study.

Comparisons were feasible for two of the above-mentioned BC plasma proteome studies; little overlap was seen between plasma and the perfusate proteome (Fig. 4C). SERPIND1, which was significantly up-regulated in the reactive perfusate samples, was also upregulated in node-negative Her2-positive BC patients^17^. KRT9 was significantly up-regulated in the metastatic perfusate samples; this was found to be up-regulated in node-positive Her2-positive patients previously^17^. Lobo et al. found APOC3 to be up-regulated in patients with stage I/II BC (i.e. patients with a low axillary tumour burden)^16^; this was significantly up-regulated in the reactive perfusate samples.

Thus, most proteins identified in the perfusate samples have been identified in primary BC tissue, but not in BC plasma, samples previously. Interestingly, the comparison of reactive to metastatic ALN perfusate samples generated different data to that which could be obtained by stratifying primary BC tissue samples according to nodal status.

### Neutrophil degranulation is repeatedly highlighted in LN metastasis, across cancers

To see if certain proteins are conserved during LN metastasis across different carcinomas, we qualitatively compared the perfusate proteome to proteomic studies analysing metastatic LNs from other cancers/sites.

**Fig. 5:**
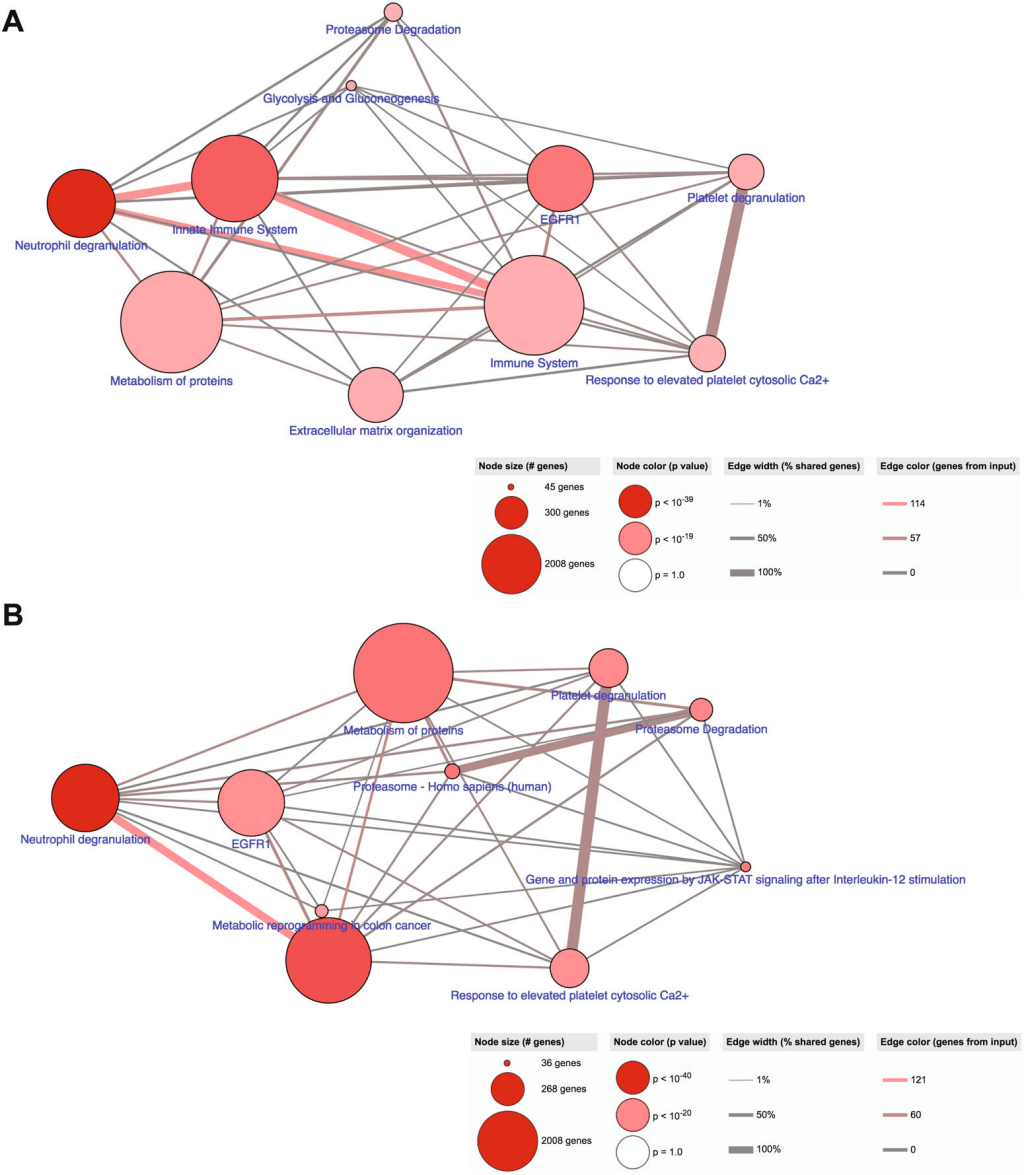
Neutrophil degranulation is recurrently highlighted when the REPLICANT proteome is compared to either pancreatic or prostatic cancer. Pathway analysis of the commonly expressed proteins obtained from an exclusive comparison of the perfusate proteome to node-positive pancreatic ductal adenocarcinoma (**A**; 515 commonly expressed proteins) or node-positive prostatic adenocarcinoma (**B**; 854 commonly expressed proteins) is shown. ‘Neutrophil degranulation’ was consistently identified as being important to lymph node metastasis.

We previously compared primary pancreatic ductal adenocarcinoma (PDAC) and matched LN metastases using laser capture microdissection coupled to multidimensional protein identification technology^41^. Interestingly, 515 of the 854 proteins (60%) identified in that study were also present in the perfusate proteome (Supplementary Table 4). The top 10 enriched pathways for these overlapping proteins are shown in Fig. 5A. Once again, ‘neutrophil degranulation’ was identified as the most enriched pathway between the two datasets.

Muller et al. compared prostate carcinoma tissue from patients with or without LN metastasis using label-free LC/MS/MS^42^. Qualitative comparison of that proteome to the perfusate protein revealed 854 commonly expressed proteins (48% of the 1750 total proteins identified; Supplementary Table 5). The top 10 enriched pathways for these overlapping proteins are shown in Fig. 5B; they are remarkably similar to those seen in Fig. 5A, with ‘neutrophil degranulation’ also being the most enriched pathway in this comparison.

**Fig. 6:**
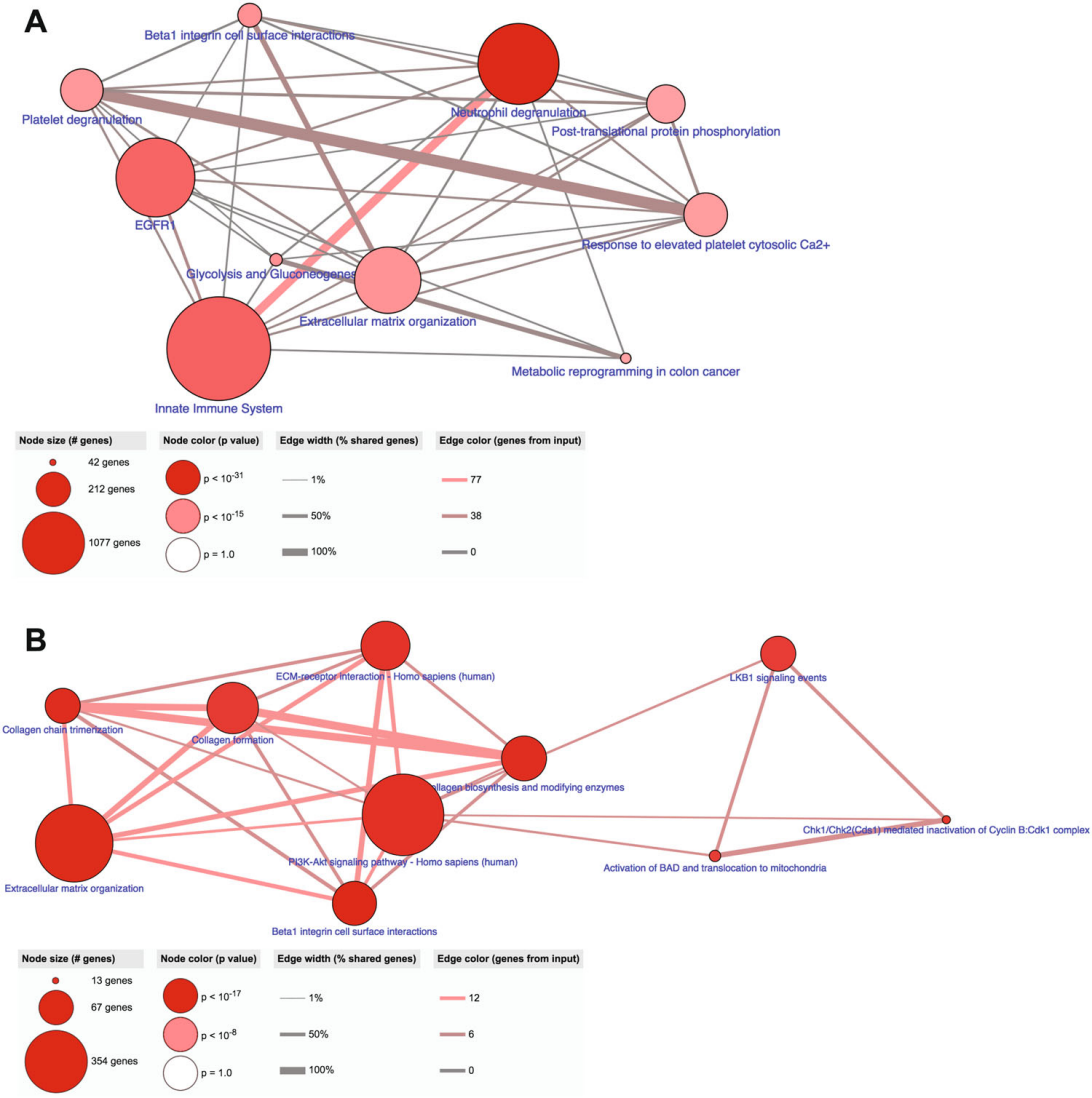
Neutrophil degranulation and 48 ‘core proteins’ are conserved in lymph node (LN) metastasis, across cancers. Concurrent comparison of the REPLICANT proteome to node-positive pancreatic and node-positive prostate adenocarcinoma identified 438 commonly expressed proteins. Pathway analysis of these 438 proteins is shown in **A**. ‘Neutrophil degranulation’ once again was identified as being important to LN metastasis. Certain protein families were also seen to recur across the three datasets (48 ‘core proteins’). Pathway analysis of these ‘core proteins’ is shown in **B**.

Across the three datasets, 438 common proteins were identified (Supplementary Table 6). Once again, the top 10 enriched pathways (Fig. 6A) were similar to the previous comparisons (Fig. 5A, B). Interestingly, all seven of the 14-3-3 family of proteins; two of the IQGAP family of proteins (IQGAP1 and IQGAP2); four of the 36 human SERPIN proteins (SERPINA1; SERPINA3; SERPINB1 and SERPINH1); four of the S100 proteins (S100A8; S100A9; S100A10 and S100A11); six of the 12 Annexin proteins (ANXA1-6); two of the ezrin/radixin/moesin (ERM) family of proteins (EZN and MSN); three of the 12 aldehyde dehydrogenase protein family (ALDH1A1; ALDH2 and ALDH9A1); and four of the 11 human cathepsins (CSTB, CSTD; CSTG and CSTZ) were conserved across the three cancers. In addition, a number of ECM proteins were conserved across the datasets: 10 collagen subunits; fibronectin, laminin B, periostin, tenascin and vitronectin. Pathway analysis of these 48 conserved proteins is shown in Fig. 6B.

Thus, ‘neutrophil degranulation’ is recurrently highlighted as the most significant immune pathway in LN metastasis, irrespective of where the primary tumour originates.

## Discussion

We have shown previously that human ALNs can be sustained ex-vivo for scientific investigation using normothermic perfusion^8^. Using shotgun proteomics, we now show that the protein repertoire of the circulating fluid collected during these experiments (‘perfusate proteome’) reflects ALN pathophysiology and thus, may be suitable for biomarker discovery.

As expected, MIF analysis of reactive and macrometastatic ALNs confirmed that the total number of B cells, CD4^+^ T cells, CD8^+^ T cells and T-regs decreases significantly as cancer colonises a node^6^. Interestingly, within macrometastastic nodes, mTIL distribution was significantly higher across the whole ALN section than in areas containing stroma. This result needs to be investigated further in a larger cohort of ALNs. Although TILs are an established biomarker in primary Her-2 positive and triple-negative BC, uncertainty exists at present as to precisely how to quantify mTILs in LNs largely for two reasons: first, not all metastases contain stroma; and second, LNs contain a large lymphoid population which confounds assessment^28^. Furthermore, how mTIL infiltration and PD-L1 expression relate to each other in LNs is unclear^43,44^. The ubiquitous co-expression of PD-L1 in our ALNs made cell density quantification difficult. However, by using average staining intensity for each cell type, we were able to show that approximately 30% of PD-L1 expression localised to T-lymphocytes. Unlike TIL distribution, PD-L1 expression appeared to be uniform in each of the compartments analysed. This may be related to the PD-L1 antibody that we used however^45,46^. Although this clone has been used previously to assess PD-L1 expression in primary and metastatic BC^47^, it is not used routinely for diagnosis. Only the SP142 clone has been approved for diagnostic use^48^, and only in triple-negative BC (not predominant in our cohort). Intriguingly, a positive PD-L1 result using this clone is defined by any staining intensity in immune cells in >1% of the tumour. It will be interesting to evaluate this clone in MIF sections of ALNs containing the various subtypes of BC in future experiments, especially in response to immune checkpoint inhibitor (ICI) therapies^8^.

Pathway analysis of the significantly upregulated proteins in reactive and/or metastatic nodes reflected the change in cell composition highlighted by MIF. Reactive nodes showed a maintenance of immune function, whereas macrometastatic nodes showed a loss in immune function (except for ‘neutrophil degranulation’), and a shift to ECM degradation and keratinisation. The latter is consistent with the presence of epithelial cells within the ALN. Alterations in the ECM are known to affect fluid flow; lymphangiogenesis; angiogenesis; cancer cell adhesion, migration and invasion; cytokine signalling; and immune modulation^49–51^, all of which contribute to metastasis and colonisation^6^. Unlike the lung, liver or bone however, changes in the ECM at a protein level in metastatic LNs is not currently well understood. Interestingly, these data could not be obtained by stratifying TCGA(CPTAC) primary BC proteome according to ALN status, which highlights the novelty and importance of perfusate sample collection/analysis in trying to understand metastasis.

Qualitative comparison of the perfusate proteome with the tissue proteomes of LN-positive pancreatic ductal and prostatic adenocarcinoma showed that 438 proteins were commonly expressed. This could be technical to some extent, since it has been shown that increasing the number of sample replicates in a proteomics experiment can correct for biological diversity^9,52,53^. Still, if one considers that sample type and collection methods differed between the studies, and that different proteomic technologies were used, this degree of overlap suggests that recurrent biological phenomena are being detected between cancers types. The fact that 48 ‘core proteins’ are conserved across these datasets is even more thought-provoking. When these were subjected to pathway analysis, the PI3K-Akt signalling pathway was the most significant one identified. This pathway is frequently dysregulated in BC, PDAC and prostate cancer, and is currently being targeted therapeutically in clinical trials^54–59^. Similarly, the role of various β1 integrins in carcinogenesis is well established, particularly in terms of their interactions with collagen and the ECM^60^. Interestingly, the integrins α4β1 and α9β1 are known to induce lymphangiogenesis and LN metastasis; for α4β1, this is mediated by PI3Kalpha^61–64^. Key questions for future experiments will be to ascertain if these ‘core proteins’ are reproducibly expressed in perfusate samples harvested from other node-positive adenocarcinomas; if they can delineate nodal tumour burden; and if combinations of these proteins can be used as biomarkers of nodal spread.

Finally, ‘neutrophil degranulation’ was highlighted recurrently by pathway analysis as being important to LN metastasis. The role of neutrophil degranulation in cancer metastasis is gaining interest, particularly in terms of how it affects the adaptive T cell response^65^. Like macrophages, neutrophils can either promote or suppress immunity via cell-to-cell contact, degranulation of intracellular contents, the release/production of neutrophil extracellular traps (NETs) and/or cytokine release^65^. Recent evidence suggests that clusters of circulating tumour cells and neutrophils accelerate haematogenous metastasis in BC^66^, and that neutrophils are required for IL11-induced and FIGF-induced polyclonal BC metastasis^67^. Precisely how neutrophils contribute to nodal metastasis in BC has yet to be elucidated. We did not see statistically significant differences in absolute neutrophil numbers between reactive and metastatic perfused nodes. It will be interesting to investigate the relationship between neutrophil tissue infiltration and neutrophil degranulation in future experiments, ideally in a larger cohort of samples taken from BC patients, as well as from patients with other epithelial malignancies.

We have shown for the first time that proteomic analysis of REPLICANT perfusate^8^ is feasible, and reflects colonisation-induced changes in the ALN microenvironment^6^. Our data also suggests that these findings could be relevant to other epithelial malignancies. Future work will include validating these findings in a larger series of perfused ALNs, including micrometastatic disease.

## Methods

### Patient cohort and ALN harvest

ALNs were harvested from 10 BC patients and perfused ex vivo at 37 ^o^C as described previously (King’s Health Partners (KHP) Cancer Biobank Research Ethics Committee No: 18/EE/0025)^8^. Informed consent was obtained from all patients prior to surgery. Four of the perfused ALNs contained macrometastases; one contained a micrometastasis; and five were reactive. A matched autologous ‘baseline control’ ALN, which mirrored the perfused ALN in terms of disease state, was harvested at time-point 0 from each patient. The clinico-pathological characteristics of the cohort, including representative H&E sections, have been described previously^8^. Of note, none of the perfused ALNs showed any histological evidence of necrosis^8^.

### Perfusate collection

Perfusate samples (*n* = 10) were collected from the perfusion circuit via three-way taps at the end-point of each experiment. Samples were stored at −80 ^o^C prior to proteomic analysis.

### Multiplex Immunofluorescence (MIF)

4μm FFPE sections from each perfused (*n* = 10) and baseline control ALN (*n* = 10) were sequentially stained using an Opal 7-colour reagent kit (Akoya Bioscience) according to the manufacturer’s instructions. The following antibodies were used: CD4 (Abcam 133616; Opal 520), CD8 (Dako, M710301; Opal 570), CD20 (Dako, M075529; Opal 540), PD-L1 (Cell Signalling, 13684; Opal 620), FoxP3 (Abcam, 20034; Opal 650), Pan-cytokeratin (Dako, M351501; Opal 690; on metastatic ALNs only), and CD68 (Dako, M087629; Opal 690 on reactive ALNs only). Control tissue samples were stained for each marker in parallel. Slides were imaged using the Vectra 3.0 pathology imaging system (Akoya Bioscience). Cell phenotyping and density (total number of cells/mm^2^) was quantified over the entire tissue section (i.e. 50–600 fields per sample depending on the size of the ALN), using a custom algorithm developed in the inForm software package. Briefly, the algorithm was initially trained by machine learning on manually annotated examples. Samples were then batch processed to segment the tissue by tissue type, then to identify/phenotype cells, and finally to quantify cell numbers or signal intensity^68^.

### Proteomic Analysis

Perfusate samples were heated to 95 °C, reduced with Dithiothreitol (50 mM) and alkylated by Iodoacetamide (100 mM). Following probe sonication, samples underwent Filter Aided Sample Preparation (FASP) in the Amicon Ultra-4 (10KDa cut-off, Millipore)^69^. Triethylammonium bicarbonate (TEAB; 100 mM) was used in the buffer exchange. Peptides were recovered from the filter after an 18-h trypsin digestion (Pierce, MS grade) at 37 °C with additional two washes of TEAB (100 mM then 1 M).

Following quantification (Nanodrop), 20 µg of each sample was TMT labelled. The mixture was fractionated on a BEH XBridge C18 column (2.1 mm i.d. × 150 mm) with a 35-min gradient from 5–35% CH_3_CN/NH_4_OH then concatenated to 8 fractions for LC-MS/MS analysis on an Orbitrap Fusion Lumos coupled with an Ultimate 3000 RSLCnano System. Samples were loaded on a nanotrap (100 µm id × 2 cm) (PepMap C18, 5 µ) then separated on an analytical column (75 µm id × 50 cm) (PepMap C18, 2 µ) over a 90-min gradient of 4–30.4% CH_3_CN/0.1% formic acid/120 min cycle time per fraction. The Orbitrap Fusion Lumos was operated in the Top Speed mode at 3 s per cycle and data was acquired via the MS3-SPS5 method. Raw files were processed in Proteome Discoverer 2.2 (Thermo Fisher) using the Sequest HT search engine. Spectra were searched against a reviewed Uniprot Homo sapiens database (March 2019). Peptides were validated by Percolator with q-value set at 0.01 (strict) and 0.05 (relaxed). The TMT reporter ion quantification used unique peptides only. The co-isolation threshold was set at 100%. Peptides with an average reported signal: noise >3 were used for protein quantification. Only master proteins were reported. Protein abundance was normalised by equalising the total abundance between different runs/channels, and then scaled to an average of 100 across all samples^70^.

### Neutrophil quantification

The average number of neutrophils per high power field (HPF; 20 fields in total) was quantified in representative H&E sections (4 µm) from each anonymised ALN sample by a histopathologist (KN).

### The Cancer Genome Atlas (TCGA) BC proteomics analysis

TCGA BC proteomics dataset (Clinical Proteomic Tumour Analysis Consortium (CPTAC) study)^37–39^ was downloaded from cBioportal (February 2019)^71^. The 74 patient primary BC samples in this dataset were stratified into three groups according to the provided clinical data: those who had undergone only a sentinel lymph node biopsy (‘SLNB’; *n* = 13), and therefore had no/low burden of axillary disease; those who had gone on after a SLNB to have a completion clearance (‘CC’; *n* = 22), and therefore had a higher axillary tumour burden than the SLNB group; and those who had an ALND upfront (‘ALND’, *n* = 22), and therefore had a high axillary tumour burden at diagnosis. Statistical comparison of protein abundance between these three groups was performed using one-way ANOVA. Tukey HSD test was applied for post-hoc analysis. Visualisations and analysis were performed in R statistical programming environment v3.5.0.

### Statistical analysis

To assess differences in the immune composition of ALNs, a Wilcoxon test was used to compare control to perfused nodes; a Kruskal-Wallis test was used to assess regulatory T cell (T-regs) and lymphocyte distribution within metastatic nodes, and to compare T-reg numbers between reactive and metastatic nodes; and a two-tailed Mann–Whitney was used to quantify differences between reactive and metastatic nodes. For the proteomics analysis, the data was initially filtered to include only those proteins with a scaled normalised protein abundance ≥2. A Student’s *t*-test was then performed to determine differential expression between reactive and metastatic perfusate samples. A *p*-value ≤ 0.05 was considered significant. Pathway enrichment analysis was performed with ConsensusPathDB^31^.

### Reporting summary

Further information on experimental design is available in the Nature Research Reporting Summary linked to this paper.

## Supplementary information

Supplementary Data

Reporting Summary Checklist

## Data Availability

The mass-spectrometry based proteomics data generated during the study, are publicly available in the PRIDE repository: https://identifiers.org/pride.project:PXD022722^70^. The multiplex immunofluorescence data generated during this study, are available in the figshare repository: 10.6084/m9.figshare.13522442^68^. The TCGA data analysed during the study, are available in the cBioPortal for Cancer Genomics: https://identifiers.org/cbioportal:brca_tcga^71^. All other data supporting the findings of this study, are available as part of the supplementary files that accompany the article.

## References

[1] Naidoo K, Pinder SE (2017). Micro- and macro-metastasis in the axillary lymph node: a review. Surgeon.

[2] de Boer M (2009). Micrometastases or isolated tumor cells and the outcome of breast cancer. N. Engl. J. Med..

[3] de Boer M, van Dijck JA, Bult P, Borm GF, Tjan-Heijnen VC (2010). Breast cancer prognosis and occult lymph node metastases, isolated tumor cells, and micrometastases. J. Natl Cancer Inst..

[4] Giuliano AE (2010). Locoregional recurrence after sentinel lymph node dissection with or without axillary dissection in patients with sentinel lymph node metastases: the American College of Surgeons Oncology Group Z0011 randomized trial. Ann. Surg..

[5] Ahmed M, Douek M (2013). Life beyond Z11. Breast.

[6] Fidler IJ (2003). The pathogenesis of cancer metastasis: the ‘seed and soil’ hypothesis revisited. Nat. Rev. Cancer.

[7] NICE. *Early and Locally Advanced Breast Cancer: Diagnosis And Management*. https://www.nice.org.uk/guidance/ng101 (2018).

[8] Barrow-McGee, R. et al. Real-time ex vivo perfusion of human lymph nodes invaded by cancer (REPLICANT): a feasibility study. *J. Pathol.*10.1002/path.5367 (2019). 10.1002/path.5367 PMC7065097 31755096

[9] Orton DJ, Doucette AA (2013). Proteomic workflows for biomarker identification using mass spectrometry-technical and statistical considerations during initial discovery. Proteomes.

[10] Barry P (2018). The spatiotemporal evolution of lymph node spread in early breast cancer. Clin. Cancer Res..

[11] Merker JD (2018). Circulating tumor DNA analysis in patients with cancer: American society of clinical oncology and college of american pathologists joint review. J. Clin. Oncol..

[12] Li J, Zhang Z, Rosenzweig J, Wang YY, Chan DW (2002). Proteomics and bioinformatics approaches for identification of serum biomarkers to detect breast cancer. Clin. Chem..

[13] Nakagawa T (2006). Proteomic profiling of primary breast cancer predicts axillary lymph node metastasis. Cancer Res..

[14] Pietrowska M (2009). Mass spectrometry-based serum proteome pattern analysis in molecular diagnostics of early stage breast cancer. J. Transl. Med..

[15] Wang L (2011). Primary study of lymph node metastasis-related serum biomarkers in breast cancer. Anat. Rec..

[16] Lobo MD (2017). Label-free proteome analysis of plasma from patients with breast cancer: stage-specific protein expression. Front. Oncol..

[17] Chen L (2018). Label-free quantitative proteomic screening of candidate plasma biomarkers for the prognosis of breast cancer with different lymph node statuses. Proteom. Clin. Appl..

[18] Thongwatchara P (2011). Differential protein expression in primary breast cancer and matched axillary node metastasis. Oncol. Rep..

[19] Li J (2008). Omics-based profiling of carcinoma of the breast and matched regional lymph node metastasis. Proteomics.

[20] Milioli HH (2015). Comparative proteomics of primary breast carcinomas and lymph node metastases outlining markers of tumor invasion. Cancer Genomics Proteom..

[21] Zeng L (2017). Identification of nucleobindin-2 as a potential biomarker for breast cancer metastasis using iTRAQ-based quantitative proteomic analysis. J. Cancer.

[22] Pozniak Y (2016). System-wide clinical proteomics of breast cancer reveals global remodeling of tissue homeostasis. Cell Syst..

[23] Zeng L (2019). Prognostic value of biomarkers EpCAM and alphaB-crystallin associated with lymphatic metastasis in breast cancer by iTRAQ analysis. BMC Cancer.

[24] Broggi MAS (2019). Tumor-associated factors are enriched in lymphatic exudate compared to plasma in metastatic melanoma patients. J. Exp. Med..

[25] Clement CC (2013). Protein expression profiles of human lymph and plasma mapped by 2D-DIGE and 1D SDS-PAGE coupled with nanoLC-ESI-MS/MS bottom-up proteomics. J. Proteom..

[26] Dzieciatkowska M (2014). Lymph is not a plasma ultrafiltrate: a proteomic analysis of injured patients. Shock.

[27] Leak LV (2004). Proteomic analysis of lymph. Proteomics.

[28] Hendry S (2017). Assessing tumor-infiltrating lymphocytes in solid tumors: a practical review for pathologists and proposal for a standardized method from the international immunooncology biomarkers working group: part 1: assessing the host immune response, TILs in invasive breast carcinoma and ductal carcinoma in situ, metastatic tumor deposits and areas for further research. Adv. Anat. Pathol..

[29] Togashi Y, Shitara K, Nishikawa H (2019). Regulatory T cells in cancer immunosuppression–implications for anticancer therapy. Nat. Rev. Clin. Oncol..

[30] Sun C, Mezzadra R, Schumacher TN (2018). Regulation and function of the PD-L1 checkpoint. Immunity.

[31] Kamburov A (2011). ConsensusPathDB: toward a more complete picture of cell biology. Nucleic Acids Res..

[32] Wiig H, Swartz MA (2012). Interstitial fluid and lymph formation and transport: physiological regulation and roles in inflammation and cancer. Physiol. Rev..

[33] Johansson A, Hamzah J, Ganss R (2016). More than a scaffold: stromal modulation of tumor immunity. Biochim. Biophys. Acta.

[34] Gretz JE, Kaldjian EP, Anderson AO, Shaw S (1996). Sophisticated strategies for information encounter in the lymph node: the reticular network as a conduit of soluble information and a highway for cell traffic. J. Immunol..

[35] Fletcher AL, Acton SE, Knoblich K (2015). Lymph node fibroblastic reticular cells in health and disease. Nat. Rev. Immunol..

[36] Martinez VG (2019). Fibroblastic reticular cells control conduit matrix deposition during lymph node expansion. Cell Rep..

[37] Edwards NJ (2015). The CPTAC data portal: a resource for cancer proteomics research. J. Proteome Res..

[38] Ellis MJ (2013). Connecting genomic alterations to cancer biology with proteomics: the NCI Clinical Proteomic Tumor Analysis Consortium. Cancer Discov..

[39] Mertins P (2016). Proteogenomics connects somatic mutations to signalling in breast cancer. Nature.

[40] Brierley J. D., G. M. a. W. C. *TNM Classification of Malignant Tumours*, 8th edn. (Wiley-Black, 2016).

[41] Naidoo K (2012). Proteome of formalin-fixed paraffin-embedded pancreatic ductal adenocarcinoma and lymph node metastases. J. Pathol..

[42] Muller AK (2018). Proteomic characterization of prostate cancer to distinguish nonmetastasizing and metastasizing primary tumors and lymph node metastases. Neoplasia.

[43] Buisseret L (2017). Tumor-infiltrating lymphocyte composition, organization and PD-1/ PD-L1 expression are linked in breast cancer. Oncoimmunology.

[44] Noske A (2019). Relevance of tumour-infiltrating lymphocytes, PD-1 and PD-L1 in patients with high-risk, nodal-metastasised breast cancer of the German Adjuvant Intergroup Node-positive study. Eur. J. Cancer.

[45] Yeong, J. et al. Multiplex immunohistochemistry/immunofluorescence (mIHC/IF) for PD-L1 testing in triple-negative breast cancer: a translational assay compared with conventional IHC. *J. Clin. Pathol.*10.1136/jclinpath-2019-206252 (2020). 10.1136/jclinpath-2019-206252 31969377

[46] Bensch F (2018). (89)Zr-atezolizumab imaging as a non-invasive approach to assess clinical response to PD-L1 blockade in cancer. Nat. Med..

[47] Szekely B (2018). Immunological differences between primary and metastatic breast cancer. Ann. Oncol..

[48] Schmid P (2018). Atezolizumab and Nab-paclitaxel in advanced triple-negative breast cancer. N. Engl. J. Med..

[49] Lu P, Weaver VM, Werb Z (2012). The extracellular matrix: a dynamic niche in cancer progression. J. Cell Biol..

[50] Psaila B, Lyden D (2009). The metastatic niche: adapting the foreign soil. Nat. Rev. Cancer.

[51] Paolillo, M. & Schinelli, S. Extracellular matrix alterations in metastatic processes. *Int. J. Mol. Sci.* **20**, 10.3390/ijms20194947 (2019). 10.3390/ijms20194947 PMC6802000 31591367

[52] Ghaemmaghami S (2003). Global analysis of protein expression in yeast. Nature.

[53] Liu H, Sadygov RG, Yates JR (2004). A model for random sampling and estimation of relative protein abundance in shotgun proteomics. Anal. Chem..

[54] Cancer Genome Atlas, N. (2012). Comprehensive molecular portraits of human breast tumours. Nature.

[55] Armenia J (2018). The long tail of oncogenic drivers in prostate cancer. Nat. Genet..

[56] Conway JR, Herrmann D, Evans TJ, Morton JP, Timpson P (2019). Combating pancreatic cancer with PI3K pathway inhibitors in the era of personalised medicine. Gut.

[57] Liu P, Cheng H, Roberts TM, Zhao JJ (2009). Targeting the phosphoinositide 3-kinase pathway in cancer. Nat. Rev. Drug Discov..

[58] Sarker D, Reid AH, Yap TA, de Bono JS (2009). Targeting the PI3K/AKT pathway for the treatment of prostate cancer. Clin. Cancer Res..

[59] Turner NC, Neven P, Loibl S, Andre F (2017). Advances in the treatment of advanced oestrogen-receptor-positive breast cancer. Lancet.

[60] Raab-Westphal, S., Marshall, J. F. & Goodman, S. L. Integrins as therapeutic targets: successes and cancers. *Cancers* **9**, 10.3390/cancers9090110 (2017). 10.3390/cancers9090110 PMC5615325 28832494

[61] Garmy-Susini B (2013). PI3Kalpha activates integrin alpha4beta1 to establish a metastatic niche in lymph nodes. Proc. Natl Acad. Sci. USA.

[62] Garmy-Susini B (2010). Integrin alpha4beta1 signaling is required for lymphangiogenesis and tumor metastasis. Cancer Res..

[63] Vlahakis NE, Young BA, Atakilit A, Sheppard D (2005). The lymphangiogenic vascular endothelial growth factors VEGF-C and -D are ligands for the integrin alpha9beta1. J. Biol. Chem..

[64] Hoye AM, Couchman JR, Wewer UM, Fukami K, Yoneda A (2012). The newcomer in the integrin family: integrin alpha9 in biology and cancer. Adv. Biol. Regul..

[65] Minns, D., Smith, K. J. & Findlay, E. G. Orchestration of adaptive T cell responses by neutrophil granule contents. *Mediat. Inflamm.* **2019**, 8968943, (2019). 10.1155/2019/8968943 PMC6431490 30983883

[66] Szczerba BM (2019). Neutrophils escort circulating tumour cells to enable cell cycle progression. Nature.

[67] Janiszewska M (2019). Subclonal cooperation drives metastasis by modulating local and systemic immune microenvironments. Nat. Cell Biol..

[68] Stevenson, J. et al. Multiplex immunofluorescence data and metadata supporting the article: proteomics of REPLICANT perfusate detects changes in the metastatic lymph node microenvironment. *figshare*10.6084/m9.figshare.13522442 (2021). 10.1038/s41523-021-00227-7 PMC7935848 33674617

[69] Wisniewski JR, Zougman A, Nagaraj N, Mann M (2009). Universal sample preparation method for proteome analysis. Nat. Methods.

[70] Stevenson, J. et al. Proteomics of REPLICANT perfusate detects changes in the metastatic lymph node microenvironment. *PRIDE Archive* https://identifiers.org/pride.project:PXD022722 (2021). 10.1038/s41523-021-00227-7 PMC7935848 33674617

[71] TCGA Breast Invasive Carcinoma*. cBioPortal for Cancer Genomics*, https://identifiers.org/cbioportal:brca_tcga (2016).

