# Supplementary material for: Proteomics of REPLICANT perfusate detects changes in the metastatic lymph node microenvironment
Source: NPJ Breast Cancer. 2021 Mar 5;7:24. doi: 10.1038/s41523-021-00227-7 (PMC7935848; doi:10.1038/s41523-021-00227-7)
Supplement: Supplementary file 1 — Supplementary Data [file 41523_2021_227_MOESM1_ESM.pdf]

Supplementary Figure Legends

Supplementary Figure 1 Representative images of PD-L1 staining in macrometastatic axillary lymph nodes (ALNs). Representative images, taken from three different macrometastatic ALNs are shown to highlight PD-L1 (cyan; red arrows) signal intensity.

Supplementary Figure 2 Comparison of the perfusate proteome to TCGA(CPTAC) primary breast cancer (BC) proteome. We stratified the TCGA(CPTAC) primary BC proteomics data into three groups according to axillary tumour burden (ATB): those who had a sentinel lymph node biopsy only (SLNB; no/low ATB); those who had a completion clearance (CC; higher burden of ATB than the SLNB group); and those who had an axillary lymph node dissection, and high ATB, at diagnosis (ALND). Of the 10 most abundant perfusate proteins, eight reactive and eight metastatic were identified in the TCGA(CPTAC) data. Five reactive proteins (**A**) were not significantly differentially expressed between the three groups. None of the metastatic proteins (**B**) were significantly differentially expressed across the three groups, although CDH1 showed a trend toward being up-regulated in patients with a high ATB ($p = 0.081$). (Graphs show median with interquartile range)

Supplementary Table 1 The perfusate proteome. Nine perfusate samples (20µg protein per sample) from nine axillary lymph nodes (ALNs) underwent Tandem Mass Tag (TMT) labelled mass spectrometry. Samples were pre-fractionated prior to quantification. The TMT reporter ion quantification used unique peptides only.

Peptides were validated by Percolator with q-value set at 0.01 (strict) and 0.05 (relaxed). Only master proteins were reported. Protein abundance was normalised by equalising the total abundance between different runs/channels, and then scaled to an average of 100 across all samples. In total, 1453 proteins were identified.

Supplementary Table 2 Tukey's honestly significant difference (HSD) post-hoc analysis of the 10 most abundant proteins in reactive and metastatic perfusate proteins in the TCGA(CPTAC) data.

Supplementary Table 3 Comparison of the perfusate proteome with the proteome of breast cancer lymph node metastasis[19] reveals 86 commonly expressed proteins.

Supplementary Table 4 Comparison of the perfusate proteome with the proteome of pancreatic ductal adenocarcinoma lymph node metastases[41] reveals 515 commonly expressed proteins.

Supplementary Table 5 Comparison of the perfusate proteome with the proteome of prostatatic adenocarcinoma lymph node metastases[42] reveals 854 commonly expressed proteins.

Supplementary Table 6 438 commonly expressed proteins between the perfusate proteome, the proteome of pancreatic ductal adenocarcinoma lymph node metastasis and the proteome of prostate adenocarcinoma lymph node metastases[41,42].

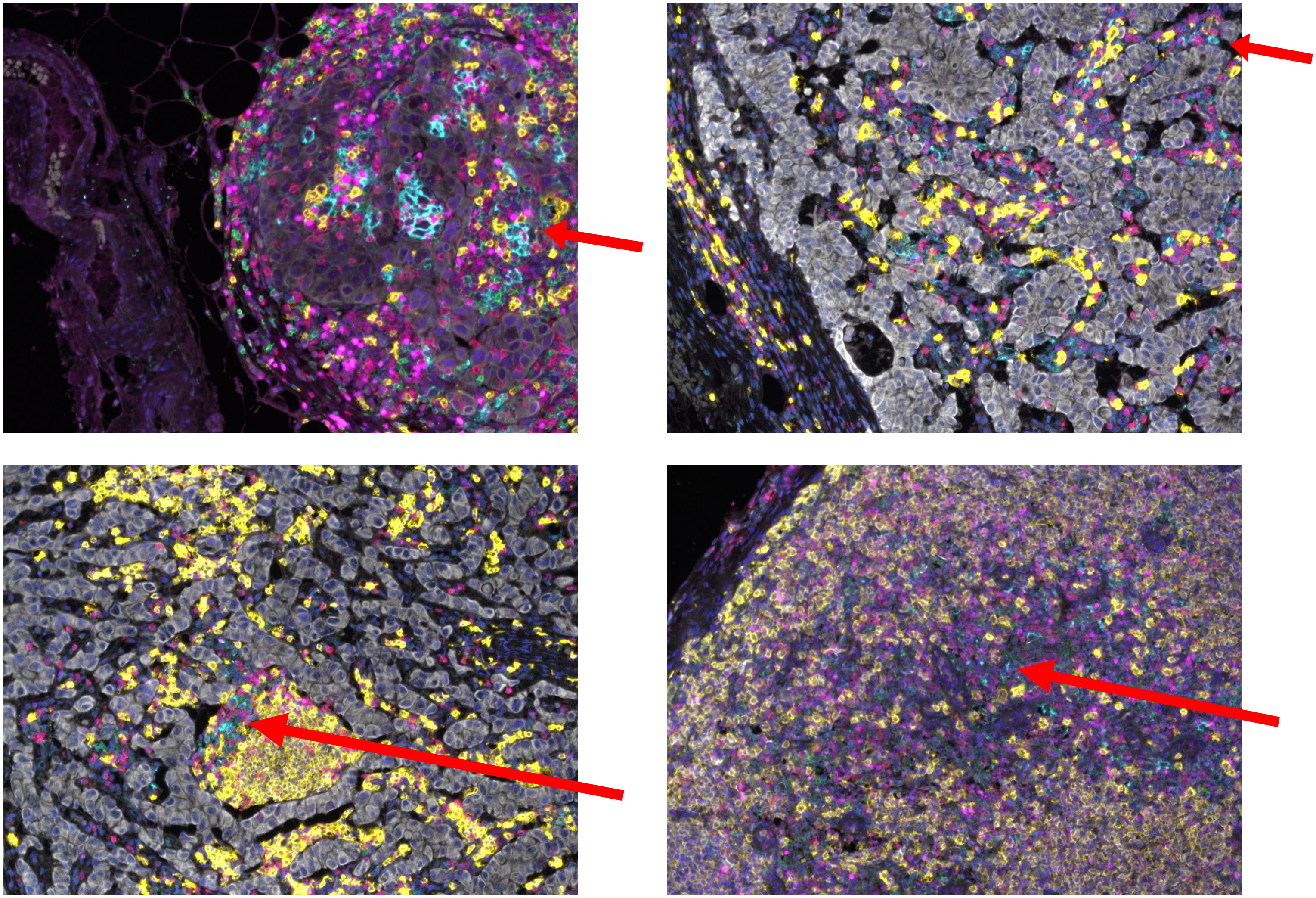

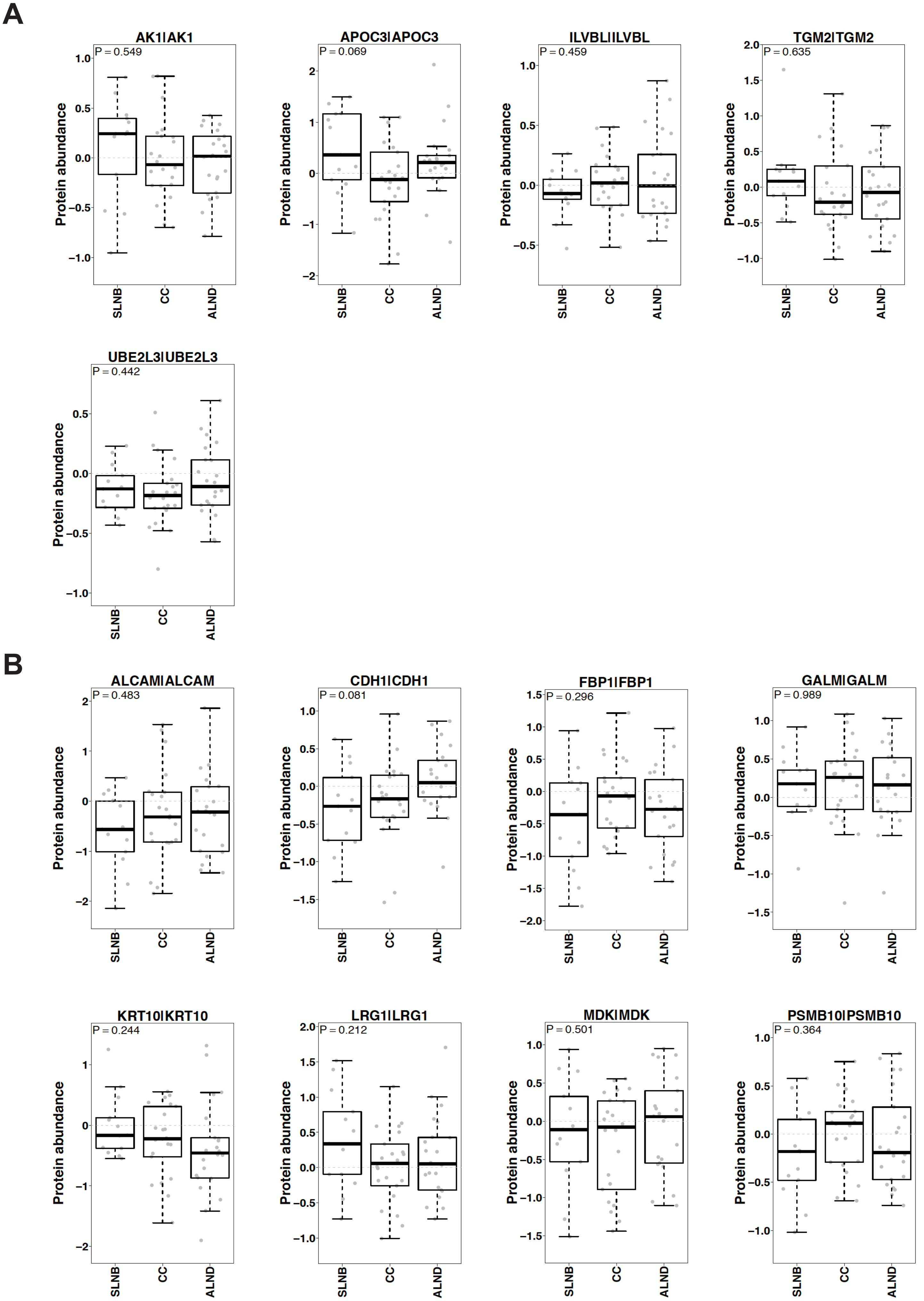
A
AK1|AK1
P = 0.549
APOC3|APOC3
P = 0.069
ILVBL|ILVBL
P = 0.459
TGM2|TGM2
P = 0.635
UBE2L3|UBE2L3
P = 0.442
B
ALCAM|ALCAM
P = 0.483
CDH1|CDH1
P = 0.081
FBP1|FBP1
P = 0.296
GALM|GALM
P = 0.989
KRT10|KRT10
P = 0.244
LRG1|LRG1
P = 0.212
MDK|MDK
P = 0.501
PSMB10|PSMB10
P = 0.364
Protein abundance
SLNB
CC
ALND

Accession	Description	# Unique Peptides	T-test
Q9C0C9	(E3-independent) E2 ubiquitin-conjugating enzyme	6	0.1065964
Q04446	1,4-alpha-glucan-branching enzyme	3	0.05115141
P61604	10 kDa heat shock protein, mitochondrial	6	0.30640883
Q9NRX4	14 kDa phosphohistidine phosphatase	5	0.19804284
P31946	14-3-3 protein beta/alpha	6	0.3574181
P62258	14-3-3 protein epsilon	17	0.2403162
Q04917	14-3-3 protein eta	6	0.4208543
P61981	14-3-3 protein gamma	5	0.23513004
P31947	14-3-3 protein sigma	4	0.18850026
P27348	14-3-3 protein theta	9	0.35773443
P63104	14-3-3 protein zeta/delta	12	0.36220077
Q9C0C2	182 kDa tankyrase-1-binding protein	18	0.66684804
P52758	2-iminobutanoate/2-iminopropanoate deaminase	3	0.16206736
Q16698	2,4-dienoyl-CoA reductase, mitochondrial	3	0.10312546
O43598	2'-deoxynucleoside 5'-phosphate N-hydrolase 1	2	0.10257786
P09543	2',3'-cyclic-nucleotide 3'-phosphodiesterase	3	0.30267683
Q99460	26S proteasome non-ATPase regulatory subunit 1	3	0.12092972
O75832	26S proteasome non-ATPase regulatory subunit 10	2	0.25918693
O00231	26S proteasome non-ATPase regulatory subunit 11	3	0.14857295
O00232	26S proteasome non-ATPase regulatory subunit 12	2	0.60767714
Q13200	26S proteasome non-ATPase regulatory subunit 2	6	0.04843486
O43242	26S proteasome non-ATPase regulatory subunit 3	2	0.05787452
P55036	26S proteasome non-ATPase regulatory subunit 4	7	0.46224495
Q15008	26S proteasome non-ATPase regulatory subunit 6	3	0.12263943
O00233	26S proteasome non-ATPase regulatory subunit 9	5	0.02904751
P62333	26S proteasome regulatory subunit 10B	5	0.23376848
P62191	26S proteasome regulatory subunit 4	4	0.06977163
P17980	26S proteasome regulatory subunit 6A	6	0.25011598
P43686	26S proteasome regulatory subunit 6B	4	0.46504295
P35998	26S proteasome regulatory subunit 7	4	0.01657462
P62195	26S proteasome regulatory subunit 8	4	0.30156212

Q13442	28 kDa heat- and acid-stable phosphoprotein	4	0.43332729
P31937	3-hydroxyisobutyrate dehydrogenase, mitochondrial	4	0.18020813
Q6NVY1	3-hydroxyisobutyryl-CoA hydrolase, mitochondrial	2	0.92751786
P42765	3-ketoacyl-CoA thiolase, mitochondrial	9	0.96841951
P25325	3-mercaptopyruvate sulfurtransferase	6	0.12613638
P49189	4-trimethylaminobutyraldehyde dehydrogenase	13	0.1299624
P62280	40S ribosomal protein S11	3	0.97483873
P25398	40S ribosomal protein S12	2	0.58164235
P15880	40S ribosomal protein S2	3	0.77175589
P62857	40S ribosomal protein S28	2	0.68615907
P23396	40S ribosomal protein S3	7	0.86275609
P61247	40S ribosomal protein S3a	3	0.24413708
P62701	40S ribosomal protein S4, X isoform	3	0.72753234
P46782	40S ribosomal protein S5	2	0.35671574
P62241	40S ribosomal protein S8	3	0.20887795
P46781	40S ribosomal protein S9	2	0.8263839
P08865	40S ribosomal protein SA	4	0.33186883
Q8IUZ5	5-phosphohydroxy-L-lysine phospho-lyase	3	0.14422822
Q5TFE4	5'-nucleotidase domain-containing protein 1	2	0.22757193
Q00013	55 kDa erythrocyte membrane protein	8	0.26198571
P52209	6-phosphogluconate dehydrogenase, decarboxylating	11	0.16425663
O95336	6-phosphogluconolactonase	6	0.13516519
P10809	60 kDa heat shock protein, mitochondrial	15	0.09379098
P10155	60 kDa SS-A/Ro ribonucleoprotein	11	0.03988387
P05388	60S acidic ribosomal protein P0	5	0.25094879
P05387	60S acidic ribosomal protein P2	2	0.68106591
P27635	60S ribosomal protein L10	2	0.43701988
P62906	60S ribosomal protein L10a	5	0.01662707
P18621	60S ribosomal protein L17	2	0.78996068
Q07020	60S ribosomal protein L18	2	0.67559314
Q02543	60S ribosomal protein L18a	2	0.09626898
P36578	60S ribosomal protein L4	3	0.96906276

P18124	60S ribosomal protein L7	2	0.82320433
P08253	72 kDa type IV collagenase	3	0.46145853
Q02952	A-kinase anchor protein 12	65	0.75078866
Q9Y2D5	A-kinase anchor protein 2	11	0.61137152
Q99996	A-kinase anchor protein 9	2	0.74932212
A1L0T0	Acetolactate synthase-like protein	2	0.01408958
Q9BWD1	Acetyl-CoA acetyltransferase, cytosolic	4	0.48941237
P24752	Acetyl-CoA acetyltransferase, mitochondrial	4	0.06459692
O00763	Acetyl-CoA carboxylase 2	2	0.22028554
Q13510	Acid ceramidase	7	0.98062901
P39687	Acidic leucine-rich nuclear phosphoprotein 32 family member A	2	0.07759676
Q9BTT0	Acidic leucine-rich nuclear phosphoprotein 32 family member E	2	0.28865985
Q99798	Aconitate hydratase, mitochondrial	10	0.73154874
P61160	Actin-related protein 2	3	0.11352127
O15143	Actin-related protein 2/3 complex subunit 1B	2	0.15321274
O15144	Actin-related protein 2/3 complex subunit 2	4	0.216943
O15145	Actin-related protein 2/3 complex subunit 3	2	0.24867046
P59998	Actin-related protein 2/3 complex subunit 4	2	0.27186107
O15511	Actin-related protein 2/3 complex subunit 5	2	0.18573684
P61158	Actin-related protein 3	9	0.16351497
P68032	Actin, alpha cardiac muscle 1	7	0.18823307
P60709	Actin, cytoplasmic 1	7	0.16772085
P53999	Activated RNA polymerase II transcriptional coactivator p15	3	0.63441422
Q9H6R3	Acyl-CoA synthetase short-chain family member 3, mitochondrial	2	0.32419529
P07108	Acyl-CoA-binding protein	4	0.33937102
P49753	Acyl-coenzyme A thioesterase 2, mitochondrial	5	0.0829892
P13798	Acylamino-acid-releasing enzyme	18	0.89138333
Q6P587	Acylpyruvase FAHD1, mitochondrial	2	0.10900661
P46108	Adapter molecule crk	10	0.90572726
Q9NZK5	Adenosine deaminase 2	5	0.03349158
P55263	Adenosine kinase	6	0.30412858
P23526	Adenosylhomocysteinase	10	0.91514134

P54819	Adenylate kinase 2, mitochondrial	2	0.58342588
P00568	Adenylate kinase isoenzyme 1	8	0.04232559
P30566	Adenylosuccinate lyase	4	0.31559922
Q01518	Adenylyl cyclase-associated protein 1	12	0.22605612
P40123	Adenylyl cyclase-associated protein 2	3	0.21377095
Q8IUX7	Adipocyte enhancer-binding protein 1	8	0.9189748
Q9HDC9	Adipocyte plasma membrane-associated protein	2	0.16508829
Q15847	Adipogenesis regulatory factor	3	0.21494603
Q15848	Adiponectin	2	0.04205883
Q9NX46	ADP-ribose glycohydrolase ARH3	3	0.09666105
P61204	ADP-ribosylation factor 3	2	0.20053698
Q9UKK9	ADP-sugar pyrophosphatase	4	0.69928629
P43652	Afamin	24	0.82816237
O43488	Aflatoxin B1 aldehyde reductase member 2	2	0.958919
P16112	Aggrecan core protein	2	0.33919056
P24298	Alanine aminotransferase 1	4	0.06111376
P49588	Alanine--tRNA ligase, cytoplasmic	2	0.22037609
P00325	Alcohol dehydrogenase 1B	20	0.35085344
P40394	Alcohol dehydrogenase class 4 mu/sigma chain	2	0.05193545
P11766	Alcohol dehydrogenase class-3	10	0.48488314
Q8IZ83	Aldehyde dehydrogenase family 16 member A1	3	0.08440444
P05091	Aldehyde dehydrogenase, mitochondrial	11	0.0205448
P14550	Aldo-keto reductase family 1 member A1	9	0.61452868
P52895	Aldo-keto reductase family 1 member C2	4	0.21713434
P42330	Aldo-keto reductase family 1 member C3	2	0.61512985
Q96C23	Aldose 1-epimerase	7	0.02280846
P15121	Aldose reductase	8	0.97119591
Q6NUM9	All-trans-retinol 13,14-reductase	3	0.06174098
P55008	Allograft inflammatory factor 1	2	0.20824296
P02763	Alpha-1-acid glycoprotein 1	9	0.06034569
P19652	Alpha-1-acid glycoprotein 2	6	0.14187205
P01011	Alpha-1-antichymotrypsin	13	0.51865719

P01009	Alpha-1-antitrypsin	32	0.54680395
P04217	Alpha-1B-glycoprotein	13	0.30901294
P08697	Alpha-2-antiplasmin	6	0.05327441
P02765	Alpha-2-HS-glycoprotein	11	0.11282628
P01023	Alpha-2-macroglobulin	53	0.08209995
P12814	Alpha-actinin-1	20	0.25309366
P35609	Alpha-actinin-2	4	0.2687996
O43707	Alpha-actinin-4	22	0.23610191
P35611	Alpha-adducin	11	0.04178851
P61163	Alpha-centractin	3	0.10280308
P02511	Alpha-crystallin B chain	6	0.12628683
P06733	Alpha-enolase	19	0.10768298
P17050	Alpha-N-acetylgalactosaminidase	2	0.61837452
Q9NVD7	Alpha-parvin	6	0.05361745
P54920	Alpha-soluble NSF attachment protein	3	0.09690567
P37840	Alpha-synuclein	8	0.27935019
P21397	Amine oxidase [flavin-containing] A	2	0.06740731
Q03154	Aminoacylase-1	5	0.04539492
Q9H4A4	Aminopeptidase B	4	0.42651923
P15144	Aminopeptidase N	9	0.13505599
Q02094	Ammonium transporter Rh type A	2	0.53988459
Q7Z5R6	Amyloid beta A4 precursor protein-binding family B member 1-interacting protein	3	0.15399572
P05067	Amyloid-beta precursor protein	4	0.74137399
P03950	Angiogenin	2	0.19243298
P01019	Angiotensinogen	7	0.81924542
P16157	Ankyrin-1	48	0.150962
Q01484	Ankyrin-2	3	0.78906256
P04083	Annexin A1	15	0.03909617
P50995	Annexin A11	5	0.05954265
P07355	Annexin A2	22	0.30410731
P12429	Annexin A3	2	0.82628562

P09525	Annexin A4	13	0.72041288
P08758	Annexin A5	13	0.33223887
P08133	Annexin A6	23	0.09606301
P20073	Annexin A7	4	0.18163087
P03973	Antileukoproteinase	2	0.23471566
P01008	Antithrombin-III	24	0.05666191
Q10567	AP-1 complex subunit beta-1	5	0.1203101
O94973	AP-2 complex subunit alpha-2	5	0.28864953
P63010	AP-2 complex subunit beta	2	0.06410056
O00203	AP-3 complex subunit beta-1	3	0.08487293
P02647	Apolipoprotein A-I	31	0.59102672
P02652	Apolipoprotein A-II	5	0.14823539
P06727	Apolipoprotein A-IV	26	0.8938635
P04114	Apolipoprotein B-100	117	0.07069785
P02654	Apolipoprotein C-I	4	0.16758006
P02655	Apolipoprotein C-II	5	0.0268315
P02656	Apolipoprotein C-III	2	0.02726916
P05090	Apolipoprotein D	6	0.87797505
P02649	Apolipoprotein E	12	0.45973108
Q13790	Apolipoprotein F	2	0.59147909
O14791	Apolipoprotein L1	8	0.17125918
O95445	Apolipoprotein M	2	0.05702386
P08519	Apolipoprotein(a)	3	0.74646561
Q9ULZ3	Apoptosis-associated speck-like protein containing a CARD	2	0.79581284
O95831	Apoptosis-inducing factor 1, mitochondrial	4	0.14063801
Q9UKV3	Apoptotic chromatin condensation inducer in the nucleus	2	0.61710285
P05089	Arginase-1	3	0.65499522
P54136	Arginine--tRNA ligase, cytoplasmic	2	0.98059676
P00966	Argininosuccinate synthase	8	0.55863499
Q7L311	Armadillo repeat-containing X-linked protein 2	2	0.30895816
O43776	Asparagine--tRNA ligase, cytoplasmic	4	0.4583349
P17174	Aspartate aminotransferase, cytoplasmic	8	0.12917371

P00505	Aspartate aminotransferase, mitochondrial	9	0.23863432
Q9ULA0	Aspartyl aminopeptidase	2	0.40376661
Q12797	Aspartyl/asparaginyl beta-hydroxylase	3	0.074501
Q9BXN1	Asporin	3	0.00117922
Q15121	Astrocytic phosphoprotein PEA-15	4	0.72024886
Q6DD88	Atlastin-3	4	0.06826784
P25705	ATP synthase subunit alpha, mitochondrial	9	0.80644754
P06576	ATP synthase subunit beta, mitochondrial	15	0.41330113
O75947	ATP synthase subunit d, mitochondrial	3	0.04008646
O94911	ATP-binding cassette sub-family A member 8	2	0.78072504
Q9NP58	ATP-binding cassette sub-family B member 6, mitochondrial	3	0.88005566
P53396	ATP-citrate synthase	14	0.1999172
P17858	ATP-dependent 6-phosphofructokinase, liver type	4	0.28766595
Q08211	ATP-dependent RNA helicase A	3	0.11352248
Q92499	ATP-dependent RNA helicase DDX1	2	0.31889259
O00571	ATP-dependent RNA helicase DDX3X	2	0.70754596
Q9UII2	ATPase inhibitor, mitochondrial	2	0.69324615
O75882	Attractin	20	0.21102713
Q9UHQ4	B-cell receptor-associated protein 29	2	0.52546806
P51572	B-cell receptor-associated protein 31	6	0.12775219
O95817	BAG family molecular chaperone regulator 3	3	0.97312012
P02730	Band 3 anion transport protein	22	0.309223
O43491	Band 4.1-like protein 2	15	0.31427492
Q9Y2J2	Band 4.1-like protein 3	3	0.44868514
P50895	Basal cell adhesion molecule	8	0.87625153
P98160	Basement membrane-specific heparan sulfate proteoglycan core protein	44	0.32301207
P35613	Basigin	3	0.17453355
P02749	Beta-2-glycoprotein 1	15	0.48795379
P61769	Beta-2-microglobulin	3	0.08951052
P35612	Beta-adducin	8	0.17605623
Q96KN2	Beta-Ala-His dipeptidase	10	0.68438535

P13929	Beta-enolase	8	0.84541851
P08236	Beta-glucuronidase	6	0.15046986
P06865	Beta-hexosaminidase subunit alpha	5	0.0210979
P07686	Beta-hexosaminidase subunit beta	7	0.00968094
P07814	Bifunctional glutamate/proline--tRNA ligase	2	0.9869805
P31939	Bifunctional purine biosynthesis protein PURH	13	0.23788628
P21810	Biglycan	5	0.23273579
P53004	Biliverdin reductase A	5	0.15205609
P43251	Biotinidase	7	0.57356988
P07738	Bisphosphoglycerate mutase	6	0.13952057
Q13867	Bleomycin hydrolase	9	0.13109216
Q9H3K6	BolA-like protein 2	2	0.16027331
P80723	Brain acid soluble protein 1	12	0.25161644
Q9NWV8	BRISC and BRCA1-A complex member 1	2	0.72259106
Q9NW68	BSD domain-containing protein 1	2	0.07289049
Q96CX2	BTB/POZ domain-containing protein KCTD12	6	0.32496536
P11586	C-1-tetrahydrofolate synthase, cytoplasmic	7	0.15497311
P02741	C-reactive protein	4	0.11295923
Q9UBG0	C-type mannose receptor 2	3	0.40655336
P04003	C4b-binding protein alpha chain	15	0.29450547
P20851	C4b-binding protein beta chain	4	0.98023564
P12830	Cadherin-1	8	0.00533632
P55290	Cadherin-13	4	0.18690363
P33151	Cadherin-5	9	0.19860514
Q6NUK1	Calcium-binding mitochondrial carrier protein SCaMC-1	2	0.7974144
Q9Y376	Calcium-binding protein 39	2	0.59577251
Q13557	Calcium/calmodulin-dependent protein kinase type II subunit delta	3	0.93747304
Q05682	Caldesmon	24	0.95223248
P0DP25	Calmodulin-3	8	0.18042553
Q9NZT1	Calmodulin-like protein 5	5	0.11056197
P27824	Calnexin	7	0.4515726
P04632	Calpain small subunit 1	6	0.15648461

P07384	Calpain-1 catalytic subunit	14	0.2688413
P17655	Calpain-2 catalytic subunit	11	0.22975067
P20810	Calpastatin	21	0.45066819
P51911	Calponin-1	8	0.44020661
Q15417	Calponin-3	2	0.69897829
P27797	Calreticulin	14	0.14825895
P22676	Calretinin	10	0.85720899
O43852	Calumenin	11	0.29841927
P17612	cAMP-dependent protein kinase catalytic subunit alpha	2	0.12837599
P10644	cAMP-dependent protein kinase type I-alpha regulatory subunit	4	0.15311806
P13861	cAMP-dependent protein kinase type II-alpha regulatory subunit	5	0.25930794
P31323	cAMP-dependent protein kinase type II-beta regulatory subunit	4	0.31113712
P30622	CAP-Gly domain-containing linker protein 1	4	0.66769657
Q14444	Caprin-1	3	0.71941742
P00915	Carbonic anhydrase 1	15	0.73115369
P00918	Carbonic anhydrase 2	14	0.89053358
P07451	Carbonic anhydrase 3	10	0.8256748
P16152	Carbonyl reductase [NADPH] 1	5	0.04789996
O75828	Carbonyl reductase [NADPH] 3	2	0.55491585
Q96DG6	Carboxymethylenebutenolidase homolog	2	0.32942923
P15086	Carboxypeptidase B	5	0.2238649
Q96IY4	Carboxypeptidase B2	3	0.88984788
P22792	Carboxypeptidase N subunit 2	8	0.08595589
Q9Y646	Carboxypeptidase Q	3	0.01656365
Q9NQ79	Cartilage acidic protein 1	3	0.02596048
P49747	Cartilage oligomeric matrix protein	7	0.62601079
P31944	Caspase-14	2	0.15461944
P04040	Catalase	30	0.69684169
P07858	Cathepsin B	6	0.26878561
P07339	Cathepsin D	7	0.44160389
P08311	Cathepsin G	4	0.6843597
P07711	Cathepsin L1	5	0.60762585

P25774	Cathepsin S	4	0.17630526
Q9UBR2	Cathepsin Z	4	0.10070267
Q6NZI2	Caveolae-associated protein 1	11	0.68214819
O95810	Caveolae-associated protein 2	13	0.38388163
Q969G5	Caveolae-associated protein 3	5	0.05071765
Q03135	Caveolin-1	5	0.12331245
Q6YHK3	CD109 antigen	3	0.81637134
Q13740	CD166 antigen	8	0.00469949
Q9Y5K6	CD2-associated protein	2	0.77861344
Q9NNX6	CD209 antigen	4	0.58535688
Q8IX05	CD302 antigen	2	0.41327224
P16070	CD44 antigen	7	0.78374639
O43866	CD5 antigen-like	11	0.41233668
P13987	CD59 glycoprotein	3	0.86280795
Q9BY67	Cell adhesion molecule 1	2	0.03185789
Q8N126	Cell adhesion molecule 3	2	0.66055147
P60953	Cell division control protein 42 homolog	5	0.09001179
P43121	Cell surface glycoprotein MUC18	15	0.71052953
P62633	Cellular nucleic acid-binding protein	2	0.36964088
O94986	Centrosomal protein of 152 kDa	2	0.10929485
P00450	Ceruloplasmin	43	0.11135234
Q9BY43	Charged multivesicular body protein 4a	5	0.09388169
Q9NZZ3	Charged multivesicular body protein 5	2	0.78772478
O00299	Chloride intracellular channel protein 1	8	0.19885046
Q9Y696	Chloride intracellular channel protein 4	4	0.34786168
P06276	Cholinesterase	8	0.36682725
Q6UVK1	Chondroitin sulfate proteoglycan 4	3	0.12785412
P83916	Chromobox protein homolog 1	2	0.41183149
Q13185	Chromobox protein homolog 3	3	0.19505823
P23946	Chymase	5	0.83449296
O75390	Citrate synthase, mitochondrial	6	0.92131769
Q00610	Clathrin heavy chain 1	29	0.0639681

P09496	Clathrin light chain A	5	0.55249153
P09497	Clathrin light chain B	4	0.82511848
P10909	Clusterin	14	0.4647994
Q8TDQ1	CMRF35-like molecule 1	2	0.19766776
Q14019	Coactosin-like protein	10	0.79922282
P00740	Coagulation factor IX	3	0.86976252
P12259	Coagulation factor V	2	0.21917543
P00742	Coagulation factor X	2	0.88205406
P03951	Coagulation factor XI	6	0.33652763
P00748	Coagulation factor XII	4	0.1097847
P00488	Coagulation factor XIII A chain	12	0.21017446
P05160	Coagulation factor XIII B chain	14	0.40024073
P53621	Coatomer subunit alpha	3	0.27783474
P35606	Coatomer subunit beta'	5	0.8729405
P48444	Coatomer subunit delta	6	0.08862598
O14579	Coatomer subunit epsilon	3	0.10745245
Q9Y678	Coatomer subunit gamma-1	3	0.40336273
P23528	Cofilin-1	9	0.13304405
Q9Y281	Cofilin-2	3	0.24438349
Q76M96	Coiled-coil domain-containing protein 80	2	0.91374915
Q9Y6H1	Coiled-coil-helix-coiled-coil-helix domain-containing protein 2	2	0.41573988
Q14011	Cold-inducible RNA-binding protein	3	0.10470682
P02452	Collagen alpha-1(I) chain	17	0.36385151
P02461	Collagen alpha-1(III) chain	11	0.55975442
P20908	Collagen alpha-1(V) chain	3	0.02319357
P12109	Collagen alpha-1(VI) chain	7	0.14741071
Q99715	Collagen alpha-1(XII) chain	2	0.05050746
Q05707	Collagen alpha-1(XIV) chain	34	0.13318118
P39059	Collagen alpha-1(XV) chain	6	0.33047189
P39060	Collagen alpha-1(XVIII) chain	7	0.98732137
P08123	Collagen alpha-2(I) chain	12	0.91690989
P08572	Collagen alpha-2(IV) chain	2	0.36452741

P12110	Collagen alpha-2(VI) chain	3	0.07635945
P25940	Collagen alpha-3(V) chain	2	0.62956019
P12111	Collagen alpha-3(VI) chain	66	0.05637282
A6NMZ7	Collagen alpha-6(VI) chain	9	0.20544257
Q96CG8	Collagen triple helix repeat-containing protein 1	4	0.10666205
Q5KU26	Collectin-12	2	0.98460635
P02745	Complement C1q subcomponent subunit A	2	0.24244069
P02746	Complement C1q subcomponent subunit B	3	0.23120921
P02747	Complement C1q subcomponent subunit C	4	0.20221089
P00736	Complement C1r subcomponent	14	0.12386937
P09871	Complement C1s subcomponent	12	0.11030729
P06681	Complement C2	17	0.29602422
P01024	Complement C3	87	0.21386399
P0C0L4	Complement C4-A	3	0.23696547
P0C0L5	Complement C4-B	4	0.28075423
P01031	Complement C5	27	0.64443114
Q07021	Complement component 1 Q subcomponent-binding protein, mitochondrial	3	0.22269391
Q9NPY3	Complement component C1q receptor	2	0.65315845
P13671	Complement component C6	17	0.03987187
P10643	Complement component C7	8	0.09831379
P07357	Complement component C8 alpha chain	10	0.17071596
P07358	Complement component C8 beta chain	9	0.09208016
P07360	Complement component C8 gamma chain	4	0.20029294
P02748	Complement component C9	11	0.87358559
P08174	Complement decay-accelerating factor	7	0.13047172
P00751	Complement factor B	27	0.22447037
P00746	Complement factor D	4	0.72072495
P08603	Complement factor H	48	0.64239095
P36980	Complement factor H-related protein 2	5	0.82377841
Q92496	Complement factor H-related protein 4	2	0.54547594
Q9BXR6	Complement factor H-related protein 5	4	0.25379005

P05156	Complement factor I	14	0.72435071
Q9NZB2	Constitutive coactivator of PPAR-gamma-like protein 1	2	0.71880456
Q13098	COP9 signalosome complex subunit 1	3	0.11778651
P61201	COP9 signalosome complex subunit 2	2	0.8225113
Q9BT78	COP9 signalosome complex subunit 4	5	0.28083863
Q92905	COP9 signalosome complex subunit 5	3	0.19026733
O14618	Copper chaperone for superoxide dismutase	3	0.09893461
O75367	Core histone macro-H2A.1	6	0.3279944
P31146	Coronin-1A	6	0.11323679
Q9ULV4	Coronin-1C	6	0.12665232
P57737	Coronin-7	2	0.39520876
P08185	Corticosteroid-binding globulin	5	0.50994274
P12277	Creatine kinase B-type	10	0.6436452
P06732	Creatine kinase M-type	12	0.74667161
P46109	Crk-like protein	3	0.13897515
Q13618	Cullin-3	4	0.11314975
Q86VP6	Cullin-associated NEDD8-dissociated protein 1	14	0.44391355
Q9NWV4	CXXC motif containing zinc binding protein	4	0.86594026
P01040	Cystatin-A	3	0.37609009
P04080	Cystatin-B	4	0.95612162
P01034	Cystatin-C	3	0.93767104
P21291	Cysteine and glycine-rich protein 1	5	0.47602005
P52943	Cysteine-rich protein 2	3	0.0308436
P54108	Cysteine-rich secretory protein 3	2	0.12102424
Q6UXH1	Cysteine-rich with EGF-like domain protein 2	2	0.24036955
P00167	Cytochrome b5	3	0.79176319
P99999	Cytochrome c	3	0.29045869
P14854	Cytochrome c oxidase subunit 6B1	2	0.90147475
Q8IUI8	Cytokine receptor-like factor 3	2	0.23990433
P21399	Cytoplasmic aconitate hydratase	24	0.27769402
Q14204	Cytoplasmic dynein 1 heavy chain 1	12	0.08363332
Q13409	Cytoplasmic dynein 1 intermediate chain 2	3	0.80901834

Q07065	Cytoskeleton-associated protein 4	3	0.20065311
P28838	Cytosol aminopeptidase	20	0.02638497
O75891	Cytosolic 10-formyltetrahydrofolate dehydrogenase	10	0.09407448
Q9H5X1	Cytosolic iron-sulfur assembly component 2A	2	0.26971416
Q96KP4	Cytosolic non-specific dipeptidase	19	0.04674425
O43175	D-3-phosphoglycerate dehydrogenase	10	0.59670491
Q96GG9	DCN1-like protein 1	4	0.4669293
Q96HY6	DDRGK domain-containing protein 1	2	0.64021573
P07585	Decorin	9	0.0704782
P30038	Delta-1-pyrroline-5-carboxylate dehydrogenase, mitochondrial	2	0.14346217
P13716	Delta-aminolevulinic acid dehydratase	8	0.50795975
Q13011	Delta(3,5)-Delta(2,4)-dienoyl-CoA isomerase, mitochondrial	3	0.03413936
Q08495	Dematin	10	0.07524045
O43583	Density-regulated protein	2	0.61866586
Q9Y3Z3	Deoxynucleoside triphosphate triphosphohydrolase SAMHD1	4	0.81367803
O00115	Deoxyribonuclease-2-alpha	3	0.17755623
Q07507	Dermatopontin	7	0.69931941
P81605	Dermcidin	3	0.88688693
P17661	Desmin	20	0.63743946
Q14126	Desmoglein-2	6	0.00063061
P15924	Desmoplakin	7	0.12629261
P60981	Destrin	6	0.59474322
Q01459	Di-N-acetylchitobiase	2	0.05875975
Q9UBP4	Dickkopf-related protein 3	2	0.56159479
P09622	Dihydrolipoyl dehydrogenase, mitochondrial	7	0.18651259
P36957	Dihydrolipoyllysine-residue succinyltransferase component of 2-oxoglutarate dehydrogenase complex, mitochondrial	2	0.22502963
P09417	Dihydropteridine reductase	3	0.15596525
Q16555	Dihydropyrimidinase-related protein 2	6	0.149508
Q14195	Dihydropyrimidinase-related protein 3	8	0.13709695
P53634	Dipeptidyl peptidase 1	3	0.16805362
Q9UHL4	Dipeptidyl peptidase 2	2	0.15229993

Q9NY33	Dipeptidyl peptidase 3	15	0.39381303
P27487	Dipeptidyl peptidase 4	8	0.16697924
P53602	Diphosphomevalonate decarboxylase	2	0.95650434
P98082	Disabled homolog 2	6	0.92707187
Q16531	DNA damage-binding protein 1	24	0.13298096
P27695	DNA-(apurinic or apyrimidinic site) lyase	10	0.04816902
O60884	DnaJ homolog subfamily A member 2	5	0.06109694
P25685	DnaJ homolog subfamily B member 1	3	0.75281835
Q9UBS4	DnaJ homolog subfamily B member 11	3	0.48002144
P25686	DnaJ homolog subfamily B member 2	2	0.58898637
O75190	DnaJ homolog subfamily B member 6	2	0.38708358
O75937	DnaJ homolog subfamily C member 8	3	0.21006182
Q8WXX5	DnaJ homolog subfamily C member 9	2	0.31790563
P04843	Dolichyl-diphosphooligosaccharide--protein glycosyltransferase subunit 1	3	0.44590438
Q16643	Drebrin	8	0.78712025
Q9UJU6	Drebrin-like protein	8	0.70210036
Q02750	Dual specificity mitogen-activated protein kinase kinase 1	3	0.00346327
P46734	Dual specificity mitogen-activated protein kinase kinase 3	2	0.23707557
Q14203	Dynactin subunit 1	9	0.30260215
Q13561	Dynactin subunit 2	10	0.45444103
O75935	Dynactin subunit 3	2	0.39375855
O00429	Dynamin-1-like protein	2	0.19585342
P50570	Dynamin-2	3	0.53305869
O60313	Dynamin-like 120 kDa protein, mitochondrial	2	0.24237533
Q96FJ2	Dynein light chain 2, cytoplasmic	2	0.08055866
P11532	Dystrophin	6	0.40529398
Q7Z6Z7	E3 ubiquitin-protein ligase HUWE1	3	0.31917447
Q5XPI4	E3 ubiquitin-protein ligase RNF123	2	0.07569393
Q63HN8	E3 ubiquitin-protein ligase RNF213	2	0.25204054
Q5T4S7	E3 ubiquitin-protein ligase UBR4	2	0.14973419
Q15075	Early endosome antigen 1	25	0.59749954

O95834	Echinoderm microtubule-associated protein-like 2	2	0.26005282
Q96C19	EF-hand domain-containing protein D2	3	0.4303332
Q12805	EGF-containing fibulin-like extracellular matrix protein 1	13	0.78699318
O95967	EGF-containing fibulin-like extracellular matrix protein 2	2	0.41581411
Q9H4M9	EH domain-containing protein 1	5	0.642451
Q9NZN4	EH domain-containing protein 2	19	0.03940865
P13804	Electron transfer flavoprotein subunit alpha, mitochondrial	3	0.41657878
P38117	Electron transfer flavoprotein subunit beta	4	0.07650975
P68104	Elongation factor 1-alpha 1	5	0.42213337
P24534	Elongation factor 1-beta	4	0.68457144
P29692	Elongation factor 1-delta	2	0.67501799
P26641	Elongation factor 1-gamma	5	0.18135861
P13639	Elongation factor 2	13	0.39514403
P49411	Elongation factor Tu, mitochondrial	3	0.26812715
Q15370	Elongin-B	2	0.31425918
Q15369	Elongin-C	2	0.45247867
Q9Y6C2	EMILIN-1	2	0.22350817
Q9UI08	Ena/VASP-like protein	2	0.16949421
O94919	Endonuclease domain-containing 1 protein	4	0.78562102
Q9NZ08	Endoplasmic reticulum aminopeptidase 1	4	0.18625171
P11021	Endoplasmic reticulum chaperone BiP	24	0.38021778
P30040	Endoplasmic reticulum resident protein 29	3	0.35757394
Q9BS26	Endoplasmic reticulum resident protein 44	2	0.23836174
P14625	Endoplasmin	13	0.15164413
Q9HCU0	Endosialin	3	0.67593233
O60869	Endothelial differentiation-related factor 1	3	0.21547638
Q9UNN8	Endothelial protein C receptor	2	0.17132794
P84090	Enhancer of rudimentary homolog	2	0.58572422
Q9UHY7	Enolase-phosphatase E1	3	0.77544082
P30084	Enoyl-CoA hydratase, mitochondrial	7	0.50206618
P42566	Epidermal growth factor receptor substrate 15	6	0.41351611
Q9UBC2	Epidermal growth factor receptor substrate 15-like 1	2	0.29812739

P07099	Epoxide hydrolase 1	7	0.15334844
P27105	Erythrocyte band 7 integral membrane protein	10	0.43970784
P16452	Erythrocyte membrane protein band 4.2	17	0.27777199
Q9H0W9	Ester hydrolase C11orf54	5	0.16758768
Q9NTX5	Ethylmalonyl-CoA decarboxylase	6	0.07991809
P60842	Eukaryotic initiation factor 4A-I	2	0.21031168
Q14240	Eukaryotic initiation factor 4A-II	3	0.8484653
P15170	Eukaryotic peptide chain release factor GTP-binding subunit ERF3A	2	0.14119413
P20042	Eukaryotic translation initiation factor 2 subunit 2	5	0.42407706
P41091	Eukaryotic translation initiation factor 2 subunit 3	2	0.38946666
Q14152	Eukaryotic translation initiation factor 3 subunit A	3	0.86591081
P55884	Eukaryotic translation initiation factor 3 subunit B	2	0.71679808
O15371	Eukaryotic translation initiation factor 3 subunit D	3	0.14283587
Q04637	Eukaryotic translation initiation factor 4 gamma 1	3	0.90081541
P23588	Eukaryotic translation initiation factor 4B	3	0.23963166
Q15056	Eukaryotic translation initiation factor 4H	2	0.69534796
P55010	Eukaryotic translation initiation factor 5	3	0.55813897
P63241	Eukaryotic translation initiation factor 5A-1	9	0.3620009
P56537	Eukaryotic translation initiation factor 6	3	0.83707291
O14980	Exportin-1	4	0.16795101
Q9BSJ8	Extended synaptotagmin-1	11	0.77454281
Q16610	Extracellular matrix protein 1	11	0.50586635
P08294	Extracellular superoxide dismutase [Cu-Zn]	7	0.20847775
P15311	Ezrin	7	0.66731421
P52907	F-actin-capping protein subunit alpha-1	5	0.10350035
P47755	F-actin-capping protein subunit alpha-2	4	0.28794334
P47756	F-actin-capping protein subunit beta	8	0.16966199
Q92945	Far upstream element-binding protein 2	5	0.78338109
Q16658	Fascin	17	0.22769613
P49327	Fatty acid synthase	36	0.53126714
Q01469	Fatty acid-binding protein 5	9	0.28284751
P15090	Fatty acid-binding protein, adipocyte	10	0.14447231

P05413	Fatty acid-binding protein, heart	2	0.35994995
P51648	Fatty aldehyde dehydrogenase	3	0.38443675
Q96AC1	Fermitin family homolog 2	13	0.12906856
Q86UX7	Fermitin family homolog 3	7	0.06572818
P02794	Ferritin heavy chain	7	0.02077203
P02792	Ferritin light chain	8	0.02698741
P35555	Fibrillin-1	43	0.33663955
P02671	Fibrinogen alpha chain	36	0.31228252
P02675	Fibrinogen beta chain	41	0.30258981
P02679	Fibrinogen gamma chain	32	0.42079586
Q14314	Fibroleukin	2	0.78517854
P02751	Fibronectin	61	0.32728167
Q4ZHG4	Fibronectin type III domain-containing protein 1	5	0.17275103
P23142	Fibulin-1	12	0.45377421
P98095	Fibulin-2	9	0.98315821
Q9UBX5	Fibulin-5	2	0.97372244
Q15485	Ficolin-2	2	0.28115553
O75636	Ficolin-3	4	0.51708978
P20930	Filaggrin	2	0.21475309
Q5D862	Filaggrin-2	3	0.0003644
P21333	Filamin-A	96	0.38797417
O75369	Filamin-B	33	0.40771295
Q14315	Filamin-C	11	0.71038185
P30043	Flavin reductase (NADPH)	9	0.37581171
O75955	Flotillin-1	4	0.03662008
Q14254	Flotillin-2	8	0.56837389
Q12841	Follistatin-related protein 1	4	0.83437435
Q96RU3	Formin-binding protein 1	2	0.35393496
Q13642	Four and a half LIM domains protein 1	10	0.07730029
Q9H479	Fructosamine-3-kinase	4	0.15312066
P09467	Fructose-1,6-bisphosphatase 1	9	0.03470547
P04075	Fructose-bisphosphate aldolase A	10	0.21327887

P09972	Fructose-bisphosphate aldolase C	7	0.96140872
P07954	Fumarate hydratase, mitochondrial	5	0.50310349
P16930	Fumarylacetoacetase	12	0.28814504
Q9BQS8	FYVE and coiled-coil domain-containing protein 1	2	0.17684518
P51570	Galactokinase	2	0.41024721
P09382	Galectin-1	6	0.66195688
P17931	Galectin-3	2	0.18641034
Q08380	Galectin-3-binding protein	10	0.00228268
P47929	Galectin-7	3	0.26663484
P09104	Gamma-enolase	10	0.23427596
Q92820	Gamma-glutamyl hydrolase	6	0.04225729
O75223	Gamma-glutamylcyclotransferase	5	0.66432046
O76070	Gamma-synuclein	6	0.28832183
Q13630	GDP-L-fucose synthase	5	0.92396215
P06396	Gelsolin	13	0.06843788
O60763	General vesicular transport factor p115	3	0.43475955
P60983	Glia maturation factor beta	3	0.26386742
P11413	Glucose-6-phosphate 1-dehydrogenase	2	0.10195785
P06744	Glucose-6-phosphate isomerase	21	0.12862929
P14314	Glucosidase 2 subunit beta	10	0.834417
P00367	Glutamate dehydrogenase 1, mitochondrial	14	0.08072552
P48506	Glutamate--cysteine ligase catalytic subunit	14	0.24567263
P48507	Glutamate--cysteine ligase regulatory subunit	2	0.23440543
P15104	Glutamine synthetase	3	0.78850208
O76003	Glutaredoxin-3	6	0.11044628
P07203	Glutathione peroxidase 1	5	0.33762193
P22352	Glutathione peroxidase 3	5	0.11893947
P00390	Glutathione reductase, mitochondrial	12	0.16543545
O43813	Glutathione S-transferase LANCL1	4	0.3037381
P09488	Glutathione S-transferase Mu 1	2	0.29400822
P28161	Glutathione S-transferase Mu 2	5	0.469014
P21266	Glutathione S-transferase Mu 3	8	0.70210907

P78417	Glutathione S-transferase omega-1	9	0.23610075
P09211	Glutathione S-transferase P	10	0.14111184
P48637	Glutathione synthetase	6	0.38174949
P04406	Glyceraldehyde-3-phosphate dehydrogenase	17	0.96686616
P21695	Glycerol-3-phosphate dehydrogenase [NAD(+)], cytoplasmic	23	0.14013653
A6NDG6	Glycerol-3-phosphate phosphatase	3	0.44932736
P11216	Glycogen phosphorylase, brain form	7	0.28843832
P06737	Glycogen phosphorylase, liver form	20	0.1164646
P11217	Glycogen phosphorylase, muscle form	2	0.62150276
P02724	Glycophorin-A	3	0.2285438
Q9HC38	Glyoxalase domain-containing protein 4	7	0.07566348
Q9UBQ7	Glyoxylate reductase/hydroxypyruvate reductase	5	0.37809742
P35052	Glypican-1	2	0.4218924
Q9H4G4	Golgi-associated plant pathogenesis-related protein 1	5	0.13698936
Q14789	Golgin subfamily B member 1	3	0.57474663
P62993	Growth factor receptor-bound protein 2	7	0.27623884
P62826	GTP-binding nuclear protein Ran	5	0.23447976
Q14C86	GTPase-activating protein and VPS9 domain-containing protein 1	2	0.28520523
P63096	Guanine nucleotide-binding protein G(i) subunit alpha-1	2	0.40363765
P04899	Guanine nucleotide-binding protein G(i) subunit alpha-2	4	0.09425912
P62873	Guanine nucleotide-binding protein G(I)/G(S)/G(T) subunit beta-1	3	0.44925129
P62879	Guanine nucleotide-binding protein G(I)/G(S)/G(T) subunit beta-2	5	0.1734526
P08754	Guanine nucleotide-binding protein G(k) subunit alpha	2	0.67904939
Q5JWF2	Guanine nucleotide-binding protein G(s) subunit alpha isoforms XLas	3	0.10417006
Q9H0R4	Haloacid dehalogenase-like hydrolase domain-containing protein 2	4	0.29243961
P00738	Haptoglobin	14	0.05170057
P00739	Haptoglobin-related protein	6	0.49280498
Q53T59	HCLS1-binding protein 3	2	0.19341269
O43301	Heat shock 70 kDa protein 12A	4	0.12104491
P0DMV9	Heat shock 70 kDa protein 1B	20	0.22028819
P34932	Heat shock 70 kDa protein 4	20	0.13097132
P11142	Heat shock cognate 71 kDa protein	16	0.11937789

P04792	Heat shock protein beta-1	10	0.17841033
O14558	Heat shock protein beta-6	3	0.25409193
P07900	Heat shock protein HSP 90-alpha	10	0.4154026
P08238	Heat shock protein HSP 90-beta	9	0.60175612
P14317	Hematopoietic lineage cell-specific protein	2	0.58587622
P09601	Heme oxygenase 1	2	0.20936151
P30519	Heme oxygenase 2	2	0.34368027
Q9NRV9	Heme-binding protein 1	2	0.07394228
Q9Y5Z4	Heme-binding protein 2	8	0.67846875
P69905	Hemoglobin subunit alpha	12	0.57382676
P68871	Hemoglobin subunit beta	13	0.7319185
P02042	Hemoglobin subunit delta	8	0.41043231
P69891	Hemoglobin subunit gamma-1	10	0.89111324
P09105	Hemoglobin subunit theta-1	2	0.46326943
P02008	Hemoglobin subunit zeta	4	0.41152668
P02790	Hemopexin	29	0.05430655
P05546	Heparin cofactor 2	10	0.04740587
P26927	Hepatocyte growth factor-like protein	2	0.7809371
P51858	Hepatoma-derived growth factor	9	0.16146745
Q7Z4V5	Hepatoma-derived growth factor-related protein 2	2	0.4098159
Q5SSJ5	Heterochromatin protein 1-binding protein 3	2	0.15393677
Q99729	Heterogeneous nuclear ribonucleoprotein A/B	2	0.0482488
P09651	Heterogeneous nuclear ribonucleoprotein A1	5	0.5615609
P51991	Heterogeneous nuclear ribonucleoprotein A3	3	0.88673855
O14979	Heterogeneous nuclear ribonucleoprotein D-like	2	0.66032334
Q14103	Heterogeneous nuclear ribonucleoprotein D0	3	0.93346618
P52597	Heterogeneous nuclear ribonucleoprotein F	2	0.6775214
P31943	Heterogeneous nuclear ribonucleoprotein H	3	0.84926352
P31942	Heterogeneous nuclear ribonucleoprotein H3	4	0.8022048
P61978	Heterogeneous nuclear ribonucleoprotein K	9	0.24879301
P14866	Heterogeneous nuclear ribonucleoprotein L	2	0.97074988
P52272	Heterogeneous nuclear ribonucleoprotein M	2	0.52695092

O60506	Heterogeneous nuclear ribonucleoprotein Q	7	0.57739112
O43390	Heterogeneous nuclear ribonucleoprotein R	4	0.62051184
Q00839	Heterogeneous nuclear ribonucleoprotein U	9	0.35897388
Q1KMD3	Heterogeneous nuclear ribonucleoprotein U-like protein 2	5	0.46037041
P22626	Heterogeneous nuclear ribonucleoproteins A2/B1	12	0.45902452
P07910	Heterogeneous nuclear ribonucleoproteins C1/C2	5	0.19220647
P19367	Hexokinase-1	10	0.75197782
P09429	High mobility group protein B1	2	0.1325581
P50135	Histamine N-methyltransferase	3	0.16634065
P49773	Histidine triad nucleotide-binding protein 1	2	0.11353657
P12081	Histidine--tRNA ligase, cytoplasmic	2	0.18094968
P04196	Histidine-rich glycoprotein	9	0.40697232
P10412	Histone H1.4	2	0.4396753
P16401	Histone H1.5	2	0.25216481
P06899	Histone H2B type 1-J	2	0.13551238
O60814	Histone H2B type 1-K	2	0.18537572
P68431	Histone H3.1	3	0.19593599
P62805	Histone H4	8	0.12427045
P01892	HLA class I histocompatibility antigen, A-2 alpha chain	2	0.6380923
P04439	HLA class I histocompatibility antigen, A-3 alpha chain	2	0.11998537
P30481	HLA class I histocompatibility antigen, B-44 alpha chain	2	0.98720556
P01889	HLA class I histocompatibility antigen, B-7 alpha chain	2	0.71745939
P04440	HLA class II histocompatibility antigen, DP beta 1 chain	2	0.19104293
P01903	HLA class II histocompatibility antigen, DR alpha chain	2	0.64042876
Q05469	Hormone-sensitive lipase	12	0.01267548
P50502	Hsc70-interacting protein	9	0.42177228
Q16543	Hsp90 co-chaperone Cdc37	5	0.67301795
Q14520	Hyaluronan-binding protein 2	7	0.74303578
Q16836	Hydroxyacyl-coenzyme A dehydrogenase, mitochondrial	5	0.13002026
Q16775	Hydroxyacylglutathione hydrolase, mitochondrial	7	0.40229373
P00492	Hypoxanthine-guanine phosphoribosyltransferase	3	0.28668684
Q9Y4L1	Hypoxia up-regulated protein 1	6	0.73869815

Q9Y6R7	IgGFc-binding protein	5	0.4650877
P0DOX2	Immunoglobulin alpha-2 heavy chain	9	0.47889613
P0DOX3	Immunoglobulin delta heavy chain	8	0.72738951
P0DOX4	Immunoglobulin epsilon heavy chain	2	0.35065926
P0DOX5	Immunoglobulin gamma-1 heavy chain	17	0.05546967
P01876	Immunoglobulin heavy constant alpha 1	8	0.86723442
P01859	Immunoglobulin heavy constant gamma 2	11	0.2225638
P01860	Immunoglobulin heavy constant gamma 3	5	0.65760123
P01861	Immunoglobulin heavy constant gamma 4	5	0.2648515
P01871	Immunoglobulin heavy constant mu	21	0.06522658
A0A0C4DH33	Immunoglobulin heavy variable 1-24	2	0.10272682
A0A0C4DH29	Immunoglobulin heavy variable 1-3	2	0.22437043
P01742	Immunoglobulin heavy variable 1-69	2	0.73160953
P0DP01	Immunoglobulin heavy variable 1-8	2	0.20871339
A0A0C4DH43	Immunoglobulin heavy variable 2-70D	2	0.73250235
A0A0B4J1V0	Immunoglobulin heavy variable 3-15	4	0.22936356
A0A0C4DH32	Immunoglobulin heavy variable 3-20	2	0.29576306
P0DP03	Immunoglobulin heavy variable 3-30-5	2	0.19439149
A0A0A0MS15	Immunoglobulin heavy variable 3-49	5	0.30368095
A0A0J9YX35	Immunoglobulin heavy variable 3-64D	2	0.07599948
P01780	Immunoglobulin heavy variable 3-7	3	0.11154927
A0A0B4J1Y9	Immunoglobulin heavy variable 3-72	4	0.08048071
P01782	Immunoglobulin heavy variable 3-9	3	0.07486574
A0A0C4DH34	Immunoglobulin heavy variable 4-28	2	0.16108447
A0A0C4DH38	Immunoglobulin heavy variable 5-51	3	0.08403592
A0A0B4J1U7	Immunoglobulin heavy variable 6-1	2	0.21617526
P01591	Immunoglobulin J chain	4	0.27527815
P01834	Immunoglobulin kappa constant	2	0.10142351
P0DOX7	Immunoglobulin kappa light chain	3	0.17442312
P01593	Immunoglobulin kappa variable 1D-33	2	0.12983938
A0A0A0MRZ8	Immunoglobulin kappa variable 3D-11	2	0.09691376
A0A087WSY6	Immunoglobulin kappa variable 3D-15	2	0.1899612

P06312	Immunoglobulin kappa variable 4-1	3	0.25026754
P0DOY2	Immunoglobulin lambda constant 2	5	0.15982805
P01700	Immunoglobulin lambda variable 1-47	2	0.05937982
P01701	Immunoglobulin lambda variable 1-51	2	0.06033041
A0A075B6J9	Immunoglobulin lambda variable 2-18	2	0.12498196
A0A075B6K4	Immunoglobulin lambda variable 3-10	4	0.14466028
A0A075B6K5	Immunoglobulin lambda variable 3-9	3	0.11073294
A0A075B6I0	Immunoglobulin lambda variable 8-61	2	0.98477332
P0DOX8	Immunoglobulin lambda-1 light chain	3	0.13545592
O00629	Importin subunit alpha-3	2	0.12160548
Q14974	Importin subunit beta-1	5	0.1243411
O00410	Importin-5	5	0.10406976
O95373	Importin-7	3	0.31430962
Q8NBJ7	Inactive C-alpha-formylglycine-generating enzyme 2	2	0.75193868
P08476	Inhibin beta A chain	3	0.90274017
Q15181	Inorganic pyrophosphatase	5	0.15303017
P14735	Insulin-degrading enzyme	4	0.99465421
P17936	Insulin-like growth factor-binding protein 3	3	0.76064321
P24593	Insulin-like growth factor-binding protein 5	5	0.51174611
Q16270	Insulin-like growth factor-binding protein 7	5	0.72473771
P35858	Insulin-like growth factor-binding protein complex acid labile subunit	5	0.81957486
P08514	Integrin alpha-IIb	4	0.11578393
P05556	Integrin beta-1	5	0.09507996
P05106	Integrin beta-3	6	0.06908856
Q13418	Integrin-linked protein kinase	3	0.07133386
Q8WWA0	Intelectin-1	2	0.84143757
P19827	Inter-alpha-trypsin inhibitor heavy chain H1	15	0.29006087
P19823	Inter-alpha-trypsin inhibitor heavy chain H2	16	0.19643962
Q06033	Inter-alpha-trypsin inhibitor heavy chain H3	5	0.08630078
Q14624	Inter-alpha-trypsin inhibitor heavy chain H4	18	0.25493121
P05362	Intercellular adhesion molecule 1	2	0.2869258
Q12905	Interleukin enhancer-binding factor 2	2	0.78898553

Q12906	Interleukin enhancer-binding factor 3	5	0.81708123
P05231	Interleukin-6	6	0.85896766
P10145	Interleukin-8	2	0.97265687
Q15811	Intersectin-1	3	0.18377275
P03956	Interstitial collagenase	3	0.28992426
Q27J81	Inverted formin-2	4	0.09273462
O75874	Isocitrate dehydrogenase [NADP] cytoplasmic	16	0.97624897
P53990	IST1 homolog	2	0.30357821
P14923	Junction plakoglobin	4	0.15941259
P29622	Kallistatin	7	0.41841476
P13645	Keratin, type I cytoskeletal 10	24	0.01288076
P02533	Keratin, type I cytoskeletal 14	5	0.15098699
P08779	Keratin, type I cytoskeletal 16	8	0.20271271
Q04695	Keratin, type I cytoskeletal 17	3	0.19399893
P05783	Keratin, type I cytoskeletal 18	2	0.42847111
P08727	Keratin, type I cytoskeletal 19	10	0.17203746
P35527	Keratin, type I cytoskeletal 9	21	0.03561061
P04264	Keratin, type II cytoskeletal 1	30	0.02280225
Q7Z794	Keratin, type II cytoskeletal 1b	2	0.21459872
P35908	Keratin, type II cytoskeletal 2 epidermal	22	0.0167309
P13647	Keratin, type II cytoskeletal 5	8	0.06096195
P02538	Keratin, type II cytoskeletal 6A	8	0.23311281
Q8N1N4	Keratin, type II cytoskeletal 78	2	0.02702244
P05787	Keratin, type II cytoskeletal 8	11	0.15886073
Q9HA64	Ketosamine-3-kinase	3	0.33262654
Q86UP2	Kinectin	10	0.72580093
P33176	Kinesin-1 heavy chain	10	0.14008586
Q96L93	Kinesin-like protein KIF16B	2	0.53374399
P01042	Kininogen-1	24	0.33320166
Q63ZY3	KN motif and ankyrin repeat domain-containing protein 2	5	0.67055716
Q6NY19	KN motif and ankyrin repeat domain-containing protein 3	3	0.85182599
Q5T7N3	KN motif and ankyrin repeat domain-containing protein 4	2	0.71719055

Q6YP21	Kynurenine--oxoglutarate transaminase 3	2	0.26006545
P00338	L-lactate dehydrogenase A chain	15	0.34093016
P07195	L-lactate dehydrogenase B chain	13	0.57435178
P14151	L-selectin	7	0.26562991
Q7Z4W1	L-xylulose reductase	3	0.1741262
P02788	Lactotransferrin	40	0.16249168
Q04760	Lactoylglutathione lyase	6	0.31313426
P20700	Lamin-B1	11	0.85522168
Q03252	Lamin-B2	13	0.69294852
P24043	Laminin subunit alpha-2	2	0.83314699
Q16363	Laminin subunit alpha-4	23	0.12966835
P07942	Laminin subunit beta-1	25	0.14101259
P55268	Laminin subunit beta-2	22	0.12475998
P11047	Laminin subunit gamma-1	38	0.18188231
P46379	Large proline-rich protein BAG6	2	0.8394332
Q14766	Latent-transforming growth factor beta-binding protein 1	15	0.73960171
Q14767	Latent-transforming growth factor beta-binding protein 2	10	0.15438064
Q8N2S1	Latent-transforming growth factor beta-binding protein 4	8	0.4994996
Q9BS40	Latexin	2	0.58343981
P29536	Leiomodin-1	2	0.84110517
P02750	Leucine-rich alpha-2-glycoprotein	6	0.01919289
Q32MZ4	Leucine-rich repeat flightless-interacting protein 1	2	0.28597918
P30740	Leukocyte elastase inhibitor	4	0.11140858
Q08722	Leukocyte surface antigen CD47	2	0.12012227
P09960	Leukotriene A-4 hydrolase	21	0.11088032
Q9NZU5	LIM and cysteine-rich domains protein 1	2	0.1211019
P48059	LIM and senescent cell antigen-like-containing domain protein 1	3	0.02173024
Q14847	LIM and SH3 domain protein 1	8	0.95836086
Q9UHB6	LIM domain and actin-binding protein 1	2	0.78005394
P31025	Lipocalin-1	2	0.593581
Q93052	Lipoma-preferred partner	6	0.32775059
P18428	Lipopolysaccharide-binding protein	2	0.86931396

P23141	Liver carboxylesterase 1	21	0.55563985
P33121	Long-chain-fatty-acid--CoA ligase 1	15	0.1277562
O95573	Long-chain-fatty-acid--CoA ligase 3	3	0.88140617
P08637	Low affinity immunoglobulin gamma Fc region receptor III-A	4	0.02953283
P24666	Low molecular weight phosphotyrosine protein phosphatase	6	0.11770433
Q14696	LRP chaperone MESD	2	0.87896211
P51884	Lumican	13	0.83030163
P05455	Lupus La protein	4	0.72227913
Q9Y5Y7	Lymphatic vessel endothelial hyaluronic acid receptor 1	8	0.26012686
P33241	Lymphocyte-specific protein 1	5	0.4678996
O00754	Lysosomal alpha-mannosidase	3	0.1243801
P11279	Lysosome-associated membrane glycoprotein 1	3	0.28218675
P13473	Lysosome-associated membrane glycoprotein 2	3	0.05539393
P61626	Lysozyme C	3	0.05493785
Q96C86	m7GpppX diphosphatase	2	0.09551558
P22897	Macrophage mannose receptor 1	13	0.46799595
P14174	Macrophage migration inhibitory factor	2	0.12795614
P40121	Macrophage-capping protein	6	0.93841651
Q14764	Major vault protein	8	0.07976396
P40925	Malate dehydrogenase, cytoplasmic	12	0.12880056
P40926	Malate dehydrogenase, mitochondrial	12	0.26305023
Q14165	Malectin	2	0.11771763
P48740	Mannan-binding lectin serine protease 1	3	0.19451303
O00187	Mannan-binding lectin serine protease 2	2	0.21371887
P11226	Mannose-binding protein C	3	0.28031431
Q3KQU3	MAP7 domain-containing protein 1	2	0.31787192
P49006	MARCKS-related protein	2	0.13287557
P15088	Mast cell carboxypeptidase A	5	0.89791911
O00339	Matrilin-2	3	0.59770187
P43243	Matrin-3	2	0.84261751
P14780	Matrix metalloproteinase-9	2	0.28497294
Q8N3F0	Maturin	2	0.74203183

Q9UNF1	Melanoma-associated antigen D2	2	0.22481286
Q16853	Membrane primary amine oxidase	6	0.12675463
O15173	Membrane-associated progesterone receptor component 2	5	0.2261849
P01033	Metalloproteinase inhibitor 1	3	0.51852105
P16035	Metalloproteinase inhibitor 2	2	0.76075179
P02795	Metallothionein-2	3	0.09694192
Q13228	Methanethiol oxidase	28	0.37364612
P51608	Methyl-CpG-binding protein 2	3	0.42906718
Q96GX9	Methylthioribulose-1-phosphate dehydratase	2	0.10923577
Q9H8H3	Methyltransferase-like protein 7A	2	0.36094737
P10620	Microsomal glutathione S-transferase 1	2	0.19638811
P46821	Microtubule-associated protein 1B	20	0.7241089
P27816	Microtubule-associated protein 4	31	0.55678677
Q15691	Microtubule-associated protein RP/EB family member 1	6	0.20153512
P10636	Microtubule-associated protein tau	6	0.37823973
P21741	Midkine	2	0.03669882
Q9BRT3	Migration and invasion enhancer 1	2	0.74815603
P20774	Mimecan	9	0.12293278
Q5VT66	Mitochondrial amidoxime-reducing component 1	2	0.98395569
Q9Y3D6	Mitochondrial fission 1 protein	2	0.27610802
Q8N4Q1	Mitochondrial intermembrane space import and assembly protein 40	2	0.28849458
P28482	Mitogen-activated protein kinase 1	7	0.08138658
O43684	Mitotic checkpoint protein BUB3	2	0.8500507
P26038	Moesin	20	0.13297805
P08571	Monocyte differentiation antigen CD14	4	0.65267332
Q99685	Monoglyceride lipase	4	0.09616549
P22234	Multifunctional protein ADE2	2	0.46205956
Q13201	Multimerin-1	5	0.33368732
O00499	Myc box-dependent-interacting protein 1	7	0.5100609
Q969H8	Myeloid-derived growth factor	3	0.171017
P05164	Myeloperoxidase	12	0.23013242
Q99972	Myocilin	2	0.38868028

Q9NZM1	Myoferlin	4	0.88844655
P02144	Myoglobin	7	0.06078465
P05976	Myosin light chain 1/3, skeletal muscle isoform	6	0.3875351
P12829	Myosin light chain 4	2	0.18095507
Q15746	Myosin light chain kinase, smooth muscle	10	0.67353393
P60660	Myosin light polypeptide 6	7	0.03723795
Q6WCQ1	Myosin phosphatase Rho-interacting protein	2	0.38843475
O14950	Myosin regulatory light chain 12B	3	0.73850329
Q96A32	Myosin regulatory light chain 2, skeletal muscle isoform	8	0.53652033
P10916	Myosin regulatory light chain 2, ventricular/cardiac muscle isoform	2	0.33357815
P24844	Myosin regulatory light polypeptide 9	4	0.21139352
P12882	Myosin-1	14	0.46398481
P35580	Myosin-10	19	0.1705077
P35749	Myosin-11	55	0.22529147
Q7Z406	Myosin-14	2	0.99096224
Q9UKX2	Myosin-2	8	0.4826894
P12883	Myosin-7	10	0.44246363
P35579	Myosin-9	59	0.18417943
P58546	Myotrophin	3	0.13304569
P29966	Myristoylated alanine-rich C-kinase substrate	6	0.86343467
Q9UJ70	N-acetyl-D-glucosamine kinase	6	0.11994421
P34059	N-acetylgalactosamine-6-sulfatase	3	0.0359965
P15586	N-acetylglucosamine-6-sulfatase	5	0.0905224
Q96PD5	N-acetylmuramoyl-L-alanine amidase	7	0.07062595
P51688	N-sulphoglucosamine sulphohydrolase	3	0.21543979
P20933	N(4)-(beta-N-acetylglucosaminyl)-L-asparaginase	2	0.07527768
O94760	N(G),N(G)-dimethylarginine dimethylaminohydrolase 1	6	0.04101404
O95865	N(G),N(G)-dimethylarginine dimethylaminohydrolase 2	4	0.2307635
O14745	Na(+)/H(+) exchange regulatory cofactor NHE-RF1	3	0.31078288
Q8NCW5	NAD(P)H-hydrate epimerase	5	0.04005311
O43920	NADH dehydrogenase [ubiquinone] iron-sulfur protein 5	2	0.63836171
O00217	NADH dehydrogenase [ubiquinone] iron-sulfur protein 8, mitochondrial	2	0.34584399

P00387	NADH-cytochrome b5 reductase 3	6	0.12319935
P48163	NADP-dependent malic enzyme	12	0.16406296
P16435	NADPH--cytochrome P450 reductase	3	0.86420668
E9PAV3	Nascent polypeptide-associated complex subunit alpha, muscle-specific form	3	0.97296511
Q15843	NEDD8	2	0.93066073
Q8WXH0	Nesprin-2	2	0.58937106
P48681	Nestin	13	0.74258847
Q9UMX5	Neudesin	3	0.75230348
O00533	Neural cell adhesion molecule L1-like protein	5	0.96182065
Q09666	Neuroblast differentiation-associated protein AHNAK	197	0.20662249
O14786	Neuropilin-1	6	0.31334801
Q9P121	Neurotrimin	5	0.48148781
Q14697	Neutral alpha-glucosidase AB	4	0.17726151
P59665	Neutrophil defensin 1	2	0.20389366
P80188	Neutrophil gelatinase-associated lipocalin	2	0.1396716
Q0ZGT2	Nexilin	3	0.91043282
P40261	Nicotinamide N-methyltransferase	2	0.96391916
P43490	Nicotinamide phosphoribosyltransferase	3	0.34560421
Q6XQN6	Nicotinate phosphoribosyltransferase	5	0.28068048
P14543	Nidogen-1	14	0.1142908
Q14112	Nidogen-2	10	0.29255506
Q9GZT8	NIF3-like protein 1	5	0.35496538
P05204	Non-histone chromosomal protein HMG-17	2	0.2725977
P10153	Non-secretory ribonuclease	2	0.30864859
P61916	NPC intracellular cholesterol transporter 2	3	0.94538633
Q9UNZ2	NSFL1 cofactor p47	7	0.1217964
P49321	Nuclear autoantigenic sperm protein	4	0.40852992
Q12857	Nuclear factor 1 A-type	2	0.17452118
Q9Y266	Nuclear migration protein nudC	2	0.70944046
Q14980	Nuclear mitotic apparatus protein 1	9	0.12124219
P35658	Nuclear pore complex protein Nup214	2	0.73046739

Q8TAT6	Nuclear protein localization protein 4 homolog	2	0.05013301
P61970	Nuclear transport factor 2	4	0.75391013
P67809	Nuclease-sensitive element-binding protein 1	2	0.61729715
Q02818	Nucleobindin-1	6	0.39045864
P80303	Nucleobindin-2	2	0.60762982
P19338	Nucleolin	13	0.29548512
P06748	Nucleophosmin	3	0.01645728
P12270	Nucleoprotein TPR	5	0.59450138
P15531	Nucleoside diphosphate kinase A	3	0.0990478
P55209	Nucleosome assembly protein 1-like 1	4	0.52432713
Q99733	Nucleosome assembly protein 1-like 4	2	0.24417399
Q9NTK5	Obg-like ATPase 1	5	0.12956871
Q9NRN5	Olfactomedin-like protein 3	3	0.79609918
Q9NQR4	Omega-amidase NIT2	4	0.18730164
Q96CV9	Optineurin	2	0.17249438
Q8WX93	Palladin	2	0.54724378
Q9NP74	Palmdelphin	3	0.33720209
O95497	Pantetheinase	5	0.56731116
O75781	Paralemmin-1	9	0.42604808
Q8IXS6	Paralemmin-2	4	0.85179084
P20962	Parathymosin	2	0.08986519
O00151	PDZ and LIM domain protein 1	8	0.03230222
Q96HC4	PDZ and LIM domain protein 5	2	0.42731599
P26022	Pentraxin-related protein PTX3	2	0.44688665
Q6UXB8	Peptidase inhibitor 16	3	0.59816079
O75594	Peptidoglycan recognition protein 1	2	#DIV/0!
P62937	Peptidyl-prolyl cis-trans isomerase A	12	0.26114312
P23284	Peptidyl-prolyl cis-trans isomerase B	10	0.11302098
Q9UNP9	Peptidyl-prolyl cis-trans isomerase E	2	0.12562389
P30405	Peptidyl-prolyl cis-trans isomerase F, mitochondrial	2	0.34786407
P26885	Peptidyl-prolyl cis-trans isomerase FKBP2	2	0.6280937
Q00688	Peptidyl-prolyl cis-trans isomerase FKBP3	2	0.18449103

Q02790	Peptidyl-prolyl cis-trans isomerase FKBP4	6	0.74051048
Q9H2H8	Peptidyl-prolyl cis-trans isomerase-like 3	2	0.98642615
O60240	Perilipin-1	20	0.12303399
O60664	Perilipin-3	6	0.68199876
Q96Q06	Perilipin-4	52	0.13388606
Q15063	Periostin	15	0.06378522
O60437	Periplakin	9	0.23971711
Q06830	Peroxiredoxin-1	9	0.09178498
P32119	Peroxiredoxin-2	7	0.11407935
P30044	Peroxiredoxin-5, mitochondrial	6	0.10891466
P30041	Peroxiredoxin-6	12	0.05774743
Q9BRX8	Peroxiredoxin-like 2A	5	0.05828673
P40855	Peroxisomal biogenesis factor 19	7	0.94504576
P51659	Peroxisomal multifunctional enzyme type 2	2	0.38104582
P04180	Phosphatidylcholine-sterol acyltransferase	2	0.15920037
P30086	Phosphatidylethanolamine-binding protein 1	9	0.06694955
P48426	Phosphatidylinositol 5-phosphate 4-kinase type-2 alpha	3	0.12153864
Q00169	Phosphatidylinositol transfer protein alpha isoform	2	0.10009947
P80108	Phosphatidylinositol-glycan-specific phospholipase D	2	0.63620482
O95394	Phosphoacetylglucosamine mutase	2	0.49531725
P36871	Phosphoglucomutase-1	22	0.72384835
Q96G03	Phosphoglucomutase-2	7	0.09973637
Q15124	Phosphoglucomutase-like protein 5	11	0.78848056
P00558	Phosphoglycerate kinase 1	22	0.06680021
P18669	Phosphoglycerate mutase 1	9	0.73971303
P36969	Phospholipid hydroperoxide glutathione peroxidase	2	0.05329249
P55058	Phospholipid transfer protein	2	0.21554743
Q9H008	Phospholysine phosphohistidine inorganic pyrophosphate phosphatase	7	0.17654194
O60256	Phosphoribosyl pyrophosphate synthase-associated protein 2	2	0.37136225
O15067	Phosphoribosylformylglycinamidine synthase	2	0.48211462
Q9Y617	Phosphoserine aminotransferase	4	0.29142744
Q96BW5	Phosphotriesterase-related protein	2	0.74086746

P36955	Pigment epithelium-derived factor	14	0.27471349
Q9GZP4	PITH domain-containing protein 1	4	0.59280689
Q9BTY2	Plasma alpha-L-fucosidase	3	0.04611034
P03952	Plasma kallikrein	17	0.19255067
P20020	Plasma membrane calcium-transporting ATPase 1	2	0.61443496
P23634	Plasma membrane calcium-transporting ATPase 4	4	0.3536271
P05155	Plasma protease C1 inhibitor	11	0.33174037
P00747	Plasminogen	30	0.30743224
P05121	Plasminogen activator inhibitor 1	3	0.56489397
Q8NC51	Plasminogen activator inhibitor 1 RNA-binding protein	5	0.5093942
P13796	Plastin-2	21	0.10701012
P13797	Plastin-3	6	0.62058894
P02775	Platelet basic protein	2	0.44261029
P16671	Platelet glycoprotein 4	6	0.02325684
P07359	Platelet glycoprotein Ib alpha chain	3	0.24856812
P43034	Platelet-activating factor acetylhydrolase IB subunit alpha	11	0.04546675
P08567	Pleckstrin	2	0.32108737
Q8TD55	Pleckstrin homology domain-containing family O member 2	2	0.12713544
Q86UU1	Pleckstrin homology-like domain family B member 1	3	0.19744545
Q15149	Plectin	65	0.81499811
Q6UX71	Plexin domain-containing protein 2	4	0.79657226
P09874	Poly [ADP-ribose] polymerase 1	2	0.49893458
P11940	Polyadenylate-binding protein 1	2	0.45534668
Q86U42	Polyadenylate-binding protein 2	2	0.16671401
Q9H074	Polyadenylate-binding protein-interacting protein 1	2	0.21773016
P01833	Polymeric immunoglobulin receptor	4	0.00577846
P26599	Polypyrimidine tract-binding protein 1	2	0.21733831
P08397	Porphobilinogen deaminase	4	0.17181656
O75915	PRA1 family protein 3	4	0.03641419
Q9NQP4	Prefoldin subunit 4	2	0.14690457
P20742	Pregnancy zone protein	6	0.39961631
P02545	Prelamin-A/C	31	0.53604836

Q9UHG3	Prenylcysteine oxidase 1	5	0.0593301
P09668	Pro-cathepsin H	3	0.20551629
Q8NDH3	Probable aminopeptidase NPEPL1	3	0.21750922
Q92841	Probable ATP-dependent RNA helicase DDX17	3	0.54419785
O76071	Probable cytosolic iron-sulfur protein assembly protein CIAO1	2	0.22811543
Q15113	Procollagen C-endopeptidase enhancer 1	6	0.4850856
P07737	Profilin-1	8	0.18383921
Q8WUM4	Programmed cell death 6-interacting protein	9	0.01190921
O14737	Programmed cell death protein 5	3	0.25911024
P28799	Progranulin	8	0.20080278
P35232	Prohibitin	2	0.00381313
P12273	Prolactin-inducible protein	4	0.20541099
P51888	Prolargin	5	0.00499292
Q9UQ80	Proliferation-associated protein 2G4	7	0.19644945
Q07954	Prolow-density lipoprotein receptor-related protein 1	18	0.55682991
P48147	Prolyl endopeptidase	7	0.05383821
P27918	Properdin	3	0.60981791
P07602	Prosaposin	5	0.05524684
Q16647	Prostacyclin synthase	2	0.22244353
Q15185	Prostaglandin E synthase 3	2	0.05771488
Q14914	Prostaglandin reductase 1	5	0.27245968
P41222	Prostaglandin-H2 D-isomerase	2	0.18059212
Q06323	Proteasome activator complex subunit 1	6	0.17775935
Q9UL46	Proteasome activator complex subunit 2	3	0.35895521
P25786	Proteasome subunit alpha type-1	10	0.25977095
P25787	Proteasome subunit alpha type-2	10	0.12622412
P25788	Proteasome subunit alpha type-3	7	0.14302813
P25789	Proteasome subunit alpha type-4	10	0.14129164
P28066	Proteasome subunit alpha type-5	9	0.11872831
P60900	Proteasome subunit alpha type-6	12	0.04915609
O14818	Proteasome subunit alpha type-7	9	0.16364288
P20618	Proteasome subunit beta type-1	4	0.20199467

P40306	Proteasome subunit beta type-10	3	0.02053527
P49721	Proteasome subunit beta type-2	6	0.1647112
P49720	Proteasome subunit beta type-3	5	0.21936644
P28070	Proteasome subunit beta type-4	6	0.06444347
P28074	Proteasome subunit beta type-5	5	0.22364536
P28072	Proteasome subunit beta type-6	6	0.41409681
Q99436	Proteasome subunit beta type-7	7	0.11822894
P28062	Proteasome subunit beta type-8	3	0.06677831
P28065	Proteasome subunit beta type-9	3	0.01314858
P11171	Protein 4.1	19	0.04741269
Q96IU4	Protein ABHD14B	3	0.11233605
Q8IVF2	Protein AHNAK2	2	0.79999938
P02760	Protein AMBP	14	0.98583931
O14744	Protein arginine N-methyltransferase 5	2	0.35727048
Q9Y2B0	Protein canopy homolog 2	4	0.44312867
Q9UKY7	Protein CDV3 homolog	4	0.21851939
Q5TDH0	Protein DDI1 homolog 2	3	0.1136282
P07237	Protein disulfide-isomerase	17	0.334147
P30101	Protein disulfide-isomerase A3	21	0.33236982
P13667	Protein disulfide-isomerase A4	11	0.97774331
Q15084	Protein disulfide-isomerase A6	6	0.16884205
Q9NUQ9	Protein FAM49B	4	0.05631958
P49354	Protein farnesyltransferase/geranylgeranyltransferase type-1 subunit alpha	3	0.17450715
Q8WZA0	Protein LZIC	2	0.28981466
Q9BZQ8	Protein Niban	2	0.92177061
Q8IWE2	Protein NOXP20	2	0.61823272
O14974	Protein phosphatase 1 regulatory subunit 12A	3	0.29747031
Q15435	Protein phosphatase 1 regulatory subunit 7	3	0.16213582
P35813	Protein phosphatase 1A	3	0.10494592
O75688	Protein phosphatase 1B	3	0.20490342
P49593	Protein phosphatase 1F	3	0.12009266

Q9Y570	Protein phosphatase methylesterase 1	3	0.3293915
P29590	Protein PML	2	0.61829531
Q9P258	Protein RCC2	2	0.03876676
P60903	Protein S100-A10	2	0.08649384
P31949	Protein S100-A11	3	0.91473777
Q99584	Protein S100-A13	4	0.16063572
P26447	Protein S100-A4	3	0.03238403
P31151	Protein S100-A7	4	0.07561823
P05109	Protein S100-A8	8	0.10072429
P06702	Protein S100-A9	7	0.08327275
P04271	Protein S100-B	3	0.81124107
P25815	Protein S100-P	2	0.11254249
Q01105	Protein SET	3	0.19090234
Q92734	Protein TFG	2	0.59321392
O94979	Protein transport protein Sec31A	2	0.38967917
Q9H993	Protein-glutamate O-methyltransferase	3	0.82986775
P21980	Protein-glutamine gamma-glutamyltransferase 2	11	0.02090715
P22061	Protein-L-isoaspartate(D-aspartate) O-methyltransferase	6	0.07898386
Q99497	Protein/nucleic acid deglycase DJ-1	10	0.27851019
Q92954	Proteoglycan 4	10	0.54256674
P00734	Prothrombin	23	0.03980759
P06454	Prothymosin alpha	2	0.0054458
Q08174	Protocadherin-1	2	0.81623712
P00491	Purine nucleoside phosphorylase	14	0.84804472
P55786	Puromycin-sensitive aminopeptidase	25	0.0475889
Q6P1N9	Putative deoxyribonuclease TATDN1	2	0.06237297
Q5T013	Putative hydroxypyruvate isomerase	6	0.22192042
A6NI72	Putative neutrophil cytosol factor 1B	3	0.39307677
Q9Y383	Putative RNA-binding protein Luc7-like 2	3	0.62047143
O00764	Pyridoxal kinase	8	0.18489881
O94903	Pyridoxal phosphate homeostasis protein	2	0.00382702
Q6P996	Pyridoxal-dependent decarboxylase domain-containing protein 1	3	0.46311555

P08559	Pyruvate dehydrogenase E1 component subunit alpha, somatic form, mitochondrial	2	0.72943855
P11177	Pyruvate dehydrogenase E1 component subunit beta, mitochondrial	2	0.70808502
P30613	Pyruvate kinase PKLR	7	0.31777725
P14618	Pyruvate kinase PKM	27	0.07475511
Q08257	Quinone oxidoreductase	7	0.05961803
Q53FA7	Quinone oxidoreductase PIG3	6	0.10526423
P31150	Rab GDP dissociation inhibitor alpha	14	0.05657981
P50395	Rab GDP dissociation inhibitor beta	15	0.36318477
Q15276	Rab GTPase-binding effector protein 1	3	0.95711088
P35241	Radixin	9	0.01504813
P43487	Ran-specific GTPase-activating protein	4	0.38544057
P46940	Ras GTPase-activating-like protein IQGAP1	22	0.32124567
Q13576	Ras GTPase-activating-like protein IQGAP2	4	0.72577163
Q15404	Ras suppressor protein 1	4	0.10204444
P15153	Ras-related C3 botulinum toxin substrate 2	2	0.12663313
O14807	Ras-related protein M-Ras	2	0.24708704
P10301	Ras-related protein R-Ras	2	0.10469847
P61026	Ras-related protein Rab-10	2	0.22977894
Q15907	Ras-related protein Rab-11B	4	0.37325886
Q9H0U4	Ras-related protein Rab-1B	2	0.05632666
Q9UL25	Ras-related protein Rab-21	3	0.36470754
P61019	Ras-related protein Rab-2A	2	0.26371252
P51149	Ras-related protein Rab-7a	5	0.1170139
P11233	Ras-related protein Ral-A	3	0.31449286
P61224	Ras-related protein Rap-1b	3	0.11012638
P61225	Ras-related protein Rap-2b	4	0.12854164
Q00765	Receptor expression-enhancing protein 5	2	0.7614447
P63244	Receptor of activated protein C kinase 1	8	0.01469873
P08575	Receptor-type tyrosine-protein phosphatase C	2	0.21661531
Q15493	Regucalcin	3	0.06665261
Q96DB5	Regulator of microtubule dynamics protein 1	2	0.05661918

P27694	Replication protein A 70 kDa DNA-binding subunit	2	0.3719351
Q15293	Reticulocalbin-1	10	0.81111574
Q14257	Reticulocalbin-2	6	0.10811846
Q96D15	Reticulocalbin-3	6	0.28375962
Q9NQC3	Reticulon-4	5	0.1444315
Q8NC44	Reticulophagy regulator 2	2	0.87708007
Q86VR2	Reticulophagy regulator 3	2	0.5456429
P00352	Retinal dehydrogenase 1	18	0.14125919
P02753	Retinol-binding protein 4	9	0.48265974
P52565	Rho GDP-dissociation inhibitor 1	5	0.24650909
P52566	Rho GDP-dissociation inhibitor 2	5	0.42463849
Q07960	Rho GTPase-activating protein 1	4	0.11909379
Q68EM7	Rho GTPase-activating protein 17	2	0.28166359
Q8N392	Rho GTPase-activating protein 18	2	0.37393263
Q7Z6I6	Rho GTPase-activating protein 30	2	0.33845718
P34096	Ribonuclease 4	2	0.03945503
P13489	Ribonuclease inhibitor	14	0.28248997
P07998	Ribonuclease pancreatic	4	0.29623341
O00584	Ribonuclease T2	3	0.15496981
P49247	Ribose-5-phosphate isomerase	6	0.66784573
P60891	Ribose-phosphate pyrophosphokinase 1	3	0.15242383
Q9Y3A5	Ribosome maturation protein SBDS	2	#DIV/0!
Q9P2E9	Ribosome-binding protein 1	17	0.46853538
P16083	Ribosyldihydronicotinamide dehydrogenase [quinone]	6	0.03837373
P38159	RNA-binding motif protein, X chromosome	3	0.11991525
Q01844	RNA-binding protein EWS	2	0.60064451
P35637	RNA-binding protein FUS	2	0.07985728
Q9UKM9	RNA-binding protein Raly	2	0.41615088
Q9Y265	RuvB-like 1	2	0.95440847
Q9Y230	RuvB-like 2	3	0.13768352
P10768	S-formylglutathione hydrolase	7	0.44043513
Q13126	S-methyl-5'-thioadenosine phosphorylase	4	0.2034799

P63208	S-phase kinase-associated protein 1	5	0.16575737
O75995	SAM and SH3 domain-containing protein 3	2	0.35810981
Q93084	Sarcoplasmic/endoplasmic reticulum calcium ATPase 3	2	0.17448304
Q15424	Scaffold attachment factor B1	5	0.04515264
Q86VB7	Scavenger receptor cysteine-rich type 1 protein M130	17	0.00744616
Q12765	Secernin-1	6	0.03066824
Q96FV2	Secernin-2	3	0.74964327
Q92765	Secreted frizzled-related protein 3	2	0.86570296
Q6FHJ7	Secreted frizzled-related protein 4	4	0.27521387
O95969	Secretoglobin family 1D member 2	3	0.10253919
P49908	Selenoprotein P	3	0.60100917
Q9NVA2	Septin-11	4	0.236072
Q15019	Septin-2	10	0.07882896
Q16181	Septin-7	4	0.07325836
Q9UHD8	Septin-9	4	0.26897931
P10124	Serglycin	2	0.28554719
P34896	Serine hydroxymethyltransferase, cytosolic	3	0.09005202
P49591	Serine--tRNA ligase, cytoplasmic	2	0.10791551
Q9Y3F4	Serine-threonine kinase receptor-associated protein	3	0.69180507
Q07955	Serine/arginine-rich splicing factor 1	3	0.24617823
O15075	Serine/threonine-protein kinase DCLK1	3	0.76541387
O95747	Serine/threonine-protein kinase OSR1	5	0.03854519
Q13177	Serine/threonine-protein kinase PAK 2	3	0.39783523
Q9H4A3	Serine/threonine-protein kinase WNK1	5	0.02889415
P63151	Serine/threonine-protein phosphatase 2A 55 kDa regulatory subunit B alpha isoform	2	0.32311677
P30153	Serine/threonine-protein phosphatase 2A 65 kDa regulatory subunit A alpha isoform	8	0.14280366
Q15257	Serine/threonine-protein phosphatase 2A activator	7	0.21968041
Q9BRF8	Serine/threonine-protein phosphatase CPPED1	7	0.17435236
P62140	Serine/threonine-protein phosphatase PP1-beta catalytic subunit	6	0.8213346
P02787	Serotransferrin	62	0.19882049

P29508	Serpin B3	2	0.33538025
P35237	Serpin B6	5	0.58543811
P50453	Serpin B9	5	0.25354997
P50454	Serpin H1	7	0.76302107
P02768	Serum albumin	91	0.62485065
P02743	Serum amyloid P-component	7	0.06656563
P27169	Serum paraoxonase/arylesterase 1	8	0.25523387
P04278	Sex hormone-binding globulin	3	0.21967989
O75368	SH3 domain-binding glutamic acid-rich-like protein	9	0.0425744
Q9H299	SH3 domain-binding glutamic acid-rich-like protein 3	4	0.24067841
Q9NR45	Sialic acid synthase	2	0.49775285
P42224	Signal transducer and activator of transcription 1-alpha/beta	2	0.46733962
Q04837	Single-stranded DNA-binding protein, mitochondrial	2	0.1616514
O43765	Small glutamine-rich tetratricopeptide repeat-containing protein alpha	2	0.32893175
P22532	Small proline-rich protein 2D	2	0.24424383
P53814	Smoothelin	2	0.33410007
A1L4H1	Soluble scavenger receptor cysteine-rich domain-containing protein SSC5D	6	0.04098175
P11166	Solute carrier family 2, facilitated glucose transporter member 1	6	0.30401439
Q9BX66	Sorbin and SH3 domain-containing protein 1	17	0.37431471
O94875	Sorbin and SH3 domain-containing protein 2	2	0.27644521
Q00796	Sorbitol dehydrogenase	2	0.38948266
P30626	Sorcin	7	0.04427742
Q13596	Sorting nexin-1	2	0.38513474
O60749	Sorting nexin-2	6	0.13492251
Q9UNH7	Sorting nexin-6	2	0.03820229
P09486	SPARC	8	0.81328733
Q14515	SPARC-like protein 1	13	0.36049608
P02549	Spectrin alpha chain, erythrocytic 1	100	0.05609143
Q13813	Spectrin alpha chain, non-erythrocytic 1	123	0.39061154
P11277	Spectrin beta chain, erythrocytic	94	0.07274633
Q01082	Spectrin beta chain, non-erythrocytic 1	69	0.12154295

Q13838	Spliceosome RNA helicase DDX39B	3	0.77500536
Q15637	Splicing factor 1	2	0.47055255
Q15459	Splicing factor 3A subunit 1	3	0.20421119
Q15427	Splicing factor 3B subunit 4	2	0.38738195
P26368	Splicing factor U2AF 65 kDa subunit	2	0.68466164
P23246	Splicing factor, proline- and glutamine-rich	3	0.45518729
Q9HCB6	Spondin-1	7	0.59166551
Q14247	Src substrate cortactin	4	0.67542458
Q7KZF4	Staphylococcal nuclease domain-containing protein 1	9	0.21988482
P16949	Stathmin	2	0.13019581
P38646	Stress-70 protein, mitochondrial	7	0.105398
P31948	Stress-induced-phosphoprotein 1	10	0.15485995
P31040	Succinate dehydrogenase [ubiquinone] flavoprotein subunit, mitochondrial	2	0.23250109
Q96I99	Succinate--CoA ligase [GDP-forming] subunit beta, mitochondrial	6	0.81635846
P55809	Succinyl-CoA:3-ketoacid coenzyme A transferase 1, mitochondrial	6	0.4975193
P0DMN0	Sulfotransferase 1A4	2	0.27328495
Q9UBE0	SUMO-activating enzyme subunit 1	2	0.26486132
P00441	Superoxide dismutase [Cu-Zn]	7	0.33452173
P04179	Superoxide dismutase [Mn], mitochondrial	5	0.18148201
Q6UWP8	Suprabasin	2	0.35955574
Q8TAQ2	SWI/SNF complex subunit SMARCC2	3	0.84453775
Q96A49	Synapse-associated protein 1	2	0.12706616
Q99536	Synaptic vesicle membrane protein VAT-1 homolog	4	0.42509771
O15498	Synaptobrevin homolog YKT6	3	0.11012367
Q9UMS6	Synaptopodin-2	4	0.42414064
O15061	Synemin	12	0.56078295
Q86Y82	Syntaxin-12	3	0.49339196
O15400	Syntaxin-7	4	0.17143693
P61764	Syntaxin-binding protein 1	3	0.53398038
P17987	T-complex protein 1 subunit alpha	11	0.03359946
P78371	T-complex protein 1 subunit beta	9	0.14203785

P50991	T-complex protein 1 subunit delta	11	0.07382312
P48643	T-complex protein 1 subunit epsilon	9	0.01261203
Q99832	T-complex protein 1 subunit eta	9	0.185245
P49368	T-complex protein 1 subunit gamma	9	0.0818366
P50990	T-complex protein 1 subunit theta	11	0.02629076
P40227	T-complex protein 1 subunit zeta	5	0.04788942
Q9Y490	Talin-1	64	0.07952159
Q9Y4G6	Talin-2	37	0.24043154
O60784	Target of Myb protein 1	3	0.33804796
Q7Z7G0	Target of Nesh-SH3	11	0.66826037
Q92804	TATA-binding protein-associated factor 2N	3	0.21885816
P24821	Tenascin	16	0.30695938
P22105	Tenascin-X	74	0.71446599
Q9HBL0	Tensin-1	18	0.27126426
Q9UGI8	Testin	3	0.99174867
P05452	Tetranectin	12	0.66241369
Q9H3S4	Thiamin pyrophosphokinase 1	2	0.1874669
P52888	Thimet oligopeptidase	7	0.16376556
P10599	Thioredoxin	6	0.31059305
Q9BRA2	Thioredoxin domain-containing protein 17	3	0.04896727
Q8NBS9	Thioredoxin domain-containing protein 5	5	0.30683021
Q16881	Thioredoxin reductase 1, cytoplasmic	6	0.13202271
P30048	Thioredoxin-dependent peroxide reductase, mitochondrial	5	0.08286363
O43396	Thioredoxin-like protein 1	3	0.32923032
Q16762	Thiosulfate sulfurtransferase	2	0.18239468
P07996	Thrombospondin-1	29	0.82225725
P35442	Thrombospondin-2	4	0.39093238
P35443	Thrombospondin-4	5	0.55649974
P19971	Thymidine phosphorylase	7	0.48240643
P63313	Thymosin beta-10	2	0.62325688
P62328	Thymosin beta-4	5	0.45538924
Q9Y2W1	Thyroid hormone receptor-associated protein 3	2	0.21301883

P05543	Thyroxine-binding globulin	14	0.0678574
Q07157	Tight junction protein ZO-1	2	0.88541583
Q9UDY2	Tight junction protein ZO-2	2	0.49028744
Q12888	TP53-binding protein 1	2	0.26291371
P37837	Transaldolase	12	0.12137213
Q969E4	Transcription elongation factor A protein-like 3	2	0.23109736
P20290	Transcription factor BTF3	2	0.39994255
Q96K17	Transcription factor BTF3 homolog 4	2	0.48995022
Q13263	Transcription intermediary factor 1-beta	5	0.11133222
Q00577	Transcriptional activator protein Pur-alpha	6	0.07725985
P02786	Transferrin receptor protein 1	6	0.07715409
Q15582	Transforming growth factor-beta-induced protein ig-h3	12	0.05714549
P61586	Transforming protein RhoA	5	0.05762
Q01995	Transgelin	13	0.49571135
P37802	Transgelin-2	9	0.2785638
P55072	Transitional endoplasmic reticulum ATPase	30	0.08001711
P29401	Transketolase	28	0.03130572
P13693	Translationally-controlled tumor protein	3	0.82963643
Q15631	Translin	3	0.11966196
Q15363	Transmembrane emp24 domain-containing protein 2	2	0.4302924
Q5JRA6	Transport and Golgi organization protein 1 homolog	2	0.82416267
P02766	Transthyretin	7	0.12528275
P04155	Trefoil factor 1	2	0.52992004
P40939	Trifunctional enzyme subunit alpha, mitochondrial	5	0.53350636
P55084	Trifunctional enzyme subunit beta, mitochondrial	5	0.02291424
P60174	Triosephosphate isomerase	18	0.18861532
P29144	Tripeptidyl-peptidase 2	3	0.61950047
P28289	Tropomodulin-1	10	0.02873719
Q9NZR1	Tropomodulin-2	3	0.0876617
Q9NYL9	Tropomodulin-3	4	0.46294424
P09493	Tropomyosin alpha-1 chain	14	0.55089873
P06753	Tropomyosin alpha-3 chain	7	0.68581556

P67936	Tropomyosin alpha-4 chain	7	0.57759753
P07951	Tropomyosin beta chain	15	0.91530717
P02585	Troponin C, skeletal muscle	4	0.59939452
P07477	Trypsin-1	2	0.33697426
Q15661	Tryptase alpha/beta-1	6	0.25508079
P23381	Tryptophan--tRNA ligase, cytoplasmic	5	0.10879856
P68363	Tubulin alpha-1B chain	4	0.16769042
P68366	Tubulin alpha-4A chain	2	0.09987031
P07437	Tubulin beta chain	3	0.29780853
Q13885	Tubulin beta-2A chain	3	0.14329128
P04350	Tubulin beta-4A chain	4	0.11057782
Q9BUF5	Tubulin beta-6 chain	4	0.18818541
Q9BW30	Tubulin polymerization-promoting protein family member 3	4	0.73322854
O75347	Tubulin-specific chaperone A	5	0.47052053
O43399	Tumor protein D54	9	0.80597622
Q6IBS0	Twinfilin-2	3	0.19844771
P07948	Tyrosine-protein kinase Lyn	2	0.31750941
P18031	Tyrosine-protein phosphatase non-receptor type 1	2	0.37481987
Q06124	Tyrosine-protein phosphatase non-receptor type 11	3	0.17450493
P29350	Tyrosine-protein phosphatase non-receptor type 6	3	0.08974073
P78324	Tyrosine-protein phosphatase non-receptor type substrate 1	4	0.29421626
O15042	U2 snRNP-associated SURP motif-containing protein	2	0.81414918
O75643	U5 small nuclear ribonucleoprotein 200 kDa helicase	2	0.06332146
O95777	U6 snRNA-associated Sm-like protein LSm8	3	0.54259462
Q9UMX0	Ubiquilin-1	4	0.85097746
O75208	Ubiquinone biosynthesis protein COQ9, mitochondrial	2	0.05907507
P54578	Ubiquitin carboxyl-terminal hydrolase 14	13	0.22029179
Q9Y4E8	Ubiquitin carboxyl-terminal hydrolase 15	5	0.09255589
P45974	Ubiquitin carboxyl-terminal hydrolase 5	8	0.1323372
P09936	Ubiquitin carboxyl-terminal hydrolase isozyme L1	4	0.47184625
P15374	Ubiquitin carboxyl-terminal hydrolase isozyme L3	3	0.58600287
O14562	Ubiquitin domain-containing protein UBFD1	2	0.62672668

Q92890	Ubiquitin recognition factor in ER-associated degradation protein 1	2	0.48021758
Q5VVQ6	Ubiquitin thioesterase OTU1	3	0.37853374
Q96FW1	Ubiquitin thioesterase OTUB1	5	0.2275813
P62979	Ubiquitin-40S ribosomal protein S27a	5	0.21711098
Q9BSL1	Ubiquitin-associated domain-containing protein 1	5	0.13404222
Q14157	Ubiquitin-associated protein 2-like	3	0.8296209
P68036	Ubiquitin-conjugating enzyme E2 L3	2	0.01228183
P61088	Ubiquitin-conjugating enzyme E2 N	5	0.05109937
Q13404	Ubiquitin-conjugating enzyme E2 variant 1	2	0.04829314
P22314	Ubiquitin-like modifier-activating enzyme 1	21	0.2184605
P30085	UMP-CMP kinase	2	0.48161759
Q8IXQ3	Uncharacterized protein C9orf40	3	0.26919833
O00159	Unconventional myosin-Ic	13	0.03160533
Q9UM54	Unconventional myosin-VI	3	0.2793231
P06132	Uroporphyrinogen decarboxylase	4	0.16105749
Q16851	UTP--glucose-1-phosphate uridylyltransferase	11	0.09973565
P46939	Utrophin	17	0.53103623
P54725	UV excision repair protein RAD23 homolog A	8	0.11772102
P54727	UV excision repair protein RAD23 homolog B	6	0.49331629
Q9BZF9	Uveal autoantigen with coiled-coil domains and ankyrin repeats	3	0.40161268
Q9Y279	V-set and immunoglobulin domain-containing protein 4	2	0.03355882
P38606	V-type proton ATPase catalytic subunit A	3	0.02432698
P21281	V-type proton ATPase subunit B, brain isoform	3	0.36234598
O75436	Vacuolar protein sorting-associated protein 26A	2	0.18873291
Q9UK41	Vacuolar protein sorting-associated protein 28 homolog	2	0.57386187
Q9UBQ0	Vacuolar protein sorting-associated protein 29	3	0.38284928
Q96QK1	Vacuolar protein sorting-associated protein 35	9	0.14434593
P19320	Vascular cell adhesion protein 1	8	0.8559675
P13611	Versican core protein	18	0.80879596
P49748	Very long-chain specific acyl-CoA dehydrogenase, mitochondrial	3	0.51574484
Q9P0L0	Vesicle-associated membrane protein-associated protein A	3	0.24827693
O95292	Vesicle-associated membrane protein-associated protein B/C	3	0.2753028

P46459	Vesicle-fusing ATPase	2	0.06301827
O75396	Vesicle-trafficking protein SEC22b	5	0.09010193
Q00341	Vigilin	2	0.27169588
P08670	Vimentin	40	0.07899468
P18206	Vinculin	51	0.10780982
O60504	Vinexin	2	0.99699651
P02774	Vitamin D-binding protein	26	0.66451107
P04070	Vitamin K-dependent protein C	2	0.24494137
P07225	Vitamin K-dependent protein S	9	0.20540816
Q6UXI7	Vitrin	2	0.96536547
P04004	Vitronectin	9	0.48941897
P45880	Voltage-dependent anion-selective channel protein 2	2	0.25794107
P54289	Voltage-dependent calcium channel subunit alpha-2/delta-1	2	0.51712693
P04275	von Willebrand factor	40	0.15285339
Q641Q2	WASH complex subunit 2A	2	0.56041119
O75083	WD repeat-containing protein 1	14	0.09706254
Q5JSH3	WD repeat-containing protein 44	4	0.98210413
P61964	WD repeat-containing protein 5	3	0.19755226
O76076	WNT1-inducible-signaling pathway protein 2	3	0.48298601
O76024	Wolframin	2	0.81679648
P13010	X-ray repair cross-complementing protein 5	7	0.33848708
P12956	X-ray repair cross-complementing protein 6	10	0.21463172
P12955	Xaa-Pro dipeptidase	12	0.15409291
Q6NZY4	Zinc finger CCHC domain-containing protein 8	2	0.66265152
P25311	Zinc-alpha-2-glycoprotein	19	0.04637609
Q15942	Zyxin	3	0.42510716

TGM2|TGM2_pS216

	Difference in means	Adjusted p-value
CC-SLNB	0.8735895	0.4124635
ALND-SLNB	0.5722404	0.5568776
ALND-CC	-0.3013491	0.8932914

TGM2|TGM2_pS215

	Difference in means	Adjusted p-value
CC-SLNB	0.4227602	0.6104227
ALND-SLNB	-0.2642242	0.7856061
ALND-CC	-0.6869844	0.1213764

TGM2|TGM2

	Difference in means	Adjusted p-value
CC-SLNB	-0.164202791	0.6729985
ALND-SLNB	-0.1688838	0.6579018
ALND-CC	-0.0046819	0.999563

AK1|AK1

	Difference in means	Adjusted p-value
CC-SLNB	-0.1179162	0.6917456
ALND-SLNB	-0.15604867	0.5265008
ALND-CC	-0.03813247	0.9490896

AK1|AK1_pT35

	Difference in means	Adjusted p-value
CC-SLNB	-0.1516954	0.7957695
ALND-SLNB	-0.2643183	0.5161931
ALND-CC	-0.1126229	0.8359649

AK1|AK1_pS178

	Difference in means	Adjusted p-value
CC-SLNB	-0.1014045	0.9137691
ALND-SLNB	-0.2927613	0.476753
ALND-CC	-0.1913569	0.6511566

UBE2L3|UBE2L3

	Difference in means	Adjusted p-value
CC-SLNB	-0.04259368	0.8945594
ALND-SLNB	0.06195627	0.790492
ALND-CC	0.10454995	0.4114276

ILVBL|ILVBL

	Difference in means	Adjusted p-value
CC-SLNB	0.09163741	0.6343111
ALND-SLNB	0.12506218	0.431461
ALND-CC	0.03342476	0.921076

ASPN|ASPN

	Difference in means	Adjusted p-value
CC-SLNB	-0.63839967	0.04692927
ALND-SLNB	-0.72313834	0.02113124
ALND-CC	-0.08473867	0.92540206

PRELP|PRELP

	Difference in means	Adjusted p-value
CC-SLNB	-0.5307257	0.1048258
ALND-SLNB	-0.7215841	0.01810742
ALND-CC	-0.1908584	0.66422564

PRELP|PRELP_pS322

	Difference in means	Adjusted p-value
CC-SLNB	0.5080779	0.38209443
ALND-SLNB	-0.2698988	0.74785833
ALND-CC	-0.7779767	0.06984579

APOC3|APOC3

	Difference in means	Adjusted p-value
CC-SLNB	-0.5875333	0.07118151
ALND-SLNB	-0.2199724	0.67743159
ALND-CC	0.3675609	0.23928204

ADD1|ADD1

	Difference in means	Adjusted p-value
CC-SLNB	-0.3454805	0.007305318
ALND-SLNB	-0.2322833	0.095138094
ALND-CC	0.1131972	0.458947953

ADD1|ADD1_pS12

	Difference in means	Adjusted p-value
CC-SLNB	0.02662804	0.9970945
ALND-SLNB	-0.21171193	0.838801
ALND-CC	-0.23833997	0.7219756

ADD1|ADD1_pS427

	Difference in means	Adjusted p-value
CC-SLNB	-0.2635957	0.3813017
ALND-SLNB	-0.4110219	0.1041658
ALND-CC	-0.1474262	0.6770519

ADD1|ADD1_pS586

	Difference in means	Adjusted p-value
CC-SLNB	0.3564338	0.761241934
ALND-SLNB	-1.2794166	0.038162976
ALND-CC	-1.6358504	0.001016098

ADD1|ADD1_pS59

	Difference in means	Adjusted p-value
CC-SLNB	-0.03864581	0.9927857
ALND-SLNB	-0.41786335	0.390775
ALND-CC	-0.37921753	0.3963338

ADD1|ADD1_pS532

	Difference in means	Adjusted p-value
CC-SLNB	0.2998181	0.7631849
ALND-SLNB	-0.1301573	0.9434896
ALND-CC	-0.4299754	0.3856364

ADD1|ADD1_pT11

	Difference in means	Adjusted p-value
CC-SLNB	-0.5337515	0.4462567
ALND-SLNB	-0.2855511	0.7473316
ALND-CC	0.2482004	0.8013046

ADD1|ADD1_pS358

	Difference in means	Adjusted p-value
CC-SLNB	0.0277981	0.9939857
ALND-SLNB	-0.1366103	0.862448
ALND-CC	-0.1644084	0.7613658

ADD1|ADD1_pS355

	Difference in means	Adjusted p-value
CC-SLNB	-0.3618394	0.5153753
ALND-SLNB	-0.6248248	0.1967113
ALND-CC	-0.2629853	0.5896391

ADD1|ADD1_pT429

	Difference in means	Adjusted p-value
CC-SLNB	-0.3634286	0.096575558
ALND-SLNB	-0.5280319	0.008584841
ALND-CC	-0.1646033	0.513610197

ADD1|ADD1_pT724

	Difference in means	Adjusted p-value
CC-SLNB	-0.60384427	0.04262471
ALND-SLNB	-0.609477556	0.04504656
ALND-CC	-0.005633285	0.99961297

ADD1|ADD1_pS726

	Difference in means	Adjusted p-value
CC-SLNB	-0.5549194	0.5698735
ALND-SLNB	-0.9524175	0.2199906
ALND-CC	-0.3974981	0.6201382

ADD1|ADD1_pS457

	Difference in means	Adjusted p-value
CC-SLNB	-0.31146	0.9143937
ALND-SLNB	-1.490775	0.1225938
ALND-CC	-1.179315	0.2812572

CDH1|CDH1_pT790

	Difference in means	Adjusted p-value
CC-SLNB	0.11619096	0.9751819
ALND-SLNB	0.189905	0.927438
ALND-CC	0.07371404	0.9819512

CDH1|CDH1

	Difference in means	Adjusted p-value
CC-SLNB	0.09555867	0.8535741
ALND-SLNB	0.36929632	0.1047112
ALND-CC	0.27373765	0.1845027

ALCAM|ALCAM

	Difference in means	Adjusted p-value
CC-SLNB	0.32255267	0.5243473
ALND-SLNB	0.326431251	0.5163384
ALND-CC	0.003878581	0.9998725

MDK|MDK

	Difference in means	Adjusted p-value
CC-SLNB	-0.1333087	0.8365381
ALND-SLNB	0.1048035	0.8954015
ALND-CC	0.2381123	0.469223

FBP1|FBP1

	Difference in means	Adjusted p-value
CC-SLNB	0.3501584	0.2798694
ALND-SLNB	0.1596857	0.7627377
ALND-CC	-0.1904727	0.5969699

PSMB10|PSMB10

	Difference in means	Adjusted p-value
CC-SLNB	0.22628462	0.3313943
ALND-SLNB	0.15189475	0.6038884
ALND-CC	-0.07438987	0.8486606

PSMB10|PSMB10_pS229

	Difference in means	Adjusted p-value
CC-SLNB	0.41250665	0.3534717
ALND-SLNB	0.34949533	0.471544
ALND-CC	-0.06301132	0.9673881

LRG1|LRG1

	Difference in means	Adjusted p-value
CC-SLNB	-0.3706325	0.18352
ALND-SLNB	-0.2389408	0.486687
ALND-CC	0.1316917	0.7428398

KRT10|KRT10_pS16

	Difference in means	Adjusted p-value
CC-SLNB	0.9700315	0.4688089
ALND-SLNB	0.1443722	0.9785132
ALND-CC	-0.8256593	0.441144

KRT10|KRT10_pS159

	Difference in means	Adjusted p-value
CC-SLNB	0.00209111	0.999997
ALND-SLNB	-0.57190367	0.8574676
ALND-CC	-0.57399479	0.7994743

KRT10|KRT10

	Difference in means	Adjusted p-value
CC-SLNB	-0.2402257	0.5497106
ALND-SLNB	-0.3896352	0.2140881
ALND-CC	-0.1494095	0.7308254

GALM|GALM

	Difference in means	Adjusted p-value
CC-SLNB	0.023653314	0.9905304
ALND-SLNB	0.004440773	0.9996646
ALND-CC	-0.019212541	0.9915851

Uniprot ID	Description
P63104	14-3-3 zeta.
P62258	14-3-3 epsilon, variant.
P31947	14-3-3 sigma, stratifin.
P27348	14-3-3 theta.
P61158	Actin-like protein 3.
P23526	Adenosylhomocysteinase.
P84077	ADP ribosylation factor.
P54920	Alpha-soluble NSF attachment protein.
P12429	Annexin III.
P08758	Annexin V.
P02647	Apolipoprotein A-I.
P06576	ATP synthase beta chain.
P27824	Calnexin.
P04632	Calpain small subunit 1.
P27797	Calreticulin.
O43852	Calumenin.
P00915	Carbonic anhydrase I.
P07339	Cathepsin D.
O00299	Chloride intracellular channel protein 1.
P00403	Cytochrome C oxidase II subunit.
P05783	Cytokeratin 18.
P08727	Cytokeratin 19.
P02538	Cytokeratin 6A.
P08729	Cytokeratin 7.
P05787	Cytokeratin 8.
Q96KP4	Cytosolic nonspecific dipeptidase.
Q07507	Dermatopontin, tyrosine-rich acidic matrix protein.
P24534	Elongation factor 1-beta.
P26641	Elongation factor 1-gamma.
P14625	Endoplasmin.
P30084	Enoyl-CoA hydratase.
P63241	Eukaryotic translation initiation factor 5A (eIF-5A).
P52907	F-actin capping protein alpha-subunit, CapZ-alpha.
P47756	F-actin capping protein beta-subunit, CapZ-beta.
Q01469	Fatty acid-binding protein,(E-FABP).
P09382	Galectin-1.
P09211	Glutathione S-transferase P.
P21266	Gluthatione S-transferase Mu 3.
P48637	Gluthatione synthase.
P62873	Guanine nucleotide-binding protein, beta subunit 1.
P62879	Guanine nucleotide-binding protein, beta subunit 2.
P11142	Heat shock 70 (hsc 70).
P34932	Heat shock 70 kDa protein 4.
P07900	Heat shock protein 90 (hsp 90).
Q9NRV9	Heme binding protein 1.
P02023	Hemoglobin beta chain.
P61978	Heterogeneous nuclear ribinucleoprotein K.
P52597	Heterogeneous nuclear ribonucleoprotein F.
P55795	Heterogeneous nuclear ribonucleoprotein H'.
P09429	High mobility group protein 1. HMG-1.
Q14974	Importin beta-1 subunit, importin 90.
Q04760	Lactoylglutathione lyase.
P20700	Lamin B1.
P40121	Macrophage capping protein.
Q14764	Major vault protein. MVP.

Q14697	Neutral alpha-glicosidase AB.
P06748	Nucleophosmin.
P32119	Peroxiredoxin 2; thioredoxin peroxidase 1.
P30041	Peroxiredoxin 6.
P18669	Phosphoglycerate mutase isozyme B.
P68402	Platellet-activating factor acetylhydrolase IB beta subunit.
P35232	Prohibitin.
P61019	Ras-related protein RAB-2A.
P52565	Rho GDP-dissociation inhibitor 1, Rho GDI 1.
P52566	Rho GDP-dissociation inhibitor 2, Rho GDI 2.
P38646	Stress-70 protein, mitochondrial, grp 75.
P00441	Superoxide dismutase [Cu-Zn].
P04179	Superoxide dismutase Mn, mitochondrial.
P50990	T-complex protein 1 subunit theta.
P48643	T-complex protein 1 subunit epsilon.
P10599	Thioredoxin.
P02787	Transferrin.
P55072	Transitional endoplasmic reticulum ATPase.
P13693	Translationally controlled tumor protein (TCTP).
Q15363	Transmembrane emp24 domain trafficking protein 2.
P02766	Transthyretin.
P60174	Triosephosphate isomerase.
P09493	Tropomyosin 1 alpha chain (N-terminal part).
P07226	Tropomyosin 4 alpha chain.
P06753	Tropomyosin alpha 3 chain.
P68366	Tubulin alpha.
P68371	Tubulin beta.
P54578	Ubiquitin carboxyl-terminal hydrolase 14.
P45974	Ubiquitin carboxyl-terminal hydrolase 5.
Q96FW1	Ubiquitin thiolesterase protein OTUB1.
P38606	Vacuolar ATP synthase catalytic subunit A.

Uniprot ID	Protein names
P04217	Alpha-1B-glycoprotein (Alpha-1-B glycoprotein)
P01023	Alpha-2-macroglobulin (Alpha-2-M) (C3 and PZP-like alpha-2-macroglobulin domain-containing protein 5)
P49588	Alanine--tRNA ligase, cytoplasmic (EC 6.1.1.7) (Alanyl-tRNA synthetase) (AlaRS) (Renal carcinoma antigen NY-REN-42)
Q96IU4	Protein ABHD14B (EC 3.-.-.-) (Alpha/beta hydrolase domain-containing protein 14B) (Abhydrolase domain-containing protein 14B) (CCG1-interacting factor B)
P42765	3-ketoacyl-CoA thiolase, mitochondrial (EC 2.3.1.16) (Acetyl-CoA acetyltransferase) (EC 2.3.1.9) (Acetyl-CoA acyltransferase) (Acyl-CoA hydrolase, mitochondrial) (EC 3.1.2.-) (EC 3.1.2.1) (EC 3.1.2.2) (Beta-ketothiolase) (Mitochondrial 3-oxoacyl-CoA thiolase) (T1)
P49748	Very long-chain specific acyl-CoA dehydrogenase, mitochondrial (VLCAD) (EC 1.3.8.9)
P24752	Acetyl-CoA acetyltransferase, mitochondrial (EC 2.3.1.9) (Acetoacetyl-CoA thiolase) (T2)
Q99798	Aconitate hydratase, mitochondrial (Aconitase) (EC 4.2.1.3) (Citrate hydro-lyase)
P49753	Acyl-coenzyme A thioesterase 2, mitochondrial (Acyl-CoA thioesterase 2) (EC 3.1.2.2) (Acyl-coenzyme A thioester hydrolase 2a) (CTE-Ia) (Long-chain acyl-CoA thioesterase 2) (ZAP128)
P24666	Low molecular weight phosphotyrosine protein phosphatase (LMW-PTP) (LMW-PTPase) (EC 3.1.3.48) (Adipocyte acid phosphatase) (Low molecular weight cytosolic acid phosphatase) (EC 3.1.3.2) (Red cell acid phosphatase 1)
P60709	Actin, cytoplasmic 1 (Beta-actin) [Cleaved into: Actin, cytoplasmic 1, N-terminally processed]
P12814	Alpha-actinin-1 (Alpha-actinin cytoskeletal isoform) (F-actin cross-linking protein) (Non-muscle alpha-actinin-1)
O43707	Alpha-actinin-4 (Non-muscle alpha-actinin 4)
P61160	Actin-related protein 2 (Actin-like protein 2)
P61158	Actin-related protein 3 (Actin-like protein 3)
P35611	Alpha-adducin (Erythrocyte adducin subunit alpha)
Q8IUX7	Adipocyte enhancer-binding protein 1 (AE-binding protein 1) (Aortic carboxypeptidase-like protein)
P23526	Adenosylhomocysteinase (AdoHcyase) (EC 3.3.1.1) (S-adenosyl-L-homocysteine hydrolase)
Q09666	Neuroblast differentiation-associated protein AHNAK (Desmoyokin)
Q8IVF2	Protein AHNAK2
O95831	Apoptosis-inducing factor 1, mitochondrial (EC 1.1.1.-) (Programmed cell death protein 8)
P54819	Adenylate kinase 2, mitochondrial (AK 2) (EC 2.7.4.3) (ATP-AMP transphosphorylase 2) (ATP:AMP phosphotransferase) (Adenylate monophosphate kinase) [Cleaved into: Adenylate kinase 2, mitochondrial, N-terminally processed]
P15121	Aldo-keto reductase family 1 member B1 (EC 1.1.1.300) (EC 1.1.1.372) (EC 1.1.1.54) (Aldehyde reductase) (Aldose reductase) (AR) (EC 1.1.1.21)
O43488	Aflatoxin B1 aldehyde reductase member 2 (EC 1.1.1.n11) (AFB1 aldehyde reductase 1) (AFB1-AR 1) (Aldoketoreductase 7) (Succinic semialdehyde reductase) (SSA reductase)
P02768	Serum albumin
P00352	Retinal dehydrogenase 1 (RALDH 1) (RalDH1) (EC 1.2.1.-) (EC 1.2.1.36) (ALDH-E1) (ALHDII) (Aldehyde dehydrogenase family 1 member A1) (Aldehyde dehydrogenase, cytosolic)
P05091	Aldehyde dehydrogenase, mitochondrial (EC 1.2.1.3) (ALDH class 2) (ALDH-E2) (ALDHI)
P49189	4-trimethylaminobutyraldehyde dehydrogenase (TMABA-DH) (TMABALDH) (EC 1.2.1.47) (Aldehyde dehydrogenase E3 isozyme) (Aldehyde dehydrogenase family 9 member A1) (EC 1.2.1.3) (Gamma-aminobutyraldehyde dehydrogenase) (EC 1.2.1.19) (R-aminobutyraldehyde dehydrogenase) [Cleaved into: 4-trimethylaminobutyraldehyde dehydrogenase, N-terminally processed]

P04075	Fructose-bisphosphate aldolase A (EC 4.1.2.13) (Lung cancer antigen NY-LU-1) (Muscle-type aldolase)
P09972	Fructose-bisphosphate aldolase C (EC 4.1.2.13) (Brain-type aldolase)
P04083	Annexin A1 (Annexin I) (Annexin-1) (Calpactin II) (Calpactin-2) (Chromobindin-9) (Lipocortin I) (Phospholipase A2 inhibitory protein) (p35)
P07355	Annexin A2 (Annexin II) (Annexin-2) (Calpactin I heavy chain) (Calpactin-1 heavy chain) (Chromobindin-8) (Lipocortin II) (Placental anticoagulant protein IV) (PAP-IV) (Protein I) (p36)
P12429	Annexin A3 (35-alpha calcimedin) (Annexin III) (Annexin-3) (Inositol 1,2-cyclic phosphate 2-phosphohydrolase) (Lipocortin III) (Placental anticoagulant protein III) (PAP-III)
P09525	Annexin A4 (35-beta calcimedin) (Annexin IV) (Annexin-4) (Carbohydrate-binding protein p33/p41) (Chromobindin-4) (Endonexin I) (Lipocortin IV) (P32.5) (PP4-X) (Placental anticoagulant protein II) (PAP-II) (Protein II)
P08758	Annexin A5 (Anchorin CII) (Annexin V) (Annexin-5) (Calphobindin I) (CBP-I) (Endonexin II) (Lipocortin V) (Placental anticoagulant protein 4) (PP4) (Placental anticoagulant protein I) (PAP-I) (Thromboplastin inhibitor) (Vascular anticoagulant-alpha) (VAC-alpha)
P08133	Annexin A6 (67 kDa calelectrin) (Annexin VI) (Annexin-6) (Calphobindin-II) (CPB-II) (Chromobindin-20) (Lipocortin VI) (Protein III) (p68) (p70)
P63010	AP-2 complex subunit beta (AP105B) (Adaptor protein complex AP-2 subunit beta) (Adaptor-related protein complex 2 subunit beta) (Beta-2-adaptin) (Beta-adaptin) (Clathrin assembly protein complex 2 beta large chain) (Plasma membrane adaptor HA2/AP2 adaptin beta subunit)
P13798	Acylamino-acid-releasing enzyme (AARE) (EC 3.4.19.1) (Acyl-peptide hydrolase) (APH) (Acylaminoacyl-peptidase) (Oxidized protein hydrolase) (OPH)
P27695	DNA-(apurinic or apyrimidinic site) lyase (EC 3.1.-.-) (EC 4.2.99.18) (APEX nuclease) (APEN) (Apurinic-apyrimidinic endonuclease 1) (AP endonuclease 1) (APE-1) (REF-1) (Redox factor-1) [Cleaved into: DNA-(apurinic or apyrimidinic site) lyase, mitochondrial]
P02647	Apolipoprotein A-I (Apo-AI) (ApoA-I) (Apolipoprotein A1) [Cleaved into: Proapolipoprotein A-I (ProapoA-I); Truncated apolipoprotein A-I (Apolipoprotein A-I(1-242))]
P48444	Coatomer subunit delta (Archain) (Delta-coat protein) (Delta-COP)
Q07960	Rho GTPase-activating protein 1 (CDC42 GTPase-activating protein) (GTPase-activating protein rhoGAP) (Rho-related small GTPase protein activator) (Rho-type GTPase-activating protein 1) (p50-RhoGAP)
O15143	Actin-related protein 2/3 complex subunit 1B (Arp2/3 complex 41 kDa subunit) (p41-ARC)
O15144	Actin-related protein 2/3 complex subunit 2 (Arp2/3 complex 34 kDa subunit) (p34-ARC)
O15145	Actin-related protein 2/3 complex subunit 3 (Arp2/3 complex 21 kDa subunit) (p21-ARC)
P59998	Actin-related protein 2/3 complex subunit 4 (Arp2/3 complex 20 kDa subunit) (p20-ARC)
O15511	Actin-related protein 2/3 complex subunit 5 (Arp2/3 complex 16 kDa subunit) (p16-ARC)
P00966	Argininosuccinate synthase (EC 6.3.4.5) (Citrulline--aspartate ligase)
P31939	Bifunctional purine biosynthesis protein PURH [Cleaved into: Bifunctional purine biosynthesis protein PURH, N-terminally processed] [Includes: Phosphoribosylaminoimidazolecarboxamide formyltransferase (EC 2.1.2.3) (5-aminoimidazole-4-carboxamide ribonucleotide formyltransferase) (AICAR transformylase); IMP cyclohydrolase (EC 3.5.4.10) (ATIC) (IMP synthase) (Inosinicase)]
Q6DD88	Atlastin-3 (EC 3.6.5.-)
P24539	ATP synthase F(0) complex subunit B1, mitochondrial (ATP synthase peripheral stalk-membrane subunit b) (ATP synthase proton-transporting mitochondrial F(0) complex subunit B1) (ATP synthase subunit b) (ATPase subunit b)
P38606	V-type proton ATPase catalytic subunit A (V-ATPase subunit A) (EC 7.1.2.2) (V-ATPase 69 kDa subunit) (Vacuolar ATPase isoform VA68) (Vacuolar proton pump subunit alpha)

P21281	V-type proton ATPase subunit B, brain isoform (V-ATPase subunit B 2) (Endomembrane proton pump 58 kDa subunit) (HO57) (Vacuolar proton pump subunit B 2)
P61769	Beta-2-microglobulin [Cleaved into: Beta-2-microglobulin form pI 5.3]
P51572	B-cell receptor-associated protein 31 (BCR-associated protein 31) (Bap31) (6C6-AG tumor-associated antigen) (Protein CDM) (p28)
P21810	Biglycan (Bone/cartilage proteoglycan I) (PG-S1)
P53004	Biliverdin reductase A (BVR A) (EC 1.3.1.24) (Biliverdin-IX alpha-reductase)
O43684	Mitotic checkpoint protein BUB3
P01024	Complement C3 (C3 and PZP-like alpha-2-macroglobulin domain-containing protein 1) [Cleaved into: Complement C3 beta chain; C3-beta-c (C3bc); Complement C3 alpha chain; C3a anaphylatoxin; Acylation stimulating protein (ASP) (C3adesArg); Complement C3b alpha' chain; Complement C3c alpha' chain fragment 1; Complement C3dg fragment; Complement C3g fragment; Complement C3d fragment; Complement C3f fragment; Complement C3c alpha' chain fragment 2]
Q05682	Caldesmon (CDM)
P27797	Calreticulin (CRP55) (Calregulin) (Endoplasmic reticulum resident protein 60) (ERp60) (HACBP) (grp60)
Q86VP6	Cullin-associated NEDD8-dissociated protein 1 (Cullin-associated and neddylation-dissociated protein 1) (TBP-interacting protein of 120 kDa A) (TBP-interacting protein 120A) (p120 CAND1)
P27824	Calnexin (IP90) (Major histocompatibility complex class I antigen-binding protein p88) (p90)
Q01518	Adenylyl cyclase-associated protein 1 (CAP 1)
P40121	Macrophage-capping protein (Actin regulatory protein CAP-G)
P07384	Calpain-1 catalytic subunit (EC 3.4.22.52) (Calcium-activated neutral proteinase 1) (CANP 1) (Calpain mu-type) (Calpain-1 large subunit) (Cell proliferation-inducing gene 30 protein) (Micromolar-calpain) (muCANP)
P17655	Calpain-2 catalytic subunit (EC 3.4.22.53) (Calcium-activated neutral proteinase 2) (CANP 2) (Calpain M-type) (Calpain large polypeptide L2) (Calpain-2 large subunit) (Millimolar-calpain) (M-calpain)
P04632	Calpain small subunit 1 (CSS1) (Calcium-activated neutral proteinase small subunit) (CANP small subunit) (Calcium-dependent protease small subunit) (CDPS) (Calcium-dependent protease small subunit 1) (Calpain regulatory subunit)
P52907	F-actin-capping protein subunit alpha-1 (CapZ alpha-1)
P04040	Catalase (EC 1.11.1.6)
P16152	Carbonyl reductase [NADPH] 1 (EC 1.1.1.184) (15-hydroxyprostaglandin dehydrogenase [NADP(+)]) (EC 1.1.1.197) (NADPH-dependent carbonyl reductase 1) (Prostaglandin 9-ketoreductase) (Prostaglandin-E(2) 9-reductase) (EC 1.1.1.189) (Short chain dehydrogenase/reductase family 21C member 1)
P78371	T-complex protein 1 subunit beta (TCP-1-beta) (CCT-beta)
P49368	T-complex protein 1 subunit gamma (TCP-1-gamma) (CCT-gamma) (hTRiC5)
P48643	T-complex protein 1 subunit epsilon (TCP-1-epsilon) (CCT-epsilon)
P40227	T-complex protein 1 subunit zeta (TCP-1-zeta) (Acute morphine dependence-related protein 2) (CCT-zeta-1) (HTR3) (Tcp20)
Q99832	T-complex protein 1 subunit eta (TCP-1-eta) (CCT-eta) (HIV-1 Nef-interacting protein) [Cleaved into: T-complex protein 1 subunit eta, N-terminally processed]
P50990	T-complex protein 1 subunit theta (TCP-1-theta) (CCT-theta) (Chaperonin containing T-complex polypeptide 1 subunit 8) (Renal carcinoma antigen NY-REN-15)

P16070	CD44 antigen (CDw44) (Epican) (Extracellular matrix receptor III) (ECMR-III) (GP90 lymphocyte homing/adhesion receptor) (HUTCH-I) (Heparan sulfate proteoglycan) (Hermes antigen) (Hyaluronate receptor) (Phagocytic glycoprotein 1) (PGP-1) (Phagocytic glycoprotein I) (PGP-I) (CD antigen CD44)
P12830	Cadherin-1 (CAM 120/80) (Epithelial cadherin) (E-cadherin) (Uvomorulin) (CD antigen CD324) [Cleaved into: E-Cad/CTF1; E-Cad/CTF2; E-Cad/CTF3]
P23528	Cofilin-1 (18 kDa phosphoprotein) (p18) (Cofilin, non-muscle isoform)
Q07065	Cytoskeleton-associated protein 4 (63-kDa cytoskeleton-linking membrane protein) (Climp-63) (p63)
O00299	Chloride intracellular channel protein 1 (Chloride channel ABP) (Nuclear chloride ion channel 27) (NCC27) (Regulatory nuclear chloride ion channel protein) (hRNCC)
Q00610	Clathrin heavy chain 1 (Clathrin heavy chain on chromosome 17) (CLH-17)
P30085	UMP-CMP kinase (EC 2.7.4.14) (Deoxycytidylate kinase) (CK) (dCMP kinase) (Nucleoside-diphosphate kinase) (EC 2.7.4.6) (Uridine monophosphate/cytidine monophosphate kinase) (UMP/CMP kinase) (UMP/CMPK)
Q96KP4	Cytosolic non-specific dipeptidase (EC 3.4.13.18) (CNDP dipeptidase 2) (Carnosine dipeptidase II) (Epididymis secretory protein Li 13) (Glutamate carboxypeptidase-like protein 1) (Peptidase A)
P51911	Calponin-1 (Basic calponin) (Calponin H1, smooth muscle)
Q15417	Calponin-3 (Calponin, acidic isoform)
P09543	2',3'-cyclic-nucleotide 3'-phosphodiesterase (CNP) (CNPase) (EC 3.1.4.37)
Q99715	Collagen alpha-1(XII) chain
Q05707	Collagen alpha-1(XIV) chain (Undulin)
P39060	Collagen alpha-1(XVIII) chain [Cleaved into: Endostatin; Non-collagenous domain 1 (NC1)]
P02452	Collagen alpha-1(I) chain (Alpha-1 type I collagen)
P08123	Collagen alpha-2(I) chain (Alpha-2 type I collagen)
P02461	Collagen alpha-1(III) chain
P08572	Collagen alpha-2(IV) chain [Cleaved into: Canstatin]
P12109	Collagen alpha-1(VI) chain
P12110	Collagen alpha-2(VI) chain
P12111	Collagen alpha-3(VI) chain
P53621	Coatomer subunit alpha (Alpha-coat protein) (Alpha-COP) (HEP-COP) (HEPCOP) [Cleaved into: Xenin (Xenopsin-related peptide); Proxenin]
P35606	Coatomer subunit beta' (Beta'-coat protein) (Beta'-COP) (p102)
O14579	Coatomer subunit epsilon (Epsilon-coat protein) (Epsilon-COP)
P57737	Coronin-7 (Crn7) (70 kDa WD repeat tumor rejection antigen homolog)
Q14019	Coactosin-like protein
P00450	Ceruloplasmin (EC 1.16.3.1) (Ferroxidase)
Q08257	Quinone oxidoreductase (EC 1.6.5.5) (NADPH:quinone reductase) (Zeta-crystallin)
O75390	Citrate synthase, mitochondrial (EC 2.3.3.1) (Citrate (Si)-synthase)
P21291	Cysteine and glycine-rich protein 1 (Cysteine-rich protein 1) (CRP) (CRP1) (Epididymis luminal protein 141) (HEL-141)
P04080	Cystatin-B (CPI-B) (Liver thiol proteinase inhibitor) (Stefin-B)
P07339	Cathepsin D (EC 3.4.23.5) [Cleaved into: Cathepsin D light chain; Cathepsin D heavy chain]

P08311	Cathepsin G (CG) (EC 3.4.21.20)
Q9UBR2	Cathepsin Z (EC 3.4.18.1) (Cathepsin P) (Cathepsin X)
Q14247	Src substrate cortactin (Amplaxin) (Oncogene EMS1)
P00167	Cytochrome b5 (Microsomal cytochrome b5 type A) (MCB5)
P00387	NADH-cytochrome b5 reductase 3 (B5R) (Cytochrome b5 reductase) (EC 1.6.2.2) (Diaphorase-1) [Cleaved into: NADH-cytochrome b5 reductase 3 membrane-bound form; NADH-cytochrome b5 reductase 3 soluble form]
P99999	Cytochrome c
Q16643	Drebrin (Developmentally-regulated brain protein)
Q9UJU6	Drebrin-like protein (Cervical SH3P7) (Cervical mucin-associated protein) (Drebrin-F) (HPK1-interacting protein of 55 kDa) (HIP-55) (SH3 domain-containing protein 7)
P81605	Dermcidin (EC 3.4.-.-) (Preproteolysin) [Cleaved into: Survival-promoting peptide; DCD-1]
P07585	Decorin (Bone proteoglycan II) (PG-S2) (PG40)
O94760	N(G),N(G)-dimethylarginine dimethylaminohydrolase 1 (DDAH-1) (Dimethylarginine dimethylaminohydrolase 1) (EC 3.5.3.18) (DDAHI) (Dimethylargininase-1)
O95865	N(G),N(G)-dimethylarginine dimethylaminohydrolase 2 (DDAH-2) (Dimethylarginine dimethylaminohydrolase 2) (EC 3.5.3.18) (DDAHII) (Dimethylargininase-2) (Protein G6a) (S-phase protein)
Q96HY6	DDRGK domain-containing protein 1 (Dashurin) (UFM1-binding and PCI domain-containing protein 1)
Q92841	Probable ATP-dependent RNA helicase DDX17 (EC 3.6.4.13) (DEAD box protein 17) (DEAD box protein p72) (DEAD box protein p82) (RNA-dependent helicase p72)
Q08211	ATP-dependent RNA helicase A (EC 3.6.4.13) (DEAH box protein 9) (DExH-box helicase 9) (Leukophysin) (LKP) (Nuclear DNA helicase II) (NDH II) (RNA helicase A)
P09622	Dihydrolipoyl dehydrogenase, mitochondrial (EC 1.8.1.4) (Dihydrolipoamide dehydrogenase) (Glycine cleavage system L protein)
P11532	Dystrophin
P25685	DnaJ homolog subfamily B member 1 (DnaJ protein homolog 1) (Heat shock 40 kDa protein 1) (HSP40) (Heat shock protein 40) (Human DnaJ protein 1) (hDj-1)
Q9ULA0	Aspartyl aminopeptidase (EC 3.4.11.21)
Q9NY33	Dipeptidyl peptidase 3 (EC 3.4.14.4) (Dipeptidyl aminopeptidase III) (Dipeptidyl arylamidase III) (Dipeptidyl peptidase III) (DPP III) (Enkephalinase B)
Q16555	Dihydropyrimidinase-related protein 2 (DRP-2) (Collapsin response mediator protein 2) (CRMP-2) (N2A3) (Unc-33-like phosphoprotein 2) (ULIP-2)
Q14195	Dihydropyrimidinase-related protein 3 (DRP-3) (Collapsin response mediator protein 4) (CRMP-4) (Unc-33-like phosphoprotein 1) (ULIP-1)
Q14126	Desmoglein-2 (Cadherin family member 5) (HDGC)
P15924	Desmoplakin (DP) (250/210 kDa paraneoplastic pemphigus antigen)
Q14204	Cytoplasmic dynein 1 heavy chain 1 (Cytoplasmic dynein heavy chain 1) (Dynein heavy chain, cytosolic)
Q13409	Cytoplasmic dynein 1 intermediate chain 2 (Cytoplasmic dynein intermediate chain 2) (Dynein intermediate chain 2, cytosolic) (DH IC-2)
Q13011	Delta(3,5)-Delta(2,4)-dienoyl-CoA isomerase, mitochondrial (EC 5.3.3.-)
P30084	Enoyl-CoA hydratase, mitochondrial (EC 4.2.1.17) (Enoyl-CoA hydratase 1) (Short-chain enoyl-CoA hydratase) (SCEH)
Q15075	Early endosome antigen 1 (Endosome-associated protein p162) (Zinc finger FYVE domain-containing protein 2)

P68104	Elongation factor 1-alpha 1 (EF-1-alpha-1) (Elongation factor Tu) (EF-Tu) (Eukaryotic elongation factor 1 A-1) (eEF1A-1) (Leukocyte receptor cluster member 7)
P29692	Elongation factor 1-delta (EF-1-delta) (Antigen NY-CO-4)
P13639	Elongation factor 2 (EF-2)
Q96C19	EF-hand domain-containing protein D2 (Swiprosin-1)
Q9H4M9	EH domain-containing protein 1 (PAST homolog 1) (hPAST1) (Testilin)
Q9NZN4	EH domain-containing protein 2 (PAST homolog 2)
P41091	Eukaryotic translation initiation factor 2 subunit 3 (Eukaryotic translation initiation factor 2 subunit gamma X) (eIF-2-gamma X) (eIF-2gX)
P56537	Eukaryotic translation initiation factor 6 (eIF-6) (B(2)GCN homolog) (B4 integrin interactor) (CAB) (p27(BBP))
P55884	Eukaryotic translation initiation factor 3 subunit B (eIF3b) (Eukaryotic translation initiation factor 3 subunit 9) (Prt1 homolog) (hPrt1) (eIF-3-eta) (eIF3 p110) (eIF3 p116)
O15371	Eukaryotic translation initiation factor 3 subunit D (eIF3d) (Eukaryotic translation initiation factor 3 subunit 7) (eIF-3-zeta) (eIF3 p66)
P23588	Eukaryotic translation initiation factor 4B (eIF-4B)
Q04637	Eukaryotic translation initiation factor 4 gamma 1 (eIF-4-gamma 1) (eIF-4G 1) (eIF-4G1) (p220)
Q9Y6C2	EMILIN-1 (Elastin microfibril interface-located protein 1) (Elastin microfibril interfacer 1)
P06733	Alpha-enolase (EC 4.2.1.11) (2-phospho-D-glycerate hydro-lyase) (C-myc promoter-binding protein) (Enolase 1) (MBP-1) (MPB-1) (Non-neural enolase) (NNE) (Phosphopyruvate hydratase) (Plasminogen-binding protein)
P09104	Gamma-enolase (EC 4.2.1.11) (2-phospho-D-glycerate hydro-lyase) (Enolase 2) (Neural enolase) (Neuron-specific enolase) (NSE)
O43491	Band 4.1-like protein 2 (Generally expressed protein 4.1) (4.1G)
P07099	Epoxide hydrolase 1 (EC 3.3.2.9) (Epoxide hydratase) (Microsomal epoxide hydrolase) (mEH)
P07814	Bifunctional glutamate/proline--tRNA ligase (Bifunctional aminoacyl-tRNA synthetase) (Cell proliferation-inducing gene 32 protein) (Glutamatyl-prolyl-tRNA synthetase) [Includes: Glutamate--tRNA ligase (EC 6.1.1.17) (Glutamyl-tRNA synthetase) (GluRS); Proline--tRNA ligase (EC 6.1.1.15) (Prolyl-tRNA synthetase)]
P84090	Enhancer of rudimentary homolog
P10768	S-formylglutathione hydrolase (FGH) (EC 3.1.2.12) (Esterase D) (Methylumbelliferyl-acetate deacetylase) (EC 3.1.1.56)
P13804	Electron transfer flavoprotein subunit alpha, mitochondrial (Alpha-ETF)
P38117	Electron transfer flavoprotein subunit beta (Beta-ETF)
Q01844	RNA-binding protein EWS (EWS oncogene) (Ewing sarcoma breakpoint region 1 protein)
P15311	Ezrin (Cytovillin) (Villin-2) (p81)
P23142	Fibulin-1 (FIBL-1)
P35555	Fibrillin-1 [Cleaved into: Asprosin]
P09467	Fructose-1,6-bisphosphatase 1 (FBPase 1) (EC 3.1.3.11) (D-fructose-1,6-bisphosphate 1-phosphohydrolase 1) (Liver FBPase)
Q96AC1	Fermitin family homolog 2 (Kindlin-2) (Mitogen-inducible gene 2 protein) (MIG-2) (Pleckstrin homology domain-containing family C member 1) (PH domain-containing family C member 1)
P02671	Fibrinogen alpha chain [Cleaved into: Fibrinopeptide A; Fibrinogen alpha chain]
P02675	Fibrinogen beta chain [Cleaved into: Fibrinopeptide B; Fibrinogen beta chain]
P02679	Fibrinogen gamma chain

Q00688	Peptidyl-prolyl cis-trans isomerase FKBP3 (PPIase FKBP3) (EC 5.2.1.8) (25 kDa FK506-binding protein) (25 kDa FKBP) (FKBP-25) (FK506-binding protein 3) (FKBP-3) (Immunophilin FKBP25) (Rapamycin-selective 25 kDa immunophilin) (Rotamase)
Q02790	Peptidyl-prolyl cis-trans isomerase FKBP4 (PPIase FKBP4) (EC 5.2.1.8) (51 kDa FK506-binding protein) (FKBP51) (52 kDa FK506-binding protein) (52 kDa FKBP) (FKBP-52) (59 kDa immunophilin) (p59) (FK506-binding protein 4) (FKBP-4) (FKBP59) (HSP-binding immunophilin) (HBI) (Immunophilin FKBP52) (Rotamase) [Cleaved into: Peptidyl-prolyl cis-trans isomerase FKBP4, N-terminally processed]
P21333	Filamin-A (FLN-A) (Actin-binding protein 280) (ABP-280) (Alpha-filamin) (Endothelial actin-binding protein) (Filamin-1) (Non-muscle filamin)
O75369	Filamin-B (FLN-B) (ABP-278) (ABP-280 homolog) (Actin-binding-like protein) (Beta-filamin) (Filamin homolog 1) (Fh1) (Filamin-3) (Thyroid autoantigen) (Truncated actin-binding protein) (Truncated ABP)
O75955	Flotillin-1
P02751	Fibronectin (FN) (Cold-insoluble globulin) (CIG) [Cleaved into: Anastellin; Ugl-Y1; Ugl-Y2; Ugl-Y3]
Q16658	Fascin (55 kDa actin-bundling protein) (Singed-like protein) (p55)
P02794	Ferritin heavy chain (Ferritin H subunit) (EC 1.16.3.1) (Cell proliferation-inducing gene 15 protein) [Cleaved into: Ferritin heavy chain, N-terminally processed]
P02792	Ferritin light chain (Ferritin L subunit)
P35637	RNA-binding protein FUS (75 kDa DNA-pairing protein) (Oncogene FUS) (Oncogene TLS) (POMp75) (Translocated in liposarcoma protein)
P11413	Glucose-6-phosphate 1-dehydrogenase (G6PD) (EC 1.1.1.49)
Q14697	Neutral alpha-glucosidase AB (EC 3.2.1.207) (Alpha-glucosidase 2) (Glucosidase II subunit alpha)
P04406	Glyceraldehyde-3-phosphate dehydrogenase (GAPDH) (EC 1.2.1.12) (Peptidyl-cysteine S-nitrosylase GAPDH) (EC 2.6.99.-)
P31150	Rab GDP dissociation inhibitor alpha (Rab GDI alpha) (Guanosine diphosphate dissociation inhibitor 1) (GDI-1) (Oligophrenin-2) (Protein XAP-4)
P50395	Rab GDP dissociation inhibitor beta (Rab GDI beta) (Guanosine diphosphate dissociation inhibitor 2) (GDI-2)
Q04760	Lactoylglutathione lyase (EC 4.4.1.5) (Aldoketomutase) (Glyoxalase I) (Glx I) (Ketone-aldehyde mutase) (Methylglyoxalase) (S-D-lactoylglutathione methylglyoxal lyase)
P00367	Glutamate dehydrogenase 1, mitochondrial (GDH 1) (EC 1.4.1.3)
Q5JWF2	Guanine nucleotide-binding protein G(s) subunit alpha isoforms XLas (Adenylate cyclase-stimulating G alpha protein) (Extra large alphas protein) (XLalphas)
P62879	Guanine nucleotide-binding protein G(I)/G(S)/G(T) subunit beta-2 (G protein subunit beta-2) (Transducin beta chain 2)
Q14789	Golgin subfamily B member 1 (372 kDa Golgi complex-associated protein) (GCP372) (Giantin) (Macrogolgin)
P17174	Aspartate aminotransferase, cytoplasmic (cAspAT) (EC 2.6.1.1) (EC 2.6.1.3) (Cysteine aminotransferase, cytoplasmic) (Cysteine transaminase, cytoplasmic) (cCAT) (Glutamate oxaloacetate transaminase 1) (Transaminase A)
P00505	Aspartate aminotransferase, mitochondrial (mAspAT) (EC 2.6.1.1) (EC 2.6.1.7) (Fatty acid-binding protein) (FABP-1) (Glutamate oxaloacetate transaminase 2) (Kynurenine aminotransferase 4) (Kynurenine aminotransferase IV) (Kynurenine--oxoglutarate transaminase 4) (Kynurenine--oxoglutarate transaminase IV) (Plasma membrane-associated fatty acid-binding protein) (FABPpm) (Transaminase A)
P06744	Glucose-6-phosphate isomerase (GPI) (EC 5.3.1.9) (Autocrine motility factor) (AMF) (Neuroleukin) (NLK) (Phosphoglucose isomerase) (PGI) (Phosphohexose isomerase) (PHI) (Sperm antigen 36) (SA-36)
P62993	Growth factor receptor-bound protein 2 (Adapter protein GRB2) (Protein Ash) (SH2/SH3 adapter GRB2)
Q9UBQ7	Glyoxylate reductase/hydroxypyruvate reductase (EC 1.1.1.79) (EC 1.1.1.81)
P06396	Gelsolin (AGEL) (Actin-depolymerizing factor) (ADF) (Brevin)
P48637	Glutathione synthetase (GSH synthetase) (GSH-S) (EC 6.3.2.3) (Glutathione synthase)

P78417	Glutathione S-transferase omega-1 (GSTO-1) (EC 2.5.1.18) (Glutathione S-transferase omega 1-1) (GSTO 1-1) (Glutathione-dependent dehydroascorbate reductase) (EC 1.8.5.1) (Monomethylarsonic acid reductase) (MMA(V) reductase) (EC 1.20.4.2) (S-(Phenacyl)glutathione reductase) (SPG-R)
P09211	Glutathione S-transferase P (EC 2.5.1.18) (GST class-pi) (GSTP1-1)
O75367	Core histone macro-H2A.1 (Histone macroH2A1) (mH2A1) (Histone H2A.y) (H2A/y) (Medulloblastoma antigen MU-MB-50.205)
P40939	Trifunctional enzyme subunit alpha, mitochondrial (78 kDa gastrin-binding protein) (Monolysocardiolipin acyltransferase) (EC 2.3.1.-) (TP-alpha) [Includes: Long-chain enoyl-CoA hydratase (EC 4.2.1.17); Long chain 3-hydroxyacyl-CoA dehydrogenase (EC 1.1.1.211)]
P55084	Trifunctional enzyme subunit beta, mitochondrial (TP-beta) [Includes: 3-ketoacyl-CoA thiolase (EC 2.3.1.155) (EC 2.3.1.16) (Acetyl-CoA acyltransferase) (Beta-ketothiolase)]
P12081	Histidine--tRNA ligase, cytoplasmic (EC 6.1.1.21) (Histidyl-tRNA synthetase) (HisRS)
P68871	Hemoglobin subunit beta (Beta-globin) (Hemoglobin beta chain) [Cleaved into: LVV-hemorphin-7; Spinorphin]
P51858	Hepatoma-derived growth factor (HDGF) (High mobility group protein 1-like 2) (HMG-1L2)
Q00341	Vigilin (High density lipoprotein-binding protein) (HDL-binding protein)
Q9NRV9	Heme-binding protein 1 (p22HBP)
Q6NVY1	3-hydroxyisobutyryl-CoA hydrolase, mitochondrial (EC 3.1.2.4) (3-hydroxyisobutyryl-coenzyme A hydrolase) (HIB-CoA hydrolase) (HIBYL-CoA-H)
P16401	Histone H1.5 (Histone H1a) (Histone H1b) (Histone H1s-3)
P10412	Histone H1.4 (Histone H1b) (Histone H1s-4)
P19367	Hexokinase-1 (EC 2.7.1.1) (Brain form hexokinase) (Hexokinase type I) (HK I) (Hexokinase-A)
P04439	HLA class I histocompatibility antigen, A alpha chain (Human leukocyte antigen A) (HLA-A)
P09429	High mobility group protein B1 (High mobility group protein 1) (HMG-1)
P09651	Heterogeneous nuclear ribonucleoprotein A1 (hnRNP A1) (Helix-destabilizing protein) (Single-strand RNA-binding protein) (hnRNP core protein A1) [Cleaved into: Heterogeneous nuclear ribonucleoprotein A1, N-terminally processed]
P22626	Heterogeneous nuclear ribonucleoproteins A2/B1 (hnRNP A2/B1)
P51991	Heterogeneous nuclear ribonucleoprotein A3 (hnRNP A3)
Q99729	Heterogeneous nuclear ribonucleoprotein A/B (hnRNP A/B) (APOBEC1-binding protein 1) (ABBP-1)
P07910	Heterogeneous nuclear ribonucleoproteins C1/C2 (hnRNP C1/C2)
Q14103	Heterogeneous nuclear ribonucleoprotein D0 (hnRNP D0) (AU-rich element RNA-binding protein 1)
P52597	Heterogeneous nuclear ribonucleoprotein F (hnRNP F) (Nucleolin-like protein mcs94-1) [Cleaved into: Heterogeneous nuclear ribonucleoprotein F, N-terminally processed]
P31943	Heterogeneous nuclear ribonucleoprotein H (hnRNP H) [Cleaved into: Heterogeneous nuclear ribonucleoprotein H, N-terminally processed]
P31942	Heterogeneous nuclear ribonucleoprotein H3 (hnRNP H3) (Heterogeneous nuclear ribonucleoprotein 2H9) (hnRNP 2H9)
P61978	Heterogeneous nuclear ribonucleoprotein K (hnRNP K) (Transformation up-regulated nuclear protein) (TUNP)
P14866	Heterogeneous nuclear ribonucleoprotein L (hnRNP L)
P52272	Heterogeneous nuclear ribonucleoprotein M (hnRNP M)
O43390	Heterogeneous nuclear ribonucleoprotein R (hnRNP R)
Q00839	Heterogeneous nuclear ribonucleoprotein U (hnRNP U) (GRIP120) (Nuclear p120 ribonucleoprotein) (Scaffold-attachment factor A) (SAF-A) (p120) (pp120)

Q1KMD3	Heterogeneous nuclear ribonucleoprotein U-like protein 2 (Scaffold-attachment factor A2) (SAF-A2)
Q5SSJ5	Heterochromatin protein 1-binding protein 3 (Protein HP1-BP74)
P07900	Heat shock protein HSP 90-alpha (Heat shock 86 kDa) (HSP 86) (HSP86) (Lipopolysaccharide-associated protein 2) (LAP-2) (LPS-associated protein 2) (Renal carcinoma antigen NY-REN-38)
P08238	Heat shock protein HSP 90-beta (HSP 90) (Heat shock 84 kDa) (HSP 84) (HSP84)
P14625	Endoplasmin (94 kDa glucose-regulated protein) (GRP-94) (Heat shock protein 90 kDa beta member 1) (Tumor rejection antigen 1) (gp96 homolog)
P34932	Heat shock 70 kDa protein 4 (HSP70RY) (Heat shock 70-related protein APG-2)
P11021	Endoplasmic reticulum chaperone BiP (EC 3.6.4.10) (78 kDa glucose-regulated protein) (GRP-78) (Binding-immunoglobulin protein) (BiP) (Heat shock protein 70 family protein 5) (HSP70 family protein 5) (Heat shock protein family A member 5) (Immunoglobulin heavy chain-binding protein)
P11142	Heat shock cognate 71 kDa protein (Heat shock 70 kDa protein 8) (Lipopolysaccharide-associated protein 1) (LAP-1) (LPS-associated protein 1)
P38646	Stress-70 protein, mitochondrial (75 kDa glucose-regulated protein) (GRP-75) (Heat shock 70 kDa protein 9) (Mortalin) (MOT) (Peptide-binding protein 74) (PBP74)
P04792	Heat shock protein beta-1 (HspB1) (28 kDa heat shock protein) (Estrogen-regulated 24 kDa protein) (Heat shock 27 kDa protein) (HSP 27) (Stress-responsive protein 27) (SRP27)
P10809	60 kDa heat shock protein, mitochondrial (EC 5.6.1.7) (60 kDa chaperonin) (Chaperonin 60) (CPN60) (Heat shock protein 60) (HSP-60) (Hsp60) (HuCHA60) (Mitochondrial matrix protein P1) (P60 lymphocyte protein)
P61604	10 kDa heat shock protein, mitochondrial (Hsp10) (10 kDa chaperonin) (Chaperonin 10) (CPN10) (Early-pregnancy factor) (EPF)
Q7Z6Z7	E3 ubiquitin-protein ligase HUWE1 (EC 2.3.2.26) (ARF-binding protein 1) (ARF-BP1) (HECT, UBA and WWE domain-containing protein 1) (HECT-type E3 ubiquitin transferase HUWE1) (Homologous to E6AP carboxyl terminus homologous protein 9) (HectH9) (Large structure of UREB1) (LASU1) (Mcl-1 ubiquitin ligase E3) (Mule) (Upstream regulatory element-binding protein 1) (URE-B1) (URE-binding protein 1)
Q9Y4L1	Hypoxia up-regulated protein 1 (150 kDa oxygen-regulated protein) (ORP-150) (170 kDa glucose-regulated protein) (GRP-170)
O75874	Isocitrate dehydrogenase [NADP] cytoplasmic (IDH) (EC 1.1.1.42) (Cytosolic NADP-isocitrate dehydrogenase) (IDP) (NADP(+)-specific ICDH) (Oxalosuccinate decarboxylase)
O00425	Insulin-like growth factor 2 mRNA-binding protein 3 (IGF2 mRNA-binding protein 3) (IMP-3) (IGF-II mRNA-binding protein 3) (KH domain-containing protein overexpressed in cancer) (hKOC) (VICKZ family member 3)
Q12905	Interleukin enhancer-binding factor 2 (Nuclear factor of activated T-cells 45 kDa)
Q12906	Interleukin enhancer-binding factor 3 (Double-stranded RNA-binding protein 76) (DRBP76) (M-phase phosphoprotein 4) (MPP4) (Nuclear factor associated with dsRNA) (NFAR) (Nuclear factor of activated T-cells 90 kDa) (NF-AT-90) (Translational control protein 80) (TCP80)
Q27J81	Inverted formin-2 (HBEBP2-binding protein C)
P46940	Ras GTPase-activating-like protein IQGAP1 (p195)
Q13576	Ras GTPase-activating-like protein IQGAP2
P05556	Integrin beta-1 (Fibronectin receptor subunit beta) (Glycoprotein IIa) (GPIIA) (VLA-4 subunit beta) (CD antigen CD29)
P19823	Inter-alpha-trypsin inhibitor heavy chain H2 (ITI heavy chain H2) (ITI-HC2) (Inter-alpha-inhibitor heavy chain 2) (Inter-alpha-trypsin inhibitor complex component II) (Serum-derived hyaluronan-associated protein) (SHAP)
P14923	Junction plakoglobin (Catenin gamma) (Desmoplakin III) (Desmoplakin-3)
Q96CX2	BTB/POZ domain-containing protein KCTD12 (Pfetin) (Predominantly fetal expressed T1 domain)

Q92945	Far upstream element-binding protein 2 (FUSE-binding protein 2) (KH type-splicing regulatory protein) (KSRP) (p75)
P33176	Kinesin-1 heavy chain (Conventional kinesin heavy chain) (Ubiquitous kinesin heavy chain) (UKHC)
Q14974	Importin subunit beta-1 (Importin-90) (Karyopherin subunit beta-1) (Nuclear factor p97) (Pore targeting complex 97 kDa subunit) (PTAC97)
P04264	Keratin, type II cytoskeletal 1 (67 kDa cytokeratin) (Cytokeratin-1) (CK-1) (Hair alpha protein) (Keratin-1) (K1) (Type-II keratin Kb1)
P13645	Keratin, type I cytoskeletal 10 (Cytokeratin-10) (CK-10) (Keratin-10) (K10)
P02533	Keratin, type I cytoskeletal 14 (Cytokeratin-14) (CK-14) (Keratin-14) (K14)
P08779	Keratin, type I cytoskeletal 16 (Cytokeratin-16) (CK-16) (Keratin-16) (K16)
Q04695	Keratin, type I cytoskeletal 17 (39.1) (Cytokeratin-17) (CK-17) (Keratin-17) (K17)
P05783	Keratin, type I cytoskeletal 18 (Cell proliferation-inducing gene 46 protein) (Cytokeratin-18) (CK-18) (Keratin-18) (K18)
P08727	Keratin, type I cytoskeletal 19 (Cytokeratin-19) (CK-19) (Keratin-19) (K19)
P35908	Keratin, type II cytoskeletal 2 epidermal (Cytokeratin-2e) (CK-2e) (Epithelial keratin-2e) (Keratin-2 epidermis) (Keratin-2e) (K2e) (Type-II keratin Kb2)
P05787	Keratin, type II cytoskeletal 8 (Cytokeratin-8) (CK-8) (Keratin-8) (K8) (Type-II keratin Kb8)
P35527	Keratin, type I cytoskeletal 9 (Cytokeratin-9) (CK-9) (Keratin-9) (K9)
Q86UP2	Kinectin (CG-1 antigen) (Kinesin receptor)
P07942	Laminin subunit beta-1 (Laminin B1 chain) (Laminin-1 subunit beta) (Laminin-10 subunit beta) (Laminin-12 subunit beta) (Laminin-2 subunit beta) (Laminin-6 subunit beta) (Laminin-8 subunit beta)
P55268	Laminin subunit beta-2 (Laminin B1s chain) (Laminin-11 subunit beta) (Laminin-14 subunit beta) (Laminin-15 subunit beta) (Laminin-3 subunit beta) (Laminin-4 subunit beta) (Laminin-7 subunit beta) (Laminin-9 subunit beta) (S-laminin subunit beta) (S-LAM beta)
P11047	Laminin subunit gamma-1 (Laminin B2 chain) (Laminin-1 subunit gamma) (Laminin-10 subunit gamma) (Laminin-11 subunit gamma) (Laminin-2 subunit gamma) (Laminin-3 subunit gamma) (Laminin-4 subunit gamma) (Laminin-6 subunit gamma) (Laminin-7 subunit gamma) (Laminin-8 subunit gamma) (Laminin-9 subunit gamma) (S-laminin subunit gamma) (S-LAM gamma)
P28838	Cytosol aminopeptidase (EC 3.4.11.1) (Leucine aminopeptidase 3) (LAP-3) (Leucyl aminopeptidase) (Peptidase S) (Proline aminopeptidase) (EC 3.4.11.5) (Prolyl aminopeptidase)
Q14847	LIM and SH3 domain protein 1 (LASP-1) (Metastatic lymph node gene 50 protein) (MLN 50)
P13796	Plastin-2 (L-plastin) (LC64P) (Lymphocyte cytosolic protein 1) (LCP-1)
P00338	L-lactate dehydrogenase A chain (LDH-A) (EC 1.1.1.27) (Cell proliferation-inducing gene 19 protein) (LDH muscle subunit) (LDH-M) (Renal carcinoma antigen NY-REN-59)
P07195	L-lactate dehydrogenase B chain (LDH-B) (EC 1.1.1.27) (LDH heart subunit) (LDH-H) (Renal carcinoma antigen NY-REN-46)
P09382	Galectin-1 (Gal-1) (14 kDa laminin-binding protein) (HLBP14) (14 kDa lectin) (Beta-galactoside-binding lectin L-14-I) (Galaptin) (HBL) (HPL) (Lactose-binding lectin 1) (Lectin galactoside-binding soluble 1) (Putative MAPK-activating protein PM12) (S-Lac lectin 1)
P17931	Galectin-3 (Gal-3) (35 kDa lectin) (Carbohydrate-binding protein 35) (CBP 35) (Galactose-specific lectin 3) (Galactoside-binding protein) (GALBP) (IgE-binding protein) (L-31) (Laminin-binding protein) (Lectin L-29) (Mac-2 antigen)
Q08380	Galectin-3-binding protein (Basement membrane autoantigen p105) (Lectin galactoside-binding soluble 3-binding protein) (Mac-2-binding protein) (MAC2BP) (Mac-2 BP) (Tumor-associated antigen 90K)
Q9NZU5	LIM and cysteine-rich domains protein 1 (Dyxin)
P02545	Prelamin-A/C [Cleaved into: Lamin-A/C (70 kDa lamin) (Renal carcinoma antigen NY-REN-32)]
P20700	Lamin-B1

Q03252	Lamin-B2
Q93052	Lipoma-preferred partner (LIM domain-containing preferred translocation partner in lipoma)
Q14767	Latent-transforming growth factor beta-binding protein 2 (LTBP-2)
Q9Y383	Putative RNA-binding protein Luc7-like 2
P51884	Lumican (Keratan sulfate proteoglycan lumican) (KSPG lumican)
P61626	Lysozyme C (EC 3.2.1.17) (1,4-beta-N-acetylmuramidase C)
Q02750	Dual specificity mitogen-activated protein kinase kinase 1 (MAP kinase kinase 1) (MAPKK 1) (MKK1) (EC 2.7.12.2) (ERK activator kinase 1) (MAPK/ERK kinase 1) (MEK 1)
P43243	Matrin-3
P40925	Malate dehydrogenase, cytoplasmic (EC 1.1.1.37) (Cytosolic malate dehydrogenase) (Diiodophenylpyruvate reductase) (EC 1.1.1.96)
P40926	Malate dehydrogenase, mitochondrial (EC 1.1.1.37)
P03971	Muellerian-inhibiting factor (Anti-Muellerian hormone) (AMH) (Muellerian-inhibiting substance) (MIS)
Q14165	Malectin
P05164	Myeloperoxidase (MPO) (EC 1.11.2.2) [Cleaved into: Myeloperoxidase; 89 kDa myeloperoxidase; 84 kDa myeloperoxidase; Myeloperoxidase light chain; Myeloperoxidase heavy chain]
P25325	3-mercaptopyruvate sulfurtransferase (MST) (EC 2.8.1.2)
P26038	Moesin (Membrane-organizing extension spike protein)
P11586	C-1-tetrahydrofolate synthase, cytoplasmic (C1-THF synthase) [Cleaved into: C-1-tetrahydrofolate synthase, cytoplasmic, N-terminally processed] [Includes: Methylenetetrahydrofolate dehydrogenase (EC 1.5.1.5); Methenyltetrahydrofolate cyclohydrolase (EC 3.5.4.9); Formyltetrahydrofolate synthetase (EC 6.3.4.3)]
Q14764	Major vault protein (MVP) (Lung resistance-related protein)
P35580	Myosin-10 (Cellular myosin heavy chain, type B) (Myosin heavy chain 10) (Myosin heavy chain, non-muscle IIb) (Non-muscle myosin heavy chain B) (NMMHC-B) (Non-muscle myosin heavy chain IIb) (NMMHC II-b) (NMMHC-IIB)
P35749	Myosin-11 (Myosin heavy chain 11) (Myosin heavy chain, smooth muscle isoform) (SMMHC)
Q7Z406	Myosin-14 (Myosin heavy chain 14) (Myosin heavy chain, non-muscle IIc) (Non-muscle myosin heavy chain IIc) (NMHC II-C)
P35579	Myosin-9 (Cellular myosin heavy chain, type A) (Myosin heavy chain 9) (Myosin heavy chain, non-muscle IIa) (Non-muscle myosin heavy chain A) (NMMHC-A) (Non-muscle myosin heavy chain IIa) (NMMHC II-a) (NMMHC-IIA)
P60660	Myosin light polypeptide 6 (17 kDa myosin light chain) (LC17) (Myosin light chain 3) (MLC-3) (Myosin light chain alkali 3) (Myosin light chain A3) (Smooth muscle and nonmuscle myosin light chain alkali 6)
O00159	Unconventional myosin-Ic (Myosin I beta) (MMI-beta) (MMIb)
Q9UM54	Unconventional myosin-VI (Unconventional myosin-6)
Q9NZM1	Myoferlin (Fer-1-like protein 3)
P43490	Nicotinamide phosphoribosyltransferase (NAmPRTase) (Nampt) (EC 2.4.2.12) (Pre-B-cell colony-enhancing factor 1) (Pre-B cell-enhancing factor) (Visfatin)
Q99733	Nucleosome assembly protein 1-like 4 (Nucleosome assembly protein 2) (NAP-2)
O43776	Asparagine--tRNA ligase, cytoplasmic (EC 6.1.1.22) (Asparaginyl-tRNA synthetase) (AsnRS) (Asparaginyl-tRNA synthetase 1)
Q14112	Nidogen-2 (NID-2) (Osteonidogen)
P55786	Puromycin-sensitive aminopeptidase (PSA) (EC 3.4.11.14) (Cytosol alanyl aminopeptidase) (AAP-S)

P06748	Nucleophosmin (NPM) (Nucleolar phosphoprotein B23) (Nucleolar protein NO38) (Numatrin)
P46459	Vesicle-fusing ATPase (EC 3.6.4.6) (N-ethylmaleimide-sensitive fusion protein) (NEM-sensitive fusion protein) (Vesicular-fusion protein NSF)
Q9UNZ2	NSFL1 cofactor p47 (UBX domain-containing protein 2C) (p97 cofactor p47)
Q02818	Nucleobindin-1 (CALNUC)
Q14980	Nuclear mitotic apparatus protein 1 (Nuclear matrix protein-22) (NMP-22) (Nuclear mitotic apparatus protein) (NuMA protein) (SP-H antigen)
P20774	Mimecan (Osteoglycin) (Osteoinductive factor) (OIF)
Q9NTK5	Obg-like ATPase 1 (DNA damage-regulated overexpressed in cancer 45) (DOC45) (GTP-binding protein 9)
Q96FW1	Ubiquitin thioesterase OTUB1 (EC 3.4.19.12) (Deubiquitinating enzyme OTUB1) (OTU domain-containing ubiquitin aldehyde-binding protein 1) (Otubain-1) (hOTU1) (Ubiquitin-specific-processing protease OTUB1)
P07237	Protein disulfide-isomerase (PDI) (EC 5.3.4.1) (Cellular thyroid hormone-binding protein) (Prolyl 4-hydroxylase subunit beta) (p55)
Q9UQ80	Proliferation-associated protein 2G4 (Cell cycle protein p38-2G4 homolog) (hG4-1) (ErbB3-binding protein 1)
P11940	Polyadenylate-binding protein 1 (PABP-1) (Poly(A)-binding protein 1)
Q99497	Protein/nucleic acid deglycase DJ-1 (EC 3.1.2.-) (EC 3.5.1.-) (EC 3.5.1.124) (Maillard deglycase) (Oncogene DJ1) (Parkinson disease protein 7) (Parkinsonism-associated deglycase) (Protein DJ-1) (DJ-1)
P09874	Poly [ADP-ribose] polymerase 1 (PARP-1) (EC 2.4.2.30) (ADP-ribosyltransferase diphtheria toxin-like 1) (ARTD1) (DNA ADP-ribosyltransferase PARP1) (EC 2.4.2.-) (NAD(+) ADP-ribosyltransferase 1) (ADPRT 1) (Poly[ADP-ribose] synthase 1) (Protein poly-ADP-ribosyltransferase PARP1) (EC 2.4.2.-)
P22061	Protein-L-isoaspartate(D-aspartate) O-methyltransferase (PIMT) (EC 2.1.1.77) (L-isoaspartyl protein carboxyl methyltransferase) (Protein L-isoaspartyl/D-aspartyl methyltransferase) (Protein-beta-aspartate methyltransferase)
Q9UHG3	Prenylcysteine oxidase 1 (EC 1.8.3.5) (Prenylcysteine lyase)
Q8WUM4	Programmed cell death 6-interacting protein (PDCD6-interacting protein) (ALG-2-interacting protein 1) (ALG-2-interacting protein X) (Hp95)
P08559	Pyruvate dehydrogenase E1 component subunit alpha, somatic form, mitochondrial (EC 1.2.4.1) (PDHE1-A type I)
P11177	Pyruvate dehydrogenase E1 component subunit beta, mitochondrial (PDHE1-B) (EC 1.2.4.1)
P30101	Protein disulfide-isomerase A3 (EC 5.3.4.1) (58 kDa glucose-regulated protein) (58 kDa microsomal protein) (p58) (Disulfide isomerase ER-60) (Endoplasmic reticulum resident protein 57) (ER protein 57) (ERp57) (Endoplasmic reticulum resident protein 60) (ER protein 60) (ERp60)
P13667	Protein disulfide-isomerase A4 (EC 5.3.4.1) (Endoplasmic reticulum resident protein 70) (ER protein 70) (ERp70) (Endoplasmic reticulum resident protein 72) (ER protein 72) (ERp-72) (ERp72)
Q15084	Protein disulfide-isomerase A6 (EC 5.3.4.1) (Endoplasmic reticulum protein 5) (ER protein 5) (ERp5) (Protein disulfide isomerase P5) (Thioredoxin domain-containing protein 7)
Q96HC4	PDZ and LIM domain protein 5 (Enigma homolog) (Enigma-like PDZ and LIM domains protein)
Q6P996	Pyridoxal-dependent decarboxylase domain-containing protein 1 (EC 4.1.1.-)
Q15121	Astrocytic phosphoprotein PEA-15 (15 kDa phosphoprotein enriched in astrocytes) (Phosphoprotein enriched in diabetes) (PED)
P30086	Phosphatidylethanolamine-binding protein 1 (PEBP-1) (HCNPpp) (Neuropolypeptide h3) (Prostatic-binding protein) (Raf kinase inhibitor protein) (RKIP) [Cleaved into: Hippocampal cholinergic neurostimulating peptide (HCNP)]
P17858	ATP-dependent 6-phosphofructokinase, liver type (ATP-PFK) (PFK-L) (EC 2.7.1.11) (6-phosphofructokinase type B) (Phosphofructo-1-kinase isozyme B) (PFK-B) (Phosphohexokinase)
P07737	Profilin-1 (Epididymis tissue protein Li 184a) (Profilin I)
P52209	6-phosphogluconate dehydrogenase, decarboxylating (EC 1.1.1.44)

P00558	Phosphoglycerate kinase 1 (EC 2.7.2.3) (Cell migration-inducing gene 10 protein) (Primer recognition protein 2) (PRP 2)
P36871	Phosphoglucomutase-1 (PGM 1) (EC 5.4.2.2) (Glucose phosphomutase 1)
Q96G03	Phosphoglucomutase-2 (PGM 2) (EC 5.4.2.2) (Glucose phosphomutase 2) (Phosphodeoxyribomutase) (Phosphopentomutase) (EC 5.4.2.7)
P35232	Prohibitin
Q15149	Plectin (PCN) (PLTN) (Hemidesmosomal protein 1) (HD1) (Plectin-1)
P29590	Protein PML (Promyelocytic leukemia protein) (RING finger protein 71) (Tripartite motif-containing protein 19)
P01298	Pancreatic prohormone (Pancreatic polypeptide) (PP) (Obinepitide) [Cleaved into: Pancreatic hormone (PH); Pancreatic icosapeptide (PI)]
P16435	NADPH--cytochrome P450 reductase (CPR) (P450R) (EC 1.6.2.4)
Q15063	Periostin (PN) (Osteoblast-specific factor 2) (OSF-2)
Q15181	Inorganic pyrophosphatase (EC 3.6.1.1) (Pyrophosphate phospho-hydrolase) (PPase)
P23284	Peptidyl-prolyl cis-trans isomerase B (PPIase B) (EC 5.2.1.8) (CYP-S1) (Cyclophilin B) (Rotamase B) (S-cyclophilin) (SCYLP)
P62140	Serine/threonine-protein phosphatase PP1-beta catalytic subunit (PP-1B) (PPP1CD) (EC 3.1.3.16) (EC 3.1.3.53)
O14974	Protein phosphatase 1 regulatory subunit 12A (Myosin phosphatase-targeting subunit 1) (Myosin phosphatase target subunit 1) (Protein phosphatase myosin-binding subunit)
Q15435	Protein phosphatase 1 regulatory subunit 7 (Protein phosphatase 1 regulatory subunit 22)
P30153	Serine/threonine-protein phosphatase 2A 65 kDa regulatory subunit A alpha isoform (Medium tumor antigen-associated 61 kDa protein) (PP2A subunit A isoform PR65-alpha) (PP2A subunit A isoform R1-alpha)
Q06830	Peroxiredoxin-1 (EC 1.11.1.15) (Natural killer cell-enhancing factor A) (NKEF-A) (Proliferation-associated gene protein) (PAG) (Thioredoxin peroxidase 2) (Thioredoxin-dependent peroxide reductase 2)
P32119	Peroxiredoxin-2 (EC 1.11.1.15) (Natural killer cell-enhancing factor B) (NKEF-B) (PRP) (Thiol-specific antioxidant protein) (TSA) (Thioredoxin peroxidase 1) (Thioredoxin-dependent peroxide reductase 1)
P30048	Thioredoxin-dependent peroxide reductase, mitochondrial (EC 1.11.1.15) (Antioxidant protein 1) (AOP-1) (HBC189) (Peroxiredoxin III) (Prx-III) (Peroxiredoxin-3) (Protein MER5 homolog)
Q13162	Peroxiredoxin-4 (EC 1.11.1.15) (Antioxidant enzyme AOE372) (AOE37-2) (Peroxiredoxin IV) (Prx-IV) (Thioredoxin peroxidase AO372) (Thioredoxin-dependent peroxide reductase A0372)
P30044	Peroxiredoxin-5, mitochondrial (EC 1.11.1.15) (Alu corepressor 1) (Antioxidant enzyme B166) (AOEB166) (Liver tissue 2D-page spot 71B) (PLP) (Peroxiredoxin V) (Prx-V) (Peroxisomal antioxidant enzyme) (TPx type VI) (Thioredoxin peroxidase PMP20)
P30041	Peroxiredoxin-6 (EC 1.11.1.15) (1-Cys peroxiredoxin) (1-Cys PRX) (24 kDa protein) (Acidic calcium-independent phospholipase A2) (aiPLA2) (EC 3.1.1.4) (Antioxidant protein 2) (Liver 2D page spot 40) (Non-selenium glutathione peroxidase) (NSGPx) (Red blood cells page spot 12)
P51888	Prolargin (Proline-arginine-rich end leucine-rich repeat protein)
P10644	cAMP-dependent protein kinase type I-alpha regulatory subunit (Tissue-specific extinguisher 1) (TSE1)
P13861	cAMP-dependent protein kinase type II-alpha regulatory subunit
P14314	Glucosidase 2 subunit beta (80K-H protein) (Glucosidase II subunit beta) (Protein kinase C substrate 60.1 kDa protein heavy chain) (PKCSH)
P07477	Trypsin-1 (EC 3.4.21.4) (Beta-trypsin) (Cationic trypsinogen) (Serine protease 1) (Trypsin I) [Cleaved into: Alpha-trypsin chain 1; Alpha-trypsin chain 2]
P07602	Prosaposin (Proactivator polypeptide) [Cleaved into: Saposin-A (Protein A); Saposin-B-Val; Saposin-B (Cerebroside sulfate activator) (CSAct) (Dispersin) (Sphingolipid activator protein 1) (SAP-1) (Sulfatide/GM1 activator); Saposin-C (A1 activator) (Co-beta-glucosidase) (Glucosylceramidase activator) (Sphingolipid activator protein 2) (SAP-2); Saposin-D (Component C) (Protein C)]

P25786	Proteasome subunit alpha type-1 (EC 3.4.25.1) (30 kDa prosomal protein) (PROS-30) (Macropain subunit C2) (Multicatalytic endopeptidase complex subunit C2) (Proteasome component C2) (Proteasome nu chain)
P25788	Proteasome subunit alpha type-3 (EC 3.4.25.1) (Macropain subunit C8) (Multicatalytic endopeptidase complex subunit C8) (Proteasome component C8)
P28066	Proteasome subunit alpha type-5 (EC 3.4.25.1) (Macropain zeta chain) (Multicatalytic endopeptidase complex zeta chain) (Proteasome zeta chain)
O14818	Proteasome subunit alpha type-7 (EC 3.4.25.1) (Proteasome subunit RC6-1) (Proteasome subunit XAPC7)
P28072	Proteasome subunit beta type-6 (EC 3.4.25.1) (Macropain delta chain) (Multicatalytic endopeptidase complex delta chain) (Proteasome delta chain) (Proteasome subunit Y)
P28062	Proteasome subunit beta type-8 (EC 3.4.25.1) (Low molecular mass protein 7) (Macropain subunit C13) (Multicatalytic endopeptidase complex subunit C13) (Proteasome component C13) (Proteasome subunit beta-5i) (Really interesting new gene 10 protein)
P28065	Proteasome subunit beta type-9 (EC 3.4.25.1) (Low molecular mass protein 2) (Macropain chain 7) (Multicatalytic endopeptidase complex chain 7) (Proteasome chain 7) (Proteasome subunit beta-1i) (Really interesting new gene 12 protein)
P62191	26S proteasome regulatory subunit 4 (P26s4) (26S proteasome AAA-ATPase subunit RPT2) (Proteasome 26S subunit ATPase 1)
P35998	26S proteasome regulatory subunit 7 (26S proteasome AAA-ATPase subunit RPT1) (Proteasome 26S subunit ATPase 2) (Protein MSS1)
P17980	26S proteasome regulatory subunit 6A (26S proteasome AAA-ATPase subunit RPT5) (Proteasome 26S subunit ATPase 3) (Proteasome subunit P50) (Tat-binding protein 1) (TBP-1)
P62195	26S proteasome regulatory subunit 8 (26S proteasome AAA-ATPase subunit RPT6) (Proteasome 26S subunit ATPase 5) (Proteasome subunit p45) (Thyroid hormone receptor-interacting protein 1) (TRIP1) (p45/SUG)
O00231	26S proteasome non-ATPase regulatory subunit 11 (26S proteasome regulatory subunit RPN6) (26S proteasome regulatory subunit S9) (26S proteasome regulatory subunit p44.5)
Q13200	26S proteasome non-ATPase regulatory subunit 2 (26S proteasome regulatory subunit RPN1) (26S proteasome regulatory subunit S2) (26S proteasome subunit p97) (Protein 55.11) (Tumor necrosis factor type 1 receptor-associated protein 2)
O43242	26S proteasome non-ATPase regulatory subunit 3 (26S proteasome regulatory subunit RPN3) (26S proteasome regulatory subunit S3) (Proteasome subunit p58)
Q15008	26S proteasome non-ATPase regulatory subunit 6 (26S proteasome regulatory subunit RPN7) (26S proteasome regulatory subunit S10) (Breast cancer-associated protein SGA-113M) (Phosphonoformate immuno-associated protein 4) (Proteasome regulatory particle subunit p44S10) (p42A)
Q06323	Proteasome activator complex subunit 1 (11S regulator complex subunit alpha) (REG-alpha) (Activator of multicatalytic protease subunit 1) (Interferon gamma up-regulated I-5111 protein) (IGUP I-5111) (Proteasome activator 28 subunit alpha) (PA28a) (PA28alpha)
Q9UL46	Proteasome activator complex subunit 2 (11S regulator complex subunit beta) (REG-beta) (Activator of multicatalytic protease subunit 2) (Proteasome activator 28 subunit beta) (PA28b) (PA28beta)
P26599	Polypyrimidine tract-binding protein 1 (PTB) (57 kDa RNA-binding protein PPTB-1) (Heterogeneous nuclear ribonucleoprotein I) (hnRNP I)
P29350	Tyrosine-protein phosphatase non-receptor type 6 (EC 3.1.3.48) (Hematopoietic cell protein-tyrosine phosphatase) (Protein-tyrosine phosphatase 1C) (PTP-1C) (Protein-tyrosine phosphatase SHP-1) (SH-PTP1)
P11216	Glycogen phosphorylase, brain form (EC 2.4.1.1)
P61026	Ras-related protein Rab-10
Q15907	Ras-related protein Rab-11B (GTP-binding protein YPT3)

Q9H0U4	Ras-related protein Rab-1B
P61019	Ras-related protein Rab-2A
P51149	Ras-related protein Rab-7a
Q9UKM9	RNA-binding protein Raly (Autoantigen p542) (Heterogeneous nuclear ribonucleoprotein C-like 2) (hnRNP core protein C-like 2) (hnRNP associated with lethal yellow protein homolog)
P61224	Ras-related protein Rap-1b (GTP-binding protein smg p21B)
P38159	RNA-binding motif protein, X chromosome (Glycoprotein p43) (Heterogeneous nuclear ribonucleoprotein G) (hnRNP G) [Cleaved into: RNA-binding motif protein, X chromosome, N-terminally processed]
Q15293	Reticulocalbin-1
Q9H4A4	Aminopeptidase B (AP-B) (EC 3.4.11.6) (Arginine aminopeptidase) (Arginyl aminopeptidase)
P27694	Replication protein A 70 kDa DNA-binding subunit (RP-A p70) (Replication factor A protein 1) (RF-A protein 1) (Single-stranded DNA-binding protein) [Cleaved into: Replication protein A 70 kDa DNA-binding subunit, N-terminally processed]
P27635	60S ribosomal protein L10 (Laminin receptor homolog) (Large ribosomal subunit protein uL16) (Protein QM) (Ribosomal protein L10) (Tumor suppressor QM)
P62906	60S ribosomal protein L10a (CSA-19) (Large ribosomal subunit protein uL1) (Neural precursor cell expressed developmentally down-regulated protein 6) (NEDD-6)
Q07020	60S ribosomal protein L18 (Large ribosomal subunit protein eL18)
P46779	60S ribosomal protein L28 (Large ribosomal subunit protein eL28)
P36578	60S ribosomal protein L4 (60S ribosomal protein L1) (Large ribosomal subunit protein uL4)
P05388	60S acidic ribosomal protein P0 (60S ribosomal protein L10E) (Large ribosomal subunit protein uL10)
P05387	60S acidic ribosomal protein P2 (Large ribosomal subunit protein P2) (Renal carcinoma antigen NY-REN-44)
P04843	Dolichyl-diphosphooligosaccharide--protein glycosyltransferase subunit 1 (Dolichyl-diphosphooligosaccharide--protein glycosyltransferase 67 kDa subunit) (Ribophorin I) (RPN-I) (Ribophorin-1)
P25398	40S ribosomal protein S12 (Small ribosomal subunit protein eS12)
P15880	40S ribosomal protein S2 (40S ribosomal protein S4) (Protein LLRep3) (Small ribosomal subunit protein uS5)
P62857	40S ribosomal protein S28 (Small ribosomal subunit protein eS28)
P23396	40S ribosomal protein S3 (EC 4.2.99.18) (Small ribosomal subunit protein uS3)
P61247	40S ribosomal protein S3a (Small ribosomal subunit protein eS1) (v-fos transformation effector protein) (Fte-1)
P62701	40S ribosomal protein S4, X isoform (SCR10) (Single copy abundant mRNA protein) (Small ribosomal subunit protein eS4)
P62241	40S ribosomal protein S8 (Small ribosomal subunit protein eS8)
P46781	40S ribosomal protein S9 (Small ribosomal subunit protein uS4)
Q9NQC3	Reticulon-4 (Foocen) (Neurite outgrowth inhibitor) (Nogo protein) (Neuroendocrine-specific protein) (NSP) (Neuroendocrine-specific protein C homolog) (RTN-x) (Reticulon-5)
Q9Y230	RuvB-like 2 (EC 3.6.4.12) (48 kDa TATA box-binding protein-interacting protein) (48 kDa TBP-interacting protein) (51 kDa erythrocyte cytosolic protein) (ECP-51) (INO80 complex subunit J) (Repressing pontin 52) (Reptin 52) (TIP49b) (TIP60-associated protein 54-beta) (TAP54-beta)
P60903	Protein S100-A10 (Calpactin I light chain) (Calpactin-1 light chain) (Cellular ligand of annexin II) (S100 calcium-binding protein A10) (p10 protein) (p11)

P31949	Protein S100-A11 (Calgizzarin) (Metastatic lymph node gene 70 protein) (MLN 70) (Protein S100-C) (S100 calcium-binding protein A11) [Cleaved into: Protein S100-A11, N-terminally processed]
P26447	Protein S100-A4 (Calvasculin) (Metastasin) (Placental calcium-binding protein) (Protein Mts1) (S100 calcium-binding protein A4)
P05109	Protein S100-A8 (Calgranulin-A) (Calprotectin L1L subunit) (Cystic fibrosis antigen) (CFAG) (Leukocyte L1 complex light chain) (Migration inhibitory factor-related protein 8) (MRP-8) (p8) (S100 calcium-binding protein A8) (Urinary stone protein band A)
P06702	Protein S100-A9 (Calgranulin-B) (Calprotectin L1H subunit) (Leukocyte L1 complex heavy chain) (Migration inhibitory factor-related protein 14) (MRP-14) (p14) (S100 calcium-binding protein A9)
P25815	Protein S100-P (Migration-inducing gene 9 protein) (MIG9) (Protein S100-E) (S100 calcium-binding protein P)
O75396	Vesicle-trafficking protein SEC22b (ER-Golgi SNARE of 24 kDa) (ERS-24) (ERS24) (SEC22 vesicle-trafficking protein homolog B) (SEC22 vesicle-trafficking protein-like 1)
O94979	Protein transport protein Sec31A (ABP125) (ABP130) (SEC31-like protein 1) (SEC31-related protein A) (Web1-like protein)
Q13228	Methanethiol oxidase (MTO) (EC 1.8.3.4) (56 kDa selenium-binding protein) (SBP56) (SP56) (Selenium-binding protein 1)
Q15019	Septin-2 (Neural precursor cell expressed developmentally down-regulated protein 5) (NEDD-5)
Q16181	Septin-7 (CDC10 protein homolog)
Q9UHD8	Septin-9 (MLL septin-like fusion protein MSF-A) (MLL septin-like fusion protein) (Ovarian/Breast septin) (Ov/Br septin) (Septin D1)
Q8NC51	Plasminogen activator inhibitor 1 RNA-binding protein (PAI1 RNA-binding protein 1) (PAI-RBP1) (SERPINE1 mRNA-binding protein 1)
P01009	Alpha-1-antitrypsin (Alpha-1 protease inhibitor) (Alpha-1-antiproteinase) (Serpin A1) [Cleaved into: Short peptide from AAT (SPAAT)]
P01011	Alpha-1-antichymotrypsin (ACT) (Cell growth-inhibiting gene 24/25 protein) (Serpin A3) [Cleaved into: Alpha-1-antichymotrypsin His-Pro-less]
P30740	Leukocyte elastase inhibitor (LEI) (Monocyte/neutrophil elastase inhibitor) (EI) (M/NEI) (Peptidase inhibitor 2) (PI-2) (Serpin B1)
P50454	Serpin H1 (47 kDa heat shock protein) (Arsenic-transactivated protein 3) (AsTP3) (Cell proliferation-inducing gene 14 protein) (Collagen-binding protein) (Colligin) (Rheumatoid arthritis-related antigen RA-A47)
Q01105	Protein SET (HLA-DR-associated protein II) (Inhibitor of granzyme A-activated DNase) (IGAAD) (PHAPII) (Phosphatase 2A inhibitor I2PP2A) (I-2PP2A) (Template-activating factor I) (TAF-I)
Q15459	Splicing factor 3A subunit 1 (SF3a120) (Spliceosome-associated protein 114) (SAP 114)
P31947	14-3-3 protein sigma (Epithelial cell marker protein 1) (Stratifin)
P23246	Splicing factor, proline- and glutamine-rich (100 kDa DNA-pairing protein) (hPOMp100) (DNA-binding p52/p100 complex, 100 kDa subunit) (Polypyrimidine tract-binding protein-associated-splicing factor) (PSF) (PTB-associated-splicing factor)
O75368	SH3 domain-binding glutamic acid-rich-like protein
Q6NUK1	Calcium-binding mitochondrial carrier protein SCaMC-1 (Mitochondrial ATP-Mg/Pi carrier protein 1) (Mitochondrial Ca(2+)-dependent solute carrier protein 1) (Small calcium-binding mitochondrial carrier protein 1) (Solute carrier family 25 member 24)
P11166	Solute carrier family 2, facilitated glucose transporter member 1 (Glucose transporter type 1, erythrocyte/brain) (GLUT-1) (HepG2 glucose transporter)
O14745	Na(+)/H(+) exchange regulatory cofactor NHE-RF1 (NHERF-1) (Ezrin-radixin-moesin-binding phosphoprotein 50) (EBP50) (Regulatory cofactor of Na(+)/H(+) exchanger) (Sodium-hydrogen exchanger regulatory factor 1) (Solute carrier family 9 isoform A3 regulatory factor 1)
O76070	Gamma-synuclein (Breast cancer-specific gene 1 protein) (Persyn) (Synoretin) (SR)
Q7KZF4	Staphylococcal nuclease domain-containing protein 1 (EC 3.1.31.1) (100 kDa coactivator) (EBNA2 coactivator p100) (Tudor domain-containing protein 11) (p100 co-activator)

O75643	U5 small nuclear ribonucleoprotein 200 kDa helicase (EC 3.6.4.13) (Activating signal cointegrator 1 complex subunit 3-like 1) (BRR2 homolog) (U5 snRNP-specific 200 kDa protein) (U5-200KD)
Q9BX66	Sorbin and SH3 domain-containing protein 1 (Ponsin) (SH3 domain protein 5) (SH3P12) (c-Cbl-associated protein) (CAP)
Q00796	Sorbitol dehydrogenase (SDH) (EC 1.1.1.-) ((R,R)-butanediol dehydrogenase) (EC 1.1.1.4) (L-iditol 2-dehydrogenase) (EC 1.1.1.14) (Polyol dehydrogenase) (Ribitol dehydrogenase) (RDH) (EC 1.1.1.56) (Xylitol dehydrogenase) (XDH) (EC 1.1.1.9)
Q13813	Spectrin alpha chain, non-erythrocytic 1 (Alpha-II spectrin) (Fodrin alpha chain) (Spectrin, non-erythroid alpha subunit)
Q01082	Spectrin beta chain, non-erythrocytic 1 (Beta-II spectrin) (Fodrin beta chain) (Spectrin, non-erythroid beta chain 1)
P30626	Sorcin (22 kDa protein) (CP-22) (CP22) (V19)
P05455	Lupus La protein (La autoantigen) (La ribonucleoprotein) (Sjoegren syndrome type B antigen) (SS-B)
P42224	Signal transducer and activator of transcription 1-alpha/beta (Transcription factor ISGF-3 components p91/p84)
P27105	Erythrocyte band 7 integral membrane protein (Protein 7.2b) (Stomatin)
P53999	Activated RNA polymerase II transcriptional coactivator p15 (Positive cofactor 4) (PC4) (SUB1 homolog) (p14)
O60506	Heterogeneous nuclear ribonucleoprotein Q (hnRNP Q) (Glycine- and tyrosine-rich RNA-binding protein) (GRY-RBP) (NS1-associated protein 1) (Synaptotagmin-binding, cytoplasmic RNA-interacting protein)
Q01995	Transgelin (22 kDa actin-binding protein) (Protein WS3-10) (Smooth muscle protein 22-alpha) (SM22-alpha)
P37802	Transgelin-2 (Epididymis tissue protein Li 7e) (SM22-alpha homolog)
P37837	Transaldolase (EC 2.2.1.2)
P17987	T-complex protein 1 subunit alpha (TCP-1-alpha) (CCT-alpha)
P02787	Serotransferrin (Transferrin) (Beta-1 metal-binding globulin) (Siderophilin)
Q15582	Transforming growth factor-beta-induced protein ig-h3 (Beta ig-h3) (Kerato-epithelin) (RGD-containing collagen-associated protein) (RGD-CAP)
P21980	Protein-glutamine gamma-glutamyltransferase 2 (EC 2.3.2.13) (Tissue transglutaminase) (Transglutaminase C) (TG(C)) (TGC) (TGase C) (Transglutaminase H) (TGase H) (Transglutaminase-2) (TGase-2)
P07996	Thrombospondin-1 (Glycoprotein G)
Q07157	Tight junction protein ZO-1 (Tight junction protein 1) (Zona occludens protein 1) (Zonula occludens protein 1)
Q9UDY2	Tight junction protein ZO-2 (Tight junction protein 2) (Zona occludens protein 2) (Zonula occludens protein 2)
P29401	Transketolase (TK) (EC 2.2.1.1)
Q9Y490	Talin-1
Q15363	Transmembrane emp24 domain-containing protein 2 (Membrane protein p24A) (p24) (p24 family protein beta-1) (p24beta1)
Q9NYL9	Tropomodulin-3 (Ubiquitous tropomodulin) (U-Tmod)
P62328	Thymosin beta-4 (T beta-4) (Fx) [Cleaved into: Hematopoietic system regulatory peptide (Seraspenide)]
P24821	Tenascin (TN) (Cytotactin) (GMEM) (GP 150-225) (Glioma-associated-extracellular matrix antigen) (Hexabrachion) (JI) (Myotendinous antigen) (Neuronectin) (Tenascin-C) (TN-C)
Q9C0C2	182 kDa tankyrase-1-binding protein
Q9HBL0	Tensin-1
P22105	Tenascin-X (TN-X) (Hexabrachion-like protein)
P09493	Tropomyosin alpha-1 chain (Alpha-tropomyosin) (Tropomyosin-1)
P07951	Tropomyosin beta chain (Beta-tropomyosin) (Tropomyosin-2)

P06753	Tropomyosin alpha-3 chain (Gamma-tropomyosin) (Tropomyosin-3) (Tropomyosin-5) (hTM5)
P67936	Tropomyosin alpha-4 chain (TM30p1) (Tropomyosin-4)
P29144	Tripeptidyl-peptidase 2 (TPP-2) (EC 3.4.14.10) (Tripeptidyl aminopeptidase) (Tripeptidyl-peptidase II) (TPP-II)
P12270	Nucleoprotein TPR (Megator) (NPC-associated intranuclear protein) (Translocated promoter region protein)
Q12931	Heat shock protein 75 kDa, mitochondrial (HSP 75) (TNFR-associated protein 1) (Tumor necrosis factor type 1 receptor-associated protein) (TRAP-1)
Q13263	Transcription intermediary factor 1-beta (TIF1-beta) (E3 SUMO-protein ligase TRIM28) (EC 2.3.2.27) (KRAB-associated protein 1) (KAP-1) (KRAB-interacting protein 1) (KRIP-1) (Nuclear corepressor KAP-1) (RING finger protein 96) (RING-type E3 ubiquitin transferase TIF1-beta) (Tripartite motif-containing protein 28)
Q15631	Translin (EC 3.1.-.-) (Component 3 of promoter of RISC) (C3PO)
Q16762	Thiosulfate sulfurtransferase (EC 2.8.1.1) (Rhodanese)
Q13630	GDP-L-fucose synthase (EC 1.1.1.271) (GDP-4-keto-6-deoxy-D-mannose-3,5-epimerase-4-reductase) (Protein FX) (Red cell NADP(H)-binding protein) (Short-chain dehydrogenase/reductase family 4E member 1)
P68366	Tubulin alpha-4A chain (Alpha-tubulin 1) (Testis-specific alpha-tubulin) (Tubulin H2-alpha) (Tubulin alpha-1 chain)
P07437	Tubulin beta chain (Tubulin beta-5 chain)
Q13885	Tubulin beta-2A chain (Tubulin beta class IIa)
P49411	Elongation factor Tu, mitochondrial (EF-Tu) (P43)
Q8NBS9	Thioredoxin domain-containing protein 5 (Endoplasmic reticulum resident protein 46) (ER protein 46) (ERp46) (Thioredoxin-like protein p46)
O43396	Thioredoxin-like protein 1 (32 kDa thioredoxin-related protein)
Q16881	Thioredoxin reductase 1, cytoplasmic (TR) (EC 1.8.1.9) (Gene associated with retinoic and interferon-induced mortality 12 protein) (GRIM-12) (Gene associated with retinoic and IFN-induced mortality 12 protein) (KM-102-derived reductase-like factor) (Thioredoxin reductase TR1)
P19971	Thymidine phosphorylase (TP) (EC 2.4.2.4) (Gliostatin) (Platelet-derived endothelial cell growth factor) (PD-ECGF) (TdRPase)
P22314	Ubiquitin-like modifier-activating enzyme 1 (EC 6.2.1.45) (Protein A1S9) (Ubiquitin-activating enzyme E1)
Q5T4S7	E3 ubiquitin-protein ligase UBR4 (EC 2.3.2.27) (600 kDa retinoblastoma protein-associated factor) (N-recognin-4) (RING-type E3 ubiquitin transferase UBR4) (Retinoblastoma-associated factor of 600 kDa) (RBAF600) (p600) (Zinc finger UBR1-type protein 1)
P15374	Ubiquitin carboxyl-terminal hydrolase isozyme L3 (UCH-L3) (EC 3.4.19.12) (Ubiquitin thioesterase L3)
Q16851	UTP--glucose-1-phosphate uridylyltransferase (EC 2.7.7.9) (UDP-glucose pyrophosphorylase) (UDPGP) (UGPase)
O60763	General vesicular transport factor p115 (Protein USO1 homolog) (Transcytosis-associated protein) (TAP) (Vesicle-docking protein)
P54578	Ubiquitin carboxyl-terminal hydrolase 14 (EC 3.4.19.12) (Deubiquitinating enzyme 14) (Ubiquitin thioesterase 14) (Ubiquitin-specific-processing protease 14)
Q9P0L0	Vesicle-associated membrane protein-associated protein A (VAMP-A) (VAMP-associated protein A) (VAP-A) (33 kDa VAMP-associated protein) (VAP-33)
Q99536	Synaptic vesicle membrane protein VAT-1 homolog (EC 1.-.-.-)
P13611	Versican core protein (Chondroitin sulfate proteoglycan core protein 2) (Chondroitin sulfate proteoglycan 2) (Glial hyaluronate-binding protein) (GHAP) (Large fibroblast proteoglycan) (PG-M)
P18206	Vinculin (Metavinculin) (MV)
P55072	Transitional endoplasmic reticulum ATPase (TER ATPase) (EC 3.6.4.6) (15S Mg(2+)-ATPase p97 subunit) (Valosin-containing protein) (VCP)

P21796	Voltage-dependent anion-selective channel protein 1 (VDAC-1) (hVDAC1) (Outer mitochondrial membrane protein porin 1) (Plasmalemmal porin) (Porin 31HL) (Porin 31HM)
P08670	Vimentin
O75436	Vacuolar protein sorting-associated protein 26A (Vesicle protein sorting 26A) (hVPS26)
Q96QK1	Vacuolar protein sorting-associated protein 35 (hVPS35) (Maternal-embryonic 3) (Vesicle protein sorting 35)
P04004	Vitronectin (VN) (S-protein) (Serum-spreading factor) (V75) [Cleaved into: Vitronectin V65 subunit; Vitronectin V10 subunit; Somatomedin-B]
O14980	Exportin-1 (Exp1) (Chromosome region maintenance 1 protein homolog)
P13010	X-ray repair cross-complementing protein 5 (EC 3.6.4.-) (86 kDa subunit of Ku antigen) (ATP-dependent DNA helicase 2 subunit 2) (ATP-dependent DNA helicase II 80 kDa subunit) (CTC box-binding factor 85 kDa subunit) (CTC85) (CTCBF) (DNA repair protein XRCC5) (Ku80) (Ku86) (Lupus Ku autoantigen protein p86) (Nuclear factor IV) (Thyroid-lupus autoantigen) (TLAA) (X-ray repair complementing defective repair in Chinese hamster cells 5 (double-strand-break rejoining))
P12956	X-ray repair cross-complementing protein 6 (EC 3.6.4.-) (EC 4.2.99.-) (5'-deoxyribose-5-phosphate lyase Ku70) (5'-dRP lyase Ku70) (70 kDa subunit of Ku antigen) (ATP-dependent DNA helicase 2 subunit 1) (ATP-dependent DNA helicase II 70 kDa subunit) (CTC box-binding factor 75 kDa subunit) (CTC75) (CTCBF) (DNA repair protein XRCC6) (Lupus Ku autoantigen protein p70) (Ku70) (Thyroid-lupus autoantigen) (TLAA) (X-ray repair complementing defective repair in Chinese hamster cells 6)
P67809	Y-box-binding protein 1 (YB-1) (CCAAT-binding transcription factor I subunit A) (CBF-A) (DNA-binding protein B) (DBPB) (Enhancer factor I subunit A) (EFI-A) (Nuclease-sensitive element-binding protein 1) (Y-box transcription factor)
P31946	14-3-3 protein beta/alpha (Protein 1054) (Protein kinase C inhibitor protein 1) (KCIP-1) [Cleaved into: 14-3-3 protein beta/alpha, N-terminally processed]
P62258	14-3-3 protein epsilon (14-3-3E)
P61981	14-3-3 protein gamma (Protein kinase C inhibitor protein 1) (KCIP-1) [Cleaved into: 14-3-3 protein gamma, N-terminally processed]
Q04917	14-3-3 protein eta (Protein AS1)
P27348	14-3-3 protein theta (14-3-3 protein T-cell) (14-3-3 protein tau) (Protein HS1)
P63104	14-3-3 protein zeta/delta (Protein kinase C inhibitor protein 1) (KCIP-1)

Uniprot ID	Protein names
P61604	10 kDa heat shock protein, mitochondrial (Hsp10) (10 kDa chaperonin) (Chaperonin 10) (CPN10) (Early-pregnancy factor) (EPF)
P31946	14-3-3 protein beta/alpha (Protein 1054) (Protein kinase C inhibitor protein 1) (KCIP-1) [Cleaved into: 14-3-3 protein beta/alpha, N-terminally processed]
P62258	14-3-3 protein epsilon (14-3-3E)
Q04917	14-3-3 protein eta (Protein AS1)
P61981	14-3-3 protein gamma (Protein kinase C inhibitor protein 1) (KCIP-1) [Cleaved into: 14-3-3 protein gamma, N-terminally processed]
P31947	14-3-3 protein sigma (Epithelial cell marker protein 1) (Stratifin)
P27348	14-3-3 protein theta (14-3-3 protein T-cell) (14-3-3 protein tau) (Protein HS1)
P63104	14-3-3 protein zeta/delta (Protein kinase C inhibitor protein 1) (KCIP-1)
Q9C0C2	182 kDa tankyrase-1-binding protein
Q16698	2,4-dienoyl-CoA reductase, mitochondrial (EC 1.3.1.34) (2,4-dienoyl-CoA reductase [NADPH]) (4-enoyl-CoA reductase [NADPH]) (Short chain dehydrogenase/reductase family 18C member 1)
P09543	2',3'-cyclic-nucleotide 3'-phosphodiesterase (CNP) (CNPase) (EC 3.1.4.37)
Q99460	26S proteasome non-ATPase regulatory subunit 1 (26S proteasome regulatory subunit RPN2) (26S proteasome regulatory subunit S1) (26S proteasome subunit p112)
O00231	26S proteasome non-ATPase regulatory subunit 11 (26S proteasome regulatory subunit RPN6) (26S proteasome regulatory subunit S9) (26S proteasome regulatory subunit p44.5)
O00232	26S proteasome non-ATPase regulatory subunit 12 (26S proteasome regulatory subunit RPN5) (26S proteasome regulatory subunit p55)
Q13200	26S proteasome non-ATPase regulatory subunit 2 (26S proteasome regulatory subunit RPN1) (26S proteasome regulatory subunit S2) (26S proteasome subunit p97) (Protein 55.11) (Tumor necrosis factor type 1 receptor-associated protein 2)
O43242	26S proteasome non-ATPase regulatory subunit 3 (26S proteasome regulatory subunit RPN3) (26S proteasome regulatory subunit S3) (Proteasome subunit p58)
Q15008	26S proteasome non-ATPase regulatory subunit 6 (26S proteasome regulatory subunit RPN7) (26S proteasome regulatory subunit S10) (Breast cancer-associated protein SGA-113M) (Phosphonoformate immuno-associated protein 4) (Proteasome regulatory particle subunit p44S10) (p42A)
O00233	26S proteasome non-ATPase regulatory subunit 9 (26S proteasome regulatory subunit p27)
P62333	26S proteasome regulatory subunit 10B (26S proteasome AAA-ATPase subunit RPT4) (Proteasome 26S subunit ATPase 6) (Proteasome subunit p42)

P62191	26S proteasome regulatory subunit 4 (P26s4) (26S proteasome AAA-ATPase subunit RPT2) (Proteasome 26S subunit ATPase 1)
P17980	26S proteasome regulatory subunit 6A (26S proteasome AAA-ATPase subunit RPT5) (Proteasome 26S subunit ATPase 3) (Proteasome subunit P50) (Tat-binding protein 1) (TBP-1)
P43686	26S proteasome regulatory subunit 6B (26S proteasome AAA-ATPase subunit RPT3) (MB67-interacting protein) (MIP224) (Proteasome 26S subunit ATPase 4) (Tat-binding protein 7) (TBP-7)
P35998	26S proteasome regulatory subunit 7 (26S proteasome AAA-ATPase subunit RPT1) (Proteasome 26S subunit ATPase 2) (Protein MSS1)
P62195	26S proteasome regulatory subunit 8 (26S proteasome AAA-ATPase subunit RPT6) (Proteasome 26S subunit ATPase 5) (Proteasome subunit p45) (Thyroid hormone receptor-interacting protein 1) (TRIP1) (p45/SUG)
Q13442	28 kDa heat- and acid-stable phosphoprotein (PDGF-associated protein) (PAP) (PDGFA-associated protein 1) (PAP1)
P31937	3-hydroxyisobutyrate dehydrogenase, mitochondrial (HIBADH) (EC 1.1.1.31)
Q6NVY1	3-hydroxyisobutyryl-CoA hydrolase, mitochondrial (EC 3.1.2.4) (3-hydroxyisobutyryl-coenzyme A hydrolase) (HIB-CoA hydrolase) (HIBYL-CoA-H)
P42765	3-ketoacyl-CoA thiolase, mitochondrial (EC 2.3.1.16) (Acetyl-CoA acyltransferase) (Beta-ketothiolase) (Mitochondrial 3-oxoacyl-CoA thiolase) (T1)
P25325	3-mercaptopyruvate sulfurtransferase (MST) (EC 2.8.1.2)
P49189	4-trimethylaminobutyraldehyde dehydrogenase (TMABADH) (EC 1.2.1.47) (Aldehyde dehydrogenase E3 isozyme) (Aldehyde dehydrogenase family 9 member A1) (EC 1.2.1.3) (Gamma-aminobutyraldehyde dehydrogenase) (EC 1.2.1.19) (R-aminobutyraldehyde dehydrogenase) [Cleaved into: 4-trimethylaminobutyraldehyde dehydrogenase, N-terminally processed]
P62280	40S ribosomal protein S11 (Small ribosomal subunit protein uS17)
P25398	40S ribosomal protein S12 (Small ribosomal subunit protein eS12)
P15880	40S ribosomal protein S2 (40S ribosomal protein S4) (Protein LLRep3) (Small ribosomal subunit protein uS5)
P62857	40S ribosomal protein S28 (Small ribosomal subunit protein eS28)
P23396	40S ribosomal protein S3 (EC 4.2.99.18) (Small ribosomal subunit protein uS3)
P61247	40S ribosomal protein S3a (Small ribosomal subunit protein eS1) (v-fos transformation effector protein) (Fte-1)
P62701	40S ribosomal protein S4, X isoform (SCR10) (Single copy abundant mRNA protein) (Small ribosomal subunit protein eS4)
P46782	40S ribosomal protein S5 (Small ribosomal subunit protein uS7) [Cleaved into: 40S ribosomal protein S5, N-terminally processed]

P62241	40S ribosomal protein S8 (Small ribosomal subunit protein eS8)
P46781	40S ribosomal protein S9 (Small ribosomal subunit protein uS4)
P08865	40S ribosomal protein SA (37 kDa laminin receptor precursor) (37LRP) (37/67 kDa laminin receptor) (LRP/LR) (67 kDa laminin receptor) (67LR) (Colon carcinoma laminin-binding protein) (Laminin receptor 1) (LamR) (Laminin-binding protein precursor p40) (LBP/p40) (Multidrug resistance-associated protein MGr1-Ag) (NEM/1CHD4) (Small ribosomal subunit protein uS2)
P52209	6-phosphogluconate dehydrogenase, decarboxylating (EC 1.1.1.44)
O95336	6-phosphogluconolactonase (6PGL) (EC 3.1.1.31)
P10809	60 kDa heat shock protein, mitochondrial (EC 3.6.4.9) (60 kDa chaperonin) (Chaperonin 60) (CPN60) (Heat shock protein 60) (HSP-60) (Hsp60) (HuCHA60) (Mitochondrial matrix protein P1) (P60 lymphocyte protein)
P10155	60 kDa SS-A/Ro ribonucleoprotein (60 kDa Ro protein) (60 kDa ribonucleoprotein Ro) (RoRNP) (Ro 60 kDa autoantigen) (Sjoegren syndrome antigen A2) (Sjoegren syndrome type A antigen) (SS-A) (TROVE domain family member 2)
P05388	60S acidic ribosomal protein P0 (60S ribosomal protein L10E) (Large ribosomal subunit protein uL10)
P05387	60S acidic ribosomal protein P2 (Large ribosomal subunit protein P2) (Renal carcinoma antigen NY-REN-44)
P27635	60S ribosomal protein L10 (Laminin receptor homolog) (Large ribosomal subunit protein uL16) (Protein QM) (Tumor suppressor QM)
P62906	60S ribosomal protein L10a (CSA-19) (Large ribosomal subunit protein uL1) (Neural precursor cell expressed developmentally down-regulated protein 6) (NEDD-6)
P18621	60S ribosomal protein L17 (60S ribosomal protein L23) (Large ribosomal subunit protein uL22) (PD-1)
Q07020	60S ribosomal protein L18 (Large ribosomal subunit protein eL18)
Q02543	60S ribosomal protein L18a (Large ribosomal subunit protein eL20)
P84098	60S ribosomal protein L19 (Large ribosomal subunit protein eL19)
P83731	60S ribosomal protein L24 (60S ribosomal protein L30) (Large ribosomal subunit protein eL24)
P36578	60S ribosomal protein L4 (60S ribosomal protein L1) (Large ribosomal subunit protein uL4)
P18124	60S ribosomal protein L7 (Large ribosomal subunit protein uL30)
P11021	78 kDa glucose-regulated protein (GRP-78) (Endoplasmic reticulum lumenal Ca(2+)-binding protein grp78) (Heat shock 70 kDa protein 5) (Immunoglobulin heavy chain-binding protein) (BiP)
Q9BWD1	Acetyl-CoA acetyltransferase, cytosolic (EC 2.3.1.9) (Acetyl-CoA transferase-like protein) (Cytosolic acetoacetyl-CoA thiolase)
P24752	Acetyl-CoA acetyltransferase, mitochondrial (EC 2.3.1.9) (Acetoacetyl-CoA thiolase) (T2)

Q13510	Acid ceramidase (AC) (ACDase) (Acid CDase) (EC 3.5.1.23) (Acylsphingosine deacylase) (N-acylsphingosine amidohydrolase) (Putative 32 kDa heart protein) (PHP32) [Cleaved into: Acid ceramidase subunit alpha; Acid ceramidase subunit beta]
P39687	Acidic leucine-rich nuclear phosphoprotein 32 family member A (Acidic nuclear phosphoprotein pp32) (pp32) (Leucine-rich acidic nuclear protein) (LANP) (Mapmodulin) (Potent heat-stable protein phosphatase 2A inhibitor I1PP2A) (Putative HLA-DR-associated protein I) (PHAPI)
Q99798	Aconitate hydratase, mitochondrial (Aconitase) (EC 4.2.1.3) (Citrate hydro-lyase)
P61160	Actin-related protein 2 (Actin-like protein 2)
O15143	Actin-related protein 2/3 complex subunit 1B (Arp2/3 complex 41 kDa subunit) (p41-ARC)
O15145	Actin-related protein 2/3 complex subunit 3 (Arp2/3 complex 21 kDa subunit) (p21-ARC)
P59998	Actin-related protein 2/3 complex subunit 4 (Arp2/3 complex 20 kDa subunit) (p20-ARC)
O15511	Actin-related protein 2/3 complex subunit 5 (Arp2/3 complex 16 kDa subunit) (p16-ARC)
P61158	Actin-related protein 3 (Actin-like protein 3)
P68032	Actin, alpha cardiac muscle 1 (Alpha-cardiac actin)
P53999	Activated RNA polymerase II transcriptional coactivator p15 (Positive cofactor 4) (PC4) (SUB1 homolog) (p14)
P07108	Acyl-CoA-binding protein (ACBP) (Diazepam-binding inhibitor) (DBI) (Endozepine) (EP)
P46108	Adapter molecule crk (Proto-oncogene c-Crk) (p38)
P23526	Adenosylhomocysteinase (AdoHcyase) (EC 3.3.1.1) (S-adenosyl-L-homocysteine hydrolase)
P54819	Adenylate kinase 2, mitochondrial (AK 2) (EC 2.7.4.3) (ATP-AMP transphosphorylase 2) (ATP:AMP phosphotransferase) (Adenylate monophosphate kinase) [Cleaved into: Adenylate kinase 2, mitochondrial, N-terminally processed]
P00568	Adenylate kinase isoenzyme 1 (AK 1) (EC 2.7.4.3) (EC 2.7.4.6) (ATP-AMP transphosphorylase 1) (ATP:AMP phosphotransferase) (Adenylate monophosphate kinase) (Myokinase)
Q01518	Adenylyl cyclase-associated protein 1 (CAP 1)
Q8IUX7	Adipocyte enhancer-binding protein 1 (AE-binding protein 1) (Aortic carboxypeptidase-like protein)
Q9HDC9	Adipocyte plasma membrane-associated protein (Protein BSCv)
Q15847	Adipogenesis regulatory factor (Adipogenesis factor rich in obesity) (Adipose most abundant gene transcript 2 protein) (Adipose-specific protein 2) (apM-2)
P84077	ADP-ribosylation factor 1
P84085	ADP-ribosylation factor 5
O43488	Aflatoxin B1 aldehyde reductase member 2 (EC 1.1.1.n11) (AFB1 aldehyde reductase 1) (AFB1-AR 1) (Aldoketoreductase 7) (Succinic semialdehyde reductase) (SSA reductase)

P49588	Alanine--tRNA ligase, cytoplasmic (EC 6.1.1.7) (Alanyl-tRNA synthetase) (AlaRS) (Renal carcinoma antigen NY-REN-42)
P14550	Alcohol dehydrogenase [NADP(+)] (EC 1.1.1.2) (Aldehyde reductase) (Aldo-keto reductase family 1 member A1)
P11766	Alcohol dehydrogenase class-3 (EC 1.1.1.1) (Alcohol dehydrogenase 5) (Alcohol dehydrogenase class chi chain) (Alcohol dehydrogenase class-III) (Glutathione-dependent formaldehyde dehydrogenase) (FALDH) (FDH) (GSH-FDH) (EC 1.1.1.-) (S-(hydroxymethyl)glutathione dehydrogenase) (EC 1.1.1.284)
P05091	Aldehyde dehydrogenase, mitochondrial (EC 1.2.1.3) (ALDH class 2) (ALDH-E2) (ALDHI)
Q96C23	Aldose 1-epimerase (EC 5.1.3.3) (Galactose mutarotase)
P02763	Alpha-1-acid glycoprotein 1 (AGP 1) (Orosomucoid-1) (OMD 1)
P01011	Alpha-1-antichymotrypsin (ACT) (Cell growth-inhibiting gene 24/25 protein) (Serpin A3) [Cleaved into: Alpha-1-antichymotrypsin His-Pro-less]
P01009	Alpha-1-antitrypsin (Alpha-1 protease inhibitor) (Alpha-1-antiproteinase) (Serpin A1) [Cleaved into: Short peptide from AAT (SPAAT)]
P04217	Alpha-1B-glycoprotein (Alpha-1-B glycoprotein)
P01023	Alpha-2-macroglobulin (Alpha-2-M) (C3 and PZP-like alpha-2-macroglobulin domain-containing protein 5)
P12814	Alpha-actinin-1 (Alpha-actinin cytoskeletal isoform) (F-actin cross-linking protein) (Non-muscle alpha-actinin-1)
O43707	Alpha-actinin-4 (Non-muscle alpha-actinin 4)
P35611	Alpha-adducin (Erythrocyte adducin subunit alpha)
P02511	Alpha-crystallin B chain (Alpha(B)-crystallin) (Heat shock protein beta-5) (HspB5) (Renal carcinoma antigen NY-REN-27) (Rosenthal fiber component)
P06733	Alpha-enolase (EC 4.2.1.11) (2-phospho-D-glycerate hydro-lyase) (C-myc promoter-binding protein) (Enolase 1) (MBP-1) (MPB-1) (Non-neural enolase) (NNE) (Phosphopyruvate hydratase) (Plasminogen-binding protein)
Q9NVD7	Alpha-parvin (Actopaxin) (CH-ILKBP) (Calponin-like integrin-linked kinase-binding protein) (Matrix-remodeling-associated protein 2)
P21397	Amine oxidase [flavin-containing] A (EC 1.4.3.4) (Monoamine oxidase type A) (MAO-A)
Q03154	Aminoacylase-1 (ACY-1) (EC 3.5.1.14) (N-acyl-L-amino-acid amidohydrolase)
P04083	Annexin A1 (Annexin I) (Annexin-1) (Calpactin II) (Calpactin-2) (Chromobindin-9) (Lipocortin I) (Phospholipase A2 inhibitory protein) (p35)
P50995	Annexin A11 (56 kDa autoantigen) (Annexin XI) (Annexin-11) (Calcyclin-associated annexin 50) (CAP-50)
P07355	Annexin A2 (Annexin II) (Annexin-2) (Calpactin I heavy chain) (Calpactin-1 heavy chain) (Chromobindin-8) (Lipocortin II) (Placental anticoagulant protein IV) (PAP-IV) (Protein I) (p36)

P12429	Annexin A3 (35-alpha calcimedin) (Annexin III) (Annexin-3) (Inositol 1,2-cyclic phosphate 2-phosphohydrolase) (Lipocortin III) (Placental anticoagulant protein III) (PAP-III)
P09525	Annexin A4 (35-beta calcimedin) (Annexin IV) (Annexin-4) (Carbohydrate-binding protein p33/p41) (Chromobindin-4) (Endonexin I) (Lipocortin IV) (P32.5) (PP4-X) (Placental anticoagulant protein II) (PAP-II) (Protein II)
P08758	Annexin A5 (Anchorin CII) (Annexin V) (Annexin-5) (Calphobindin I) (CBP-I) (Endonexin II) (Lipocortin V) (Placental anticoagulant protein 4) (PP4) (Placental anticoagulant protein I) (PAP-I) (Thromboplastin inhibitor) (Vascular anticoagulant-alpha) (VAC-alpha)
P08133	Annexin A6 (67 kDa calelectrin) (Annexin VI) (Annexin-6) (Calphobindin-II) (CPB-II) (Chromobindin-20) (Lipocortin VI) (Protein III) (p68) (p70)
P20073	Annexin A7 (Annexin VII) (Annexin-7) (Synexin)
P01008	Antithrombin-III (ATIII) (Serpin C1)
Q10567	AP-1 complex subunit beta-1 (Adaptor protein complex AP-1 subunit beta-1) (Adaptor-related protein complex 1 subunit beta-1) (Beta-1-adaptin) (Beta-adaptin 1) (Clathrin assembly protein complex 1 beta large chain) (Golgi adaptor HA1/AP1 adaptin beta subunit)
O94973	AP-2 complex subunit alpha-2 (100 kDa coated vesicle protein C) (Adaptor protein complex AP-2 subunit alpha-2) (Adaptor-related protein complex 2 subunit alpha-2) (Alpha-adaptin C) (Alpha2-adaptin) (Clathrin assembly protein complex 2 alpha-C large chain) (Huntingtin yeast partner J) (Huntingtin-interacting protein 9) (HIP-9) (Huntingtin-interacting protein J) (Plasma membrane adaptor HA2/AP2 adaptin alpha C subunit)
P63010	AP-2 complex subunit beta (AP105B) (Adaptor protein complex AP-2 subunit beta) (Adaptor-related protein complex 2 subunit beta) (Beta-2-adaptin) (Beta-adaptin) (Clathrin assembly protein complex 2 beta large chain) (Plasma membrane adaptor HA2/AP2 adaptin beta subunit)
P02647	Apolipoprotein A-I (Apo-AI) (ApoA-I) (Apolipoprotein A1) [Cleaved into: Proapolipoprotein A-I (ProapoA-I); Truncated apolipoprotein A-I (Apolipoprotein A-I(1-242))]
P06727	Apolipoprotein A-IV (Apo-AIV) (ApoA-IV) (Apolipoprotein A4)
P04114	Apolipoprotein B-100 (Apo B-100) [Cleaved into: Apolipoprotein B-48 (Apo B-48)]
P05090	Apolipoprotein D (Apo-D) (ApoD)
P02649	Apolipoprotein E (Apo-E)
O95831	Apoptosis-inducing factor 1, mitochondrial (EC 1.1.1.-) (Programmed cell death protein 8)
Q9UKV3	Apoptotic chromatin condensation inducer in the nucleus (Acinus)
P54136	Arginine--tRNA ligase, cytoplasmic (EC 6.1.1.19) (Arginyl-tRNA synthetase) (ArgRS)
P00966	Argininosuccinate synthase (EC 6.3.4.5) (Citrulline--aspartate ligase)
O43776	Asparagine--tRNA ligase, cytoplasmic (EC 6.1.1.22) (Asparaginyl-tRNA synthetase) (AsnRS)

P17174	Aspartate aminotransferase, cytoplasmic (cAspAT) (EC 2.6.1.1) (EC 2.6.1.3) (Cysteine aminotransferase, cytoplasmic) (Cysteine transaminase, cytoplasmic) (cCAT) (Glutamate oxaloacetate transaminase 1) (Transaminase A)
P00505	Aspartate aminotransferase, mitochondrial (mAspAT) (EC 2.6.1.1) (EC 2.6.1.7) (Fatty acid-binding protein) (FABP-1) (Glutamate oxaloacetate transaminase 2) (Kynurenine aminotransferase 4) (Kynurenine aminotransferase IV) (Kynurenine--oxoglutarate transaminase 4) (Kynurenine--oxoglutarate transaminase IV) (Plasma membrane-associated fatty acid-binding protein) (FABPpm) (Transaminase A)
Q9ULA0	Aspartyl aminopeptidase (EC 3.4.11.21)
Q9BXN1	Asporin (Periodontal ligament-associated protein 1) (PLAP-1)
P25705	ATP synthase subunit alpha, mitochondrial
P06576	ATP synthase subunit beta, mitochondrial (EC 3.6.3.14)
O75947	ATP synthase subunit d, mitochondrial (ATPase subunit d)
P53396	ATP-citrate synthase (EC 2.3.3.8) (ATP-citrate (pro-S-)-lyase) (ACL) (Citrate cleavage enzyme)
P17858	ATP-dependent 6-phosphofructokinase, liver type (ATP-PFK) (PFK-L) (EC 2.7.1.11) (6-phosphofructokinase type B) (Phosphofructo-1-kinase isozyme B) (PFK-B) (Phosphohexokinase)
Q92499	ATP-dependent RNA helicase DDX1 (EC 3.6.4.13) (DEAD box protein 1) (DEAD box protein retinoblastoma) (DBP-RB)
O00571	ATP-dependent RNA helicase DDX3X (EC 3.6.4.13) (DEAD box protein 3, X-chromosomal) (DEAD box, X isoform) (Helicase-like protein 2) (HLP2)
Q9UII2	ATPase inhibitor, mitochondrial (Inhibitor of F(1)F(o)-ATPase) (IF(1)) (IF1)
P51572	B-cell receptor-associated protein 31 (BCR-associated protein 31) (Bap31) (6C6-AG tumor-associated antigen) (Protein CDM) (p28)
P02730	Band 3 anion transport protein (Anion exchange protein 1) (AE 1) (Anion exchanger 1) (Solute carrier family 4 member 1) (CD antigen CD233)
O43491	Band 4.1-like protein 2 (Generally expressed protein 4.1) (4.1G)
P50895	Basal cell adhesion molecule (Auberger B antigen) (B-CAM cell surface glycoprotein) (F8/G253 antigen) (Lutheran antigen) (Lutheran blood group glycoprotein) (CD antigen CD239)
P98160	Basement membrane-specific heparan sulfate proteoglycan core protein (HSPG) (Perlecan) (PLC) [Cleaved into: Endorepellin; LG3 peptide]
P35613	Basigin (5F7) (Collagenase stimulatory factor) (Extracellular matrix metalloproteinase inducer) (EMMPRIN) (Leukocyte activation antigen M6) (OK blood group antigen) (Tumor cell-derived collagenase stimulatory factor) (TCSF) (CD antigen CD147)
P02749	Beta-2-glycoprotein 1 (APC inhibitor) (Activated protein C-binding protein) (Anticardiolipin cofactor) (Apolipoprotein H) (Apo-H) (Beta-2-glycoprotein I) (B2GPI) (Beta(2)GPI)

P61769	Beta-2-microglobulin [Cleaved into: Beta-2-microglobulin form pI 5.3]
P13929	Beta-enolase (EC 4.2.1.11) (2-phospho-D-glycerate hydro-lyase) (Enolase 3) (Muscle-specific enolase) (MSE) (Skeletal muscle enolase)
P08236	Beta-glucuronidase (EC 3.2.1.31) (Beta-G1)
P06865	Beta-hexosaminidase subunit alpha (EC 3.2.1.52) (Beta-N-acetylhexosaminidase subunit alpha) (Hexosaminidase subunit A) (N-acetyl-beta-glucosaminidase subunit alpha)
P07686	Beta-hexosaminidase subunit beta (EC 3.2.1.52) (Beta-N-acetylhexosaminidase subunit beta) (Hexosaminidase subunit B) (Cervical cancer proto-oncogene 7 protein) (HCC-7) (N-acetyl-beta-glucosaminidase subunit beta) [Cleaved into: Beta-hexosaminidase subunit beta chain B; Beta-hexosaminidase subunit beta chain A]
P31939	Bifunctional purine biosynthesis protein PURH [Cleaved into: Bifunctional purine biosynthesis protein PURH, N-terminally processed] [Includes: Phosphoribosylaminoimidazolecarboxamide formyltransferase (EC 2.1.2.3) (5-aminoimidazole-4-carboxamide ribonucleotide formyltransferase) (AICAR transformylase); IMP cyclohydrolase (EC 3.5.4.10) (ATIC) (IMP synthase) (Inosinicase)]
P21810	Biglycan (Bone/cartilage proteoglycan I) (PG-S1)
P53004	Biliverdin reductase A (BVR A) (EC 1.3.1.24) (Biliverdin-IX alpha-reductase)
Q9H3K6	BolA-like protein 2
P80723	Brain acid soluble protein 1 (22 kDa neuronal tissue-enriched acidic protein) (Neuronal axonal membrane protein NAP-22)
Q96CX2	BTB/POZ domain-containing protein KCTD12 (Pfetin) (Predominantly fetal expressed T1 domain)
P11586	C-1-tetrahydrofolate synthase, cytoplasmic (C1-THF synthase) [Cleaved into: C-1-tetrahydrofolate synthase, cytoplasmic, N-terminally processed] [Includes: Methylenetetrahydrofolate dehydrogenase (EC 1.5.1.5); Methenyltetrahydrofolate cyclohydrolase (EC 3.5.4.9); Formyltetrahydrofolate synthetase (EC 6.3.4.3)]
P12830	Cadherin-1 (CAM 120/80) (Epithelial cadherin) (E-cadherin) (Uvomorulin) (CD antigen CD324) [Cleaved into: E-Cad/CTF1; E-Cad/CTF2; E-Cad/CTF3]
P98194	Calcium-transporting ATPase type 2C member 1 (ATPase 2C1) (EC 3.6.3.8) (ATP-dependent Ca(2+) pump PMR1)
Q13557	Calcium/calmodulin-dependent protein kinase type II subunit delta (CaM kinase II subunit delta) (CaMK-II subunit delta) (EC 2.7.11.17)
Q05682	Caldesmon (CDM)
P0DP25	Calmodulin-3
P27824	Calnexin (IP90) (Major histocompatibility complex class I antigen-binding protein p88) (p90)

P04632	Calpain small subunit 1 (CSS1) (Calcium-activated neutral proteinase small subunit) (CANP small subunit) (Calcium-dependent protease small subunit) (CDPS) (Calcium-dependent protease small subunit 1) (Calpain regulatory subunit)
P07384	Calpain-1 catalytic subunit (EC 3.4.22.52) (Calcium-activated neutral proteinase 1) (CANP 1) (Calpain mu-type) (Calpain-1 large subunit) (Cell proliferation-inducing gene 30 protein) (Micromolar-calpain) (muCANP)
P17655	Calpain-2 catalytic subunit (EC 3.4.22.53) (Calcium-activated neutral proteinase 2) (CANP 2) (Calpain M-type) (Calpain large polypeptide L2) (Calpain-2 large subunit) (Millimolar-calpain) (M-calpain)
P20810	Calpastatin (Calpain inhibitor) (Sperm BS-17 component)
P51911	Calponin-1 (Basic calponin) (Calponin H1, smooth muscle)
Q15417	Calponin-3 (Calponin, acidic isoform)
P27797	Calreticulin (CRP55) (Calregulin) (Endoplasmic reticulum resident protein 60) (ERp60) (HACBP) (grp60)
O43852	Calumenin (Crocalbin) (IEF SSP 9302)
P10644	cAMP-dependent protein kinase type I-alpha regulatory subunit (Tissue-specific extinguisher 1) (TSE1) [Cleaved into: cAMP-dependent protein kinase type I-alpha regulatory subunit, N-terminally processed]
P13861	cAMP-dependent protein kinase type II-alpha regulatory subunit
P00915	Carbonic anhydrase 1 (EC 4.2.1.1) (Carbonate dehydratase I) (Carbonic anhydrase B) (CAB) (Carbonic anhydrase I) (CA-I)
P00918	Carbonic anhydrase 2 (EC 4.2.1.1) (Carbonate dehydratase II) (Carbonic anhydrase C) (CAC) (Carbonic anhydrase II) (CA-II)
P16152	Carbonyl reductase [NADPH] 1 (EC 1.1.1.184) (15-hydroxyprostaglandin dehydrogenase [NADP(+)]) (EC 1.1.1.197) (NADPH-dependent carbonyl reductase 1) (Prostaglandin 9-ketoreductase) (Prostaglandin-E(2) 9-reductase) (EC 1.1.1.189) (Short chain dehydrogenase/reductase family 21C member 1)
O75828	Carbonyl reductase [NADPH] 3 (EC 1.1.1.184) (NADPH-dependent carbonyl reductase 3) (Short chain dehydrogenase/reductase family 21C member 2)
Q9Y646	Carboxypeptidase Q (EC 3.4.17.-) (Lysosomal dipeptidase) (Plasma glutamate carboxypeptidase)
P49747	Cartilage oligomeric matrix protein (COMP) (Thrombospondin-5) (TSP5)
P04040	Catalase (EC 1.11.1.6)
P07858	Cathepsin B (EC 3.4.22.1) (APP secretase) (APPS) (Cathepsin B1) [Cleaved into: Cathepsin B light chain; Cathepsin B heavy chain]
P07339	Cathepsin D (EC 3.4.23.5) [Cleaved into: Cathepsin D light chain; Cathepsin D heavy chain]
P08311	Cathepsin G (CG) (EC 3.4.21.20)
Q9UBR2	Cathepsin Z (EC 3.4.18.1) (Cathepsin P) (Cathepsin X)
Q6NZI2	Caveolae-associated protein 1 (Cavin-1) (Polymerase I and transcript release factor)

Q969G5	Caveolae-associated protein 3 (Cavin-3) (Protein kinase C delta-binding protein) (Serum deprivation response factor-related gene product that binds to C-kinase) (hSRBC)
Q03135	Caveolin-1
Q13740	CD166 antigen (Activated leukocyte cell adhesion molecule) (CD antigen CD166)
Q9Y5K6	CD2-associated protein (Adapter protein CMS) (Cas ligand with multiple SH3 domains)
P16070	CD44 antigen (CDw44) (Epican) (Extracellular matrix receptor III) (ECMR-III) (GP90 lymphocyte homing/adhesion receptor) (HUTCH-I) (Heparan sulfate proteoglycan) (Hermes antigen) (Hyaluronate receptor) (Phagocytic glycoprotein 1) (PGP-1) (Phagocytic glycoprotein I) (PGP-I) (CD antigen CD44)
P60953	Cell division control protein 42 homolog (G25K GTP-binding protein)
P43121	Cell surface glycoprotein MUC18 (Cell surface glycoprotein P1H12) (Melanoma cell adhesion molecule) (Melanoma-associated antigen A32) (Melanoma-associated antigen MUC18) (S-endo 1 endothelial-associated antigen) (CD antigen CD146)
P00450	Ceruloplasmin (EC 1.16.3.1) (Ferroxidase)
O00299	Chloride intracellular channel protein 1 (Chloride channel ABP) (Nuclear chloride ion channel 27) (NCC27) (Regulatory nuclear chloride ion channel protein) (hRNCC)
Q13185	Chromobox protein homolog 3 (HECH) (Heterochromatin protein 1 homolog gamma) (HP1 gamma) (Modifier 2 protein)
P23946	Chymase (EC 3.4.21.39) (Alpha-chymase) (Mast cell protease I)
O75390	Citrate synthase, mitochondrial (EC 2.3.3.1) (Citrate (Si)-synthase)
Q00610	Clathrin heavy chain 1 (Clathrin heavy chain on chromosome 17) (CLH-17)
P09496	Clathrin light chain A (Lca)
P09497	Clathrin light chain B (Lcb)
P10909	Clusterin (Aging-associated gene 4 protein) (Apolipoprotein J) (Apo-J) (Complement cytolysis inhibitor) (CLI) (Complement-associated protein SP-40,40) (Ku70-binding protein 1) (NA1/NA2) (Testosterone-repressed prostate message 2) (TRPM-2) [Cleaved into: Clusterin beta chain (ApoJalpha) (Complement cytolysis inhibitor a chain); Clusterin alpha chain (ApoJbeta) (Complement cytolysis inhibitor b chain)]
Q14019	Coactosin-like protein
P00488	Coagulation factor XIII A chain (Coagulation factor XIIIa) (EC 2.3.2.13) (Protein-glutamine gamma-glutamyltransferase A chain) (Transglutaminase A chain)
P53621	Coatomer subunit alpha (Alpha-coat protein) (Alpha-COP) (HEP-COP) (HEPCOP) [Cleaved into: Xenin (Xenopsin-related peptide); Proxenin]
P35606	Coatomer subunit beta' (Beta'-coat protein) (Beta'-COP) (p102)
P48444	Coatomer subunit delta (Archain) (Delta-coat protein) (Delta-COP)

O14579	Coatomer subunit epsilon (Epsilon-coat protein) (Epsilon-COP)
Q9Y678	Coatomer subunit gamma-1 (Gamma-1-coat protein) (Gamma-1-COP)
P23528	Cofilin-1 (18 kDa phosphoprotein) (p18) (Cofilin, non-muscle isoform)
Q9Y281	Cofilin-2 (Cofilin, muscle isoform)
Q9Y6H1	Coiled-coil-helix-coiled-coil-helix domain-containing protein 2 (Aging-associated gene 10 protein) (HCV NS2 trans-regulated protein) (NS2TP)
Q14011	Cold-inducible RNA-binding protein (A18 hnRNP) (Glycine-rich RNA-binding protein CIRP)
P02452	Collagen alpha-1(I) chain (Alpha-1 type I collagen)
P12109	Collagen alpha-1(VI) chain
Q99715	Collagen alpha-1(XII) chain
Q05707	Collagen alpha-1(XIV) chain (Undulin)
P39059	Collagen alpha-1(XV) chain [Cleaved into: Restin (Endostatin-XV) (Related to endostatin) (Restin-I); Restin-2 (Restin-II); Restin-3 (Restin-III); Restin-4 (Restin-IV)]
P39060	Collagen alpha-1(XVIII) chain [Cleaved into: Endostatin]
P08123	Collagen alpha-2(I) chain (Alpha-2 type I collagen)
P08572	Collagen alpha-2(IV) chain [Cleaved into: Canstatin]
P12110	Collagen alpha-2(VI) chain
P12111	Collagen alpha-3(VI) chain
P02746	Complement C1q subcomponent subunit B
P01024	Complement C3 (C3 and PZP-like alpha-2-macroglobulin domain-containing protein 1) [Cleaved into: Complement C3 beta chain; C3-beta-c (C3bc); Complement C3 alpha chain; C3a anaphylatoxin; Acylation stimulating protein (ASP) (C3adesArg); Complement C3b alpha' chain; Complement C3c alpha' chain fragment 1; Complement C3dg fragment; Complement C3g fragment; Complement C3d fragment; Complement C3f fragment; Complement C3c alpha' chain fragment 2]
P0C0L4	Complement C4-A (Acidic complement C4) (C3 and PZP-like alpha-2-macroglobulin domain-containing protein 2) [Cleaved into: Complement C4 beta chain; Complement C4-A alpha chain; C4a anaphylatoxin; C4b-A; C4d-A; Complement C4 gamma chain]
Q07021	Complement component 1 Q subcomponent-binding protein, mitochondrial (ASF/SF2-associated protein p32) (Glycoprotein gC1qBP) (C1qBP) (Hyaluronan-binding protein 1) (Mitochondrial matrix protein p32) (gC1q-R protein) (p33)
P02748	Complement component C9 [Cleaved into: Complement component C9a; Complement component C9b]
P00751	Complement factor B (EC 3.4.21.47) (C3/C5 convertase) (Glycine-rich beta glycoprotein) (GBG) (PBF2) (Properdin factor B) [Cleaved into: Complement factor B Ba fragment; Complement factor B Bb fragment]

P08603	Complement factor H (H factor 1)
Q13098	COP9 signalosome complex subunit 1 (SGN1) (Signalosome subunit 1) (G protein pathway suppressor 1) (GPS-1) (JAB1-containing signalosome subunit 1) (Protein MFH)
Q9BT78	COP9 signalosome complex subunit 4 (SGN4) (Signalosome subunit 4) (JAB1-containing signalosome subunit 4)
Q92905	COP9 signalosome complex subunit 5 (SGN5) (Signalosome subunit 5) (EC 3.4.-.-) (Jun activation domain-binding protein 1)
O14618	Copper chaperone for superoxide dismutase (Superoxide dismutase copper chaperone)
O75367	Core histone macro-H2A.1 (Histone macroH2A1) (mH2A1) (Histone H2A.y) (H2A/y) (Medulloblastoma antigen MU-MB-50.205)
P31146	Coronin-1A (Coronin-like protein A) (Clipin-A) (Coronin-like protein p57) (Tryptophan aspartate-containing coat protein) (TACO)
Q9ULV4	Coronin-1C (Coronin-3) (hCRNN4)
P12277	Creatine kinase B-type (EC 2.7.3.2) (B-CK) (Creatine kinase B chain)
Q86VP6	Cullin-associated NEDD8-dissociated protein 1 (Cullin-associated and neddylation-dissociated protein 1) (TBP-interacting protein of 120 kDa A) (TBP-interacting protein 120A) (p120 CAND1)
P04080	Cystatin-B (CPI-B) (Liver thiol proteinase inhibitor) (Stefin-B)
P01034	Cystatin-C (Cystatin-3) (Gamma-trace) (Neuroendocrine basic polypeptide) (Post-gamma-globulin)
P21291	Cysteine and glycine-rich protein 1 (Cysteine-rich protein 1) (CRP) (CRP1) (Epididymis luminal protein 141) (HEL-141)
P52943	Cysteine-rich protein 2 (CRP-2) (Protein ESP1)
P00167	Cytochrome b5 (Microsomal cytochrome b5 type A) (MCB5)
P99999	Cytochrome c
P14854	Cytochrome c oxidase subunit 6B1 (Cytochrome c oxidase subunit VIb isoform 1) (COX VIb-1)
P21399	Cytoplasmic aconitate hydratase (Aconitase) (EC 4.2.1.3) (Citrate hydro-lyase) (Ferritin repressor protein) (Iron regulatory protein 1) (IRP1) (Iron-responsive element-binding protein 1) (IRE-BP 1)
Q14204	Cytoplasmic dynein 1 heavy chain 1 (Cytoplasmic dynein heavy chain 1) (Dynein heavy chain, cytosolic)
Q13409	Cytoplasmic dynein 1 intermediate chain 2 (Cytoplasmic dynein intermediate chain 2) (Dynein intermediate chain 2, cytosolic) (DH IC-2)
Q07065	Cytoskeleton-associated protein 4 (63-kDa cytoskeleton-linking membrane protein) (Climp-63) (p63)
P28838	Cytosol aminopeptidase (EC 3.4.11.1) (Leucine aminopeptidase 3) (LAP-3) (Leucyl aminopeptidase) (Peptidase S) (Proline aminopeptidase) (EC 3.4.11.5) (Prolyl aminopeptidase)

Q96KP4	Cytosolic non-specific dipeptidase (EC 3.4.13.18) (CNDP dipeptidase 2) (Carnosine dipeptidase II) (Epididymis secretory protein Li 13) (Glutamate carboxypeptidase-like protein 1) (Peptidase A)
O43175	D-3-phosphoglycerate dehydrogenase (3-PGDH) (EC 1.1.1.95) (2-oxoglutarate reductase) (EC 1.1.1.399) (Malate dehydrogenase) (EC 1.1.1.37)
Q96HY6	DDRGK domain-containing protein 1 (Dashurin) (UFM1-binding and PCI domain-containing protein 1)
P07585	Decorin (Bone proteoglycan II) (PG-S2) (PG40)
P30038	Delta-1-pyrroline-5-carboxylate dehydrogenase, mitochondrial (P5C dehydrogenase) (EC 1.2.1.88) (Aldehyde dehydrogenase family 4 member A1) (L-glutamate gamma-semialdehyde dehydrogenase)
Q13011	Delta(3,5)-Delta(2,4)-dienoyl-CoA isomerase, mitochondrial (EC 5.3.3.-)
O43583	Density-regulated protein (DRP) (Protein DRP1) (Smooth muscle cell-associated protein 3) (SMAP-3)
Q9Y3Z3	Deoxynucleoside triphosphate triphosphohydrolase SAMHD1 (dNTPase) (EC 3.1.5.-) (Dendritic cell-derived IFNG-induced protein) (DCIP) (Monocyte protein 5) (MOP-5) (SAM domain and HD domain-containing protein 1)
O00115	Deoxyribonuclease-2-alpha (EC 3.1.22.1) (Acid DNase) (Deoxyribonuclease II alpha) (DNase II alpha) (Lysosomal DNase II) (R31240_2)
Q07507	Dermatopontin (Tyrosine-rich acidic matrix protein) (TRAMP)
P17661	Desmin
Q14126	Desmoglein-2 (Cadherin family member 5) (HDGC)
P15924	Desmoplakin (DP) (250/210 kDa paraneoplastic pemphigus antigen)
P60981	Destrin (Actin-depolymerizing factor) (ADF)
P09622	Dihydrolipoyl dehydrogenase, mitochondrial (EC 1.8.1.4) (Dihydrolipoamide dehydrogenase) (Glycine cleavage system L protein)
P36957	Dihydrolipoyllysine-residue succinyltransferase component of 2-oxoglutarate dehydrogenase complex, mitochondrial (EC 2.3.1.61) (2-oxoglutarate dehydrogenase complex component E2) (OGDC-E2) (Dihydrolipoamide succinyltransferase component of 2-oxoglutarate dehydrogenase complex) (E2K)
P09417	Dihydropteridine reductase (EC 1.5.1.34) (HDHPR) (Quinoid dihydropteridine reductase) (Short chain dehydrogenase/reductase family 33C member 1)
Q16555	Dihydropyrimidinase-related protein 2 (DRP-2) (Collapsin response mediator protein 2) (CRMP-2) (N2A3) (Unc-33-like phosphoprotein 2) (ULIP-2)
Q14195	Dihydropyrimidinase-related protein 3 (DRP-3) (Collapsin response mediator protein 4) (CRMP-4) (Unc-33-like phosphoprotein 1) (ULIP-1)
Q9UHL4	Dipeptidyl peptidase 2 (EC 3.4.14.2) (Dipeptidyl aminopeptidase II) (Dipeptidyl peptidase 7) (Dipeptidyl peptidase II) (DPP II) (Quiescent cell proline dipeptidase)

Q9NY33	Dipeptidyl peptidase 3 (EC 3.4.14.4) (Dipeptidyl aminopeptidase III) (Dipeptidyl arylamidase III) (Dipeptidyl peptidase III) (DPP III) (Enkephalinase B)
P27487	Dipeptidyl peptidase 4 (EC 3.4.14.5) (ADABP) (Adenosine deaminase complexing protein 2) (ADCP-2) (Dipeptidyl peptidase IV) (DPP IV) (T-cell activation antigen CD26) (TP103) (CD antigen CD26) [Cleaved into: Dipeptidyl peptidase 4 membrane form (Dipeptidyl peptidase IV membrane form); Dipeptidyl peptidase 4 soluble form (Dipeptidyl peptidase IV soluble form)]
Q16531	DNA damage-binding protein 1 (DDB p127 subunit) (DNA damage-binding protein a) (DDBa) (Damage-specific DNA-binding protein 1) (HBV X-associated protein 1) (XAP-1) (UV-damaged DNA-binding factor) (UV-damaged DNA-binding protein 1) (UV-DDB 1) (XPE-binding factor) (XPE-BF) (Xeroderma pigmentosum group E-complementing protein) (XPCe)
P27695	DNA-(apurinic or apyrimidinic site) lyase (EC 3.1.-.-) (EC 4.2.99.18) (APEX nuclease) (APEN) (Apurinic-apyrimidinic endonuclease 1) (AP endonuclease 1) (APE-1) (REF-1) (Redox factor-1) [Cleaved into: DNA-(apurinic or apyrimidinic site) lyase, mitochondrial]
O60884	DnaJ homolog subfamily A member 2 (Cell cycle progression restoration gene 3 protein) (Dnj3) (Dj3) (HIRA-interacting protein 4) (Renal carcinoma antigen NY-REN-14)
P25685	DnaJ homolog subfamily B member 1 (DnaJ protein homolog 1) (Heat shock 40 kDa protein 1) (HSP40) (Heat shock protein 40) (Human DnaJ protein 1) (hDj-1)
O75937	DnaJ homolog subfamily C member 8 (Splicing protein spf31)
P04843	Dolichyl-diphosphooligosaccharide--protein glycosyltransferase subunit 1 (Dolichyl-diphosphooligosaccharide--protein glycosyltransferase 67 kDa subunit) (Ribophorin I) (RPN-I) (Ribophorin-1)
Q16643	Drebrin (Developmentally-regulated brain protein)
Q9UJU6	Drebrin-like protein (Cervical SH3P7) (Cervical mucin-associated protein) (Drebrin-F) (HPK1-interacting protein of 55 kDa) (HIP-55) (SH3 domain-containing protein 7)
Q02750	Dual specificity mitogen-activated protein kinase kinase 1 (MAP kinase kinase 1) (MAPKK 1) (MKK1) (EC 2.7.12.2) (ERK activator kinase 1) (MAPK/ERK kinase 1) (MEK 1)
Q14203	Dynactin subunit 1 (150 kDa dynein-associated polypeptide) (DAP-150) (DP-150) (p135) (p150-glued)
Q13561	Dynactin subunit 2 (50 kDa dynein-associated polypeptide) (Dynactin complex 50 kDa subunit) (DCTN-50) (p50 dynamitin)
O00429	Dynamin-1-like protein (EC 3.6.5.5) (Dnm1p/Vps1p-like protein) (DVLP) (Dynamin family member proline-rich carboxyl-terminal domain less) (Dymple) (Dynamin-like protein) (Dynamin-like protein 4) (Dynamin-like protein IV) (HdynIV) (Dynamin-related protein 1)
P50570	Dynamin-2 (EC 3.6.5.5)
O60313	Dynamin-like 120 kDa protein, mitochondrial (EC 3.6.5.5) (Optic atrophy protein 1) [Cleaved into: Dynamin-like 120 kDa protein, form S1]

Q96FJ2	Dynein light chain 2, cytoplasmic (8 kDa dynein light chain b) (DLC8b) (Dynein light chain LC8-type 2)
Q7Z6Z7	E3 ubiquitin-protein ligase HUWE1 (EC 2.3.2.26) (ARF-binding protein 1) (ARF-BP1) (HECT, UBA and WWE domain-containing protein 1) (HECT-type E3 ubiquitin transferase HUWE1) (Homologous to E6AP carboxyl terminus homologous protein 9) (HectH9) (Large structure of UREB1) (LASU1) (Mcl-1 ubiquitin ligase E3) (Mule) (Upstream regulatory element-binding protein 1) (URE-B1) (URE-binding protein 1)
Q15075	Early endosome antigen 1 (Endosome-associated protein p162) (Zinc finger FYVE domain-containing protein 2)
O95834	Echinoderm microtubule-associated protein-like 2 (EMAP-2) (HuEMAP-2)
Q96C19	EF-hand domain-containing protein D2 (Swiprosin-1)
Q12805	EGF-containing fibulin-like extracellular matrix protein 1 (Extracellular protein S1-5) (Fibrillin-like protein) (Fibulin-3) (FIBL-3)
Q9NZN4	EH domain-containing protein 2 (PAST homolog 2)
P13804	Electron transfer flavoprotein subunit alpha, mitochondrial (Alpha-ETF)
P38117	Electron transfer flavoprotein subunit beta (Beta-ETF)
P29692	Elongation factor 1-delta (EF-1-delta) (Antigen NY-CO-4)
P26641	Elongation factor 1-gamma (EF-1-gamma) (eEF-1B gamma)
P13639	Elongation factor 2 (EF-2)
P49411	Elongation factor Tu, mitochondrial (EF-Tu) (P43)
Q15370	Elongin-B (EloB) (Elongin 18 kDa subunit) (RNA polymerase II transcription factor SIII subunit B) (SIII p18) (Transcription elongation factor B polypeptide 2)
Q15369	Elongin-C (EloC) (Elongin 15 kDa subunit) (RNA polymerase II transcription factor SIII subunit C) (SIII p15) (Transcription elongation factor B polypeptide 1)
Q9Y6C2	EMILIN-1 (Elastin microfibril interface-located protein 1) (Elastin microfibril interfacer 1)
O94919	Endonuclease domain-containing 1 protein (EC 3.1.30.-)
P30040	Endoplasmic reticulum resident protein 29 (ERp29) (Endoplasmic reticulum resident protein 28) (ERp28) (Endoplasmic reticulum resident protein 31) (ERp31)
Q9BS26	Endoplasmic reticulum resident protein 44 (ER protein 44) (ERp44) (Thioredoxin domain-containing protein 4)
P14625	Endoplasmin (94 kDa glucose-regulated protein) (GRP-94) (Heat shock protein 90 kDa beta member 1) (Tumor rejection antigen 1) (gp96 homolog)
O60869	Endothelial differentiation-related factor 1 (EDF-1) (Multiprotein-bridging factor 1) (MBF1)
P30084	Enoyl-CoA hydratase, mitochondrial (EC 4.2.1.17) (Enoyl-CoA hydratase 1) (Short-chain enoyl-CoA hydratase) (SCEH)

P61916	Epididymal secretory protein E1 (Human epididymis-specific protein 1) (He1) (Niemann-Pick disease type C2 protein)
P07099	Epoxide hydrolase 1 (EC 3.3.2.9) (Epoxide hydratase) (Microsomal epoxide hydrolase)
P27105	Erythrocyte band 7 integral membrane protein (Protein 7.2b) (Stomatin)
Q9H0W9	Ester hydrolase C11orf54 (EC 3.1.-.-)
P60842	Eukaryotic initiation factor 4A-I (eIF-4A-I) (eIF4A-I) (EC 3.6.4.13) (ATP-dependent RNA helicase eIF4A-1)
Q14240	Eukaryotic initiation factor 4A-II (eIF-4A-II) (eIF4A-II) (EC 3.6.4.13) (ATP-dependent RNA helicase eIF4A-2)
P15170	Eukaryotic peptide chain release factor GTP-binding subunit ERF3A (Eukaryotic peptide chain release factor subunit 3a) (eRF3a) (G1 to S phase transition protein 1 homolog)
P41091	Eukaryotic translation initiation factor 2 subunit 3 (Eukaryotic translation initiation factor 2 subunit gamma X) (eIF-2-gamma X) (eIF-2gX)
Q14152	Eukaryotic translation initiation factor 3 subunit A (eIF3a) (Eukaryotic translation initiation factor 3 subunit 10) (eIF-3-theta) (eIF3 p167) (eIF3 p180) (eIF3 p185)
P55884	Eukaryotic translation initiation factor 3 subunit B (eIF3b) (Eukaryotic translation initiation factor 3 subunit 9) (Prt1 homolog) (hPrt1) (eIF-3-eta) (eIF3 p110) (eIF3 p116)
Q04637	Eukaryotic translation initiation factor 4 gamma 1 (eIF-4-gamma 1) (eIF-4G 1) (eIF-4G1) (p220)
P23588	Eukaryotic translation initiation factor 4B (eIF-4B)
Q15056	Eukaryotic translation initiation factor 4H (eIF-4H) (Williams-Beuren syndrome chromosomal region 1 protein)
P63241	Eukaryotic translation initiation factor 5A-1 (eIF-5A-1) (eIF-5A1) (Eukaryotic initiation factor 5A isoform 1) (eIF-5A) (Rev-binding factor) (eIF-4D)
P56537	Eukaryotic translation initiation factor 6 (eIF-6) (B(2)GCN homolog) (B4 integrin interactor) (CAB) (p27(BBP))
O14980	Exportin-1 (Exp1) (Chromosome region maintenance 1 protein homolog)
Q9BSJ8	Extended synaptotagmin-1 (E-Syt1) (Membrane-bound C2 domain-containing protein)
P15311	Ezrin (Cytovillin) (Villin-2) (p81)
P52907	F-actin-capping protein subunit alpha-1 (CapZ alpha-1)
P47755	F-actin-capping protein subunit alpha-2 (CapZ alpha-2)
P47756	F-actin-capping protein subunit beta (CapZ beta)
Q08945	FACT complex subunit SSRP1 (Chromatin-specific transcription elongation factor 80 kDa subunit) (Facilitates chromatin transcription complex 80 kDa subunit) (FACT 80 kDa subunit) (FACTp80) (Facilitates chromatin transcription complex subunit SSRP1) (Recombination signal sequence recognition protein 1) (Structure-specific recognition protein 1) (hSSRP1) (T160)
Q92945	Far upstream element-binding protein 2 (FUSE-binding protein 2) (KH type-splicing regulatory protein) (KSRP) (p75)

Q16658	Fascin (55 kDa actin-bundling protein) (Singed-like protein) (p55)
Q01469	Fatty acid-binding protein, epidermal (Epidermal-type fatty acid-binding protein) (E-FABP) (Fatty acid-binding protein 5) (Psoriasis-associated fatty acid-binding protein homolog) (PA-FABP)
P05413	Fatty acid-binding protein, heart (Fatty acid-binding protein 3) (Heart-type fatty acid-binding protein) (H-FABP) (Mammary-derived growth inhibitor) (MDGI) (Muscle fatty acid-binding protein) (M-FABP)
Q96AC1	Fermitin family homolog 2 (Kindlin-2) (Mitogen-inducible gene 2 protein) (MIG-2) (Pleckstrin homology domain-containing family C member 1) (PH domain-containing family C member 1)
P02794	Ferritin heavy chain (Ferritin H subunit) (EC 1.16.3.1) (Cell proliferation-inducing gene 15 protein) [Cleaved into: Ferritin heavy chain, N-terminally processed]
P02792	Ferritin light chain (Ferritin L subunit)
P35555	Fibrillin-1 [Cleaved into: Asprosin]
P02671	Fibrinogen alpha chain [Cleaved into: Fibrinopeptide A; Fibrinogen alpha chain]
P02675	Fibrinogen beta chain [Cleaved into: Fibrinopeptide B; Fibrinogen beta chain]
P02679	Fibrinogen gamma chain
P02751	Fibronectin (FN) (Cold-insoluble globulin) (CIG) [Cleaved into: Anastellin; Ugl-Y1; Ugl-Y2; Ugl-Y3]
P23142	Fibulin-1 (FIBL-1)
P98095	Fibulin-2 (FIBL-2)
Q9UBX5	Fibulin-5 (FIBL-5) (Developmental arteries and neural crest EGF-like protein) (Dance) (Urine p50 protein) (UP50)
P21333	Filamin-A (FLN-A) (Actin-binding protein 280) (ABP-280) (Alpha-filamin) (Endothelial actin-binding protein) (Filamin-1) (Non-muscle filamin)
O75369	Filamin-B (FLN-B) (ABP-278) (ABP-280 homolog) (Actin-binding-like protein) (Beta-filamin) (Filamin homolog 1) (Fh1) (Filamin-3) (Thyroid autoantigen) (Truncated actin-binding protein) (Truncated ABP)
Q14315	Filamin-C (FLN-C) (FLNc) (ABP-280-like protein) (ABP-L) (Actin-binding-like protein) (Filamin-2) (Gamma-filamin)
P30043	Flavin reductase (NADPH) (FR) (EC 1.5.1.30) (Biliverdin reductase B) (BVR-B) (EC 1.3.1.24) (Biliverdin-IX beta-reductase) (Green heme-binding protein) (GHBP) (NADPH-dependent diaphorase) (NADPH-flavin reductase) (FLR)
O75955	Flotillin-1
Q14254	Flotillin-2 (Epidermal surface antigen) (ESA) (Membrane component chromosome 17 surface marker 1)
Q12841	Follistatin-related protein 1 (Follistatin-like protein 1)
Q13642	Four and a half LIM domains protein 1 (FHL-1) (Skeletal muscle LIM-protein 1) (SLIM) (SLIM-1)

P09467	Fructose-1,6-bisphosphatase 1 (FBPase 1) (EC 3.1.3.11) (D-fructose-1,6-bisphosphate 1-phosphohydrolase 1) (Liver FBPase)
P04075	Fructose-bisphosphate aldolase A (EC 4.1.2.13) (Lung cancer antigen NY-LU-1) (Muscle-type aldolase)
P09972	Fructose-bisphosphate aldolase C (EC 4.1.2.13) (Brain-type aldolase)
P07954	Fumarate hydratase, mitochondrial (Fumarase) (EC 4.2.1.2)
P09382	Galectin-1 (Gal-1) (14 kDa laminin-binding protein) (HLBP14) (14 kDa lectin) (Beta-galactoside-binding lectin L-14-I) (Galaptin) (HBL) (HPL) (Lactose-binding lectin 1) (Lectin galactoside-binding soluble 1) (Putative MAPK-activating protein PM12) (S-Lac lectin 1)
P17931	Galectin-3 (Gal-3) (35 kDa lectin) (Carbohydrate-binding protein 35) (CBP 35) (Galactose-specific lectin 3) (Galactoside-binding protein) (GALBP) (IgE-binding protein) (L-31) (Laminin-binding protein) (Lectin L-29) (Mac-2 antigen)
Q08380	Galectin-3-binding protein (Basement membrane autoantigen p105) (Lectin galactoside-binding soluble 3-binding protein) (Mac-2-binding protein) (MAC2BP) (Mac-2 BP) (Tumor-associated antigen 90K)
P09104	Gamma-enolase (EC 4.2.1.11) (2-phospho-D-glycerate hydro-lyase) (Enolase 2) (Neural enolase) (Neuron-specific enolase) (NSE)
Q13630	GDP-L-fucose synthase (EC 1.1.1.271) (GDP-4-keto-6-deoxy-D-mannose-3,5-epimerase-4-reductase) (Protein FX) (Red cell NADP(H)-binding protein) (Short-chain dehydrogenase/reductase family 4E member 1)
P06396	Gelsolin (AGEL) (Actin-depolymerizing factor) (ADF) (Brevin)
O60763	General vesicular transport factor p115 (Protein USO1 homolog) (Transcytosis-associated protein) (TAP) (Vesicle-docking protein)
P60983	Glia maturation factor beta (GMF-beta)
P11413	Glucose-6-phosphate 1-dehydrogenase (G6PD) (EC 1.1.1.49)
P06744	Glucose-6-phosphate isomerase (GPI) (EC 5.3.1.9) (Autocrine motility factor) (AMF) (Neuroleukin) (NLK) (Phosphoglucose isomerase) (PGI) (Phosphohexose isomerase) (PHI) (Sperm antigen 36) (SA-36)
P14314	Glucosidase 2 subunit beta (80K-H protein) (Glucosidase II subunit beta) (Protein kinase C substrate 60.1 kDa protein heavy chain) (PKCSH)
P00367	Glutamate dehydrogenase 1, mitochondrial (GDH 1) (EC 1.4.1.3)
P07203	Glutathione peroxidase 1 (GPx-1) (GSHPx-1) (EC 1.11.1.9) (Cellular glutathione peroxidase)
P00390	Glutathione reductase, mitochondrial (GR) (GRase) (EC 1.8.1.7)
P78417	Glutathione S-transferase omega-1 (GSTO-1) (EC 2.5.1.18) (Glutathione S-transferase omega 1-1) (GSTO 1-1) (Glutathione-dependent dehydroascorbate reductase) (EC 1.8.5.1) (Monomethylarsonic acid reductase) (MMA(V) reductase) (EC 1.20.4.2) (S-(Phenacyl)glutathione reductase) (SPG-R)
P09211	Glutathione S-transferase P (EC 2.5.1.18) (GST class-pi) (GSTP1-1)

P48637	Glutathione synthetase (GSH synthetase) (GSH-S) (EC 6.3.2.3) (Glutathione synthase)
P04406	Glyceraldehyde-3-phosphate dehydrogenase (GAPDH) (EC 1.2.1.12) (Peptidyl-cysteine S-nitrosylase GAPDH) (EC 2.6.99.-)
P11216	Glycogen phosphorylase, brain form (EC 2.4.1.1)
Q9HC38	Glyoxalase domain-containing protein 4
Q9UBQ7	Glyoxylate reductase/hydroxypyruvate reductase (EC 1.1.1.79) (EC 1.1.1.81)
Q9H4G4	Golgi-associated plant pathogenesis-related protein 1 (GAPR-1) (Golgi-associated PR-1 protein) (Glioma pathogenesis-related protein 2) (GliPR 2)
Q14789	Golgin subfamily B member 1 (372 kDa Golgi complex-associated protein) (GCP372) (Giantin) (Macrogolgin)
P62993	Growth factor receptor-bound protein 2 (Adapter protein GRB2) (Protein Ash) (SH2/SH3 adapter GRB2)
P62826	GTP-binding nuclear protein Ran (Androgen receptor-associated protein 24) (GTPase Ran) (Ras-like protein TC4) (Ras-related nuclear protein)
P04899	Guanine nucleotide-binding protein G(i) subunit alpha-2 (Adenylate cyclase-inhibiting G alpha protein)
P62873	Guanine nucleotide-binding protein G(I)/G(S)/G(T) subunit beta-1 (Transducin beta chain 1)
P62879	Guanine nucleotide-binding protein G(I)/G(S)/G(T) subunit beta-2 (G protein subunit beta-2) (Transducin beta chain 2)
P08754	Guanine nucleotide-binding protein G(k) subunit alpha (G(i) alpha-3)
P00738	Haptoglobin (Zonulin) [Cleaved into: Haptoglobin alpha chain; Haptoglobin beta chain]
Q53T59	HCLS1-binding protein 3 (HS1-binding protein 3) (HSP1BP-3)
P0DMV9	Heat shock 70 kDa protein 1B (Heat shock 70 kDa protein 2) (HSP70-2) (HSP70.2)
P34932	Heat shock 70 kDa protein 4 (HSP70RY) (Heat shock 70-related protein APG-2)
P11142	Heat shock cognate 71 kDa protein (Heat shock 70 kDa protein 8) (Lipopolysaccharide-associated protein 1) (LAP-1) (LPS-associated protein 1)
P04792	Heat shock protein beta-1 (HspB1) (28 kDa heat shock protein) (Estrogen-regulated 24 kDa protein) (Heat shock 27 kDa protein) (HSP 27) (Stress-responsive protein 27) (SRP27)
O14558	Heat shock protein beta-6 (HspB6) (Heat shock 20 kDa-like protein p20)
P07900	Heat shock protein HSP 90-alpha (Heat shock 86 kDa) (HSP 86) (HSP86) (Lipopolysaccharide-associated protein 2) (LAP-2) (LPS-associated protein 2) (Renal carcinoma antigen NY-REN-38)
P08238	Heat shock protein HSP 90-beta (HSP 90) (Heat shock 84 kDa) (HSP 84) (HSP84)
Q9Y5Z4	Heme-binding protein 2 (Placental protein 23) (PP23) (Protein SOUL)
P69905	Hemoglobin subunit alpha (Alpha-globin) (Hemoglobin alpha chain)
P02042	Hemoglobin subunit delta (Delta-globin) (Hemoglobin delta chain)
P02790	Hemopexin (Beta-1B-glycoprotein)

P51858	Hepatoma-derived growth factor (HDGF) (High mobility group protein 1-like 2) (HMG-1L2)
Q5SSJ5	Heterochromatin protein 1-binding protein 3 (Protein HP1-BP74)
Q99729	Heterogeneous nuclear ribonucleoprotein A/B (hnRNP A/B) (APOBEC1-binding protein 1) (ABBP-1)
P09651	Heterogeneous nuclear ribonucleoprotein A1 (hnRNP A1) (Helix-destabilizing protein) (Single-strand RNA-binding protein) (hnRNP core protein A1) [Cleaved into: Heterogeneous nuclear ribonucleoprotein A1, N-terminally processed]
P51991	Heterogeneous nuclear ribonucleoprotein A3 (hnRNP A3)
O14979	Heterogeneous nuclear ribonucleoprotein D-like (hnRNP D-like) (hnRNP DL) (AU-rich element RNA-binding factor) (JKT41-binding protein) (Protein laAUF1)
Q14103	Heterogeneous nuclear ribonucleoprotein D0 (hnRNP D0) (AU-rich element RNA-binding protein 1)
P52597	Heterogeneous nuclear ribonucleoprotein F (hnRNP F) (Nucleolin-like protein mcs94-1) [Cleaved into: Heterogeneous nuclear ribonucleoprotein F, N-terminally processed]
P31943	Heterogeneous nuclear ribonucleoprotein H (hnRNP H) [Cleaved into: Heterogeneous nuclear ribonucleoprotein H, N-terminally processed]
P31942	Heterogeneous nuclear ribonucleoprotein H3 (hnRNP H3) (Heterogeneous nuclear ribonucleoprotein 2H9) (hnRNP 2H9)
P61978	Heterogeneous nuclear ribonucleoprotein K (hnRNP K) (Transformation up-regulated nuclear protein) (TUNP)
P14866	Heterogeneous nuclear ribonucleoprotein L (hnRNP L)
P52272	Heterogeneous nuclear ribonucleoprotein M (hnRNP M)
O60506	Heterogeneous nuclear ribonucleoprotein Q (hnRNP Q) (Glycine- and tyrosine-rich RNA-binding protein) (GRY-RBP) (NS1-associated protein 1) (Synaptotagmin-binding, cytoplasmic RNA-interacting protein)
O43390	Heterogeneous nuclear ribonucleoprotein R (hnRNP R)
Q00839	Heterogeneous nuclear ribonucleoprotein U (hnRNP U) (Scaffold attachment factor A) (SAF-A) (p120) (pp120)
Q1KMD3	Heterogeneous nuclear ribonucleoprotein U-like protein 2 (Scaffold-attachment factor A2) (SAF-A2)
P22626	Heterogeneous nuclear ribonucleoproteins A2/B1 (hnRNP A2/B1)
P07910	Heterogeneous nuclear ribonucleoproteins C1/C2 (hnRNP C1/C2)
P19367	Hexokinase-1 (EC 2.7.1.1) (Brain form hexokinase) (Hexokinase type I) (HK I)
P09429	High mobility group protein B1 (High mobility group protein 1) (HMG-1)
P49773	Histidine triad nucleotide-binding protein 1 (EC 3.-.-.-) (Adenosine 5'-monophosphoramidase) (Protein kinase C inhibitor 1) (Protein kinase C-interacting protein 1) (PKCI-1)
P04196	Histidine-rich glycoprotein (Histidine-proline-rich glycoprotein) (HPRG)
P16401	Histone H1.5 (Histone H1a) (Histone H1b) (Histone H1s-3)
P84243	Histone H3.3

P62805	Histone H4
P01903	HLA class II histocompatibility antigen, DR alpha chain (MHC class II antigen DRA)
P50502	Hsc70-interacting protein (Hip) (Aging-associated protein 2) (Progesterone receptor-associated p48 protein) (Protein FAM10A1) (Putative tumor suppressor ST13) (Renal carcinoma antigen NY-REN-33) (Suppression of tumorigenicity 13 protein)
Q16543	Hsp90 co-chaperone Cdc37 (Hsp90 chaperone protein kinase-targeting subunit) (p50Cdc37) [Cleaved into: Hsp90 co-chaperone Cdc37, N-terminally processed]
Q16836	Hydroxyacyl-coenzyme A dehydrogenase, mitochondrial (HCDH) (EC 1.1.1.35) (Medium and short-chain L-3-hydroxyacyl-coenzyme A dehydrogenase) (Short-chain 3-hydroxyacyl-CoA dehydrogenase)
Q16775	Hydroxyacylglutathione hydrolase, mitochondrial (EC 3.1.2.6) (Glyoxalase II) (Glx II)
P00492	Hypoxanthine-guanine phosphoribosyltransferase (HGPRT) (HGPRTase) (EC 2.4.2.8)
Q9Y4L1	Hypoxia up-regulated protein 1 (150 kDa oxygen-regulated protein) (ORP-150) (170 kDa glucose-regulated protein) (GRP-170)
P01876	Immunoglobulin heavy constant alpha 1 (Ig alpha-1 chain C region) (Ig alpha-1 chain C region BUR) (Ig alpha-1 chain C region TRO)
P01859	Immunoglobulin heavy constant gamma 2 (Ig gamma-2 chain C region) (Ig gamma-2 chain C region DOT) (Ig gamma-2 chain C region TIL) (Ig gamma-2 chain C region ZIE)
P01860	Immunoglobulin heavy constant gamma 3 (HDC) (Heavy chain disease protein) (Ig gamma-3 chain C region)
P01861	Immunoglobulin heavy constant gamma 4 (Ig gamma-4 chain C region)
P01871	Immunoglobulin heavy constant mu (Ig mu chain C region) (Ig mu chain C region GAL) (Ig mu chain C region OU)
P01591	Immunoglobulin J chain (Joining chain of multimeric IgA and IgM)
P01834	Immunoglobulin kappa constant (Ig kappa chain C region) (Ig kappa chain C region AG) (Ig kappa chain C region CUM) (Ig kappa chain C region EU) (Ig kappa chain C region OU) (Ig kappa chain C region ROY) (Ig kappa chain C region TI)
P80748	Immunoglobulin lambda variable 3-21 (Ig lambda chain V-III region LOI) (Ig lambda chain V-V region DEL) (Ig lambda chain V-VII region MOT)
Q14974	Importin subunit beta-1 (Importin-90) (Karyopherin subunit beta-1) (Nuclear factor p97) (Pore targeting complex 97 kDa subunit) (PTAC97)
Q15181	Inorganic pyrophosphatase (EC 3.6.1.1) (Pyrophosphate phospho-hydrolase) (PPase)
P05556	Integrin beta-1 (Fibronectin receptor subunit beta) (Glycoprotein IIa) (GPIIA) (VLA-4 subunit beta) (CD antigen CD29)
Q13418	Integrin-linked protein kinase (EC 2.7.11.1) (59 kDa serine/threonine-protein kinase) (ILK-1) (ILK-2) (p59ILK)

P19827	Inter-alpha-trypsin inhibitor heavy chain H1 (ITI heavy chain H1) (ITI-HC1) (Inter-alpha-inhibitor heavy chain 1) (Inter-alpha-trypsin inhibitor complex component III) (Serum-derived hyaluronan-associated protein) (SHAP)
P19823	Inter-alpha-trypsin inhibitor heavy chain H2 (ITI heavy chain H2) (ITI-HC2) (Inter-alpha-inhibitor heavy chain 2) (Inter-alpha-trypsin inhibitor complex component II) (Serum-derived hyaluronan-associated protein) (SHAP)
Q14624	Inter-alpha-trypsin inhibitor heavy chain H4 (ITI heavy chain H4) (ITI-HC4) (Inter-alpha-inhibitor heavy chain 4) (Inter-alpha-trypsin inhibitor family heavy chain-related protein) (IHRP) (Plasma kallikrein sensitive glycoprotein 120) (Gp120) (PK-120) [Cleaved into: 70 kDa inter-alpha-trypsin inhibitor heavy chain H4; 35 kDa inter-alpha-trypsin inhibitor heavy chain H4]
Q12905	Interleukin enhancer-binding factor 2 (Nuclear factor of activated T-cells 45 kDa)
Q12906	Interleukin enhancer-binding factor 3 (Double-stranded RNA-binding protein 76) (DRBP76) (M-phase phosphoprotein 4) (MPP4) (Nuclear factor associated with dsRNA) (NFAR) (Nuclear factor of activated T-cells 90 kDa) (NF-AT-90) (Translational control protein 80) (TCP80)
O75874	Isocitrate dehydrogenase [NADP] cytoplasmic (IDH) (EC 1.1.1.42) (Cytosolic NADP-isocitrate dehydrogenase) (IDP) (NADP(+)-specific ICDH) (Oxalosuccinate decarboxylase)
P53990	IST1 homolog (hIST1) (Putative MAPK-activating protein PM28)
P14923	Junction plakoglobin (Catenin gamma) (Desmoplakin III) (Desmoplakin-3)
P13645	Keratin, type I cytoskeletal 10 (Cytokeratin-10) (CK-10) (Keratin-10) (K10)
P02533	Keratin, type I cytoskeletal 14 (Cytokeratin-14) (CK-14) (Keratin-14) (K14)
Q04695	Keratin, type I cytoskeletal 17 (39.1) (Cytokeratin-17) (CK-17) (Keratin-17) (K17)
P05783	Keratin, type I cytoskeletal 18 (Cell proliferation-inducing gene 46 protein) (Cytokeratin-18) (CK-18) (Keratin-18) (K18)
P08727	Keratin, type I cytoskeletal 19 (Cytokeratin-19) (CK-19) (Keratin-19) (K19)
P35527	Keratin, type I cytoskeletal 9 (Cytokeratin-9) (CK-9) (Keratin-9) (K9)
P04264	Keratin, type II cytoskeletal 1 (67 kDa cytokeratin) (Cytokeratin-1) (CK-1) (Hair alpha protein) (Keratin-1) (K1) (Type-II keratin Kb1)
P13647	Keratin, type II cytoskeletal 5 (58 kDa cytokeratin) (Cytokeratin-5) (CK-5) (Keratin-5) (K5) (Type-II keratin Kb5)
P02538	Keratin, type II cytoskeletal 6A (Cytokeratin-6A) (CK-6A) (Cytokeratin-6D) (CK-6D) (Keratin-6A) (K6A) (Type-II keratin Kb6) (allergen Hom s 5)
P05787	Keratin, type II cytoskeletal 8 (Cytokeratin-8) (CK-8) (Keratin-8) (K8) (Type-II keratin Kb8)
Q9HA64	Ketosamine-3-kinase (EC 2.7.1.-) (Fructosamine-3-kinase-related protein) (FN3K-RP) (FN3K-related protein)
Q86UP2	Kinectin (CG-1 antigen) (Kinesin receptor)
P33176	Kinesin-1 heavy chain (Conventional kinesin heavy chain) (Ubiquitous kinesin heavy chain) (UKHC)

P01042	Kininogen-1 (Alpha-2-thiol proteinase inhibitor) (Fitzgerald factor) (High molecular weight kininogen) (HMWK) (Williams-Fitzgerald-Flaujeac factor) [Cleaved into: Kininogen-1 heavy chain; T-kinin (Ile-Ser-Bradykinin); Bradykinin (Kallidin I); Lysyl-bradykinin (Kallidin II); Kininogen-1 light chain; Low molecular weight growth-promoting factor]
P00338	L-lactate dehydrogenase A chain (LDH-A) (EC 1.1.1.27) (Cell proliferation-inducing gene 19 protein) (LDH muscle subunit) (LDH-M) (Renal carcinoma antigen NY-REN-59)
P07195	L-lactate dehydrogenase B chain (LDH-B) (EC 1.1.1.27) (LDH heart subunit) (LDH-H) (Renal carcinoma antigen NY-REN-46)
Q7Z4W1	L-xylulose reductase (XR) (EC 1.1.1.10) (Carbonyl reductase II) (Dicarbonyl/L-xylulose reductase) (Kidney dicarbonyl reductase) (kiDCR) (Short chain dehydrogenase/reductase family 20C member 1) (Sperm surface protein P34H)
P02788	Lactotransferrin (Lactoferrin) (EC 3.4.21.-) (Growth-inhibiting protein 12) (Talalactoferrin) [Cleaved into: Lactoferricin-H (Lfcin-H); Kaliocin-1; Lactoferroxin-A; Lactoferroxin-B; Lactoferroxin-C]
Q04760	Lactoylglutathione lyase (EC 4.4.1.5) (Aldoketomutase) (Glyoxalase I) (Glx I) (Ketone-aldehyde mutase) (Methylglyoxalase) (S-D-lactoylglutathione methylglyoxal lyase)
P20700	Lamin-B1
Q03252	Lamin-B2
Q16363	Laminin subunit alpha-4 (Laminin-14 subunit alpha) (Laminin-8 subunit alpha) (Laminin-9 subunit alpha)
P07942	Laminin subunit beta-1 (Laminin B1 chain) (Laminin-1 subunit beta) (Laminin-10 subunit beta) (Laminin-12 subunit beta) (Laminin-2 subunit beta) (Laminin-6 subunit beta) (Laminin-8 subunit beta)
P55268	Laminin subunit beta-2 (Laminin B1s chain) (Laminin-11 subunit beta) (Laminin-14 subunit beta) (Laminin-15 subunit beta) (Laminin-3 subunit beta) (Laminin-4 subunit beta) (Laminin-7 subunit beta) (Laminin-9 subunit beta) (S-laminin subunit beta) (S-LAM beta)
P11047	Laminin subunit gamma-1 (Laminin B2 chain) (Laminin-1 subunit gamma) (Laminin-10 subunit gamma) (Laminin-11 subunit gamma) (Laminin-2 subunit gamma) (Laminin-3 subunit gamma) (Laminin-4 subunit gamma) (Laminin-6 subunit gamma) (Laminin-7 subunit gamma) (Laminin-8 subunit gamma) (Laminin-9 subunit gamma) (S-laminin subunit gamma) (S-LAM gamma)
Q9BS40	Latexin (Endogenous carboxypeptidase inhibitor) (ECI) (Protein MUM) (Tissue carboxypeptidase inhibitor) (TCI)
P29536	Leiomodin-1 (64 kDa autoantigen 1D) (64 kDa autoantigen 1D3) (64 kDa autoantigen D1) (Leiomodin, muscle form) (Smooth muscle leiomodin) (SM-Lmod) (Thyroid-associated ophthalmopathy autoantigen)
P02750	Leucine-rich alpha-2-glycoprotein (LRG)
Q32MZ4	Leucine-rich repeat flightless-interacting protein 1 (LRR FLII-interacting protein 1) (GC-binding factor 2) (TAR RNA-interacting protein)

P30740	Leukocyte elastase inhibitor (LEI) (Monocyte/neutrophil elastase inhibitor) (EI) (M/NEI) (Peptidase inhibitor 2) (PI-2) (Serpin B1)
P09960	Leukotriene A-4 hydrolase (LTA-4 hydrolase) (EC 3.3.2.6) (Leukotriene A(4) hydrolase)
Q14847	LIM and SH3 domain protein 1 (LASP-1) (Metastatic lymph node gene 50 protein) (MLN 50)
Q9UHB6	LIM domain and actin-binding protein 1 (Epithelial protein lost in neoplasm)
Q93052	Lipoma-preferred partner (LIM domain-containing preferred translocation partner in lipoma)
P23141	Liver carboxylesterase 1 (Acyl-coenzyme A:cholesterol acyltransferase) (ACAT) (Brain carboxylesterase hBr1) (Carboxylesterase 1) (CE-1) (hCE-1) (EC 3.1.1.1) (Cocaine carboxylesterase) (Egasyn) (HMSE) (Methylumbelliferyl-acetate deacetylase 1) (EC 3.1.1.56) (Monocyte/macrophage serine esterase) (Retinyl ester hydrolase) (REH) (Serine esterase 1) (Triacylglycerol hydrolase) (TGH)
P33121	Long-chain-fatty-acid--CoA ligase 1 (EC 6.2.1.3) (Acyl-CoA synthetase 1) (ACS1) (Long-chain acyl-CoA synthetase 1) (LACS 1) (Long-chain acyl-CoA synthetase 2) (LACS 2) (Long-chain fatty acid-CoA ligase 2) (Palmitoyl-CoA ligase 1) (Palmitoyl-CoA ligase 2)
O95573	Long-chain-fatty-acid--CoA ligase 3 (EC 6.2.1.3) (Long-chain acyl-CoA synthetase 3) (LACS 3)
P24666	Low molecular weight phosphotyrosine protein phosphatase (LMW-PTP) (LMW-PTPase) (EC 3.1.3.48) (Adipocyte acid phosphatase) (Low molecular weight cytosolic acid phosphatase) (EC 3.1.3.2) (Red cell acid phosphatase 1)
P51884	Lumican (Keratan sulfate proteoglycan lumican) (KSPG lumican)
P05455	Lupus La protein (La autoantigen) (La ribonucleoprotein) (Sjoegren syndrome type B antigen) (SS-B)
O00754	Lysosomal alpha-mannosidase (Laman) (EC 3.2.1.24) (Lysosomal acid alpha-mannosidase) (Mannosidase alpha class 2B member 1) (Mannosidase alpha-B) [Cleaved into: Lysosomal alpha-mannosidase A peptide; Lysosomal alpha-mannosidase B peptide; Lysosomal alpha-mannosidase C peptide; Lysosomal alpha-mannosidase D peptide; Lysosomal alpha-mannosidase E peptide]
P11279	Lysosome-associated membrane glycoprotein 1 (LAMP-1) (Lysosome-associated membrane protein 1) (CD107 antigen-like family member A) (CD antigen CD107a)
Q96C86	m7GpppX diphosphatase (EC 3.6.1.59) (DCS-1) (Decapping scavenger enzyme) (Hint-related 7meGMP-directed hydrolase) (Histidine triad nucleotide-binding protein 5) (Histidine triad protein member 5) (HINT-5) (Scavenger mRNA-decapping enzyme DcpS)
P40121	Macrophage-capping protein (Actin regulatory protein CAP-G)
Q14764	Major vault protein (MVP) (Lung resistance-related protein)
P40925	Malate dehydrogenase, cytoplasmic (EC 1.1.1.37) (Cytosolic malate dehydrogenase) (Diiodophenylpyruvate reductase) (EC 1.1.1.96)
P40926	Malate dehydrogenase, mitochondrial (EC 1.1.1.37)
Q14165	Malectin

P49006	MARCKS-related protein (MARCKS-like protein 1) (Macrophage myristoylated alanine-rich C kinase substrate) (Mac-MARCKS) (MacMARCKS)
P15088	Mast cell carboxypeptidase A (MC-CPA) (EC 3.4.17.1) (Carboxypeptidase A3)
O00339	Matrilin-2
P43243	Matrin-3
Q5JRA6	Melanoma inhibitory activity protein 3 (C219-reactive peptide) (D320) (Transport and Golgi organization protein 1)
Q16853	Membrane primary amine oxidase (EC 1.4.3.21) (Copper amine oxidase) (HPAO) (Semicarbazide-sensitive amine oxidase) (SSAO) (Vascular adhesion protein 1) (VAP-1)
O15173	Membrane-associated progesterone receptor component 2 (Progesterone membrane-binding protein) (Steroid receptor protein DG6)
P51608	Methyl-CpG-binding protein 2 (MeCp-2 protein) (MeCp2)
P46821	Microtubule-associated protein 1B (MAP-1B) [Cleaved into: MAP1B heavy chain; MAP1 light chain LC1]
P27816	Microtubule-associated protein 4 (MAP-4)
Q15691	Microtubule-associated protein RP/EB family member 1 (APC-binding protein EB1) (End-binding protein 1) (EB1)
P20774	Mimecan (Osteoglycin) (Osteoinductive factor) (OIF)
Q9Y3D6	Mitochondrial fission 1 protein (FIS1 homolog) (hFis1) (Tetratricopeptide repeat protein 11) (TPR repeat protein 11)
P28482	Mitogen-activated protein kinase 1 (MAP kinase 1) (MAPK 1) (EC 2.7.11.24) (ERT1) (Extracellular signal-regulated kinase 2) (ERK-2) (MAP kinase isoform p42) (p42-MAPK) (Mitogen-activated protein kinase 2) (MAP kinase 2) (MAPK 2)
O43684	Mitotic checkpoint protein BUB3
P26038	Moesin (Membrane-organizing extension spike protein)
P22234	Multifunctional protein ADE2 [Includes: Phosphoribosylaminoimidazole-succinocarboxamide synthase (EC 6.3.2.6) (SAICAR synthetase); Phosphoribosylaminoimidazole carboxylase (EC 4.1.1.21) (AIR carboxylase) (AIRC)]
Q969H8	Myeloid-derived growth factor (MYDGF) (Interleukin-25) (IL-25) (Stromal cell-derived growth factor SF20)
Q9NZM1	Myoferlin (Fer-1-like protein 3)
Q15746	Myosin light chain kinase, smooth muscle (MLCK) (smMLCK) (EC 2.7.11.18) (Kinase-related protein) (KRP) (Telokin) [Cleaved into: Myosin light chain kinase, smooth muscle, deglutamylated form]
P60660	Myosin light polypeptide 6 (17 kDa myosin light chain) (LC17) (Myosin light chain 3) (MLC-3) (Myosin light chain alkali 3) (Myosin light chain A3) (Smooth muscle and nonmuscle myosin light chain alkali 6)

P24844	Myosin regulatory light polypeptide 9 (20 kDa myosin light chain) (LC20) (MLC-2C) (Myosin RLC) (Myosin regulatory light chain 2, smooth muscle isoform) (Myosin regulatory light chain 9) (Myosin regulatory light chain MRLC1)
P35580	Myosin-10 (Cellular myosin heavy chain, type B) (Myosin heavy chain 10) (Myosin heavy chain, non-muscle IIb) (Non-muscle myosin heavy chain B) (NMMHC-B) (Non-muscle myosin heavy chain IIb) (NMMHC II-b) (NMMHC-IIB)
P35579	Myosin-9 (Cellular myosin heavy chain, type A) (Myosin heavy chain 9) (Myosin heavy chain, non-muscle IIa) (Non-muscle myosin heavy chain A) (NMMHC-A) (Non-muscle myosin heavy chain IIa) (NMMHC II-a) (NMMHC-IIA)
P58546	Myotrophin (Protein V-1)
P29966	Myristoylated alanine-rich C-kinase substrate (MARCKS) (Protein kinase C substrate, 80 kDa protein, light chain) (80K-L protein) (PKCSL)
Q9UJ70	N-acetyl-D-glucosamine kinase (N-acetylglucosamine kinase) (EC 2.7.1.59) (GlcNAc kinase)
P15586	N-acetylglucosamine-6-sulfatase (EC 3.1.6.14) (Glucosamine-6-sulfatase) (G6S)
P51688	N-sulphoglucosamine sulphohydrolase (EC 3.10.1.1) (Sulfoglucosamine sulfamidase) (Sulphamidase)
P20933	N(4)-(beta-N-acetylglucosaminyl)-L-asparaginase (EC 3.5.1.26) (Aspartylglucosaminidase) (Glycosylasparaginase) (N4-(N-acetyl-beta-glucosaminyl)-L-asparagine amidase) [Cleaved into: Glycosylasparaginase alpha chain; Glycosylasparaginase beta chain]
O94760	N(G),N(G)-dimethylarginine dimethylaminohydrolase 1 (DDAH-1) (Dimethylarginine dimethylaminohydrolase 1) (EC 3.5.3.18) (DDAHI) (Dimethylargininase-1)
O95865	N(G),N(G)-dimethylarginine dimethylaminohydrolase 2 (DDAH-2) (Dimethylarginine dimethylaminohydrolase 2) (EC 3.5.3.18) (DDAHII) (Dimethylargininase-2) (Protein G6a) (S-phase protein)
O14745	Na(+)/H(+) exchange regulatory cofactor NHE-RF1 (NHERF-1) (Ezrin-radixin-moesin-binding phosphoprotein 50) (EBP50) (Regulatory cofactor of Na(+)/H(+) exchanger) (Sodium-hydrogen exchanger regulatory factor 1) (Solute carrier family 9 isoform A3 regulatory factor 1)
Q8NCW5	NAD(P)H-hydrate epimerase (EC 5.1.99.6) (Apolipoprotein A-I-binding protein) (AI-BP) (NAD(P)HX epimerase) (YjeF N-terminal domain-containing protein 1) (YjeF_N1)
P00387	NADH-cytochrome b5 reductase 3 (B5R) (Cytochrome b5 reductase) (EC 1.6.2.2) (Diaphorase-1) [Cleaved into: NADH-cytochrome b5 reductase 3 membrane-bound form; NADH-cytochrome b5 reductase 3 soluble form]
Q15843	NEDD8 (Neddylin) (Neural precursor cell expressed developmentally down-regulated protein 8) (NEDD-8) (Ubiquitin-like protein Nedd8)
P48681	Nestin

Q9UMX5	Neudesin (Cell immortalization-related protein 2) (Neuron-derived neurotrophic factor) (Protein GIG47) (Secreted protein of unknown function) (SPUF protein)
P84074	Neuron-specific calcium-binding protein hippocalcin (Calcium-binding protein BDR-2)
Q14697	Neutral alpha-glucosidase AB (EC 3.2.1.84) (Alpha-glucosidase 2) (Glucosidase II subunit alpha)
P43490	Nicotinamide phosphoribosyltransferase (NAmPRTase) (Nampt) (EC 2.4.2.12) (Pre-B-cell colony-enhancing factor 1) (Pre-B cell-enhancing factor) (Visfatin)
Q6XQN6	Nicotinate phosphoribosyltransferase (NAPRTase) (EC 6.3.4.21) (FHA-HIT-interacting protein) (Nicotinate phosphoribosyltransferase domain-containing protein 1)
P14543	Nidogen-1 (NID-1) (Entactin)
Q14112	Nidogen-2 (NID-2) (Osteonidogen)
Q9GZT8	NIF3-like protein 1 (Amyotrophic lateral sclerosis 2 chromosomal region candidate gene 1 protein)
P05204	Non-histone chromosomal protein HMG-17 (High mobility group nucleosome-binding domain-containing protein 2)
Q9UNZ2	NSFL1 cofactor p47 (UBX domain-containing protein 2C) (p97 cofactor p47)
Q9Y266	Nuclear migration protein nudC (Nuclear distribution protein C homolog)
Q14980	Nuclear mitotic apparatus protein 1 (Nuclear matrix protein-22) (NMP-22) (Nuclear mitotic apparatus protein) (NuMA protein) (SP-H antigen)
P61970	Nuclear transport factor 2 (NTF-2) (Placental protein 15) (PP15)
P67809	Nuclease-sensitive element-binding protein 1 (CCAAT-binding transcription factor I subunit A) (CBF-A) (DNA-binding protein B) (DBPB) (Enhancer factor I subunit A) (EFI-A) (Y-box transcription factor) (Y-box-binding protein 1) (YB-1)
Q02818	Nucleobindin-1 (CALNUC)
P80303	Nucleobindin-2 (DNA-binding protein NEFA) (Gastric cancer antigen Zg4) (Prepronesfatin) [Cleaved into: Nesfatin-1]
P19338	Nucleolin (Protein C23)
P06748	Nucleophosmin (NPM) (Nucleolar phosphoprotein B23) (Nucleolar protein NO38) (Numatrin)
P12270	Nucleoprotein TPR (Megator) (NPC-associated intranuclear protein) (Translocated promoter region protein)
P15531	Nucleoside diphosphate kinase A (NDK A) (NDP kinase A) (EC 2.7.4.6) (Granzyme A-activated DNase) (GAAD) (Metastasis inhibition factor nm23) (NM23-H1) (Tumor metastatic process-associated protein)
P55209	Nucleosome assembly protein 1-like 1 (NAP-1-related protein) (hNRP)
Q99733	Nucleosome assembly protein 1-like 4 (Nucleosome assembly protein 2) (NAP-2)
Q9NTK5	Obg-like ATPase 1 (DNA damage-regulated overexpressed in cancer 45) (DOC45) (GTP-binding protein 9)
Q9NQR4	Omega-amidase NIT2 (EC 3.5.1.3) (Nitrilase homolog 2)

Q8WX93	Palladin (SIH002) (Sarcoma antigen NY-SAR-77)
P20962	Parathymosin
O00151	PDZ and LIM domain protein 1 (C-terminal LIM domain protein 1) (Elfin) (LIM domain protein CLP-36)
Q96HC4	PDZ and LIM domain protein 5 (Enigma homolog) (Enigma-like PDZ and LIM domains protein)
P62937	Peptidyl-prolyl cis-trans isomerase A (PPIase A) (EC 5.2.1.8) (Cyclophilin A) (Cyclosporin A-binding protein) (Rotamase A) [Cleaved into: Peptidyl-prolyl cis-trans isomerase A, N-terminally processed]
P23284	Peptidyl-prolyl cis-trans isomerase B (PPIase B) (EC 5.2.1.8) (CYP-S1) (Cyclophilin B) (Rotamase B) (S-cyclophilin) (SCYLP)
Q00688	Peptidyl-prolyl cis-trans isomerase FKBP3 (PPIase FKBP3) (EC 5.2.1.8) (25 kDa FK506-binding protein) (25 kDa FKBP) (FKBP-25) (FK506-binding protein 3) (FKBP-3) (Immunophilin FKBP25) (Rapamycin-selective 25 kDa immunophilin) (Rotamase)
Q02790	Peptidyl-prolyl cis-trans isomerase FKBP4 (PPIase FKBP4) (EC 5.2.1.8) (51 kDa FK506-binding protein) (FKBP51) (52 kDa FK506-binding protein) (52 kDa FKBP) (FKBP-52) (59 kDa immunophilin) (p59) (FK506-binding protein 4) (FKBP-4) (FKBP59) (HSP-binding immunophilin) (HBI) (Immunophilin FKBP52) (Rotamase) [Cleaved into: Peptidyl-prolyl cis-trans isomerase FKBP4, N-terminally processed]
O60664	Perilipin-3 (47 kDa mannose 6-phosphate receptor-binding protein) (47 kDa MPR-binding protein) (Cargo selection protein TIP47) (Mannose-6-phosphate receptor-binding protein 1) (Placental protein 17) (PP17)
Q15063	Periostin (PN) (Osteoblast-specific factor 2) (OSF-2)
O60437	Periplakin (190 kDa paraneoplastic pemphigus antigen) (195 kDa cornified envelope precursor protein)
Q06830	Peroxiredoxin-1 (EC 1.11.1.15) (Natural killer cell-enhancing factor A) (NKEF-A) (Proliferation-associated gene protein) (PAG) (Thioredoxin peroxidase 2) (Thioredoxin-dependent peroxide reductase 2)
P32119	Peroxiredoxin-2 (EC 1.11.1.15) (Natural killer cell-enhancing factor B) (NKEF-B) (PRP) (Thiol-specific antioxidant protein) (TSA) (Thioredoxin peroxidase 1) (Thioredoxin-dependent peroxide reductase 1)
P30044	Peroxiredoxin-5, mitochondrial (EC 1.11.1.15) (Alu corepressor 1) (Antioxidant enzyme B166) (AOEB166) (Liver tissue 2D-page spot 71B) (PLP) (Peroxiredoxin V) (Prx-V) (Peroxisomal antioxidant enzyme) (TPx type VI) (Thioredoxin peroxidase PMP20) (Thioredoxin reductase)
P30041	Peroxiredoxin-6 (EC 1.11.1.15) (1-Cys peroxiredoxin) (1-Cys PRX) (24 kDa protein) (Acidic calcium-independent phospholipase A2) (aiPLA2) (EC 3.1.1.-) (Antioxidant protein 2) (Liver 2D page spot 40) (Non-selenium glutathione peroxidase) (NSGPx) (EC 1.11.1.9) (Red blood cells page spot 12)
P51659	Peroxisomal multifunctional enzyme type 2 (MFE-2) (17-beta-hydroxysteroid dehydrogenase 4) (17-beta-HSD 4) (D-bifunctional protein) (DBP) (Multifunctional protein 2) (MPF-2) (Short chain dehydrogenase/reductase family 8C member 1) [Cleaved into: (3R)-hydroxyacyl-CoA dehydrogenase (EC 1.1.1.n12); Enoyl-CoA hydratase 2 (EC 4.2.1.107) (EC 4.2.1.119) (3-alpha,7-alpha,12-alpha-trihydroxy-5-beta-cholest-24-enoyl-CoA hydratase)]

Q00325	Phosphate carrier protein, mitochondrial (Phosphate transport protein) (PTP) (Solute carrier family 25 member 3)
P30086	Phosphatidylethanolamine-binding protein 1 (PEBP-1) (HCNPpp) (Neuropolypeptide h3) (Prostatic-binding protein) (Raf kinase inhibitor protein) (RKIP) [Cleaved into: Hippocampal cholinergic neurostimulating peptide (HCNP)]
O95394	Phosphoacetylglucosamine mutase (PAGM) (EC 5.4.2.3) (Acetylglucosamine phosphomutase) (N-acetylglucosamine-phosphate mutase) (Phosphoglucomutase-3) (PGM 3)
P36871	Phosphoglucomutase-1 (PGM 1) (EC 5.4.2.2) (Glucose phosphomutase 1)
Q96G03	Phosphoglucomutase-2 (PGM 2) (EC 5.4.2.2) (Glucose phosphomutase 2) (Phosphodeoxyribomutase) (Phosphopentomutase) (EC 5.4.2.7)
Q15124	Phosphoglucomutase-like protein 5 (Aciculin) (Phosphoglucomutase-related protein) (PGM-RP)
P00558	Phosphoglycerate kinase 1 (EC 2.7.2.3) (Cell migration-inducing gene 10 protein) (Primer recognition protein 2) (PRP 2)
P18669	Phosphoglycerate mutase 1 (EC 5.4.2.11) (EC 5.4.2.4) (BPG-dependent PGAM 1) (Phosphoglycerate mutase isozyme B) (PGAM-B)
O60256	Phosphoribosyl pyrophosphate synthase-associated protein 2 (PRPP synthase-associated protein 2) (41 kDa phosphoribosypyrophosphate synthetase-associated protein) (PAP41)
P36955	Pigment epithelium-derived factor (PEDF) (Cell proliferation-inducing gene 35 protein) (EPC-1) (Serpin F1)
Q9GZP4	PITH domain-containing protein 1
P23634	Plasma membrane calcium-transporting ATPase 4 (PMCA4) (EC 3.6.3.8) (Matrix-remodeling-associated protein 1) (Plasma membrane calcium ATPase isoform 4) (Plasma membrane calcium pump isoform 4)
P05155	Plasma protease C1 inhibitor (C1 Inh) (C1Inh) (C1 esterase inhibitor) (C1-inhibiting factor) (Serpin G1)
P00747	Plasminogen (EC 3.4.21.7) [Cleaved into: Plasmin heavy chain A; Activation peptide; Angiostatin; Plasmin heavy chain A, short form; Plasmin light chain B]
Q8NC51	Plasminogen activator inhibitor 1 RNA-binding protein (PAI1 RNA-binding protein 1) (PAI-RBP1) (SERPINE1 mRNA-binding protein 1)
P13796	Plastin-2 (L-plastin) (LC64P) (Lymphocyte cytosolic protein 1) (LCP-1)
P13797	Plastin-3 (T-plastin)
Q15149	Plectin (PCN) (PLTN) (Hemidesmosomal protein 1) (HD1) (Plectin-1)
P09874	Poly [ADP-ribose] polymerase 1 (PARP-1) (EC 2.4.2.30) (ADP-ribosyltransferase diphtheria toxin-like 1) (ARTD1) (NAD(+) ADP-ribosyltransferase 1) (ADPRT 1) (Poly[ADP-ribose] synthase 1)
P11940	Polyadenylate-binding protein 1 (PABP-1) (Poly(A)-binding protein 1)
Q86U42	Polyadenylate-binding protein 2 (PABP-2) (Poly(A)-binding protein 2) (Nuclear poly(A)-binding protein 1) (Poly(A)-binding protein II) (PABII) (Polyadenylate-binding nuclear protein 1)

P26599	Polypyrimidine tract-binding protein 1 (PTB) (57 kDa RNA-binding protein PPTB-1) (Heterogeneous nuclear ribonucleoprotein I) (hnRNP I)
O75915	PRA1 family protein 3 (ADP-ribosylation factor-like protein 6-interacting protein 5) (ARL-6-interacting protein 5) (Aip-5) (Cytoskeleton-related vitamin A-responsive protein) (Dermal papilla-derived protein 11) (GTRAP3-18) (Glutamate transporter EAAC1-interacting protein) (JM5) (Prenylated Rab acceptor protein 2) (Protein JWa) (Putative MAPK-activating protein PM27)
P02545	Prelamin-A/C [Cleaved into: Lamin-A/C (70 kDa lamin) (Renal carcinoma antigen NY-REN-32)]
Q9UHG3	Prenylcysteine oxidase 1 (EC 1.8.3.5) (Prenylcysteine lyase)
P07737	Profilin-1 (Epididymis tissue protein Li 184a) (Profilin I)
Q8WUM4	Programmed cell death 6-interacting protein (PDCD6-interacting protein) (ALG-2-interacting protein 1) (ALG-2-interacting protein X) (Hp95)
O14737	Programmed cell death protein 5 (TF-1 cell apoptosis-related protein 19) (Protein TFAR19)
P35232	Prohibitin
P51888	Prolargin (Proline-arginine-rich end leucine-rich repeat protein)
Q9UQ80	Proliferation-associated protein 2G4 (Cell cycle protein p38-2G4 homolog) (hG4-1) (ErbB3-binding protein 1)
Q07954	Prolow-density lipoprotein receptor-related protein 1 (LRP-1) (Alpha-2-macroglobulin receptor) (A2MR) (Apolipoprotein E receptor) (APOER) (CD antigen CD91) [Cleaved into: Low-density lipoprotein receptor-related protein 1 85 kDa subunit (LRP-85); Low-density lipoprotein receptor-related protein 1 515 kDa subunit (LRP-515); Low-density lipoprotein receptor-related protein 1 intracellular domain (LRPICD)]
P48147	Prolyl endopeptidase (PE) (EC 3.4.21.26) (Post-proline cleaving enzyme)
Q16647	Prostacyclin synthase (EC 5.3.99.4) (Prostaglandin I2 synthase)
Q15185	Prostaglandin E synthase 3 (EC 5.3.99.3) (Cytosolic prostaglandin E2 synthase) (cPGES) (Hsp90 co-chaperone) (Progesterone receptor complex p23) (Telomerase-binding protein p23)
Q14914	Prostaglandin reductase 1 (PRG-1) (EC 1.3.1.-) (15-oxoprostaglandin 13-reductase) (EC 1.3.1.48) (NADP-dependent leukotriene B4 12-hydroxydehydrogenase) (EC 1.3.1.74)
Q06323	Proteasome activator complex subunit 1 (11S regulator complex subunit alpha) (REG-alpha) (Activator of multicatalytic protease subunit 1) (Interferon gamma up-regulated I-5111 protein) (IGUP I-5111) (Proteasome activator 28 subunit alpha) (PA28a) (PA28alpha)
Q9UL46	Proteasome activator complex subunit 2 (11S regulator complex subunit beta) (REG-beta) (Activator of multicatalytic protease subunit 2) (Proteasome activator 28 subunit beta) (PA28b) (PA28beta)
P25786	Proteasome subunit alpha type-1 (EC 3.4.25.1) (30 kDa prosomal protein) (PROS-30) (Macropain subunit C2) (Multicatalytic endopeptidase complex subunit C2) (Proteasome component C2) (Proteasome nu chain)
P25787	Proteasome subunit alpha type-2 (EC 3.4.25.1) (Macropain subunit C3) (Multicatalytic endopeptidase complex subunit C3) (Proteasome component C3)

P25788	Proteasome subunit alpha type-3 (EC 3.4.25.1) (Macropain subunit C8) (Multicatalytic endopeptidase complex subunit C8) (Proteasome component C8)
P25789	Proteasome subunit alpha type-4 (EC 3.4.25.1) (Macropain subunit C9) (Multicatalytic endopeptidase complex subunit C9) (Proteasome component C9) (Proteasome subunit L)
P28066	Proteasome subunit alpha type-5 (EC 3.4.25.1) (Macropain zeta chain) (Multicatalytic endopeptidase complex zeta chain) (Proteasome zeta chain)
P60900	Proteasome subunit alpha type-6 (EC 3.4.25.1) (27 kDa prosomal protein) (PROS-27) (p27K) (Macropain iota chain) (Multicatalytic endopeptidase complex iota chain) (Proteasome iota chain)
O14818	Proteasome subunit alpha type-7 (EC 3.4.25.1) (Proteasome subunit RC6-1) (Proteasome subunit XAPC7)
P20618	Proteasome subunit beta type-1 (EC 3.4.25.1) (Macropain subunit C5) (Multicatalytic endopeptidase complex subunit C5) (Proteasome component C5) (Proteasome gamma chain)
P40306	Proteasome subunit beta type-10 (EC 3.4.25.1) (Low molecular mass protein 10) (Macropain subunit MECl-1) (Multicatalytic endopeptidase complex subunit MECl-1) (Proteasome MECl-1) (Proteasome subunit beta-2i)
P28070	Proteasome subunit beta type-4 (EC 3.4.25.1) (26 kDa prosomal protein) (HsBPROS26) (PROS-26) (Macropain beta chain) (Multicatalytic endopeptidase complex beta chain) (Proteasome beta chain) (Proteasome chain 3) (HsN3)
P28074	Proteasome subunit beta type-5 (EC 3.4.25.1) (Macropain epsilon chain) (Multicatalytic endopeptidase complex epsilon chain) (Proteasome chain 6) (Proteasome epsilon chain) (Proteasome subunit MB1) (Proteasome subunit X)
P28072	Proteasome subunit beta type-6 (EC 3.4.25.1) (Macropain delta chain) (Multicatalytic endopeptidase complex delta chain) (Proteasome delta chain) (Proteasome subunit Y)
Q99436	Proteasome subunit beta type-7 (EC 3.4.25.1) (Macropain chain Z) (Multicatalytic endopeptidase complex chain Z) (Proteasome subunit Z)
P28065	Proteasome subunit beta type-9 (EC 3.4.25.1) (Low molecular mass protein 2) (Macropain chain 7) (Multicatalytic endopeptidase complex chain 7) (Proteasome chain 7) (Proteasome subunit beta-1i) (Really interesting new gene 12 protein)
P11171	Protein 4.1 (P4.1) (4.1R) (Band 4.1) (EPB4.1)
Q96IU4	Protein ABHD14B (EC 3.-.-.-) (Alpha/beta hydrolase domain-containing protein 14B) (Abhydrolase domain-containing protein 14B) (CCG1-interacting factor B)
O14744	Protein arginine N-methyltransferase 5 (EC 2.1.1.320) (72 kDa ICln-binding protein) (Histone-arginine N-methyltransferase PRMT5) (Jak-binding protein 1) (Shk1 kinase-binding protein 1 homolog) (SKB1 homolog) (SKB1Hs) [Cleaved into: Protein arginine N-methyltransferase 5, N-terminally processed]
Q9Y2B0	Protein canopy homolog 2 (MIR-interacting saposin-like protein) (Putative secreted protein Zsig9) (Transmembrane protein 4)

Q9UKY7	Protein CDV3 homolog
Q5TDH0	Protein DDI1 homolog 2
P07237	Protein disulfide-isomerase (PDI) (EC 5.3.4.1) (Cellular thyroid hormone-binding protein) (Prolyl 4-hydroxylase subunit beta) (p55)
P30101	Protein disulfide-isomerase A3 (EC 5.3.4.1) (58 kDa glucose-regulated protein) (58 kDa microsomal protein) (p58) (Disulfide isomerase ER-60) (Endoplasmic reticulum resident protein 57) (ER protein 57) (ERp57) (Endoplasmic reticulum resident protein 60) (ER protein 60) (ERp60)
P13667	Protein disulfide-isomerase A4 (EC 5.3.4.1) (Endoplasmic reticulum resident protein 70) (ER protein 70) (ERp70) (Endoplasmic reticulum resident protein 72) (ER protein 72) (ERp-72) (ERp72)
Q15084	Protein disulfide-isomerase A6 (EC 5.3.4.1) (Endoplasmic reticulum protein 5) (ER protein 5) (ERp5) (Protein disulfide isomerase P5) (Thioredoxin domain-containing protein 7)
Q99497	Protein DJ-1 (DJ-1) (Oncogene DJ1) (Parkinson disease protein 7) (Parkinsonism-associated deglycase) (Protein deglycase DJ-1) (EC 3.1.2.-) (EC 3.5.1.124)
Q9NUQ9	Protein FAM49B (L1)
Q9BZQ8	Protein Niban (Cell growth-inhibiting gene 39 protein) (Protein FAM129A)
O14974	Protein phosphatase 1 regulatory subunit 12A (Myosin phosphatase-targeting subunit 1) (Myosin phosphatase target subunit 1) (Protein phosphatase myosin-binding subunit)
Q15435	Protein phosphatase 1 regulatory subunit 7 (Protein phosphatase 1 regulatory subunit 22)
Q9Y570	Protein phosphatase methylesterase 1 (PME-1) (EC 3.1.1.89)
P29590	Protein PML (Promyelocytic leukemia protein) (RING finger protein 71) (Tripartite motif-containing protein 19)
Q9P258	Protein RCC2 (RCC1-like protein TD-60) (Telophase disk protein of 60 kDa)
P60903	Protein S100-A10 (Calpactin I light chain) (Calpactin-1 light chain) (Cellular ligand of annexin II) (S100 calcium-binding protein A10) (p10 protein) (p11)
P31949	Protein S100-A11 (Calgizzarin) (Metastatic lymph node gene 70 protein) (MLN 70) (Protein S100-C) (S100 calcium-binding protein A11) [Cleaved into: Protein S100-A11, N-terminally processed]
P05109	Protein S100-A8 (Calgranulin-A) (Calprotectin L1L subunit) (Cystic fibrosis antigen) (CFAG) (Leukocyte L1 complex light chain) (Migration inhibitory factor-related protein 8) (MRP-8) (p8) (S100 calcium-binding protein A8) (Urinary stone protein band A) [Cleaved into: Protein S100-A8, N-terminally processed]
P06702	Protein S100-A9 (Calgranulin-B) (Calprotectin L1H subunit) (Leukocyte L1 complex heavy chain) (Migration inhibitory factor-related protein 14) (MRP-14) (p14) (S100 calcium-binding protein A9)
Q01105	Protein SET (HLA-DR-associated protein II) (Inhibitor of granzyme A-activated DNase) (IGAAD) (PHAPII) (Phosphatase 2A inhibitor I2PP2A) (I-2PP2A) (Template-activating factor I) (TAF-I)
Q92734	Protein TFG (TRK-fused gene protein)

O94979	Protein transport protein Sec31A (ABP125) (ABP130) (SEC31-like protein 1) (SEC31-related protein A) (Web1-like protein)
P21980	Protein-glutamine gamma-glutamyltransferase 2 (EC 2.3.2.13) (Tissue transglutaminase) (Transglutaminase C) (TG(C)) (TGC) (TGase C) (Transglutaminase H) (TGase H) (Transglutaminase-2) (TGase-2)
P22061	Protein-L-isoaspartate(D-aspartate) O-methyltransferase (PIMT) (EC 2.1.1.77) (L-isoaspartyl protein carboxyl methyltransferase) (Protein L-isoaspartyl/D-aspartyl methyltransferase) (Protein-beta-aspartate methyltransferase)
P00734	Prothrombin (EC 3.4.21.5) (Coagulation factor II) [Cleaved into: Activation peptide fragment 1; Activation peptide fragment 2; Thrombin light chain; Thrombin heavy chain]
P06454	Prothymosin alpha [Cleaved into: Prothymosin alpha, N-terminally processed; Thymosin alpha-1]
P55786	Puromycin-sensitive aminopeptidase (PSA) (EC 3.4.11.14) (Cytosol alanyl aminopeptidase) (AAP-S)
O00764	Pyridoxal kinase (EC 2.7.1.35) (Pyridoxine kinase)
Q6P996	Pyridoxal-dependent decarboxylase domain-containing protein 1 (EC 4.1.1.-)
P08559	Pyruvate dehydrogenase E1 component subunit alpha, somatic form, mitochondrial (EC 1.2.4.1) (PDHE1-A type I)
P11177	Pyruvate dehydrogenase E1 component subunit beta, mitochondrial (PDHE1-B) (EC 1.2.4.1)
P14618	Pyruvate kinase PKM (EC 2.7.1.40) (Cytosolic thyroid hormone-binding protein) (CTHBP) (Opa-interacting protein 3) (OIP-3) (Pyruvate kinase 2/3) (Pyruvate kinase muscle isozyme) (Thyroid hormone-binding protein 1) (THBP1) (Tumor M2-PK) (p58)
Q08257	Quinone oxidoreductase (EC 1.6.5.5) (NADPH:quinone reductase) (Zeta-crystallin)
P31150	Rab GDP dissociation inhibitor alpha (Rab GDI alpha) (Guanosine diphosphate dissociation inhibitor 1) (GDI-1) (Oligophrenin-2) (Protein XAP-4)
P43487	Ran-specific GTPase-activating protein (Ran-binding protein 1) (RanBP1)
P46940	Ras GTPase-activating-like protein IQGAP1 (p195)
Q13576	Ras GTPase-activating-like protein IQGAP2
Q15404	Ras suppressor protein 1 (RSP-1) (Rsu-1)
P10301	Ras-related protein R-Ras (p23)
P61026	Ras-related protein Rab-10
Q9UL25	Ras-related protein Rab-21
P61019	Ras-related protein Rab-2A
P51149	Ras-related protein Rab-7a
P11233	Ras-related protein Ral-A

P63244	Receptor of activated protein C kinase 1 (Cell proliferation-inducing gene 21 protein) (Guanine nucleotide-binding protein subunit beta-2-like 1) (Guanine nucleotide-binding protein subunit beta-like protein 12.3) (Human lung cancer oncogene 7 protein) (HLC-7) (Receptor for activated C kinase) (Small ribosomal subunit protein RACK1) [Cleaved into: Receptor of activated protein C kinase 1, N-terminally processed (Guanine nucleotide-binding protein subunit beta-2-like 1, N-terminally processed)]
P08575	Receptor-type tyrosine-protein phosphatase C (EC 3.1.3.48) (Leukocyte common antigen) (L-CA) (T200) (CD antigen CD45)
Q9BRX8	Redox-regulatory protein FAM213A (Peroxiredoxin-like 2 activated in M-CSF stimulated monocytes) (Protein PAMM)
Q15293	Reticulocalbin-1
Q9NQC3	Reticulon-4 (Foocen) (Neurite outgrowth inhibitor) (Nogo protein) (Neuroendocrine-specific protein) (NSP) (Neuroendocrine-specific protein C homolog) (RTN-x) (Reticulon-5)
P00352	Retinal dehydrogenase 1 (RALDH 1) (RalDH1) (EC 1.2.1.-) (EC 1.2.1.36) (ALDH-E1) (ALHDII) (Aldehyde dehydrogenase family 1 member A1) (Aldehyde dehydrogenase, cytosolic)
P52565	Rho GDP-dissociation inhibitor 1 (Rho GDI 1) (Rho-GDI alpha)
P52566	Rho GDP-dissociation inhibitor 2 (Rho GDI 2) (Ly-GDI) (Rho-GDI beta)
Q07960	Rho GTPase-activating protein 1 (CDC42 GTPase-activating protein) (GTPase-activating protein rhoGAP) (Rho-related small GTPase protein activator) (Rho-type GTPase-activating protein 1) (p50-RhoGAP)
P84095	Rho-related GTP-binding protein RhoG
P13489	Ribonuclease inhibitor (Placental ribonuclease inhibitor) (Placental RNase inhibitor) (Ribonuclease/angiogenin inhibitor 1) (RAI)
P60891	Ribose-phosphate pyrophosphokinase 1 (EC 2.7.6.1) (PPRibP) (Phosphoribosyl pyrophosphate synthase I) (PRS-I)
Q9Y3A5	Ribosome maturation protein SBDS (Shwachman-Bodian-Diamond syndrome protein)
Q9P2E9	Ribosome-binding protein 1 (180 kDa ribosome receptor homolog) (RRp) (ES/130-related protein) (Ribosome receptor protein)
P38159	RNA-binding motif protein, X chromosome (Glycoprotein p43) (Heterogeneous nuclear ribonucleoprotein G) (hnRNP G) [Cleaved into: RNA-binding motif protein, X chromosome, N-terminally processed]
P98179	RNA-binding protein 3 (RNA-binding motif protein 3) (RNPL)
Q01844	RNA-binding protein EWS (EWS oncogene) (Ewing sarcoma breakpoint region 1 protein)
P35637	RNA-binding protein FUS (75 kDa DNA-pairing protein) (Oncogene FUS) (Oncogene TLS) (POMp75) (Translocated in liposarcoma protein)
Q9UKM9	RNA-binding protein Raly (Autoantigen p542) (Heterogeneous nuclear ribonucleoprotein C-like 2) (hnRNP core protein C-like 2) (hnRNP associated with lethal yellow protein homolog)

Q9Y265	RuvB-like 1 (EC 3.6.4.12) (49 kDa TATA box-binding protein-interacting protein) (49 kDa TBP-interacting protein) (54 kDa erythrocyte cytosolic protein) (ECP-54) (INO80 complex subunit H) (Nuclear matrix protein 238) (NMP 238) (Pontin 52) (TIP49a) (TIP60-associated protein 54-alpha) (TAP54-alpha)
Q9Y230	RuvB-like 2 (EC 3.6.4.12) (48 kDa TATA box-binding protein-interacting protein) (48 kDa TBP-interacting protein) (51 kDa erythrocyte cytosolic protein) (ECP-51) (INO80 complex subunit J) (Repressing pontin 52) (Reptin 52) (TIP49b) (TIP60-associated protein 54-beta) (TAP54-beta)
P10768	S-formylglutathione hydrolase (FGH) (EC 3.1.2.12) (Esterase D) (Methylumbelliferyl-acetate deacetylase) (EC 3.1.1.56)
Q13126	S-methyl-5'-thioadenosine phosphorylase (EC 2.4.2.28) (5'-methylthioadenosine phosphorylase) (MTA phosphorylase) (MTAP) (MTAPase)
P82979	SAP domain-containing ribonucleoprotein (Cytokine-induced protein of 29 kDa) (Nuclear protein Hcc-1) (Proliferation-associated cytokine-inducible protein CIP29)
Q15424	Scaffold attachment factor B1 (SAF-B) (SAF-B1) (HSP27 estrogen response element-TATA box-binding protein) (HSP27 ERE-TATA-binding protein)
Q12765	Secernin-1
Q96FV2	Secernin-2
Q13228	Selenium-binding protein 1 (56 kDa selenium-binding protein) (SBP56) (SP56)
Q9NVA2	Septin-11
Q15019	Septin-2 (Neural precursor cell expressed developmentally down-regulated protein 5) (NEDD-5)
Q16181	Septin-7 (CDC10 protein homolog)
Q9UHD8	Septin-9 (MLL septin-like fusion protein MSF-A) (MLL septin-like fusion protein) (Ovarian/Breast septin) (Ov/Br septin) (Septin D1)
P49591	Serine--tRNA ligase, cytoplasmic (EC 6.1.1.11) (Seryl-tRNA synthetase) (SerRS) (Seryl-tRNA(Ser/Sec) synthetase)
Q9Y3F4	Serine-threonine kinase receptor-associated protein (MAP activator with WD repeats) (UNR-interacting protein) (WD-40 repeat protein PT-WD)
Q07955	Serine/arginine-rich splicing factor 1 (Alternative-splicing factor 1) (ASF-1) (Splicing factor, arginine/serine-rich 1) (pre-mRNA-splicing factor SF2, P33 subunit)
O75494	Serine/arginine-rich splicing factor 10 (40 kDa SR-repressor protein) (SRrp40) (FUS-interacting serine-arginine-rich protein 1) (Splicing factor SRp38) (Splicing factor, arginine/serine-rich 13A) (TLS-associated protein with Ser-Arg repeats) (TASR) (TLS-associated protein with SR repeats) (TLS-associated serine-arginine protein) (TLS-associated SR protein)
P84103	Serine/arginine-rich splicing factor 3 (Pre-mRNA-splicing factor SRP20) (Splicing factor, arginine/serine-rich 3)

O95747	Serine/threonine-protein kinase OSR1 (EC 2.7.11.1) (Oxidative stress-responsive 1 protein)
Q13177	Serine/threonine-protein kinase PAK 2 (EC 2.7.11.1) (Gamma-PAK) (PAK65) (S6/H4 kinase) (p21-activated kinase 2) (PAK-2) (p58) [Cleaved into: PAK-2p27 (p27); PAK-2p34 (p34) (C-t-PAK2)]
P63151	Serine/threonine-protein phosphatase 2A 55 kDa regulatory subunit B alpha isoform (PP2A subunit B isoform B55-alpha) (PP2A subunit B isoform PR55-alpha) (PP2A subunit B isoform R2-alpha) (PP2A subunit B isoform alpha)
P30153	Serine/threonine-protein phosphatase 2A 65 kDa regulatory subunit A alpha isoform (Medium tumor antigen-associated 61 kDa protein) (PP2A subunit A isoform PR65-alpha) (PP2A subunit A isoform R1-alpha)
Q15257	Serine/threonine-protein phosphatase 2A activator (EC 5.2.1.8) (PP2A, subunit B', PR53 isoform) (Phosphotyrosyl phosphatase activator) (PTPA) (Serine/threonine-protein phosphatase 2A regulatory subunit 4) (Serine/threonine-protein phosphatase 2A regulatory subunit B')
P62140	Serine/threonine-protein phosphatase PP1-beta catalytic subunit (PP-1B) (PPP1CD) (EC 3.1.3.16) (EC 3.1.3.53)
P02787	Serotransferrin (Transferrin) (Beta-1 metal-binding globulin) (Siderophilin)
P35237	Serpin B6 (Cytoplasmic antiproteinase) (CAP) (Peptidase inhibitor 6) (PI-6) (Placental thrombin inhibitor)
P50454	Serpin H1 (47 kDa heat shock protein) (Arsenic-transactivated protein 3) (AsTP3) (Cell proliferation-inducing gene 14 protein) (Collagen-binding protein) (Colligin) (Rheumatoid arthritis-related antigen RA-A47)
P02768-1	Serum albumin
P02743	Serum amyloid P-component (SAP) (9.5S alpha-1-glycoprotein) [Cleaved into: Serum amyloid P-component(1-203)]
O75368	SH3 domain-binding glutamic acid-rich-like protein
Q9H299	SH3 domain-binding glutamic acid-rich-like protein 3 (SH3 domain-binding protein 1) (SH3BP-1)
Q9NR45	Sialic acid synthase (N-acetylneuraminate synthase) (EC 2.5.1.56) (N-acetylneuraminate-9-phosphate synthase) (EC 2.5.1.57) (N-acetylneuraminic acid phosphate synthase) (N-acetylneuraminic acid synthase)
P42224	Signal transducer and activator of transcription 1-alpha/beta (Transcription factor ISGF-3 components p91/p84)
Q04837	Single-stranded DNA-binding protein, mitochondrial (Mt-SSB) (MtSSB) (PWP1-interacting protein 17)
P53814	Smoothelin
P11166	Solute carrier family 2, facilitated glucose transporter member 1 (Glucose transporter type 1, erythrocyte/brain) (GLUT-1) (HepG2 glucose transporter)
Q9BX66	Sorbin and SH3 domain-containing protein 1 (Ponsin) (SH3 domain protein 5) (SH3P12) (c-Cbl-associated protein) (CAP)
O94875	Sorbin and SH3 domain-containing protein 2 (Arg-binding protein 2) (ArgBP2) (Arg/Abl-interacting protein 2) (Sorbin)

Q00796	Sorbitol dehydrogenase (EC 1.1.1.14) (L-iditol 2-dehydrogenase)
P30626	Sorcin (22 kDa protein) (CP-22) (CP22) (V19)
Q13596	Sorting nexin-1
O60749	Sorting nexin-2 (Transformation-related gene 9 protein) (TRG-9)
Q9UNH7	Sorting nexin-6 (TRAF4-associated factor 2) [Cleaved into: Sorting nexin-6, N-terminally processed]
Q13813	Spectrin alpha chain, non-erythrocytic 1 (Alpha-II spectrin) (Fodrin alpha chain) (Spectrin, non-erythroid alpha subunit)
Q01082	Spectrin beta chain, non-erythrocytic 1 (Beta-II spectrin) (Fodrin beta chain) (Spectrin, non-erythroid beta chain 1)
Q13838	Spliceosome RNA helicase DDX39B (EC 3.6.4.13) (56 kDa U2AF65-associated protein) (ATP-dependent RNA helicase p47) (DEAD box protein UAP56) (HLA-B-associated transcript 1 protein)
Q15637	Splicing factor 1 (Mammalian branch point-binding protein) (BBP) (mBBP) (Transcription factor ZFM1) (Zinc finger gene in MEN1 locus) (Zinc finger protein 162)
Q15459	Splicing factor 3A subunit 1 (SF3a120) (Spliceosome-associated protein 114) (SAP 114)
Q15427	Splicing factor 3B subunit 4 (Pre-mRNA-splicing factor SF3b 49 kDa subunit) (SF3b50) (Spliceosome-associated protein 49) (SAP 49)
P23246	Splicing factor, proline- and glutamine-rich (100 kDa DNA-pairing protein) (hPOMp100) (DNA-binding p52/p100 complex, 100 kDa subunit) (Polypyrimidine tract-binding protein-associated-splicing factor) (PSF) (PTB-associated-splicing factor)
Q14247	Src substrate cortactin (Amplaxin) (Oncogene EMS1)
Q7KZF4	Staphylococcal nuclease domain-containing protein 1 (100 kDa coactivator) (EBNA2 coactivator p100) (Tudor domain-containing protein 11) (p100 co-activator)
P38646	Stress-70 protein, mitochondrial (75 kDa glucose-regulated protein) (GRP-75) (Heat shock 70 kDa protein 9) (Mortalin) (MOT) (Peptide-binding protein 74) (PBP74)
P31948	Stress-induced-phosphoprotein 1 (STI1) (Hsc70/Hsp90-organizing protein) (Hop) (Renal carcinoma antigen NY-REN-11) (Transformation-sensitive protein IEF SSP 3521)
P31040	Succinate dehydrogenase [ubiquinone] flavoprotein subunit, mitochondrial (EC 1.3.5.1) (Flavoprotein subunit of complex II) (Fp)
Q96I99	Succinate--CoA ligase [GDP-forming] subunit beta, mitochondrial (EC 6.2.1.4) (GTP-specific succinyl-CoA synthetase subunit beta) (G-SCS) (GTPSCS) (Succinyl-CoA synthetase beta-G chain) (SCS-betaG)
P55809	Succinyl-CoA:3-ketoacid coenzyme A transferase 1, mitochondrial (EC 2.8.3.5) (3-oxoacid CoA-transferase 1) (Somatic-type succinyl-CoA:3-oxoacid CoA-transferase) (SCOT-s)
Q8NBJ7	Sulfatase-modifying factor 2 (C-alpha-formylglycine-generating enzyme 2)
P00441	Superoxide dismutase [Cu-Zn] (EC 1.15.1.1) (Superoxide dismutase 1) (hSod1)

P04179	Superoxide dismutase [Mn], mitochondrial (EC 1.15.1.1)
Q99536	Synaptic vesicle membrane protein VAT-1 homolog (EC 1.-.-.-)
Q9UMS6	Synaptopodin-2 (Genethonin-2) (Myopodin)
O15061	Synemin (Desmuslin)
O15400	Syntaxin-7
P17987	T-complex protein 1 subunit alpha (TCP-1-alpha) (CCT-alpha)
P78371	T-complex protein 1 subunit beta (TCP-1-beta) (CCT-beta)
P50991	T-complex protein 1 subunit delta (TCP-1-delta) (CCT-delta) (Stimulator of TAR RNA-binding)
P48643	T-complex protein 1 subunit epsilon (TCP-1-epsilon) (CCT-epsilon)
Q99832	T-complex protein 1 subunit eta (TCP-1-eta) (CCT-eta) (HIV-1 Nef-interacting protein) [Cleaved into: T-complex protein 1 subunit eta, N-terminally processed]
P49368	T-complex protein 1 subunit gamma (TCP-1-gamma) (CCT-gamma) (hTRiC5)
P50990	T-complex protein 1 subunit theta (TCP-1-theta) (CCT-theta) (Renal carcinoma antigen NY-REN-15)
P40227	T-complex protein 1 subunit zeta (TCP-1-zeta) (Acute morphine dependence-related protein 2) (CCT-zeta-1) (HTR3) (Tcp20)
Q9Y490	Talin-1
P24821	Tenascin (TN) (Cytotactin) (GMEM) (GP 150-225) (Glioma-associated-extracellular matrix antigen) (Hexabrachion) (JI) (Myotendinous antigen) (Neuronectin) (Tenascin-C) (TN-C)
P22105	Tenascin-X (TN-X) (Hexabrachion-like protein)
P10599	Thioredoxin (Trx) (ATL-derived factor) (ADF) (Surface-associated sulphydryl protein) (SASP)
Q9BRA2	Thioredoxin domain-containing protein 17 (14 kDa thioredoxin-related protein) (TRP14) (Protein 42-9-9) (Thioredoxin-like protein 5)
Q8NBS9	Thioredoxin domain-containing protein 5 (Endoplasmic reticulum resident protein 46) (ER protein 46) (ERp46) (Thioredoxin-like protein p46)
Q16881	Thioredoxin reductase 1, cytoplasmic (TR) (EC 1.8.1.9) (Gene associated with retinoic and interferon-induced mortality 12 protein) (GRIM-12) (Gene associated with retinoic and IFN-induced mortality 12 protein) (KM-102-derived reductase-like factor) (Thioredoxin reductase TR1)
P30048	Thioredoxin-dependent peroxide reductase, mitochondrial (EC 1.11.1.15) (Antioxidant protein 1) (AOP-1) (HBC189) (Peroxiredoxin III) (Prx-III) (Peroxiredoxin-3) (Protein MER5 homolog)
O43396	Thioredoxin-like protein 1 (32 kDa thioredoxin-related protein)
Q16762	Thiosulfate sulfurtransferase (EC 2.8.1.1) (Rhodanese)
P07996	Thrombospondin-1
P35443	Thrombospondin-4

P19971	Thymidine phosphorylase (TP) (EC 2.4.2.4) (Gliostatin) (Platelet-derived endothelial cell growth factor) (PD-ECGF) (TdRPase)
P62328	Thymosin beta-4 (T beta-4) (Fx) [Cleaved into: Hematopoietic system regulatory peptide (Seraspenide)]
Q9Y2W1	Thyroid hormone receptor-associated protein 3 (Thyroid hormone receptor-associated protein complex 150 kDa component) (Trap150)
Q07157	Tight junction protein ZO-1 (Tight junction protein 1) (Zona occludens protein 1) (Zonula occludens protein 1)
Q12888	TP53-binding protein 1 (53BP1) (p53-binding protein 1) (p53BP1)
P37837	Transaldolase (EC 2.2.1.2)
Q969E4	Transcription elongation factor A protein-like 3 (TCEA-like protein 3) (Transcription elongation factor S-II protein-like 3)
Q00059	Transcription factor A, mitochondrial (mtTFA) (Mitochondrial transcription factor 1) (MtTF1) (Transcription factor 6) (TCF-6) (Transcription factor 6-like 2)
Q13263	Transcription intermediary factor 1-beta (TIF1-beta) (E3 SUMO-protein ligase TRIM28) (EC 2.3.2.27) (KRAB-associated protein 1) (KAP-1) (KRAB-interacting protein 1) (KRIP-1) (Nuclear corepressor KAP-1) (RING finger protein 96) (RING-type E3 ubiquitin transferase TIF1-beta) (Tripartite motif-containing protein 28)
Q00577	Transcriptional activator protein Pur-alpha (Purine-rich single-stranded DNA-binding protein alpha)
Q15582	Transforming growth factor-beta-induced protein ig-h3 (Beta ig-h3) (Kerato-epithelin) (RGD-containing collagen-associated protein) (RGD-CAP)
P61586	Transforming protein RhoA (Rho cDNA clone 12) (h12)
Q01995	Transgelin (22 kDa actin-binding protein) (Protein WS3-10) (Smooth muscle protein 22-alpha) (SM22-alpha)
P37802	Transgelin-2 (Epididymis tissue protein Li 7e) (SM22-alpha homolog)
P55072	Transitional endoplasmic reticulum ATPase (TER ATPase) (EC 3.6.4.6) (15S Mg(2+)-ATPase p97 subunit) (Valosin-containing protein) (VCP)
P29401	Transketolase (TK) (EC 2.2.1.1)
P13693	Translationally-controlled tumor protein (TCTP) (Fortilin) (Histamine-releasing factor) (HRF) (p23)
Q15631	Translin (EC 3.1.-.-) (Component 3 of promoter of RISC) (C3PO)
P02766	Transthyretin (ATTR) (Prealbumin) (TBPA)
P40939	Trifunctional enzyme subunit alpha, mitochondrial (78 kDa gastrin-binding protein) (TP-alpha) [Includes: Long-chain enoyl-CoA hydratase (EC 4.2.1.17); Long chain 3-hydroxyacyl-CoA dehydrogenase (EC 1.1.1.211)]
P55084	Trifunctional enzyme subunit beta, mitochondrial (TP-beta) [Includes: 3-ketoacyl-CoA thiolase (EC 2.3.1.16) (Acetyl-CoA acyltransferase) (Beta-ketothiolase)]
P60174	Triosephosphate isomerase (TIM) (EC 5.3.1.1) (Triose-phosphate isomerase)
Q9NYL9	Tropomodulin-3 (Ubiquitous tropomodulin) (U-Tmod)

P09493	Tropomyosin alpha-1 chain (Alpha-tropomyosin) (Tropomyosin-1)
P06753	Tropomyosin alpha-3 chain (Gamma-tropomyosin) (Tropomyosin-3) (Tropomyosin-5) (hTM5)
P67936	Tropomyosin alpha-4 chain (TM30p1) (Tropomyosin-4)
P07951	Tropomyosin beta chain (Beta-tropomyosin) (Tropomyosin-2)
Q15661	Tryptase alpha/beta-1 (Tryptase-1) (EC 3.4.21.59) (Tryptase I) (Tryptase alpha-1)
P23381	Tryptophan--tRNA ligase, cytoplasmic (EC 6.1.1.2) (Interferon-induced protein 53) (IFP53) (Tryptophanyl-tRNA synthetase) (TrpRS) (hWRS) [Cleaved into: T1-TrpRS; T2-TrpRS]
P68363	Tubulin alpha-1B chain (Alpha-tubulin ubiquitous) (Tubulin K-alpha-1) (Tubulin alpha-ubiquitous chain) [Cleaved into: Detyrosinated tubulin alpha-1B chain]
P68366	Tubulin alpha-4A chain (Alpha-tubulin 1) (Testis-specific alpha-tubulin) (Tubulin H2-alpha) (Tubulin alpha-1 chain)
P07437	Tubulin beta chain (Tubulin beta-5 chain)
Q13885	Tubulin beta-2A chain (Tubulin beta class IIa)
P68371	Tubulin beta-4B chain (Tubulin beta-2 chain) (Tubulin beta-2C chain)
O75347	Tubulin-specific chaperone A (TCP1-chaperonin cofactor A) (Tubulin-folding cofactor A) (CFA)
Q06124	Tyrosine-protein phosphatase non-receptor type 11 (EC 3.1.3.48) (Protein-tyrosine phosphatase 1D) (PTP-1D) (Protein-tyrosine phosphatase 2C) (PTP-2C) (SH-PTP2) (SHP-2) (Shp2) (SH-PTP3)
P29350	Tyrosine-protein phosphatase non-receptor type 6 (EC 3.1.3.48) (Hematopoietic cell protein-tyrosine phosphatase) (Protein-tyrosine phosphatase 1C) (PTP-1C) (Protein-tyrosine phosphatase SHP-1) (SH-PTP1)
O75643	U5 small nuclear ribonucleoprotein 200 kDa helicase (EC 3.6.4.13) (Activating signal cointegrator 1 complex subunit 3-like 1) (BRR2 homolog) (U5 snRNP-specific 200 kDa protein) (U5-200KD)
O95777	U6 snRNA-associated Sm-like protein LSm8
Q9UMX0	Ubiquilin-1 (Protein linking IAP with cytoskeleton 1) (PLIC-1) (hPLIC-1)
P54578	Ubiquitin carboxyl-terminal hydrolase 14 (EC 3.4.19.12) (Deubiquitinating enzyme 14) (Ubiquitin thioesterase 14) (Ubiquitin-specific-processing protease 14)
P45974	Ubiquitin carboxyl-terminal hydrolase 5 (EC 3.4.19.12) (Deubiquitinating enzyme 5) (Isopeptidase T) (Ubiquitin thioesterase 5) (Ubiquitin-specific-processing protease 5)
Q92890	Ubiquitin recognition factor in ER-associated degradation protein 1 (Ubiquitin fusion degradation protein 1) (UB fusion protein 1)
Q96FW1	Ubiquitin thioesterase OTUB1 (EC 3.4.19.12) (Deubiquitinating enzyme OTUB1) (OTU domain-containing ubiquitin aldehyde-binding protein 1) (Otubain-1) (hOTU1) (Ubiquitin-specific-processing protease OTUB1)
P62979	Ubiquitin-40S ribosomal protein S27a (Ubiquitin carboxyl extension protein 80) [Cleaved into: Ubiquitin; 40S ribosomal protein S27a (Small ribosomal subunit protein eS31)]

Q14157	Ubiquitin-associated protein 2-like (Protein NICE-4)
P68036	Ubiquitin-conjugating enzyme E2 L3 (EC 2.3.2.23) (E2 ubiquitin-conjugating enzyme L3) (L-UBC) (UbcH7) (Ubiquitin carrier protein L3) (Ubiquitin-conjugating enzyme E2-F1) (Ubiquitin-protein ligase L3)
P61088	Ubiquitin-conjugating enzyme E2 N (EC 2.3.2.23) (Bendless-like ubiquitin-conjugating enzyme) (E2 ubiquitin-conjugating enzyme N) (Ubc13) (UbcH13) (Ubiquitin carrier protein N) (Ubiquitin-protein ligase N)
P22314	Ubiquitin-like modifier-activating enzyme 1 (EC 6.2.1.45) (Protein A1S9) (Ubiquitin-activating enzyme E1)
O00159	Unconventional myosin-Ic (Myosin I beta) (MMI-beta) (MMIb)
Q9UM54	Unconventional myosin-VI (Unconventional myosin-6)
Q9NWV4	UPF0587 protein C1orf123
Q16851	UTP--glucose-1-phosphate uridylyltransferase (EC 2.7.7.9) (UDP-glucose pyrophosphorylase) (UDPGP) (UGPase)
P46939	Utrophin (Dystrophin-related protein 1) (DRP-1)
P54725	UV excision repair protein RAD23 homolog A (HR23A) (hHR23A)
P54727	UV excision repair protein RAD23 homolog B (HR23B) (hHR23B) (XP-C repair-complementing complex 58 kDa protein) (p58)
P38606	V-type proton ATPase catalytic subunit A (V-ATPase subunit A) (EC 3.6.3.14) (V-ATPase 69 kDa subunit) (Vacuolar ATPase isoform VA68) (Vacuolar proton pump subunit alpha)
P21281	V-type proton ATPase subunit B, brain isoform (V-ATPase subunit B 2) (Endomembrane proton pump 58 kDa subunit) (HO57) (Vacuolar proton pump subunit B 2)
O75436	Vacuolar protein sorting-associated protein 26A (Vesicle protein sorting 26A) (hVPS26)
Q9UBQ0	Vacuolar protein sorting-associated protein 29 (hVPS29) (PEP11 homolog) (Vesicle protein sorting 29)
Q96QK1	Vacuolar protein sorting-associated protein 35 (hVPS35) (Maternal-embryonic 3) (Vesicle protein sorting 35)
P13611	Versican core protein (Chondroitin sulfate proteoglycan core protein 2) (Chondroitin sulfate proteoglycan 2) (Glial hyaluronate-binding protein) (GHAP) (Large fibroblast proteoglycan) (PG-M)
P49748	Very long-chain specific acyl-CoA dehydrogenase, mitochondrial (VLCAD) (EC 1.3.8.9)
O95292	Vesicle-associated membrane protein-associated protein B/C (VAMP-B/VAMP-C) (VAMP-associated protein B/C) (VAP-B/VAP-C)
P46459	Vesicle-fusing ATPase (EC 3.6.4.6) (N-ethylmaleimide-sensitive fusion protein) (NEM-sensitive fusion protein) (Vesicular-fusion protein NSF)
O75396	Vesicle-trafficking protein SEC22b (ER-Golgi SNARE of 24 kDa) (ERS-24) (ERS24) (SEC22 vesicle-trafficking protein homolog B) (SEC22 vesicle-trafficking protein-like 1)
Q00341	Vigilin (High density lipoprotein-binding protein) (HDL-binding protein)
P08670	Vimentin

P18206	Vinculin (Metavinculin) (MV)
P02774	Vitamin D-binding protein (DBP) (VDB) (Gc protein-derived macrophage activating factor) (Gc-MAF) (GcMAF) (Gc-globulin) (Group-specific component) (Gc) (Vitamin D-binding protein-macrophage activating factor) (DBP-maf)
P04004	Vitronectin (VN) (S-protein) (Serum-spreading factor) (V75) [Cleaved into: Vitronectin V65 subunit; Vitronectin V10 subunit; Somatomedin-B]
P45880	Voltage-dependent anion-selective channel protein 2 (VDAC-2) (hVDAC2) (Outer mitochondrial membrane protein porin 2)
O75083	WD repeat-containing protein 1 (Actin-interacting protein 1) (AIP1) (NORI-1)
O76024	Wolframin
P13010	X-ray repair cross-complementing protein 5 (EC 3.6.4.-) (86 kDa subunit of Ku antigen) (ATP-dependent DNA helicase 2 subunit 2) (ATP-dependent DNA helicase II 80 kDa subunit) (CTC box-binding factor 85 kDa subunit) (CTC85) (CTCBF) (DNA repair protein XRCC5) (Ku80) (Ku86) (Lupus Ku autoantigen protein p86) (Nuclear factor IV) (Thyroid-lupus autoantigen) (TLAA) (X-ray repair complementing defective repair in Chinese hamster cells 5 (double-strand-break rejoining))
P12956	X-ray repair cross-complementing protein 6 (EC 3.6.4.-) (EC 4.2.99.-) (5'-deoxyribose-5-phosphate lyase Ku70) (5'-dRP lyase Ku70) (70 kDa subunit of Ku antigen) (ATP-dependent DNA helicase 2 subunit 1) (ATP-dependent DNA helicase II 70 kDa subunit) (CTC box-binding factor 75 kDa subunit) (CTC75) (CTCBF) (DNA repair protein XRCC6) (Lupus Ku autoantigen protein p70) (Ku70) (Thyroid-lupus autoantigen) (TLAA) (X-ray repair complementing defective repair in Chinese hamster cells 6)
P12955	Xaa-Pro dipeptidase (X-Pro dipeptidase) (EC 3.4.13.9) (Imidodipeptidase) (Peptidase D) (Proline dipeptidase) (Prolidase)
P25311	Zinc-alpha-2-glycoprotein (Zn-alpha-2-GP) (Zn-alpha-2-glycoprotein)
Q15942	Zyxin (Zyxin-2)

Uniprot ID	Protein names
P04217	Alpha-1B-glycoprotein (Alpha-1-B glycoprotein)
P01023	Alpha-2-macroglobulin (Alpha-2-M) (C3 and PZP-like alpha-2-macroglobulin domain-containing protein 5)
P49588	Alanine--tRNA ligase, cytoplasmic (EC 6.1.1.7) (Alanyl-tRNA synthetase) (AlaRS) (Renal carcinoma antigen NY-REN-42)
Q96IU4	Protein ABHD14B (EC 3.-.-.-) (Alpha/beta hydrolase domain-containing protein 14B) (Abhydrolase domain-containing protein 14B) (CCG1-interacting factor B)
P42765	3-ketoacyl-CoA thiolase, mitochondrial (EC 2.3.1.16) (Acetyl-CoA acetyltransferase) (EC 2.3.1.9) (Acetyl-CoA acyltransferase) (Acyl-CoA hydrolase, mitochondrial) (EC 3.1.2.-) (EC 3.1.2.1) (EC 3.1.2.2) (Beta-ketothiolase) (Mitochondrial 3-oxoacyl-CoA thiolase) (T1)
P49748	Very long-chain specific acyl-CoA dehydrogenase, mitochondrial (VLCAD) (EC 1.3.8.9)
P24752	Acetyl-CoA acetyltransferase, mitochondrial (EC 2.3.1.9) (Acetoacetyl-CoA thiolase) (T2)
Q99798	Aconitate hydratase, mitochondrial (Aconitase) (EC 4.2.1.3) (Citrate hydro-lyase)
P24666	Low molecular weight phosphotyrosine protein phosphatase (LMW-PTP) (LMW-PTPase) (EC 3.1.3.48) (Adipocyte acid phosphatase) (Low molecular weight cytosolic acid phosphatase) (EC 3.1.3.2) (Red cell acid phosphatase 1)
P12814	Alpha-actinin-1 (Alpha-actinin cytoskeletal isoform) (F-actin cross-linking protein) (Non-muscle alpha-actinin-1)
O43707	Alpha-actinin-4 (Non-muscle alpha-actinin 4)
P61160	Actin-related protein 2 (Actin-like protein 2)
P61158	Actin-related protein 3 (Actin-like protein 3)
P35611	Alpha-adducin (Erythrocyte adducin subunit alpha)
Q8IUX7	Adipocyte enhancer-binding protein 1 (AE-binding protein 1) (Aortic carboxypeptidase-like protein)
P23526	Adenosylhomocysteinase (AdoHcyase) (EC 3.3.1.1) (S-adenosyl-L-homocysteine hydrolase)
O95831	Apoptosis-inducing factor 1, mitochondrial (EC 1.1.1.-) (Programmed cell death protein 8)
P54819	Adenylate kinase 2, mitochondrial (AK 2) (EC 2.7.4.3) (ATP-AMP transphosphorylase 2) (ATP:AMP phosphotransferase) (Adenylate monophosphate kinase) [Cleaved into: Adenylate kinase 2, mitochondrial, N-terminally processed]
O43488	Aflatoxin B1 aldehyde reductase member 2 (EC 1.1.1.n11) (AFB1 aldehyde reductase 1) (AFB1-AR 1) (Aldoketoreductase 7) (Succinic semialdehyde reductase) (SSA reductase)
P00352	Retinal dehydrogenase 1 (RALDH 1) (RalDH1) (EC 1.2.1.-) (EC 1.2.1.36) (ALDH-E1) (ALHDII) (Aldehyde dehydrogenase family 1 member A1) (Aldehyde dehydrogenase, cytosolic)
P05091	Aldehyde dehydrogenase, mitochondrial (EC 1.2.1.3) (ALDH class 2) (ALDH-E2) (ALDHI)

P49189	4-trimethylaminobutyraldehyde dehydrogenase (TMABA-DH) (TMABALDH) (EC 1.2.1.47) (Aldehyde dehydrogenase E3 isozyme) (Aldehyde dehydrogenase family 9 member A1) (EC 1.2.1.3) (Gamma-aminobutyraldehyde dehydrogenase) (EC 1.2.1.19) (R-aminobutyraldehyde dehydrogenase) [Cleaved into: 4-trimethylaminobutyraldehyde dehydrogenase, N-terminally processed]
P04075	Fructose-bisphosphate aldolase A (EC 4.1.2.13) (Lung cancer antigen NY-LU-1) (Muscle-type aldolase)
P09972	Fructose-bisphosphate aldolase C (EC 4.1.2.13) (Brain-type aldolase)
P04083	Annexin A1 (Annexin I) (Annexin-1) (Calpactin II) (Calpactin-2) (Chromobindin-9) (Lipocortin I) (Phospholipase A2 inhibitory protein) (p35)
P07355	Annexin A2 (Annexin II) (Annexin-2) (Calpactin I heavy chain) (Calpactin-1 heavy chain) (Chromobindin-8) (Lipocortin II) (Placental anticoagulant protein IV) (PAP-IV) (Protein I) (p36)
P12429	Annexin A3 (35-alpha calcimedin) (Annexin III) (Annexin-3) (Inositol 1,2-cyclic phosphate 2-phosphohydrolase) (Lipocortin III) (Placental anticoagulant protein III) (PAP-III)
P09525	Annexin A4 (35-beta calcimedin) (Annexin IV) (Annexin-4) (Carbohydrate-binding protein p33/p41) (Chromobindin-4) (Endonexin I) (Lipocortin IV) (P32.5) (PP4-X) (Placental anticoagulant protein II) (PAP-II) (Protein II)
P08758	Annexin A5 (Anchorin CII) (Annexin V) (Annexin-5) (Calphobindin I) (CBP-I) (Endonexin II) (Lipocortin V) (Placental anticoagulant protein 4) (PP4) (Placental anticoagulant protein I) (PAP-I) (Thromboplastin inhibitor) (Vascular anticoagulant-alpha) (VAC-alpha)
P08133	Annexin A6 (67 kDa calelectrin) (Annexin VI) (Annexin-6) (Calphobindin-II) (CPB-II) (Chromobindin-20) (Lipocortin VI) (Protein III) (p68) (p70)
P27695	DNA-(apurinic or apyrimidinic site) lyase (EC 3.1.-.-) (EC 4.2.99.18) (APEX nuclease) (APEN) (Apurinic-apyrimidinic endonuclease 1) (AP endonuclease 1) (APE-1) (REF-1) (Redox factor-1) [Cleaved into: DNA-(apurinic or apyrimidinic site) lyase, mitochondrial]
P02647	Apolipoprotein A-I (Apo-AI) (ApoA-I) (Apolipoprotein A1) [Cleaved into: Proapolipoprotein A-I (ProapoA-I); Truncated apolipoprotein A-I (Apolipoprotein A-I(1-242))]
P48444	Coatomer subunit delta (Archain) (Delta-coat protein) (Delta-COP)
Q07960	Rho GTPase-activating protein 1 (CDC42 GTPase-activating protein) (GTPase-activating protein rhoGAP) (Rho-related small GTPase protein activator) (Rho-type GTPase-activating protein 1) (p50-RhoGAP)
O15145	Actin-related protein 2/3 complex subunit 3 (Arp2/3 complex 21 kDa subunit) (p21-ARC)
P59998	Actin-related protein 2/3 complex subunit 4 (Arp2/3 complex 20 kDa subunit) (p20-ARC)
O15511	Actin-related protein 2/3 complex subunit 5 (Arp2/3 complex 16 kDa subunit) (p16-ARC)
P00966	Argininosuccinate synthase (EC 6.3.4.5) (Citrulline--aspartate ligase)

P31939	Bifunctional purine biosynthesis protein PURH [Cleaved into: Bifunctional purine biosynthesis protein PURH, N-terminally processed] [Includes: Phosphoribosylaminoimidazolecarboxamide formyltransferase (EC 2.1.2.3) (5-aminoimidazole-4-carboxamide ribonucleotide formyltransferase) (AICAR transformylase); IMP cyclohydrolase (EC 3.5.4.10) (ATIC) (IMP synthase) (Inosinicase)]
P38606	V-type proton ATPase catalytic subunit A (V-ATPase subunit A) (EC 7.1.2.2) (V-ATPase 69 kDa subunit) (Vacuolar ATPase isoform VA68) (Vacuolar proton pump subunit alpha)
P21281	V-type proton ATPase subunit B, brain isoform (V-ATPase subunit B 2) (Endomembrane proton pump 58 kDa subunit) (HO57) (Vacuolar proton pump subunit B 2)
P61769	Beta-2-microglobulin [Cleaved into: Beta-2-microglobulin form pI 5.3]
P21810	Biglycan (Bone/cartilage proteoglycan I) (PG-S1)
P53004	Biliverdin reductase A (BVR A) (EC 1.3.1.24) (Biliverdin-IX alpha-reductase)
O43684	Mitotic checkpoint protein BUB3
P01024	Complement C3 (C3 and PZP-like alpha-2-macroglobulin domain-containing protein 1) [Cleaved into: Complement C3 beta chain; C3-beta-c (C3bc); Complement C3 alpha chain; C3a anaphylatoxin; Acylation stimulating protein (ASP) (C3adesArg); Complement C3b alpha' chain; Complement C3c alpha' chain fragment 1; Complement C3dg fragment; Complement C3g fragment; Complement C3d fragment; Complement C3f fragment; Complement C3c alpha' chain fragment 2]
Q05682	Caldesmon (CDM)
P27797	Calreticulin (CRP55) (Calregulin) (Endoplasmic reticulum resident protein 60) (ERp60) (HACBP) (grp60)
Q86VP6	Cullin-associated NEDD8-dissociated protein 1 (Cullin-associated and neddylation-dissociated protein 1) (TBP-interacting protein of 120 kDa A) (TBP-interacting protein 120A) (p120 CAND1)
P27824	Calnexin (IP90) (Major histocompatibility complex class I antigen-binding protein p88) (p90)
Q01518	Adenylyl cyclase-associated protein 1 (CAP 1)
P40121	Macrophage-capping protein (Actin regulatory protein CAP-G)
P07384	Calpain-1 catalytic subunit (EC 3.4.22.52) (Calcium-activated neutral proteinase 1) (CANP 1) (Calpain mu-type) (Calpain-1 large subunit) (Cell proliferation-inducing gene 30 protein) (Micromolar-calpain) (muCANP)
P17655	Calpain-2 catalytic subunit (EC 3.4.22.53) (Calcium-activated neutral proteinase 2) (CANP 2) (Calpain M-type) (Calpain large polypeptide L2) (Calpain-2 large subunit) (Millimolar-calpain) (M-calpain)
P04632	Calpain small subunit 1 (CSS1) (Calcium-activated neutral proteinase small subunit) (CANP small subunit) (Calcium-dependent protease small subunit) (CDPS) (Calcium-dependent protease small subunit 1) (Calpain regulatory subunit)
P52907	F-actin-capping protein subunit alpha-1 (CapZ alpha-1)
P04040	Catalase (EC 1.11.1.6)

P16152	Carbonyl reductase [NADPH] 1 (EC 1.1.1.184) (15-hydroxyprostaglandin dehydrogenase [NADP(+)]) (EC 1.1.1.197) (NADPH-dependent carbonyl reductase 1) (Prostaglandin 9-ketoreductase) (Prostaglandin-E(2) 9-reductase) (EC 1.1.1.189) (Short chain dehydrogenase/reductase family 21C member 1)
P78371	T-complex protein 1 subunit beta (TCP-1-beta) (CCT-beta)
P49368	T-complex protein 1 subunit gamma (TCP-1-gamma) (CCT-gamma) (hTRiC5)
P48643	T-complex protein 1 subunit epsilon (TCP-1-epsilon) (CCT-epsilon)
P40227	T-complex protein 1 subunit zeta (TCP-1-zeta) (Acute morphine dependence-related protein 2) (CCT-zeta-1) (HTR3) (Tcp20)
Q99832	T-complex protein 1 subunit eta (TCP-1-eta) (CCT-eta) (HIV-1 Nef-interacting protein) [Cleaved into: T-complex protein 1 subunit eta, N-terminally processed]
P50990	T-complex protein 1 subunit theta (TCP-1-theta) (CCT-theta) (Chaperonin containing T-complex polypeptide 1 subunit 8) (Renal carcinoma antigen NY-REN-15)
P16070	CD44 antigen (CDw44) (Epican) (Extracellular matrix receptor III) (ECMR-III) (GP90 lymphocyte homing/adhesion receptor) (HUTCH-I) (Heparan sulfate proteoglycan) (Hermes antigen) (Hyaluronate receptor) (Phagocytic glycoprotein 1) (PGP-1) (Phagocytic glycoprotein I) (PGP-I) (CD antigen CD44)
P12830	Cadherin-1 (CAM 120/80) (Epithelial cadherin) (E-cadherin) (Uvomorulin) (CD antigen CD324) [Cleaved into: E-Cad/CTF1; E-Cad/CTF2; E-Cad/CTF3]
P23528	Cofilin-1 (18 kDa phosphoprotein) (p18) (Cofilin, non-muscle isoform)
Q07065	Cytoskeleton-associated protein 4 (63-kDa cytoskeleton-linking membrane protein) (Climp-63) (p63)
O00299	Chloride intracellular channel protein 1 (Chloride channel ABP) (Nuclear chloride ion channel 27) (NCC27) (Regulatory nuclear chloride ion channel protein) (hRNCC)
Q00610	Clathrin heavy chain 1 (Clathrin heavy chain on chromosome 17) (CLH-17)
Q96KP4	Cytosolic non-specific dipeptidase (EC 3.4.13.18) (CNDP dipeptidase 2) (Carnosine dipeptidase II) (Epididymis secretory protein Li 13) (Glutamate carboxypeptidase-like protein 1) (Peptidase A)
P51911	Calponin-1 (Basic calponin) (Calponin H1, smooth muscle)
Q15417	Calponin-3 (Calponin, acidic isoform)
P09543	2',3'-cyclic-nucleotide 3'-phosphodiesterase (CNP) (CNPase) (EC 3.1.4.37)
Q99715	Collagen alpha-1(XII) chain
Q05707	Collagen alpha-1(XIV) chain (Undulin)
P39060	Collagen alpha-1(XVIII) chain [Cleaved into: Endostatin; Non-collagenous domain 1 (NC1)]
P02452	Collagen alpha-1(I) chain (Alpha-1 type I collagen)
P08123	Collagen alpha-2(I) chain (Alpha-2 type I collagen)
P02461	Collagen alpha-1(III) chain
P08572	Collagen alpha-2(IV) chain [Cleaved into: Canstatin]
P12109	Collagen alpha-1(VI) chain

P12110	Collagen alpha-2(VI) chain
P12111	Collagen alpha-3(VI) chain
P53621	Coatomer subunit alpha (Alpha-coat protein) (Alpha-COP) (HEP-COP) (HEPCOP) [Cleaved into: Xenin (Xenopsin-related peptide); Proxenin]
P35606	Coatomer subunit beta' (Beta'-coat protein) (Beta'-COP) (p102)
O14579	Coatomer subunit epsilon (Epsilon-coat protein) (Epsilon-COP)
Q14019	Coactosin-like protein
P00450	Ceruloplasmin (EC 1.16.3.1) (Ferroxidase)
Q08257	Quinone oxidoreductase (EC 1.6.5.5) (NADPH:quinone reductase) (Zeta-crystallin)
O75390	Citrate synthase, mitochondrial (EC 2.3.3.1) (Citrate (Si)-synthase)
P21291	Cysteine and glycine-rich protein 1 (Cysteine-rich protein 1) (CRP) (CRP1) (Epididymis luminal protein 141) (HEL-141)
P04080	Cystatin-B (CPI-B) (Liver thiol proteinase inhibitor) (Stefin-B)
P07339	Cathepsin D (EC 3.4.23.5) [Cleaved into: Cathepsin D light chain; Cathepsin D heavy chain]
P08311	Cathepsin G (CG) (EC 3.4.21.20)
Q9UBR2	Cathepsin Z (EC 3.4.18.1) (Cathepsin P) (Cathepsin X)
Q14247	Src substrate cortactin (Amplaxin) (Oncogene EMS1)
P00167	Cytochrome b5 (Microsomal cytochrome b5 type A) (MCB5)
P00387	NADH-cytochrome b5 reductase 3 (B5R) (Cytochrome b5 reductase) (EC 1.6.2.2) (Diaphorase-1) [Cleaved into: NADH-cytochrome b5 reductase 3 membrane-bound form; NADH-cytochrome b5 reductase 3 soluble form]
P99999	Cytochrome c
Q16643	Drebrin (Developmentally-regulated brain protein)
Q9UJU6	Drebrin-like protein (Cervical SH3P7) (Cervical mucin-associated protein) (Drebrin-F) (HPK1-interacting protein of 55 kDa) (HIP-55) (SH3 domain-containing protein 7)
P81605	Dermcidin (EC 3.4.-.-) (Preproteolysin) [Cleaved into: Survival-promoting peptide; DCD-1]
P07585	Decorin (Bone proteoglycan II) (PG-S2) (PG40)
O94760	N(G),N(G)-dimethylarginine dimethylaminohydrolase 1 (DDAH-1) (Dimethylarginine dimethylaminohydrolase 1) (EC 3.5.3.18) (DDAHI) (Dimethylargininase-1)
O95865	N(G),N(G)-dimethylarginine dimethylaminohydrolase 2 (DDAH-2) (Dimethylarginine dimethylaminohydrolase 2) (EC 3.5.3.18) (DDAHII) (Dimethylargininase-2) (Protein G6a) (S-phase protein)
Q96HY6	DDRGK domain-containing protein 1 (Dashurin) (UFM1-binding and PCI domain-containing protein 1)
P09622	Dihydrolipoyl dehydrogenase, mitochondrial (EC 1.8.1.4) (Dihydrolipoamide dehydrogenase) (Glycine cleavage system L protein)

P25685	DnaJ homolog subfamily B member 1 (DnaJ protein homolog 1) (Heat shock 40 kDa protein 1) (HSP40) (Heat shock protein 40) (Human DnaJ protein 1) (hDj-1)
Q9ULA0	Aspartyl aminopeptidase (EC 3.4.11.21)
Q9NY33	Dipeptidyl peptidase 3 (EC 3.4.14.4) (Dipeptidyl aminopeptidase III) (Dipeptidyl arylamidase III) (Dipeptidyl peptidase III) (DPP III) (Enkephalinase B)
Q16555	Dihydropyrimidinase-related protein 2 (DRP-2) (Collapsin response mediator protein 2) (CRMP-2) (N2A3) (Unc-33-like phosphoprotein 2) (ULIP-2)
Q14195	Dihydropyrimidinase-related protein 3 (DRP-3) (Collapsin response mediator protein 4) (CRMP-4) (Unc-33-like phosphoprotein 1) (ULIP-1)
Q14126	Desmoglein-2 (Cadherin family member 5) (HDGC)
P15924	Desmoplakin (DP) (250/210 kDa paraneoplastic pemphigus antigen)
Q14204	Cytoplasmic dynein 1 heavy chain 1 (Cytoplasmic dynein heavy chain 1) (Dynein heavy chain, cytosolic)
Q13409	Cytoplasmic dynein 1 intermediate chain 2 (Cytoplasmic dynein intermediate chain 2) (Dynein intermediate chain 2, cytosolic) (DH IC-2)
Q13011	Delta(3,5)-Delta(2,4)-dienoyl-CoA isomerase, mitochondrial (EC 5.3.3.-)
P30084	Enoyl-CoA hydratase, mitochondrial (EC 4.2.1.17) (Enoyl-CoA hydratase 1) (Short-chain enoyl-CoA hydratase) (SCEH)
Q15075	Early endosome antigen 1 (Endosome-associated protein p162) (Zinc finger FYVE domain-containing protein 2)
P29692	Elongation factor 1-delta (EF-1-delta) (Antigen NY-CO-4)
P13639	Elongation factor 2 (EF-2)
Q96C19	EF-hand domain-containing protein D2 (Swiprosin-1)
Q9H4M9	EH domain-containing protein 1 (PAST homolog 1) (hPAST1) (Testilin)
Q9NZN3	EH domain-containing protein 3 (PAST homolog 3)
P41091	Eukaryotic translation initiation factor 2 subunit 3 (Eukaryotic translation initiation factor 2 subunit gamma X) (eIF-2-gamma X) (eIF-2gX)
P56537	Eukaryotic translation initiation factor 6 (eIF-6) (B(2)GCN homolog) (B4 integrin interactor) (CAB) (p27(BBP))
P55884	Eukaryotic translation initiation factor 3 subunit B (eIF3b) (Eukaryotic translation initiation factor 3 subunit 9) (Prt1 homolog) (hPrt1) (eIF-3-eta) (eIF3 p110) (eIF3 p116)
P23588	Eukaryotic translation initiation factor 4B (eIF-4B)
Q04637	Eukaryotic translation initiation factor 4 gamma 1 (eIF-4-gamma 1) (eIF-4G 1) (eIF-4G1) (p220)
Q9Y6C2	EMILIN-1 (Elastin microfibril interface-located protein 1) (Elastin microfibril interfacer 1)

P06733	Alpha-enolase (EC 4.2.1.11) (2-phospho-D-glycerate hydro-lyase) (C-myc promoter-binding protein) (Enolase 1) (MBP-1) (MPB-1) (Non-neural enolase) (NNE) (Phosphopyruvate hydratase) (Plasminogen-binding protein)
P09104	Gamma-enolase (EC 4.2.1.11) (2-phospho-D-glycerate hydro-lyase) (Enolase 2) (Neural enolase) (Neuron-specific enolase) (NSE)
O43491	Band 4.1-like protein 2 (Generally expressed protein 4.1) (4.1G)
P07099	Epoxide hydrolase 1 (EC 3.3.2.9) (Epoxide hydratase) (Microsomal epoxide hydrolase) (mEH)
P10768	S-formylglutathione hydrolase (FGH) (EC 3.1.2.12) (Esterase D) (Methylumbelliferyl-acetate deacetylase) (EC 3.1.1.56)
P13804	Electron transfer flavoprotein subunit alpha, mitochondrial (Alpha-ETF)
P38117	Electron transfer flavoprotein subunit beta (Beta-ETF)
Q01844	RNA-binding protein EWS (EWS oncogene) (Ewing sarcoma breakpoint region 1 protein)
P15311	Ezrin (Cytovillin) (Villin-2) (p81)
P23142	Fibulin-1 (FIBL-1)
P35555	Fibrillin-1 [Cleaved into: Asprosin]
P09467	Fructose-1,6-bisphosphatase 1 (FBPase 1) (EC 3.1.3.11) (D-fructose-1,6-bisphosphate 1-phosphohydrolase 1) (Liver FBPase)
Q96AC1	Fermitin family homolog 2 (Kindlin-2) (Mitogen-inducible gene 2 protein) (MIG-2) (Pleckstrin homology domain-containing family C member 1) (PH domain-containing family C member 1)
P02671	Fibrinogen alpha chain [Cleaved into: Fibrinopeptide A; Fibrinogen alpha chain]
P02675	Fibrinogen beta chain [Cleaved into: Fibrinopeptide B; Fibrinogen beta chain]
P02679	Fibrinogen gamma chain
Q00688	Peptidyl-prolyl cis-trans isomerase FKBP3 (PPIase FKBP3) (EC 5.2.1.8) (25 kDa FK506-binding protein) (25 kDa FKBP) (FKBP-25) (FK506-binding protein 3) (FKBP-3) (Immunophilin FKBP25) (Rapamycin-selective 25 kDa immunophilin) (Rotamase)
Q02790	Peptidyl-prolyl cis-trans isomerase FKBP4 (PPIase FKBP4) (EC 5.2.1.8) (51 kDa FK506-binding protein) (FKBP51) (52 kDa FK506-binding protein) (52 kDa FKBP) (FKBP-52) (59 kDa immunophilin) (p59) (FK506-binding protein 4) (FKBP-4) (FKBP59) (HSP-binding immunophilin) (HBI) (Immunophilin FKBP52) (Rotamase) [Cleaved into: Peptidyl-prolyl cis-trans isomerase FKBP4, N-terminally processed]
P21333	Filamin-A (FLN-A) (Actin-binding protein 280) (ABP-280) (Alpha-filamin) (Endothelial actin-binding protein) (Filamin-1) (Non-muscle filamin)
O75369	Filamin-B (FLN-B) (ABP-278) (ABP-280 homolog) (Actin-binding-like protein) (Beta-filamin) (Filamin homolog 1) (Fh1) (Filamin-3) (Thyroid autoantigen) (Truncated actin-binding protein) (Truncated ABP)
O75955	Flotillin-1
P02751	Fibronectin (FN) (Cold-insoluble globulin) (CIG) [Cleaved into: Anastellin; Ugl-Y1; Ugl-Y2; Ugl-Y3]

Q16658	Fascin (55 kDa actin-bundling protein) (Singed-like protein) (p55)
P02794	Ferritin heavy chain (Ferritin H subunit) (EC 1.16.3.1) (Cell proliferation-inducing gene 15 protein) [Cleaved into: Ferritin heavy chain, N-terminally processed]
P02792	Ferritin light chain (Ferritin L subunit)
P35637	RNA-binding protein FUS (75 kDa DNA-pairing protein) (Oncogene FUS) (Oncogene TLS) (POMp75) (Translocated in liposarcoma protein)
P11413	Glucose-6-phosphate 1-dehydrogenase (G6PD) (EC 1.1.1.49)
P31150	Rab GDP dissociation inhibitor alpha (Rab GDI alpha) (Guanosine diphosphate dissociation inhibitor 1) (GDI-1) (Oligophrenin-2) (Protein XAP-4)
Q04760	Lactoylglutathione lyase (EC 4.4.1.5) (Aldoketomutase) (Glyoxalase I) (Glx I) (Ketone-aldehyde mutase) (Methylglyoxalase) (S-D-lactoylglutathione methylglyoxal lyase)
P00367	Glutamate dehydrogenase 1, mitochondrial (GDH 1) (EC 1.4.1.3)
P62879	Guanine nucleotide-binding protein G(I)/G(S)/G(T) subunit beta-2 (G protein subunit beta-2) (Transducin beta chain 2)
Q14789	Golgin subfamily B member 1 (372 kDa Golgi complex-associated protein) (GCP372) (Giantin) (Macrogolgin)
P17174	Aspartate aminotransferase, cytoplasmic (cAspAT) (EC 2.6.1.1) (EC 2.6.1.3) (Cysteine aminotransferase, cytoplasmic) (Cysteine transaminase, cytoplasmic) (cCAT) (Glutamate oxaloacetate transaminase 1) (Transaminase A)
P00505	Aspartate aminotransferase, mitochondrial (mAspAT) (EC 2.6.1.1) (EC 2.6.1.7) (Fatty acid-binding protein) (FABP-1) (Glutamate oxaloacetate transaminase 2) (Kynurenine aminotransferase 4) (Kynurenine aminotransferase IV) (Kynurenine--oxoglutarate transaminase 4) (Kynurenine--oxoglutarate transaminase IV) (Plasma membrane-associated fatty acid-binding protein) (FABPpm) (Transaminase A)
P06744	Glucose-6-phosphate isomerase (GPI) (EC 5.3.1.9) (Autocrine motility factor) (AMF) (Neuroleukin) (NLK) (Phosphoglucose isomerase) (PGI) (Phosphohexose isomerase) (PHI) (Sperm antigen 36) (SA-36)
P62993	Growth factor receptor-bound protein 2 (Adapter protein GRB2) (Protein Ash) (SH2/SH3 adapter GRB2)
Q9UBQ7	Glyoxylate reductase/hydroxypyruvate reductase (EC 1.1.1.79) (EC 1.1.1.81)
P06396	Gelsolin (AGEL) (Actin-depolymerizing factor) (ADF) (Brevin)
P48637	Glutathione synthetase (GSH synthetase) (GSH-S) (EC 6.3.2.3) (Glutathione synthase)
P78417	Glutathione S-transferase omega-1 (GSTO-1) (EC 2.5.1.18) (Glutathione S-transferase omega 1-1) (GSTO 1-1) (Glutathione-dependent dehydroascorbate reductase) (EC 1.8.5.1) (Monomethylarsonic acid reductase) (MMA(V) reductase) (EC 1.20.4.2) (S-(Phenacyl)glutathione reductase) (SPG-R)
P09211	Glutathione S-transferase P (EC 2.5.1.18) (GST class-pi) (GSTP1-1)

O75367	Core histone macro-H2A.1 (Histone macroH2A1) (mH2A1) (Histone H2A.y) (H2A/y) (Medulloblastoma antigen MU-MB-50.205)
P40939	Trifunctional enzyme subunit alpha, mitochondrial (78 kDa gastrin-binding protein) (Monolysocardiolipin acyltransferase) (EC 2.3.1.-) (TP-alpha) [Includes: Long-chain enoyl-CoA hydratase (EC 4.2.1.17); Long chain 3-hydroxyacyl-CoA dehydrogenase (EC 1.1.1.211)]
P55084	Trifunctional enzyme subunit beta, mitochondrial (TP-beta) [Includes: 3-ketoacyl-CoA thiolase (EC 2.3.1.155) (EC 2.3.1.16) (Acetyl-CoA acyltransferase) (Beta-ketothiolase)]
P51858	Hepatoma-derived growth factor (HDGF) (High mobility group protein 1-like 2) (HMG-1L2)
Q00341	Vigilin (High density lipoprotein-binding protein) (HDL-binding protein)
Q6NVY1	3-hydroxyisobutyryl-CoA hydrolase, mitochondrial (EC 3.1.2.4) (3-hydroxyisobutyryl-coenzyme A hydrolase) (HIB-CoA hydrolase) (HIBYL-CoA-H)
P19367	Hexokinase-1 (EC 2.7.1.1) (Brain form hexokinase) (Hexokinase type I) (HK I) (Hexokinase-A)
P09429	High mobility group protein B1 (High mobility group protein 1) (HMG-1)
P09651	Heterogeneous nuclear ribonucleoprotein A1 (hnRNP A1) (Helix-destabilizing protein) (Single-strand RNA-binding protein) (hnRNP core protein A1) [Cleaved into: Heterogeneous nuclear ribonucleoprotein A1, N-terminally processed]
P22626	Heterogeneous nuclear ribonucleoproteins A2/B1 (hnRNP A2/B1)
P51991	Heterogeneous nuclear ribonucleoprotein A3 (hnRNP A3)
Q99729	Heterogeneous nuclear ribonucleoprotein A/B (hnRNP A/B) (APOBEC1-binding protein 1) (ABBP-1)
P07910	Heterogeneous nuclear ribonucleoproteins C1/C2 (hnRNP C1/C2)
Q14103	Heterogeneous nuclear ribonucleoprotein D0 (hnRNP D0) (AU-rich element RNA-binding protein 1)
P52597	Heterogeneous nuclear ribonucleoprotein F (hnRNP F) (Nucleolin-like protein mcs94-1) [Cleaved into: Heterogeneous nuclear ribonucleoprotein F, N-terminally processed]
P31943	Heterogeneous nuclear ribonucleoprotein H (hnRNP H) [Cleaved into: Heterogeneous nuclear ribonucleoprotein H, N-terminally processed]
P31942	Heterogeneous nuclear ribonucleoprotein H3 (hnRNP H3) (Heterogeneous nuclear ribonucleoprotein 2H9) (hnRNP 2H9)
P61978	Heterogeneous nuclear ribonucleoprotein K (hnRNP K) (Transformation up-regulated nuclear protein) (TUNP)
P14866	Heterogeneous nuclear ribonucleoprotein L (hnRNP L)
P52272	Heterogeneous nuclear ribonucleoprotein M (hnRNP M)
O43390	Heterogeneous nuclear ribonucleoprotein R (hnRNP R)
Q00839	Heterogeneous nuclear ribonucleoprotein U (hnRNP U) (GRIP120) (Nuclear p120 ribonucleoprotein) (Scaffold-attachment factor A) (SAF-A) (p120) (pp120)
Q1KMD3	Heterogeneous nuclear ribonucleoprotein U-like protein 2 (Scaffold-attachment factor A2) (SAF-A2)

Q5SSJ5	Heterochromatin protein 1-binding protein 3 (Protein HP1-BP74)
P07900	Heat shock protein HSP 90-alpha (Heat shock 86 kDa) (HSP 86) (HSP86) (Lipopolysaccharide-associated protein 2) (LAP-2) (LPS-associated protein 2) (Renal carcinoma antigen NY-REN-38)
P08238	Heat shock protein HSP 90-beta (HSP 90) (Heat shock 84 kDa) (HSP 84) (HSP84)
P14625	Endoplasmin (94 kDa glucose-regulated protein) (GRP-94) (Heat shock protein 90 kDa beta member 1) (Tumor rejection antigen 1) (gp96 homolog)
P34932	Heat shock 70 kDa protein 4 (HSP70RY) (Heat shock 70-related protein APG-2)
P11021	Endoplasmic reticulum chaperone BiP (EC 3.6.4.10) (78 kDa glucose-regulated protein) (GRP-78) (Binding-immunoglobulin protein) (BiP) (Heat shock protein 70 family protein 5) (HSP70 family protein 5) (Heat shock protein family A member 5) (Immunoglobulin heavy chain-binding protein)
P11142	Heat shock cognate 71 kDa protein (Heat shock 70 kDa protein 8) (Lipopolysaccharide-associated protein 1) (LAP-1) (LPS-associated protein 1)
P38646	Stress-70 protein, mitochondrial (75 kDa glucose-regulated protein) (GRP-75) (Heat shock 70 kDa protein 9) (Mortalin) (MOT) (Peptide-binding protein 74) (PBP74)
P04792	Heat shock protein beta-1 (HspB1) (28 kDa heat shock protein) (Estrogen-regulated 24 kDa protein) (Heat shock 27 kDa protein) (HSP 27) (Stress-responsive protein 27) (SRP27)
P10809	60 kDa heat shock protein, mitochondrial (EC 5.6.1.7) (60 kDa chaperonin) (Chaperonin 60) (CPN60) (Heat shock protein 60) (HSP-60) (Hsp60) (HuCHA60) (Mitochondrial matrix protein P1) (P60 lymphocyte protein)
P61604	10 kDa heat shock protein, mitochondrial (Hsp10) (10 kDa chaperonin) (Chaperonin 10) (CPN10) (Early-pregnancy factor) (EPF)
Q7Z6Z7	E3 ubiquitin-protein ligase HUWE1 (EC 2.3.2.26) (ARF-binding protein 1) (ARF-BP1) (HECT, UBA and WWE domain-containing protein 1) (HECT-type E3 ubiquitin transferase HUWE1) (Homologous to E6AP carboxyl terminus homologous protein 9) (HectH9) (Large structure of UREB1) (LASU1) (Mcl-1 ubiquitin ligase E3) (Mule) (Upstream regulatory element-binding protein 1) (URE-B1) (URE-binding protein 1)
Q9Y4L1	Hypoxia up-regulated protein 1 (150 kDa oxygen-regulated protein) (ORP-150) (170 kDa glucose-regulated protein) (GRP-170)
O75874	Isocitrate dehydrogenase [NADP] cytoplasmic (IDH) (EC 1.1.1.42) (Cytosolic NADP-isocitrate dehydrogenase) (IDP) (NADP(+)-specific ICDH) (Oxalosuccinate decarboxylase)
Q12905	Interleukin enhancer-binding factor 2 (Nuclear factor of activated T-cells 45 kDa)
Q12906	Interleukin enhancer-binding factor 3 (Double-stranded RNA-binding protein 76) (DRBP76) (M-phase phosphoprotein 4) (MPP4) (Nuclear factor associated with dsRNA) (NFAR) (Nuclear factor of activated T-cells 90 kDa) (NF-AT-90) (Translational control protein 80) (TCP80)
P46940	Ras GTPase-activating-like protein IQGAP1 (p195)
Q13576	Ras GTPase-activating-like protein IQGAP2

P05556	Integrin beta-1 (Fibronectin receptor subunit beta) (Glycoprotein IIa) (GPIIA) (VLA-4 subunit beta) (CD antigen CD29)
P19823	Inter-alpha-trypsin inhibitor heavy chain H2 (ITI heavy chain H2) (ITI-HC2) (Inter-alpha-inhibitor heavy chain 2) (Inter-alpha-trypsin inhibitor complex component II) (Serum-derived hyaluronan-associated protein) (SHAP)
P14923	Junction plakoglobin (Catenin gamma) (Desmoplakin III) (Desmoplakin-3)
Q96CX2	BTB/POZ domain-containing protein KCTD12 (Pfetin) (Predominantly fetal expressed T1 domain)
Q92945	Far upstream element-binding protein 2 (FUSE-binding protein 2) (KH type-splicing regulatory protein) (KSRP) (p75)
P33176	Kinesin-1 heavy chain (Conventional kinesin heavy chain) (Ubiquitous kinesin heavy chain) (UKHC)
Q14974	Importin subunit beta-1 (Importin-90) (Karyopherin subunit beta-1) (Nuclear factor p97) (Pore targeting complex 97 kDa subunit) (PTAC97)
P04264	Keratin, type II cytoskeletal 1 (67 kDa cytokeratin) (Cytokeratin-1) (CK-1) (Hair alpha protein) (Keratin-1) (K1) (Type-II keratin Kb1)
P13645	Keratin, type I cytoskeletal 10 (Cytokeratin-10) (CK-10) (Keratin-10) (K10)
P02533	Keratin, type I cytoskeletal 14 (Cytokeratin-14) (CK-14) (Keratin-14) (K14)
Q04695	Keratin, type I cytoskeletal 17 (39.1) (Cytokeratin-17) (CK-17) (Keratin-17) (K17)
P05783	Keratin, type I cytoskeletal 18 (Cell proliferation-inducing gene 46 protein) (Cytokeratin-18) (CK-18) (Keratin-18) (K18)
P08727	Keratin, type I cytoskeletal 19 (Cytokeratin-19) (CK-19) (Keratin-19) (K19)
P05787	Keratin, type II cytoskeletal 8 (Cytokeratin-8) (CK-8) (Keratin-8) (K8) (Type-II keratin Kb8)
P35527	Keratin, type I cytoskeletal 9 (Cytokeratin-9) (CK-9) (Keratin-9) (K9)
Q86UP2	Kinectin (CG-1 antigen) (Kinesin receptor)
P07942	Laminin subunit beta-1 (Laminin B1 chain) (Laminin-1 subunit beta) (Laminin-10 subunit beta) (Laminin-12 subunit beta) (Laminin-2 subunit beta) (Laminin-6 subunit beta) (Laminin-8 subunit beta)
P55268	Laminin subunit beta-2 (Laminin B1s chain) (Laminin-11 subunit beta) (Laminin-14 subunit beta) (Laminin-15 subunit beta) (Laminin-3 subunit beta) (Laminin-4 subunit beta) (Laminin-7 subunit beta) (Laminin-9 subunit beta) (S-laminin subunit beta) (S-LAM beta)
P11047	Laminin subunit gamma-1 (Laminin B2 chain) (Laminin-1 subunit gamma) (Laminin-10 subunit gamma) (Laminin-11 subunit gamma) (Laminin-2 subunit gamma) (Laminin-3 subunit gamma) (Laminin-4 subunit gamma) (Laminin-6 subunit gamma) (Laminin-7 subunit gamma) (Laminin-8 subunit gamma) (Laminin-9 subunit gamma) (S-laminin subunit gamma) (S-LAM gamma)
P28838	Cytosol aminopeptidase (EC 3.4.11.1) (Leucine aminopeptidase 3) (LAP-3) (Leucyl aminopeptidase) (Peptidase S) (Proline aminopeptidase) (EC 3.4.11.5) (Prolyl aminopeptidase)
Q14847	LIM and SH3 domain protein 1 (LASP-1) (Metastatic lymph node gene 50 protein) (MLN 50)

P13796	Plastin-2 (L-plastin) (LC64P) (Lymphocyte cytosolic protein 1) (LCP-1)
P00338	L-lactate dehydrogenase A chain (LDH-A) (EC 1.1.1.27) (Cell proliferation-inducing gene 19 protein) (LDH muscle subunit) (LDH-M) (Renal carcinoma antigen NY-REN-59)
P07195	L-lactate dehydrogenase B chain (LDH-B) (EC 1.1.1.27) (LDH heart subunit) (LDH-H) (Renal carcinoma antigen NY-REN-46)
P09382	Galectin-1 (Gal-1) (14 kDa laminin-binding protein) (HLBP14) (14 kDa lectin) (Beta-galactoside-binding lectin L-14-I) (Galaptin) (HBL) (HPL) (Lactose-binding lectin 1) (Lectin galactoside-binding soluble 1) (Putative MAPK-activating protein PM12) (S-Lac lectin 1)
P17931	Galectin-3 (Gal-3) (35 kDa lectin) (Carbohydrate-binding protein 35) (CBP 35) (Galactose-specific lectin 3) (Galactoside-binding protein) (GALBP) (IgE-binding protein) (L-31) (Laminin-binding protein) (Lectin L-29) (Mac-2 antigen)
Q08380	Galectin-3-binding protein (Basement membrane autoantigen p105) (Lectin galactoside-binding soluble 3-binding protein) (Mac-2-binding protein) (MAC2BP) (Mac-2 BP) (Tumor-associated antigen 90K)
P02545	Prelamin-A/C [Cleaved into: Lamin-A/C (70 kDa lamin) (Renal carcinoma antigen NY-REN-32)]
P20700	Lamin-B1
Q03252	Lamin-B2
Q93052	Lipoma-preferred partner (LIM domain-containing preferred translocation partner in lipoma)
P51884	Lumican (Keratan sulfate proteoglycan lumican) (KSPG lumican)
Q02750	Dual specificity mitogen-activated protein kinase kinase 1 (MAP kinase kinase 1) (MAPKK 1) (MKK1) (EC 2.7.12.2) (ERK activator kinase 1) (MAPK/ERK kinase 1) (MEK 1)
P43243	Matrin-3
P40925	Malate dehydrogenase, cytoplasmic (EC 1.1.1.37) (Cytosolic malate dehydrogenase) (Diiodophenylpyruvate reductase) (EC 1.1.1.96)
P40926	Malate dehydrogenase, mitochondrial (EC 1.1.1.37)
Q14165	Malectin
P25325	3-mercaptopyruvate sulfurtransferase (MST) (EC 2.8.1.2)
P26038	Moesin (Membrane-organizing extension spike protein)
P11586	C-1-tetrahydrofolate synthase, cytoplasmic (C1-THF synthase) [Cleaved into: C-1-tetrahydrofolate synthase, cytoplasmic, N-terminally processed] [Includes: Methylenetetrahydrofolate dehydrogenase (EC 1.5.1.5); Methenyltetrahydrofolate cyclohydrolase (EC 3.5.4.9); Formyltetrahydrofolate synthetase (EC 6.3.4.3)]
Q14764	Major vault protein (MVP) (Lung resistance-related protein)

P35580	Myosin-10 (Cellular myosin heavy chain, type B) (Myosin heavy chain 10) (Myosin heavy chain, non-muscle IIb) (Non-muscle myosin heavy chain B) (NMMHC-B) (Non-muscle myosin heavy chain IIb) (NMMHC II-b) (NMMHC-IIB)
P35579	Myosin-9 (Cellular myosin heavy chain, type A) (Myosin heavy chain 9) (Myosin heavy chain, non-muscle IIa) (Non-muscle myosin heavy chain A) (NMMHC-A) (Non-muscle myosin heavy chain IIa) (NMMHC II-a) (NMMHC-IIA)
O00159	Unconventional myosin-Ic (Myosin I beta) (MMI-beta) (MMIb)
Q9UM54	Unconventional myosin-VI (Unconventional myosin-6)
Q9NZM1	Myoferlin (Fer-1-like protein 3)
P43490	Nicotinamide phosphoribosyltransferase (NAmPRTase) (Nampt) (EC 2.4.2.12) (Pre-B-cell colony-enhancing factor 1) (Pre-B cell-enhancing factor) (Visfatin)
Q99733	Nucleosome assembly protein 1-like 4 (Nucleosome assembly protein 2) (NAP-2)
Q14112	Nidogen-2 (NID-2) (Osteonidogen)
P55786	Puromycin-sensitive aminopeptidase (PSA) (EC 3.4.11.14) (Cytosol alanyl aminopeptidase) (AAP-S)
P06748	Nucleophosmin (NPM) (Nucleolar phosphoprotein B23) (Nucleolar protein NO38) (Numatrin)
P46459	Vesicle-fusing ATPase (EC 3.6.4.6) (N-ethylmaleimide-sensitive fusion protein) (NEM-sensitive fusion protein) (Vesicular-fusion protein NSF)
Q9UNZ2	NSFL1 cofactor p47 (UBX domain-containing protein 2C) (p97 cofactor p47)
Q02818	Nucleobindin-1 (CALNUC)
Q14980	Nuclear mitotic apparatus protein 1 (Nuclear matrix protein-22) (NMP-22) (Nuclear mitotic apparatus protein) (NuMA protein) (SP-H antigen)
P20774	Mimecan (Osteoglycin) (Osteoinductive factor) (OIF)
Q9NTK5	Obg-like ATPase 1 (DNA damage-regulated overexpressed in cancer 45) (DOC45) (GTP-binding protein 9)
Q96FW1	Ubiquitin thioesterase OTUB1 (EC 3.4.19.12) (Deubiquitinating enzyme OTUB1) (OTU domain-containing ubiquitin aldehyde-binding protein 1) (Otubain-1) (hOTU1) (Ubiquitin-specific-processing protease OTUB1)
P07237	Protein disulfide-isomerase (PDI) (EC 5.3.4.1) (Cellular thyroid hormone-binding protein) (Prolyl 4-hydroxylase subunit beta) (p55)
Q9UQ80	Proliferation-associated protein 2G4 (Cell cycle protein p38-2G4 homolog) (hG4-1) (ErbB3-binding protein 1)
P11940	Polyadenylate-binding protein 1 (PABP-1) (Poly(A)-binding protein 1)
Q99497	Protein/nucleic acid deglycase DJ-1 (EC 3.1.2.-) (EC 3.5.1.-) (EC 3.5.1.124) (Maillard deglycase) (Oncogene DJ1) (Parkinson disease protein 7) (Parkinsonism-associated deglycase) (Protein DJ-1) (DJ-1)

P09874	Poly [ADP-ribose] polymerase 1 (PARP-1) (EC 2.4.2.30) (ADP-ribosyltransferase diphtheria toxin-like 1) (ARTD1) (DNA ADP-ribosyltransferase PARP1) (EC 2.4.2.-) (NAD(+) ADP-ribosyltransferase 1) (ADPRT 1) (Poly[ADP-ribose] synthase 1) (Protein poly-ADP-ribosyltransferase PARP1) (EC 2.4.2.-)
P22061	Protein-L-isoaspartate(D-aspartate) O-methyltransferase (PIMT) (EC 2.1.1.77) (L-isoaspartyl protein carboxyl methyltransferase) (Protein L-isoaspartyl/D-aspartyl methyltransferase) (Protein-beta-aspartate methyltransferase)
Q9UHG3	Prenylcysteine oxidase 1 (EC 1.8.3.5) (Prenylcysteine lyase)
Q8WUM4	Programmed cell death 6-interacting protein (PDCD6-interacting protein) (ALG-2-interacting protein 1) (ALG-2-interacting protein X) (Hp95)
P08559	Pyruvate dehydrogenase E1 component subunit alpha, somatic form, mitochondrial (EC 1.2.4.1) (PDHE1-A type I)
P11177	Pyruvate dehydrogenase E1 component subunit beta, mitochondrial (PDHE1-B) (EC 1.2.4.1)
P30101	Protein disulfide-isomerase A3 (EC 5.3.4.1) (58 kDa glucose-regulated protein) (58 kDa microsomal protein) (p58) (Disulfide isomerase ER-60) (Endoplasmic reticulum resident protein 57) (ER protein 57) (ERp57) (Endoplasmic reticulum resident protein 60) (ER protein 60) (ERp60)
P13667	Protein disulfide-isomerase A4 (EC 5.3.4.1) (Endoplasmic reticulum resident protein 70) (ER protein 70) (ERp70) (Endoplasmic reticulum resident protein 72) (ER protein 72) (ERp-72) (ERp72)
Q15084	Protein disulfide-isomerase A6 (EC 5.3.4.1) (Endoplasmic reticulum protein 5) (ER protein 5) (ERp5) (Protein disulfide isomerase P5) (Thioredoxin domain-containing protein 7)
Q96HC4	PDZ and LIM domain protein 5 (Enigma homolog) (Enigma-like PDZ and LIM domains protein)
Q6P996	Pyridoxal-dependent decarboxylase domain-containing protein 1 (EC 4.1.1.-)
P30086	Phosphatidylethanolamine-binding protein 1 (PEBP-1) (HCNPpp) (Neuropolypeptide h3) (Prostatic-binding protein) (Raf kinase inhibitor protein) (RKIP) [Cleaved into: Hippocampal cholinergic neurostimulating peptide (HCNP)]
P17858	ATP-dependent 6-phosphofructokinase, liver type (ATP-PFK) (PFK-L) (EC 2.7.1.11) (6-phosphofructokinase type B) (Phosphofructo-1-kinase isozyme B) (PFK-B) (Phosphohexokinase)
P07737	Profilin-1 (Epididymis tissue protein Li 184a) (Profilin I)
P52209	6-phosphogluconate dehydrogenase, decarboxylating (EC 1.1.1.44)
P00558	Phosphoglycerate kinase 1 (EC 2.7.2.3) (Cell migration-inducing gene 10 protein) (Primer recognition protein 2) (PRP 2)
P36871	Phosphoglucomutase-1 (PGM 1) (EC 5.4.2.2) (Glucose phosphomutase 1)
Q96G03	Phosphoglucomutase-2 (PGM 2) (EC 5.4.2.2) (Glucose phosphomutase 2) (Phosphodeoxyribomutase) (Phosphopentomutase) (EC 5.4.2.7)
P35232	Prohibitin
Q15149	Plectin (PCN) (PLTN) (Hemidesmosomal protein 1) (HD1) (Plectin-1)

P29590	Protein PML (Promyelocytic leukemia protein) (RING finger protein 71) (Tripartite motif-containing protein 19)
Q15063	Periostin (PN) (Osteoblast-specific factor 2) (OSF-2)
Q15181	Inorganic pyrophosphatase (EC 3.6.1.1) (Pyrophosphate phospho-hydrolase) (PPase)
P23284	Peptidyl-prolyl cis-trans isomerase B (PPIase B) (EC 5.2.1.8) (CYP-S1) (Cyclophilin B) (Rotamase B) (S-cyclophilin) (SCYLP)
P62140	Serine/threonine-protein phosphatase PP1-beta catalytic subunit (PP-1B) (PPP1CD) (EC 3.1.3.16) (EC 3.1.3.53)
O14974	Protein phosphatase 1 regulatory subunit 12A (Myosin phosphatase-targeting subunit 1) (Myosin phosphatase target subunit 1) (Protein phosphatase myosin-binding subunit)
Q15435	Protein phosphatase 1 regulatory subunit 7 (Protein phosphatase 1 regulatory subunit 22)
P30153	Serine/threonine-protein phosphatase 2A 65 kDa regulatory subunit A alpha isoform (Medium tumor antigen-associated 61 kDa protein) (PP2A subunit A isoform PR65-alpha) (PP2A subunit A isoform R1-alpha)
Q06830	Peroxiredoxin-1 (EC 1.11.1.15) (Natural killer cell-enhancing factor A) (NKEF-A) (Proliferation-associated gene protein) (PAG) (Thioredoxin peroxidase 2) (Thioredoxin-dependent peroxide reductase 2)
P32119	Peroxiredoxin-2 (EC 1.11.1.15) (Natural killer cell-enhancing factor B) (NKEF-B) (PRP) (Thiol-specific antioxidant protein) (TSA) (Thioredoxin peroxidase 1) (Thioredoxin-dependent peroxide reductase 1)
P30048	Thioredoxin-dependent peroxide reductase, mitochondrial (EC 1.11.1.15) (Antioxidant protein 1) (AOP-1) (HBC189) (Peroxiredoxin III) (Prx-III) (Peroxiredoxin-3) (Protein MER5 homolog)
Q13162	Peroxiredoxin-4 (EC 1.11.1.15) (Antioxidant enzyme AOE372) (AOE37-2) (Peroxiredoxin IV) (Prx-IV) (Thioredoxin peroxidase AO372) (Thioredoxin-dependent peroxide reductase A0372)
P30044	Peroxiredoxin-5, mitochondrial (EC 1.11.1.15) (Alu corepressor 1) (Antioxidant enzyme B166) (AOEB166) (Liver tissue 2D-page spot 71B) (PLP) (Peroxiredoxin V) (Prx-V) (Peroxisomal antioxidant enzyme) (TPx type VI) (Thioredoxin peroxidase PMP20)
P30041	Peroxiredoxin-6 (EC 1.11.1.15) (1-Cys peroxiredoxin) (1-Cys PRX) (24 kDa protein) (Acidic calcium-independent phospholipase A2) (aiPLA2) (EC 3.1.1.4) (Antioxidant protein 2) (Liver 2D page spot 40) (Non-selenium glutathione peroxidase) (NSGPx) (Red blood cells page spot 12)
P51888	Prolargin (Proline-arginine-rich end leucine-rich repeat protein)
P10644	cAMP-dependent protein kinase type I-alpha regulatory subunit (Tissue-specific extinguisher 1) (TSE1)
P13861	cAMP-dependent protein kinase type II-alpha regulatory subunit
P14314	Glucosidase 2 subunit beta (80K-H protein) (Glucosidase II subunit beta) (Protein kinase C substrate 60.1 kDa protein heavy chain) (PKCSH)

P25786	Proteasome subunit alpha type-1 (EC 3.4.25.1) (30 kDa prosomal protein) (PROS-30) (Macropain subunit C2) (Multicatalytic endopeptidase complex subunit C2) (Proteasome component C2) (Proteasome nu chain)
P25788	Proteasome subunit alpha type-3 (EC 3.4.25.1) (Macropain subunit C8) (Multicatalytic endopeptidase complex subunit C8) (Proteasome component C8)
P28066	Proteasome subunit alpha type-5 (EC 3.4.25.1) (Macropain zeta chain) (Multicatalytic endopeptidase complex zeta chain) (Proteasome zeta chain)
O14818	Proteasome subunit alpha type-7 (EC 3.4.25.1) (Proteasome subunit RC6-1) (Proteasome subunit XAPC7)
P62191	26S proteasome regulatory subunit 4 (P26s4) (26S proteasome AAA-ATPase subunit RPT2) (Proteasome 26S subunit ATPase 1)
P35998	26S proteasome regulatory subunit 7 (26S proteasome AAA-ATPase subunit RPT1) (Proteasome 26S subunit ATPase 2) (Protein MSS1)
P17980	26S proteasome regulatory subunit 6A (26S proteasome AAA-ATPase subunit RPT5) (Proteasome 26S subunit ATPase 3) (Proteasome subunit P50) (Tat-binding protein 1) (TBP-1)
P62195	26S proteasome regulatory subunit 8 (26S proteasome AAA-ATPase subunit RPT6) (Proteasome 26S subunit ATPase 5) (Proteasome subunit p45) (Thyroid hormone receptor-interacting protein 1) (TRIP1) (p45/SUG)
O00231	26S proteasome non-ATPase regulatory subunit 11 (26S proteasome regulatory subunit RPN6) (26S proteasome regulatory subunit S9) (26S proteasome regulatory subunit p44.5)
Q13200	26S proteasome non-ATPase regulatory subunit 2 (26S proteasome regulatory subunit RPN1) (26S proteasome regulatory subunit S2) (26S proteasome subunit p97) (Protein 55.11) (Tumor necrosis factor type 1 receptor-associated protein 2)
O43242	26S proteasome non-ATPase regulatory subunit 3 (26S proteasome regulatory subunit RPN3) (26S proteasome regulatory subunit S3) (Proteasome subunit p58)
Q15008	26S proteasome non-ATPase regulatory subunit 6 (26S proteasome regulatory subunit RPN7) (26S proteasome regulatory subunit S10) (Breast cancer-associated protein SGA-113M) (Phosphonoformate immuno-associated protein 4) (Proteasome regulatory particle subunit p44S10) (p42A)
Q06323	Proteasome activator complex subunit 1 (11S regulator complex subunit alpha) (REG-alpha) (Activator of multicatalytic protease subunit 1) (Interferon gamma up-regulated I-5111 protein) (IGUP I-5111) (Proteasome activator 28 subunit alpha) (PA28a) (PA28alpha)
Q9UL46	Proteasome activator complex subunit 2 (11S regulator complex subunit beta) (REG-beta) (Activator of multicatalytic protease subunit 2) (Proteasome activator 28 subunit beta) (PA28b) (PA28beta)
P26599	Polypyrimidine tract-binding protein 1 (PTB) (57 kDa RNA-binding protein PPTB-1) (Heterogeneous nuclear ribonucleoprotein I) (hnRNP I)

P11216	Glycogen phosphorylase, brain form (EC 2.4.1.1)
P61026	Ras-related protein Rab-10
P61019	Ras-related protein Rab-2A
P51149	Ras-related protein Rab-7a
Q9UKM9	RNA-binding protein Raly (Autoantigen p542) (Heterogeneous nuclear ribonucleoprotein C-like 2) (hnRNP core protein C-like 2) (hnRNP associated with lethal yellow protein homolog)
P38159	RNA-binding motif protein, X chromosome (Glycoprotein p43) (Heterogeneous nuclear ribonucleoprotein G) (hnRNP G) [Cleaved into: RNA-binding motif protein, X chromosome, N-terminally processed]
Q15293	Reticulocalbin-1
P27635	60S ribosomal protein L10 (Laminin receptor homolog) (Large ribosomal subunit protein uL16) (Protein QM) (Ribosomal protein L10) (Tumor suppressor QM)
P62906	60S ribosomal protein L10a (CSA-19) (Large ribosomal subunit protein uL1) (Neural precursor cell expressed developmentally down-regulated protein 6) (NEDD-6)
Q07020	60S ribosomal protein L18 (Large ribosomal subunit protein eL18)
P36578	60S ribosomal protein L4 (60S ribosomal protein L1) (Large ribosomal subunit protein uL4)
P05388	60S acidic ribosomal protein P0 (60S ribosomal protein L10E) (Large ribosomal subunit protein uL10)
P05387	60S acidic ribosomal protein P2 (Large ribosomal subunit protein P2) (Renal carcinoma antigen NY-REN-44)
P04843	Dolichyl-diphosphooligosaccharide--protein glycosyltransferase subunit 1 (Dolichyl-diphosphooligosaccharide--protein glycosyltransferase 67 kDa subunit) (Ribophorin I) (RPN-I) (Ribophorin-1)
P25398	40S ribosomal protein S12 (Small ribosomal subunit protein eS12)
P15880	40S ribosomal protein S2 (40S ribosomal protein S4) (Protein LLRep3) (Small ribosomal subunit protein uS5)
P62857	40S ribosomal protein S28 (Small ribosomal subunit protein eS28)
P23396	40S ribosomal protein S3 (EC 4.2.99.18) (Small ribosomal subunit protein uS3)
P61247	40S ribosomal protein S3a (Small ribosomal subunit protein eS1) (v-fos transformation effector protein) (Fte-1)
P62701	40S ribosomal protein S4, X isoform (SCR10) (Single copy abundant mRNA protein) (Small ribosomal subunit protein eS4)
P62241	40S ribosomal protein S8 (Small ribosomal subunit protein eS8)
P46781	40S ribosomal protein S9 (Small ribosomal subunit protein uS4)
Q9NQC3	Reticulon-4 (Foocen) (Neurite outgrowth inhibitor) (Nogo protein) (Neuroendocrine-specific protein) (NSP) (Neuroendocrine-specific protein C homolog) (RTN-x) (Reticulon-5)

Q9Y230	RuvB-like 2 (EC 3.6.4.12) (48 kDa TATA box-binding protein-interacting protein) (48 kDa TBP-interacting protein) (51 kDa erythrocyte cytosolic protein) (ECP-51) (INO80 complex subunit J) (Repressing pontin 52) (Reptin 52) (TIP49b) (TIP60-associated protein 54-beta) (TAP54-beta)
P60903	Protein S100-A10 (Calpactin I light chain) (Calpactin-1 light chain) (Cellular ligand of annexin II) (S100 calcium-binding protein A10) (p10 protein) (p11)
P31949	Protein S100-A11 (Calgizzarin) (Metastatic lymph node gene 70 protein) (MLN 70) (Protein S100-C) (S100 calcium-binding protein A11) [Cleaved into: Protein S100-A11, N-terminally processed]
P05109	Protein S100-A8 (Calgranulin-A) (Calprotectin L1L subunit) (Cystic fibrosis antigen) (CFAG) (Leukocyte L1 complex light chain) (Migration inhibitory factor-related protein 8) (MRP-8) (p8) (S100 calcium-binding protein A8) (Urinary stone protein band A)
P06702	Protein S100-A9 (Calgranulin-B) (Calprotectin L1H subunit) (Leukocyte L1 complex heavy chain) (Migration inhibitory factor-related protein 14) (MRP-14) (p14) (S100 calcium-binding protein A9)
O75396	Vesicle-trafficking protein SEC22b (ER-Golgi SNARE of 24 kDa) (ERS-24) (ERS24) (SEC22 vesicle-trafficking protein homolog B) (SEC22 vesicle-trafficking protein-like 1)
O94979	Protein transport protein Sec31A (ABP125) (ABP130) (SEC31-like protein 1) (SEC31-related protein A) (Web1-like protein)
Q13228	Methanethiol oxidase (MTO) (EC 1.8.3.4) (56 kDa selenium-binding protein) (SBP56) (SP56) (Selenium-binding protein 1)
Q8NC51	Plasminogen activator inhibitor 1 RNA-binding protein (PAI1 RNA-binding protein 1) (PAI-RBP1) (SERPINE1 mRNA-binding protein 1)
P01009	Alpha-1-antitrypsin (Alpha-1 protease inhibitor) (Alpha-1-antiproteinase) (Serpin A1) [Cleaved into: Short peptide from AAT (SPAAT)]
P01011	Alpha-1-antichymotrypsin (ACT) (Cell growth-inhibiting gene 24/25 protein) (Serpin A3) [Cleaved into: Alpha-1-antichymotrypsin His-Pro-less]
P30740	Leukocyte elastase inhibitor (LEI) (Monocyte/neutrophil elastase inhibitor) (EI) (M/NEI) (Peptidase inhibitor 2) (PI-2) (Serpin B1)
P50454	Serpin H1 (47 kDa heat shock protein) (Arsenic-transactivated protein 3) (AsTP3) (Cell proliferation-inducing gene 14 protein) (Collagen-binding protein) (Colligin) (Rheumatoid arthritis-related antigen RA-A47)
Q01105	Protein SET (HLA-DR-associated protein II) (Inhibitor of granzyme A-activated DNase) (IGAAD) (PHAPII) (Phosphatase 2A inhibitor I2PP2A) (I-2PP2A) (Template-activating factor I) (TAF-I)
Q15459	Splicing factor 3A subunit 1 (SF3a120) (Spliceosome-associated protein 114) (SAP 114)
P31947	14-3-3 protein sigma (Epithelial cell marker protein 1) (Stratifin)

P23246	Splicing factor, proline- and glutamine-rich (100 kDa DNA-pairing protein) (hPOMp100) (DNA-binding p52/p100 complex, 100 kDa subunit) (Polypyrimidine tract-binding protein-associated-splicing factor) (PSF) (PTB-associated-splicing factor)
O75368	SH3 domain-binding glutamic acid-rich-like protein
P11166	Solute carrier family 2, facilitated glucose transporter member 1 (Glucose transporter type 1, erythrocyte/brain) (GLUT-1) (HepG2 glucose transporter)
O14745	Na(+)/H(+) exchange regulatory cofactor NHE-RF1 (NHERF-1) (Ezrin-radixin-moesin-binding phosphoprotein 50) (EBP50) (Regulatory cofactor of Na(+)/H(+) exchanger) (Sodium-hydrogen exchanger regulatory factor 1) (Solute carrier family 9 isoform A3 regulatory factor 1)
Q7KZF4	Staphylococcal nuclease domain-containing protein 1 (EC 3.1.31.1) (100 kDa coactivator) (EBNA2 coactivator p100) (Tudor domain-containing protein 11) (p100 co-activator)
O75643	U5 small nuclear ribonucleoprotein 200 kDa helicase (EC 3.6.4.13) (Activating signal cointegrator 1 complex subunit 3-like 1) (BRR2 homolog) (U5 snRNP-specific 200 kDa protein) (U5-200KD)
Q9BX66	Sorbin and SH3 domain-containing protein 1 (Ponsin) (SH3 domain protein 5) (SH3P12) (c-Cbl-associated protein) (CAP)
Q00796	Sorbitol dehydrogenase (SDH) (EC 1.1.1.-) ((R,R)-butanediol dehydrogenase) (EC 1.1.1.4) (L-iditol 2-dehydrogenase) (EC 1.1.1.14) (Polyol dehydrogenase) (Ribitol dehydrogenase) (RDH) (EC 1.1.1.56) (Xylitol dehydrogenase) (XDH) (EC 1.1.1.9)
Q13813	Spectrin alpha chain, non-erythrocytic 1 (Alpha-II spectrin) (Fodrin alpha chain) (Spectrin, non-erythroid alpha subunit)
Q01082	Spectrin beta chain, non-erythrocytic 1 (Beta-II spectrin) (Fodrin beta chain) (Spectrin, non-erythroid beta chain 1)
P30626	Sorcin (22 kDa protein) (CP-22) (CP22) (V19)
P05455	Lupus La protein (La autoantigen) (La ribonucleoprotein) (Sjoegren syndrome type B antigen) (SS-B)
P42224	Signal transducer and activator of transcription 1-alpha/beta (Transcription factor ISGF-3 components p91/p84)
P27105	Erythrocyte band 7 integral membrane protein (Protein 7.2b) (Stomatin)
P53999	Activated RNA polymerase II transcriptional coactivator p15 (Positive cofactor 4) (PC4) (SUB1 homolog) (p14)
O60506	Heterogeneous nuclear ribonucleoprotein Q (hnRNP Q) (Glycine- and tyrosine-rich RNA-binding protein) (GRY-RBP) (NS1-associated protein 1) (Synaptotagmin-binding, cytoplasmic RNA-interacting protein)
Q01995	Transgelin (22 kDa actin-binding protein) (Protein WS3-10) (Smooth muscle protein 22-alpha) (SM22-alpha)
P37802	Transgelin-2 (Epididymis tissue protein Li 7e) (SM22-alpha homolog)

P37837	Transaldolase (EC 2.2.1.2)
P17987	T-complex protein 1 subunit alpha (TCP-1-alpha) (CCT-alpha)
P02787	Serotransferrin (Transferrin) (Beta-1 metal-binding globulin) (Siderophilin)
Q15582	Transforming growth factor-beta-induced protein ig-h3 (Beta ig-h3) (Kerato-epithelin) (RGD-containing collagen-associated protein) (RGD-CAP)
P21980	Protein-glutamine gamma-glutamyltransferase 2 (EC 2.3.2.13) (Tissue transglutaminase) (Transglutaminase C) (TG(C)) (TGC) (TGase C) (Transglutaminase H) (TGase H) (Transglutaminase-2) (TGase-2)
P07996	Thrombospondin-1 (Glycoprotein G)
Q07157	Tight junction protein ZO-1 (Tight junction protein 1) (Zona occludens protein 1) (Zonula occludens protein 1)
P29401	Transketolase (TK) (EC 2.2.1.1)
Q9Y490	Talin-1
Q9NYL9	Tropomodulin-3 (Ubiquitous tropomodulin) (U-Tmod)
P62328	Thymosin beta-4 (T beta-4) (Fx) [Cleaved into: Hematopoietic system regulatory peptide (Seraspenide)]
P24821	Tenascin (TN) (Cytotactin) (GMEM) (GP 150-225) (Glioma-associated-extracellular matrix antigen) (Hexabrachion) (JI) (Myotendinous antigen) (Neuronectin) (Tenascin-C) (TN-C)
Q9C0C2	182 kDa tankyrase-1-binding protein
P22105	Tenascin-X (TN-X) (Hexabrachion-like protein)
P09493	Tropomyosin alpha-1 chain (Alpha-tropomyosin) (Tropomyosin-1)
P07951	Tropomyosin beta chain (Beta-tropomyosin) (Tropomyosin-2)
P06753	Tropomyosin alpha-3 chain (Gamma-tropomyosin) (Tropomyosin-3) (Tropomyosin-5) (hTM5)
P67936	Tropomyosin alpha-4 chain (TM30p1) (Tropomyosin-4)
P12270	Nucleoprotein TPR (Megator) (NPC-associated intranuclear protein) (Translocated promoter region protein)
Q13263	Transcription intermediary factor 1-beta (TIF1-beta) (E3 SUMO-protein ligase TRIM28) (EC 2.3.2.27) (KRAB-associated protein 1) (KAP-1) (KRAB-interacting protein 1) (KRIP-1) (Nuclear corepressor KAP-1) (RING finger protein 96) (RING-type E3 ubiquitin transferase TIF1-beta) (Tripartite motif-containing protein 28)
Q15631	Translin (EC 3.1.-.-) (Component 3 of promoter of RISC) (C3PO)
Q16762	Thiosulfate sulfurtransferase (EC 2.8.1.1) (Rhodanese)
Q13630	GDP-L-fucose synthase (EC 1.1.1.271) (GDP-4-keto-6-deoxy-D-mannose-3,5-epimerase-4-reductase) (Protein FX) (Red cell NADP(H)-binding protein) (Short-chain dehydrogenase/reductase family 4E member 1)

P68366	Tubulin alpha-4A chain (Alpha-tubulin 1) (Testis-specific alpha-tubulin) (Tubulin H2-alpha) (Tubulin alpha-1 chain)
P07437	Tubulin beta chain (Tubulin beta-5 chain)
Q13885	Tubulin beta-2A chain (Tubulin beta class IIa)
P49411	Elongation factor Tu, mitochondrial (EF-Tu) (P43)
Q8NBS9	Thioredoxin domain-containing protein 5 (Endoplasmic reticulum resident protein 46) (ER protein 46) (ERp46) (Thioredoxin-like protein p46)
O43396	Thioredoxin-like protein 1 (32 kDa thioredoxin-related protein)
Q16881	Thioredoxin reductase 1, cytoplasmic (TR) (EC 1.8.1.9) (Gene associated with retinoic and interferon-induced mortality 12 protein) (GRIM-12) (Gene associated with retinoic and IFN-induced mortality 12 protein) (KM-102-derived reductase-like factor) (Thioredoxin reductase TR1)
P19971	Thymidine phosphorylase (TP) (EC 2.4.2.4) (Gliostatin) (Platelet-derived endothelial cell growth factor) (PD-ECGF) (TdRPase)
P22314	Ubiquitin-like modifier-activating enzyme 1 (EC 6.2.1.45) (Protein A1S9) (Ubiquitin-activating enzyme E1)
Q16851	UTP--glucose-1-phosphate uridylyltransferase (EC 2.7.7.9) (UDP-glucose pyrophosphorylase) (UDPGP) (UGPase)
O60763	General vesicular transport factor p115 (Protein USO1 homolog) (Transcytosis-associated protein) (TAP) (Vesicle-docking protein)
P54578	Ubiquitin carboxyl-terminal hydrolase 14 (EC 3.4.19.12) (Deubiquitinating enzyme 14) (Ubiquitin thioesterase 14) (Ubiquitin-specific-processing protease 14)
Q99536	Synaptic vesicle membrane protein VAT-1 homolog (EC 1.-.-.-)
P13611	Versican core protein (Chondroitin sulfate proteoglycan core protein 2) (Chondroitin sulfate proteoglycan 2) (Glial hyaluronate-binding protein) (GHAP) (Large fibroblast proteoglycan) (PG-M)
P18206	Vinculin (Metavinculin) (MV)
P55072	Transitional endoplasmic reticulum ATPase (TER ATPase) (EC 3.6.4.6) (15S Mg(2+)-ATPase p97 subunit) (Valosin-containing protein) (VCP)
P08670	Vimentin
O75436	Vacuolar protein sorting-associated protein 26A (Vesicle protein sorting 26A) (hVPS26)
Q96QK1	Vacuolar protein sorting-associated protein 35 (hVPS35) (Maternal-embryonic 3) (Vesicle protein sorting 35)
P04004	Vitronectin (VN) (S-protein) (Serum-spreading factor) (V75) [Cleaved into: Vitronectin V65 subunit; Vitronectin V10 subunit; Somatomedin-B]
O14980	Exportin-1 (Exp1) (Chromosome region maintenance 1 protein homolog)

P13010	X-ray repair cross-complementing protein 5 (EC 3.6.4.-) (86 kDa subunit of Ku antigen) (ATP-dependent DNA helicase 2 subunit 2) (ATP-dependent DNA helicase II 80 kDa subunit) (CTC box-binding factor 85 kDa subunit) (CTC85) (CTCBF) (DNA repair protein XRCC5) (Ku80) (Ku86) (Lupus Ku autoantigen protein p86) (Nuclear factor IV) (Thyroid-lupus autoantigen) (TLAA) (X-ray repair complementing defective repair in Chinese hamster cells 5 (double-strand-break rejoining))
P12956	X-ray repair cross-complementing protein 6 (EC 3.6.4.-) (EC 4.2.99.-) (5'-deoxyribose-5-phosphate lyase Ku70) (5'-dRP lyase Ku70) (70 kDa subunit of Ku antigen) (ATP-dependent DNA helicase 2 subunit 1) (ATP-dependent DNA helicase II 70 kDa subunit) (CTC box-binding factor 75 kDa subunit) (CTC75) (CTCBF) (DNA repair protein XRCC6) (Lupus Ku autoantigen protein p70) (Ku70) (Thyroid-lupus autoantigen) (TLAA) (X-ray repair complementing defective repair in Chinese hamster cells 6)
P67809	Y-box-binding protein 1 (YB-1) (CCAAT-binding transcription factor I subunit A) (CBF-A) (DNA-binding protein B) (DBPB) (Enhancer factor I subunit A) (EFI-A) (Nuclease-sensitive element-binding protein 1) (Y-box transcription factor)
P31946	14-3-3 protein beta/alpha (Protein 1054) (Protein kinase C inhibitor protein 1) (KCIP-1) [Cleaved into: 14-3-3 protein beta/alpha, N-terminally processed]
P62258	14-3-3 protein epsilon (14-3-3E)
P61981	14-3-3 protein gamma (Protein kinase C inhibitor protein 1) (KCIP-1) [Cleaved into: 14-3-3 protein gamma, N-terminally processed]
Q04917	14-3-3 protein eta (Protein AS1)
P27348	14-3-3 protein theta (14-3-3 protein T-cell) (14-3-3 protein tau) (Protein HS1)
P63104	14-3-3 protein zeta/delta (Protein kinase C inhibitor protein 1) (KCIP-1)
