# Supplementary material for: Proteomics of REPLICANT perfusate detects changes in the metastatic lymph node microenvironment
Source: NPJ Breast Cancer. 2021 Mar 5;7:24. doi: 10.1038/s41523-021-00227-7 (PMC7935848; doi:10.1038/s41523-021-00227-7)
Supplement: Supplementary file 2 — Reporting Summary Checklist [file 41523_2021_227_MOESM2_ESM.pdf]

nature research

Corresponding author(s): Kalnisha Naidoo

Last updated by author(s): Nov 28, 2020

Reporting Summary

Nature Research wishes to improve the reproducibility of the work that we publish. This form provides structure for consistency and transparency in reporting. For further information on Nature Research policies, see our Editorial Policies and the Editorial Policy Checklist.

Statistics

For all statistical analyses, confirm that the following items are present in the figure legend, table legend, main text, or Methods section.

n/a	Confirmed	
☐	☒	The exact sample size (*n*) for each experimental group/condition, given as a discrete number and unit of measurement
☐	☒	A statement on whether measurements were taken from distinct samples or whether the same sample was measured repeatedly
☐	☒	The statistical test(s) used AND whether they are one- or two-sided<br>*Only common tests should be described solely by name; describe more complex techniques in the Methods section.*
☐	☒	A description of all covariates tested
☐	☒	A description of any assumptions or corrections, such as tests of normality and adjustment for multiple comparisons
☐	☒	A full description of the statistical parameters including central tendency (e.g. means) or other basic estimates (e.g. regression coefficient) AND variation (e.g. standard deviation) or associated estimates of uncertainty (e.g. confidence intervals)
☐	☒	For null hypothesis testing, the test statistic (e.g. *F*, *t*, *r*) with confidence intervals, effect sizes, degrees of freedom and *P* value noted<br>*Give P values as exact values whenever suitable.*
☒	☐	For Bayesian analysis, information on the choice of priors and Markov chain Monte Carlo settings
☐	☒	For hierarchical and complex designs, identification of the appropriate level for tests and full reporting of outcomes
☒	☐	Estimates of effect sizes (e.g. Cohen's *d*, Pearson's *r*), indicating how they were calculated

Our web collection on statistics for biologists contains articles on many of the points above.

Software and code

Policy information about availability of computer code

Data collection	*Provide a description of all commercial, open source and custom code used to collect the data in this study, specifying the version used OR state that no software was used.*
Data analysis	*Provide a description of all commercial, open source and custom code used to analyse the data in this study, specifying the version used OR state that no software was used.*

For manuscripts utilizing custom algorithms or software that are central to the research but not yet described in published literature, software must be made available to editors and reviewers. We strongly encourage code deposition in a community repository (e.g. GitHub). See the Nature Research guidelines for submitting code & software for further information.

Data

Policy information about availability of data

All manuscripts must include a data availability statement. This statement should provide the following information, where applicable:

- Accession codes, unique identifiers, or web links for publicly available datasets
- A list of figures that have associated raw data
- A description of any restrictions on data availability

The proteomics data generated during this study has been deposited to the ProteomeXchange Consortium via the PRIDE69 partner repository (PXD022722). All other data generated or analysed are included herein.

Field-specific reporting

Please select the one below that is the best fit for your research. If you are not sure, read the appropriate sections before making your selection.

☒ Life sciences ☐ Behavioural & social sciences ☐ Ecological, evolutionary & environmental sciences

For a reference copy of the document with all sections, see nature.com/documents/nr-reporting-summary-flat.pdf

Life sciences study design

All studies must disclose on these points even when the disclosure is negative.

Sample size	This study analysed in total 10 human axillary lymph nodes, and perfusate samples collected from these samples. Five lymph nodes were reactive; five contained metastases. This represents the exploratory analysis of a previous study proving viability (Barrow-McGee et al, J Path, 2019). The calculations surrounding sample size determination have thus been published previously.
Data exclusions	One metastatic lymph node, which contained a micrometastasis, was excluded from the analysis.
Replication	Each group comprised more than 3 samples.
Randomization	Samples were allocated to a group based on their inherent biological features, and this could not, therefore be randomised.
Blinding	All samples were anonymised prior to analysis.

Reporting for specific materials, systems and methods

We require information from authors about some types of materials, experimental systems and methods used in many studies. Here, indicate whether each material, system or method listed is relevant to your study. If you are not sure if a list item applies to your research, read the appropriate section before selecting a response.

Materials & experimental systems

n/a	Involved in the study	
☐	☒	Antibodies
☒	☐	Eukaryotic cell lines
☒	☐	Palaeontology and archaeology
☒	☐	Animals and other organisms
☐	☒	Human research participants
☒	☐	Clinical data
☒	☐	Dual use research of concern

Methods

n/a	Involved in the study	
☒	☐	ChIP-seq
☒	☐	Flow cytometry
☒	☐	MRI-based neuroimaging

Antibodies

Antibodies used	The following antibodies were used: CD4 (Abcam 133616; Opal 520), CD8 (Dako, M710301; Opal 570), CD20 (Dako, M075529; Opal 540), PD-L1 (Cell Signalling, 13684; Opal 620), FoxP3 (Abcam, 20034; Opal 650), Pan-cytokeratin (Dako, M351501; Opal 690; on metastatic ALNs only), and CD68 (Dako, M087629; Opal 690 on reactive ALNs only).
Validation	Antibody optimisation had been performed and published previously. Control tissue samples were stained for each marker in parallel.

Human research participants

Policy information about studies involving human research participants

Population characteristics	Samples were harvested from patients with breast cancer, who were undergoing an axillary lymph node dissection as standard of care.
Recruitment	Patients were consented and recruited by surgeons and members of the King's Health Partners (KHP) Biobank. Samples were collected from theatre by Biobank staff, and were anonymised before researchers harvested and perfused them.
Ethics oversight	King's Health Partners Biobank (Research Ethics Committee No: 18/EE/0025)

Note that full information on the approval of the study protocol must also be provided in the manuscript.
